# Enantioseparation of *P*-Stereogenic Secondary Phosphine Oxides and Their Stereospecific Transformation to Various Tertiary Phosphine Oxides and a Thiophosphinate

**DOI:** 10.1021/acs.joc.1c01364

**Published:** 2021-10-11

**Authors:** Bence Varga, Péter Szemesi, Petra Nagy, Réka Herbay, Tamás Holczbauer, Elemér Fogassy, György Keglevich, Péter Bagi

**Affiliations:** † Department of Organic Chemistry and Technology, Budapest University of Technology and Economics, Műegyetem rkp. 3, H-1111 Budapest, Hungary; ⊥ Gedeon Richter Plc., H-1475 Budapest, Hungary; ¶ Center for Structural Science, Chemical Crystallography Research Laboratory and Institute for Organic Chemistry, Research Centre for Natural Sciences, Magyar tudósok körútja 2, H-1519 Budapest, Hungary

## Abstract

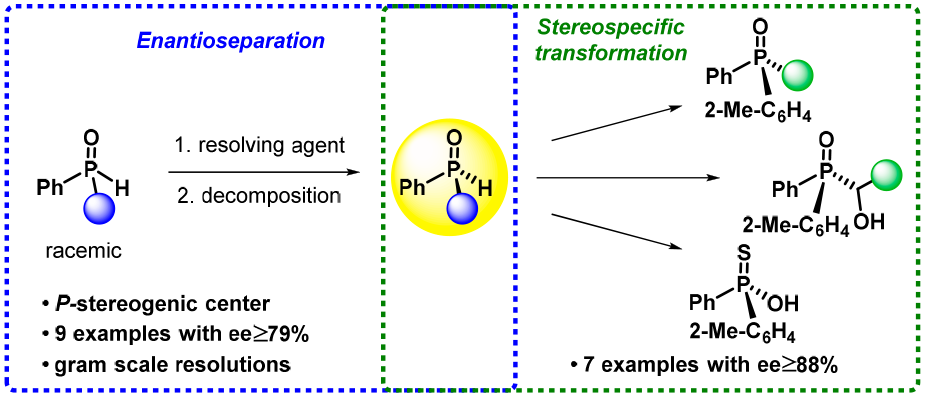

Secondary phosphine oxides incorporating various aryl and alkyl groups were synthesized in racemic form, and these products formed the library reported in this study. TADDOL derivatives were used to obtain the optical resolution of these *P*-stereogenic secondary phosphine oxides. The developed resolution method showed a good scope under the optimized reaction conditions, as 9 out of 14 derivatives could be prepared with an enantiomeric excess (ee) ≥ 79% and 5 of these derivatives were practically enantiopure >P(O)H compounds (ee ≥ 98%). The scalability of this resolution method was also demonstrated. Noncovalent interactions responsible for the formation of diasteromeric complexes were elucidated by single-crystal XRD measurements. (*S*)-(2-Methylphenyl)phenylphosphine oxide was transformed to a variety of *P*-stereogenic tertiary phosphine oxides and a thiophosphinate in stereospecific Michaelis–Becker, Hirao, or Pudovik reactions.

## Introduction

*P*-Stereogenic phosphines, phosphine oxides, or phosphonium salts represent an important class among organophophorus compounds, as these chiral derivatives have found widespread application as ligands,^1^ organocatalysts,^2^ or even biologically active compounds.^3^ The preparation of enantiopure *P*-compounds still represents a challenge, which inhibits their more diverse use. Most of the synthetic methods give the organophosphorus compound of interest in optically active form.^4^ Syntheses involving the preparation of *P*-chiral precursors followed by their incorporation in the desired scaffold represent a modular strategy carrying high synthetic potential. Bench stability, low toxicity, odorless property, and the stereospecific functionalization of their P–H bond make secondary phosphine oxides (SPOs) desired *P*-stereogenic precursors.^5^ Moreover, when the >P(O)H–>P(OH) tautomerization is utilized,^6^ secondary phosphine oxides can be regarded as (pre)ligands, which can be used in transition metal catalyzed asymmetric transformations.^7^

**Figure 1:**
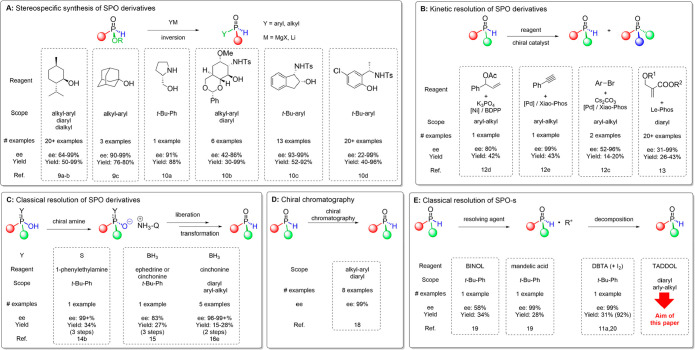
Selected examples showing the main methods for the preparation of *P*-stereogenic secondary phosphine oxides in optically active form (yields were calculated on the basis of the full amount of the racemate).

These properties make *P*-stereogenic SPOs valuable targets, and a variety of stereoselective syntheses or enantioseparation methods have been developed in recent years.^8^ Most of the stereoselective syntheses rely on nucleophilic substitution of enantiopure *H*-phosphinates (Figure 1A). The mode of preparation of the chiral *H*-phosphinate is a key difference between the given methods. Menthyl and later adamantyl phenyl-*H*-phosphinate were among the first simple optically active *H*-phosphinates used for stereoselective synthesis of chiral SPOs. Separation of the corresponding racemic compounds was used for the preparation of optically active *H*-phosphinate starting materials, and these studies also highlighted that undesired racemization may happen during nucleophilic substitution when certain organometallic reagents are used.^9^ Another strategy utilizes amino-alcohols as chiral templates, and hydrolysis of the chiral oxazaphospholidine intermediate gives the corresponding *H*-phosphinates, which are then reacted with organometallic reagents.^10^ Despite the advantages of the stereoselective syntheses, carefully controlled reaction conditions were required for each of the three key steps in order to avoid partial racemization and to obtain secondary phosphine oxides with high enantiomeric purity. In contrast, racemic secondary phosphine oxides can be prepared in just two steps considering the most straightforward synthetic path.^11^

Thus, various separation methods of the corresponding racemates represent alternative methods for the preparation of optically active SPOs. In recent years, a few kinetic resolutions were developed employing chiral catalysts to give various *P*-stereogenic tertiary phosphine oxides with excellent enantiomeric excesses (ee’s).^12^ In theory, such reactions would give the unreacted portion of the SPO with high enanantiomeric purity (Figure 1B). However, optically active SPOs were seldom isolated from the corresponding reaction mixtures,^12c−12e^ and a few mechanistic studies revealed that optically active SPOs may racemize to some extent under the reaction conditions.^12b−12d^ In a recent publication, Zhang and co-workers demonstrated an organocatalytic kinetic resolution using chiral phosphine catalysts. Besides the TPO products, the corresponding optically active SPOs were also isolated in good to excellent ee-s, showing a wide scope among *P*-stereogenic diaryl secondary phosphine oxides.^13^

Classical resolutions, which are based on the formation and separation of crystalline diastereomeric salts or complexes, were among the first few methods developed for the enantioseparation of *P*-stereogenic secondary phosphine oxides. It is noteworthy that *t*-butyl-phenylphosphine oxide was the main focus of those studies. One type of resolution method involves the transformation of SPOs to the corresponding thiophosphinic acids or phosphinous acid-boranes. The enantioseparation of these acidic derivatives was elaborated with chiral bases, and optically active SPOs were obtained after the removal of the sulfur or borane. Haynes et al. used this reaction sequence for the preparation of enantiopure *t*-Bu(Ph)P(O)H in multigram quantities via a thiophosphinic intermediate,^14^ whereas the phosphinous acid-borane route was explored by Stankevič and Pietrusiewicz.^15^ There are a few other reports detailing the resolution of the acidic derivatives of other SPOs, but deprotection is generally omitted in those studies.^16^ These optical resolution methods comprise two additional steps, which potentially lower the yield and increase the risk of undesired racemization (Figure 1C).^15,17^ Enantiomeric separation without derivatization is a more straightforward approach to obtain optically active secondary phosphine oxides. Chromatographic separation using chiral stationary phases was used to prepare the enantiomers of chiral SPOs on a small scale (Figure 1D).^18^ Considering classical resolutions of secondary phosphine oxides, enantiopure *t*-butyl-phenylphosphine oxide could be prepared using mandelic acid or *O*,*O*′-dibenzoyltartaric acid (DBTA) as the resolving agents,^11a,19^ and partial enantiomeric separation was achieved with BINOL^19^ or chalcone sulfonic acid.^11a^ Minnaard and co-workers found that the radical racemization of *t*-Bu(Ph)P(O)H could be promoted using a catalytic amount of iodine. When this racemization process was implemented into the optical resolution with *O*,*O*′-dibenzoyltartaric acid, a dynamic resolution procedure was developed, which afforded nearly full conversion of the racemate to (*R*)-*t*-Bu(Ph)P(O)H with high enantiomeric purity.^20^ Despite these promising results, such direct resolutions have never been extended to other secondary phosphine oxides, leaving the enantiomers of *t*-butyl-phenylphosphine oxide as the sole example prepared by this method (Figure 1E).

This summary shows that renewed interest in *P*-stereogenic SPOs populated especially the stereoselective or kinetic resolution methods, whereas the number of classical resolutions still remains low with limited scope. One of our ongoing interests involves the preparation of chiral organophosphorus compounds, and the high synthetic potential of *P*-stereogenic SPOs prompted us to develop enantiomeric separation methods for this compound class. To date, only tertiary phosphine oxides were made available in optically active form by our resolution methods.^21^ The elaboration of such a method for *P*-stereogenic SPOs is not straightforward, as one of the TPO functional groups capable of the formation of secondary interactions is replaced by a proton, which makes the enantiomeric recognition and the separation of SPOs challenging.

**Figure 2:**
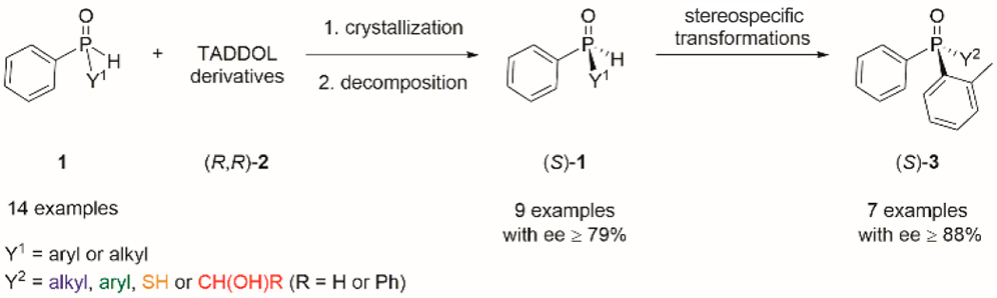
Outline of this research project.

The aim of this research was to develop a versatile enantioseparation method for the preparation of optically active secondary phosphine oxides (**1**). On the one hand, diaryl secondary phosphine oxides (**1a**–**i**) containing substituents in the ortho-, meta-, or para-position were our focus in order to investigate the effect of the substitution pattern on the efficiency of the enantiomeric separation and to increase the number of separation methods for such SPOs. On the other hand, alkyl-aryl derivatives (**1j**–**n**) were also included in this study in order to investigate the general scope of our resolution method. The secondary interactions within the diastereomers were investigated by single crystal X-ray crystallography. Starting from a selected secondary phosphine oxide, the synthesis of various optically active *P*-stereogenic tertiary phosphine oxides and thiophosphinates were attempted using stereospecific transformations (Figure 2).

## Results and Discussion

**Scheme 1:**
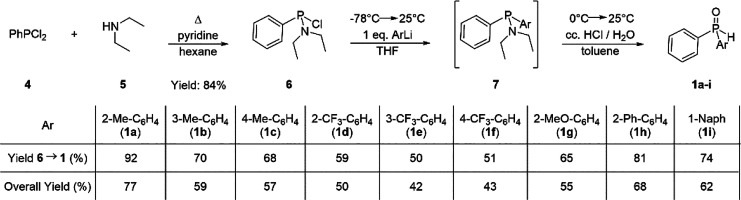
Preparation of Racemic Diaryl Secondary Phosphine Oxides (**1a**–**i**)

A synthetic strategy employing the diethylamino protecting group was selected for the synthesis of the racemic diaryl phosphine oxides (**1a**–**i**) (Scheme 1). First, *P*,*P*-dichlorophenylphosphine (**4**) was reacted with diethylamine, and pure *N*,*N*-diethylamino-chloro-phenylphosphine (**6**) could be isolated by vacuum distillation. *N*,*N*-Diethylamino-chloro-phenylphosphine (**6**) was the key intermediate in our divergent synthetic strategy, and it was reacted with aryllithiums to give the corresponding aminophosphines (**7**). Then, the toluene solution of the protected intermediate (**7**) was treated with cc. HCl (aq.) in order to remove the diethylamino group and to facilitate hydrolysis to give the corresponding secondary phosphine oxides (**1a**–**i**). The overall yield of this reaction sequence (**1a**–**i**) was in between 42% and 77% after a final purification by column chromatography. This one pot strategy for the aminophosphine SPO transformation was first demonstrated by Hoge et al.,^22^ and it has advantages over other literature procedures in which the removal of the protecting group and the hydrolysis of the intermediate are performed in two separate steps.^23^ The racemic alkyl-aryl secondary phosphine oxides (**1j**–**n**) were prepared according to a literature procedure by reacting PhPCl_2_ with ethanol and treating the *in situ* formed ethyl phenyl-*H*-phosphinate with the corresponding organometallic reagent.^12b,24^

**Scheme 2:**
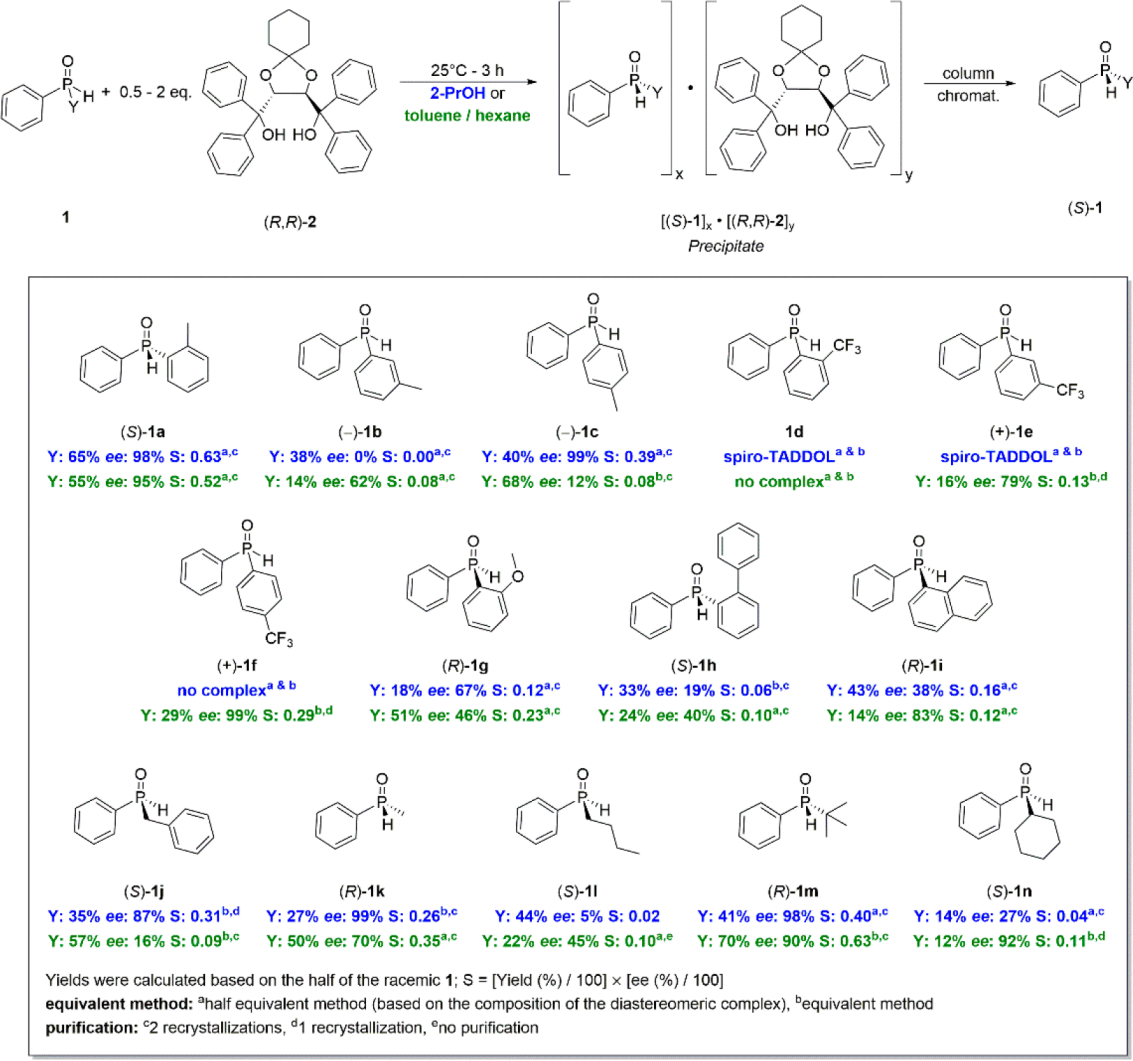
Summary of the Best Results for the Resolution of *P*-Stereogenic Secondary Phosphine Oxides (**1**) with (*R*,*R*)-spiro-TADDOL [(*R*,*R*)-**2**]

With the racemic secondary phosphine oxides (**1**) in hand, we began our investigation to find a suitable resolving agent. Our previous research^21a−21c^ suggested that TADDOL derivatives might be good candidates for the optical resolution of the target secondary phosphine oxides (**1**). From our SPO library, the (2-methylphenyl)-phenylphosphine oxide (**1a**) was selected as the model racemic compound, and its resolution was attempted by various TADDOL derivatives and in a variety of solvents or solvent mixtures selected on the basis of our previous study (see the Supporting Information for details) (Scheme 2).^21c^ Even the first few experiments suggested that spiro-TADDOL [(*R*,*R*)-**2**] is the most suitable resolving agent, as a change either in the aromatic groups or in the dioxolane ring of the TADDOL scaffold led to a significant decrease in the enantioseparation of secondary phosphine oxide **1a**. Considering the solvents and the crystallization techniques, a precipitation-mediated crystallization from a mixture of toluene and hexane and a “classical” crystallization from 2-PrOH were the two methods that showed the most promising results affording the (*S*)-(2-methylphenyl)-phenylphosphine oxide [(*S*)-**1a**] in an ee above 95%. The other solvents investigated gave significantly lower ee values and yields (see the Supporting Information for details).

Considering the scope of the secondary phosphine oxides (**1**), diaryl SPOs incorporating either a moderately electron donating methyl group or a strong electron withdrawing trifluoromethyl group in the ortho-, meta-, or para-position were investigated (**1a**–**f**). Moreover, 1-naphthyl-, 2-methoxyphenyl, and (2-phenylphenyl)-phenylphosphine oxides (**1g**–**i**) were also included in the study. Considering the alkyl-phenylphospine oxide derivatives (**1j**–**n**), normal, branched, and cycloalkyl groups along with the benzyl group were selected in order to represent a variety of alkyl chains.

On the basis of the results of the preliminary studies, the optical resolution of all target secondary phosphine oxides (**1**) was attempted with spiro-TADDOL [(*R*,*R*)-**2**] using either 2-propanol or a mixture of toluene and hexane as the solvent. It was found that even traces of water in the solvent cause inconsistent results; for this, dry solvents were used during the crystallization of the diastereomers. In all resolution experiments, the diastereomeric complexes were purified by recrystallization(s) in order to increase the diastereomeric purity. The given enantiomerically enriched secondary phosphine oxide (**1**) was liberated from the diastereomeric complexes by column chromatography, and the resolving agent [(*R*,*R*)-**2**] could also be recycled. Our previous study showed that organic solvent nanofiltration could be a scalable alternative process of such decompositions and recycling.^21c^ Most of the resolution experiments were attempted according to half-equivalent and equivalent methods in order to investigate which separation method is the more effective one. Thus, the amount of spiro-TADDOL [(*R*,*R*)-**2**] varied between 0.5 and 2 equiv, depending on the composition of the diastereomeric complex formed (*vide infra*). Results are summarized in Scheme 2 (see the Supporting Information for the complete set of experimental data).

The results indicated that the position and the nature of the substituent significantly influence the efficiency of the enantiomeric separation. Beside (2-methylphenyl)-phenylphosphine oxide (**1a**), the (4-methylphenyl) and (4-trifluoromethylphenyl) derivatives (**1c** and **1f**) could be prepared in enantiopure form (ee: 99%) in yields of 40% or 29%, respectively. Interestingly, the enantiomers of (2-methylphenyl)-phenylphosphine oxide (**1a**) could be separated effectively (ee > 95%, *S* > 0.52) either in 2-propanol or in a mixture of toluene and hexane. On the other hand, only one of the two crystallization methods afforded pure enantiomers for the para-substituted derivatives (**1c** and **1f**). One may conclude that the substituent at the para-position has the least effect on the outcome of the resolution. A para-substituent is not in the proximity of the >P(O)H function; hence, it does not interfere with the mode of binding and noncovalent interactions formed between the given secondary phosphine oxide (**1c** or **1f**) and spiro-TADDOL [(*R*,*R*)-**2**].

In contrast, ortho- and meta-substituents in a phenyl ring have a more decisive role on the overall efficiency of the resolution. 3-Methyl- or (3-trifluoromethyl-phenyl)phenylphosphine oxide (**1b** and **1e**) could be prepared with a maximum ee of 62% or 79%, respectively. The yields were rather low (14–16%), and consequently, the resolving capability values fell in the range of 0.08–0.13. Considering the ortho-substituted derivatives (**1a**, **1d**, **1g**, and **1h**), the change of the methyl group of the racemic compound to a trifluoromethyl, methoxy, or phenyl group led to a decrease in enantiomeric excess (0–67%) and in resolving capability values (0.00–0.23). The (2-trifluoromethylphenyl)-phenylphosphine oxide (**1d**) was the only derivative when the enantiomeric separation was completely unsuccessful, as no diastereomeric complex was formed. One may assume that the trifluoromethyl group being close to the P(O)H group lowers the Lewis basicity of the P=O function; thus, the guest molecule (**1d**) loses its ability to form a stable *H* bond with the resolving agent [(*R*,*R*)-**2**] (*vide infra*). The methoxy group is another H-bond acceptor, whereas the 2-phenylphenyl group significantly increases the steric bulk in the ortho-position. Each effect has a negative impact on the enantiomeric recognition and, consequently, on the efficiency of the resolution (ee = 67% and *S* = 0.12 for **1g**; ee = 40% and *S* = 0.10 for **1h**).

Moderate results were obtained for (1-naphthyl)-phenylphosphine oxide (**1i**) (ee = 38% and *S* = 0.16 or ee = 83% and *S* = 0.12), which was another indication that increased steric demand has a negative impact on the host–guest interactions and, consequently, on the overall efficiency of the resolution. Considering the alkyl-aryl secondary phosphine oxides (**1j**–**n**), the selected derivatives could be resolved with good overall efficiency. Four (**1j**, **1k**, **1m**, and **1n**) out of the five derivatives could be prepared with an ee greater than 87%, and the resolving capability values fell in the range of 0.11–0.63. The (*S*)-methyl- or (*t*-butyl)-phenylphoshine oxide [(*S*)-**1k** or (*S*)-**1m**] were the two derivatives that could be prepared in practically enantiopure form (ee > 98%). These results indicated that the optical resolution with (*R*,*R*)-spiro-TADDOL [(*R*,*R*)-**2**] tolerated normal, cyclo, and aralkyl chains. The butyl-phenylphosphine oxide (**1l**) was the only derivative that could not be separated effectively (ee ≤ 45%; *S* ≤ 0.10). One underlying reason might be that the normal butyl chain cannot form stable second order interactions, whereas these contacts might be more pronounced for the more compact or sterically more demanding (cyclo)alkyl groups. The resolution of a few selected SPOs of special interest, such as the 2-methoxyphenyl-, 1-naphthyl-, and *t*-butyl-derivative (**1g**, **1i**, and **1m**), was also attempted with two additional spiro-TADDOL derivatives. The partial separation of the enantiomers was only successful in the case of the (1-naphthyl)-phenylphosphine oxide (**1i**), but a lower enantiomeric excess value could be obtained with **1i** (ee: 70%) than with the spiro-TADDOL [(*R*,*R*)-**2**] (ee: 83%). These results show that better enantioseparation in this SPO library may not be obtained by changing the structure of the resolving agent (see Table S4 for details).

^1^H NMR studies revealed that the composition of the diastereomeric complexes was dependent on the solvent used. Diastereomers incorporating the given secondary phosphine oxide enantiomer (**1**) and (*R*,*R*)-spiro-TADDOL [(*R*,*R*)-**2**] in a 1:1 ratio were formed in a mixture of toluene and hexane, whereas this SPO–spiro-TADDOL [**1**–[(*R*,*R*)-**2**] ratio changed predominantly to 1:2 when 2-propanol was used as solvent. This change in the composition of the host–guest complexes indicates that the mode of binding and, consequently, the ratio of **1** and [(*R*,*R*)-**2**] changes when the protic 2-propanol or the aprotic toluene–hexane mixture is used as solvent. The enantiopreference of the resolving agent [(*R*,*R*)-**2**] was not solvent dependent,^21a^ and the same secondary phosphine enantiomer [(*R*)-**1** or (*S*)-**1**] was incorporated into the diastereomeric complex under both crystallization conditions. The absolute configuration of the secondary phosphine oxides **1a** and **1g**–**n** was assigned according to literature data.^9b,10b,14b^ Moreover, a single crystal XRD measurement has also confirmed the absolute configuration of (*S*)-**1a** (*vide infra*).

**Scheme 3:**
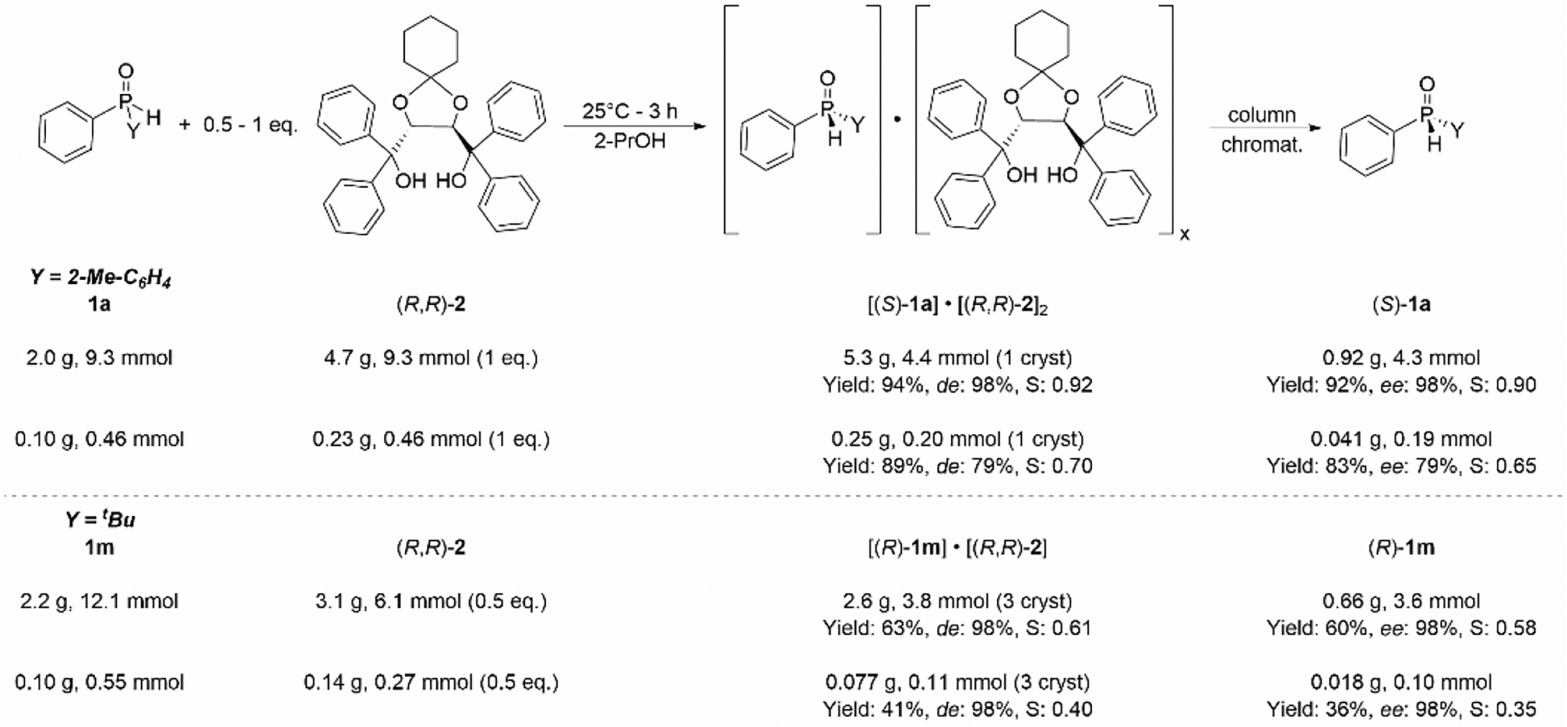
Gram-Scale Resolution of (2-Methylphenyl)-phenylphosphine Oxide (**1a**) and (*t*-Butyl)-phenylphosphine Oxide (**1m**) with (*R*,*R*)-spiro-TADDOL [(*R*,*R*)-**2**]

After the evaluation of the substrate scope of our resolution method, the scalability was assessed. A submillimolar scale (*ca.* 0.10 g) was typically used for optimization, and the resolution of (2-methylphenyl)- and (*t*-butyl)-phenylphosphine oxide (**1a** and **1m**) were also elaborated on a gram scale (*ca.* 2 g of **1a** or **1m**) using (*R*,*R*)-spiro-TADDOL [(*R*,*R*)-**2**] as the resolving agent and 2-PrOH as the solvent (Scheme 3). In both cases, the enantioseparation could be elaborated successfully on a gram scale affording (*S*)-**1a** and (*R*)-**1m** with an ee of 98%. Similarly to the optimization experiments, three crystallizations of the corresponding diastereomer (*R*)-**1m**·(spiro-TADDOL) were necessary to reach a high optical purity of (*R*)-**1m**, and the yield improved from 36% to 60%. Intriguingly, the (*S*)-(2-methylphenyl)-phenylphosphine oxide [(*S*)-**1a**] was obtained with an ee of 98% and in a yield of 92% (*S*: 0.90) even after one crystallization and decomposition of the diastereomeric complex (*S*)-**1a**·(spiro-TADDOL)_2_ by column chromatography. These results are somewhat better than the ones obtained after the first crystallization and decomposition of the corresponding diastereomer [(*S*)-**1a**·(spiro-TADDOL)_2_] during the preliminary studies (ee: 79%; yield: 83%; *S*: 0.65). The larger scale allowed a more efficient crystallization and a better separation of the crystalline diastereomer from the mother liquor, which consequently led to a better yield in both cases and a more simple procedure for (*S*)-**1a**. It is noteworthy that the mother liquor of the optical resolutions contained the other SPO antipode (*R*)-**1a** in excess. Enantiopure (*R*)-**1a** could be obtained by the optical resolution of this mother liquor with (*S*,*S*)-spiro-TADDOL under the same crystallization conditions, as it was demonstrated in our previous studies.^21a,21c^

**Figure 3:**
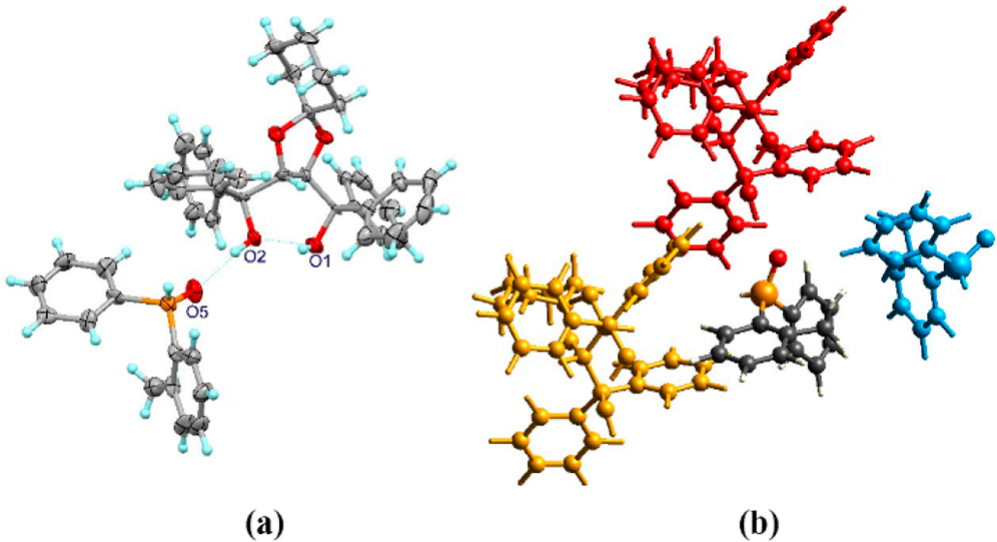
(a) Hydrogen bonds formed in the diastereomeric complex (*S*)-**1a**·(spiro-TADDOL). Nonhydrogen atom ellipsoids are drawn on a 30% probability level. (b) The neighboring molecules with the strongest interactions of (*S*)-**1a** for the calculation of interaction energies (DFT calculation details can be found in Table 1).

The diastereomeric complex (*S*)-**1a**·(spiro-TADDOL) (diastereomeric excess (de): 95%) prepared from a mixture of toluene and hexane was selected for X-ray analysis, and the single crystals were grown from the same solvent mixture. The absolute configuration of (*S*)-(2-methylphenyl)-phenylphosphine oxide [(*S*)-**1a**] was determined using the known absolute configuration of (*R*,*R*)-spiro-TADDOL [(*R*,*R*)-**2**] as reference. The crystal lattice comprises the SPO (*S*)-**1a** and the resolving agent (*R*,*R*)-**2** in a 1:1 ratio, which is in accordance with our NMR measurements. A characteristic intramolecular H bond is present between the two OH groups of (*R*,*R*)-spiro-TADDOL [(*R*,*R*)-**2**],^21a,21c^ and the (*S*)-**1a** and (*R*,*R*)-**2** host–guest molecules are held together by an O–H···O bridge (Figure 3).^25^ Energy calculations were conducted with the Crystal Explorer program using the B3LYP/6-31G(d,p) level of theory (Table 1).^26^ Total energies (*E*_tot_) were the sum of the Coulomb interactions (*E*_ele_), polar interactions (*E*_pol_), dispersion interactions (*E*_dis_), and repulsive interactions (*E*_rep_). The four energy components were scaled in the total energy (*E*_tot_ = 1.019*E*_ele_ + 0.651*E*_pol_ + 0.901*E*_dis_ + 0.811*E*_rep_). Interaction energies were investigated for a 3.8 Å cluster around the selected secondary phosphine oxide (*S*)-**1a**. These DFT calculations also confirmed that the two O–H···O interactions are the strongest ones in the crystal lattice (Table 1 and Figure 3). The second and third strongest interaction come from another neighboring (*R*,*R*)-spiro-TADDOL [(*R*,*R*)-**2**] or (*S*)-(2-methylphenyl)-phenylphosphine oxide [(*S*)-**1a**] molecule, respectively. The O–H···O hydrophilic contact turns the (*R*,*R*)-**2** and (*S*)-**1a** molecules toward each other, creating a tight fit in between the host and guest molecules and creating a hydrophobic outer surface for this associate. Hirshfeld surface analysis also showed a high number of H contacts of the associated molecules. The H atom interactions corresponded to 84% of the intermolecular interactions, while the O atoms had a contribution of only 2.7% (see the Supporting Information for details).^27^

**Table 1:**
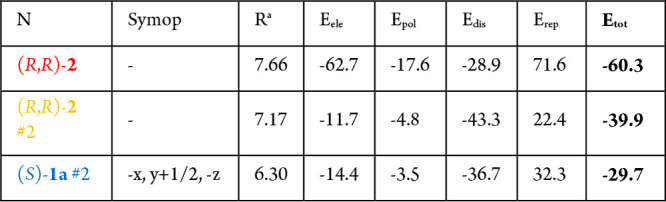
Calculated Total Energies for the Neighboring Molecules of (*S*)-**1a**^b^

a *R* is the distance within the center of the molecules.

b The color of the first column corresponds to the color of the given molecules in Figure 3b.

**Scheme 4:**
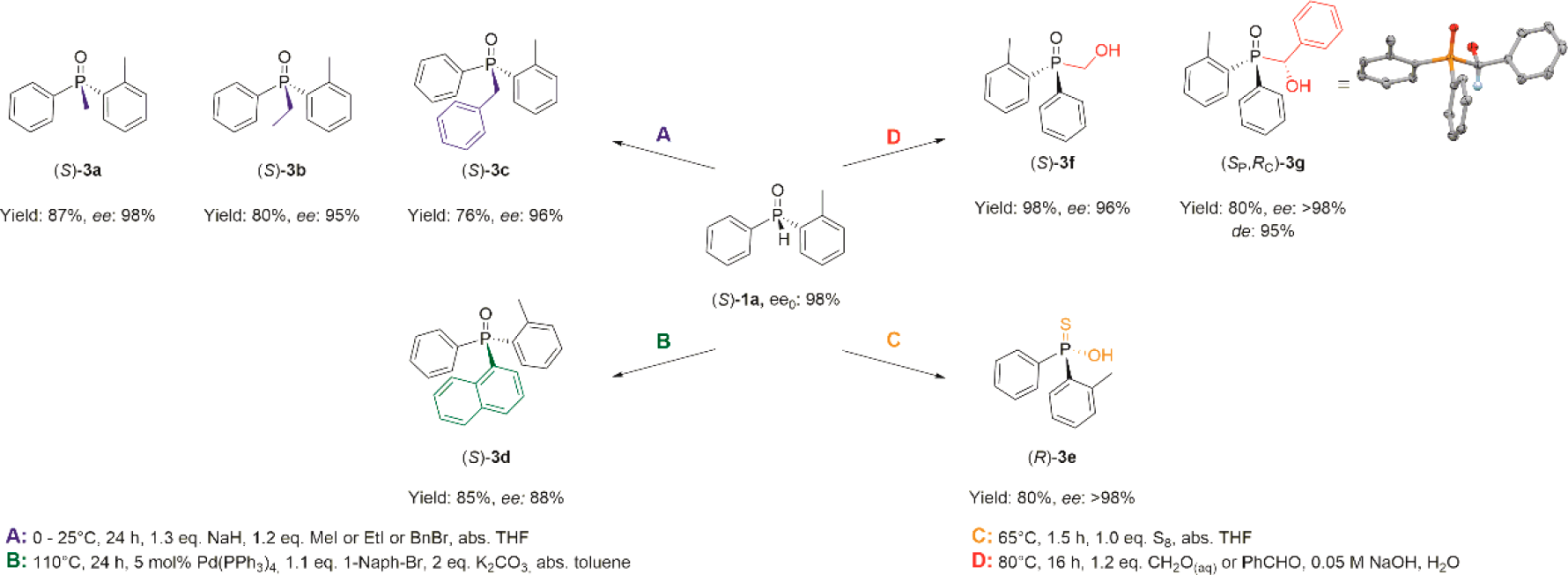
Stereoselective Synthesis of Various *P*-Stereogenic Tertiary Phosphine Oxides and a Thiophosphinate (**3**) from (*S*)-(2-Methylphenyl)phenylphosphine Oxide [(*S*)-**1a**]

As the last step of this study, a few typical SPO transformations were also elaborated in order to prepare the corresponding tertiary phosphine oxides [(*S*)-**3a**–**d**], hydroxyphosphine-oxides [(*S*)-**3f** and (*S*_P_,*R*_C_)-**3g**], and a tiophosphonate [(*R*)-**3e**] (Scheme 4). Previously, such transformations were described using mainly the *t*-butyl-phenylphosphine oxide (**1m**) as the benchmark starting material. As the nature of the substituents may influence the *P*-inversion barrier,^28^ the (*S*)-(2-methylphenyl)phenylphosphine oxide [(*S*)-**1a**] with an ee of 98% was chosen as a diaryl-SPO model compound for these stereoselective transformations. One method for the preparation of *P*-stereogenic tertiary phosphine oxides is the alkylation of metalated secondary phosphine oxides (Michaelis–Becker reaction).^14b,29^ First, (*S*)-**1a** was added to the THF suspension of NaH at 0 °C to prepare (2-MePh)PhP(ONa), and it was treated with the corresponding primary alkyl halide to give (*S*)-methyl-, ethyl- or benzyl-(2-methylphenyl)-phenylphosphine oxide [(*S*)-**3a**–**c**] in yields of 76–87%. The enantiomeric purity of the tertiary phosphine oxides (*S*)-**3a**–**c** was in the range of 95–98%, indicating high configurational stability of the secondary phosphine oxides and their deprotonated derivatives under the reaction conditions (Scheme 4A). Literature data suggested that this reaction proceeds with a retention at the *P*-stereogenic center.^14b,29^

Hirao coupling of (*S*)-(2-methylphenyl)-phenylphosphine oxide [(*S*)-**1a**] with 1-bromonaphtalene was also elaborated. Despite the popularity of this method for the formation of P–C_sp2_ bonds,^30^ only a few reports can be found on the stereospecific arylation of *P*-stereogenic secondary phosphine oxides,^10c,13,31^ and the chiral SPOs might be prone to (partial) racemization under the reaction conditions.^12b^ The P–C cross coupling of (*S*)-**1a** was performed in toluene under reflux conditions in the presence of Pd(PPh_3_)_4_ using K_2_CO_3_ as the base, and these conditions were successfully applied for *t*-BuPhP(O)H (**1m**).^31b^ (*S*)-(1-Naphthyl)-(2-methylphenyl)-phenylphosphine oxide [(*S*)-**3d**] could be prepared with a yield of 85% and in an ee of 88%. The high reaction temperature might be responsible for the partial racemization observed (ee_0_ = 98% → ee = 88%). This cross-coupling is expected to take place with retention of the *P* absolute configuration (Scheme 4B).^31^

The addition of (*S*)-**1a** to formaldehyde or benzaldehyde was also studied in the presence of aq. NaOH. The (*S*)-hydroxymethyl-(2-methylphenyl)-phenylphosphine oxide [(*S*)-**3f**] could be prepared in nearly quantitative yield (98%) and with an ee of 96%. The addition of benzaldehyde to *P*-stereogenic SPOs involves the formation of a new carbon stereogenic center. ^31^P NMR spectra indicated that this reaction was highly diastereoselective, as the ratio of the two diastereomers was 97.5:2.5 in the crude product. Moreover, the (*S*_P_,*R*_C_) enantiomer could be prepared with excellent enantiomeric purity (ee > 98%) and in a yield of 80% (Scheme 4D). We believe that a crystallization induced asymmetric transformation is the underlying reason for this high diastereoselectivity.^20,32^ Crystalline product (**3g**) was immediately formed when (*S*)-**1a** was reacted with benzaldehyde at room temperature. The subsequent stirring of the aqueous suspension at 80 °C for 16 h allows the dissolution of the more soluble diastereomer and its transformation to the less soluble stereoisomer via an equilibrium cascade, which involves a retro-Pudovik reaction in the supernatant. This hypothesis was supported by a few control experiments. When the reaction was performed either at a lower temperature or with shorter reaction times, a significant decrease in the diastereomeric excess of **3g** could be observed. As in the literature,^33^ X-ray crystallography was used for characterization. The XRD measurement of (*S*_P_,*R*_C_)-**3g** confirmed that the reaction proceeds with retention of the *P*-configuration, which is in accordance with the literature.^20,34^ Moreover, the (*R*) absolute configuration of the formed carbon stereogenic center could also be established. The neighboring hydroxy(phenyl)methyl-(2-methylphenyl)-phenylphosphine oxides [(*S*_P_,*R*_C_)-**3g**] were held together by H bonds formed between OH and P=O functional groups of two different (*S*_P_,*R*_C_)-**3g** molecules (see the Supporting Information for details). (*S*)-**1a** was also reacted with elemental sulfur to prepare the corresponding thiophosphonic acid **3e** with retention of the *P*-configuration (Scheme 4C).^9b,16d^ Interestingly, the enantiomeric excess values of **3g** could be determined by ^31^P NMR using (*S*)-1-naphthylethylamine as a chiral solvating agent, as no chiral chromatographic method could be developed for **3g**.

## Conclusions

In conclusion, a series of *P*-stereogenic secondary phosphine oxides (**1**) was prepared in racemic form, and their enantiomeric separation was elaborated with (*R*,*R*)-spiro-TADDOL [(*R*,*R*)-**2**]. The scope of the SPOs comprised several diaryl-derivatives containing a phenyl group and another aryl moiety with various substitution patterns (**1a**–**i**) and alkyl-phenylphospine oxides (**1j**–**n**) incorporating normal, branched, or cycloalkyl groups or a benzyl function. According to the preliminary experiments, the optical resolution of the target secondary phosphine oxides (**1**) was elaborated with (*R*,*R*)-spiro-TADDOL [(*R*,*R*)-**2**] in 2-propanol or in a mixture of toluene and hexane. Nine derivatives (**1a**, **1c**, **1e**, **1f**, **1i**–**k**, **1m**, and **1n**) could be prepared with an ee > 79% (yield: 12–65%), and five of these derivatives (**1a**, **1c**, **1f**, **1k**, and **1m**) were practically enantiopure secondary phosphine oxides (ee > 98%, yield 27–65%). To date, this current method has the widest scope among classical resolution methods developed for *P*-chiral secondary phosphine oxides. Our results indicated that substituents in the para-position of a phenyl group has the least effect on the overall efficiency of the resolution. On the contrary, an increased steric bulk, especially in the ortho-position, had a negative effect on the enantiomeric separation. Several alkyl-phenylphospine oxides (**1j**, **1k**, **1m**, and **1n**) containing various normal, cyclo, or aralkyl chains could be prepared with good enantiomeric purity and in acceptable yields (ee: 87–99%; yield: 12–41%). A gram-scale resolution of (2-methylphenyl)-phenylphosphine oxide (**1a**) was performed, indicating the scalability of this resolution method. X-ray quality crystals were prepared from (*S*)-**1a**·(spiro-TADDOL), which was complemented with the DFT calculation to study the main interaction between the secondary phosphine oxide and the resolving agent.

Starting from (*S*)-(2-methylphenyl)-phenylphosphine oxide [(*S*)-**1a**] (ee: 98%), several *P*-stereogenic phosphine oxides and a thiophosphonate (**3**) were prepared with excellent enantiomeric excess values (ee > 95%). Only the Hirao coupling of secondary phosphine oxide (*S*)-**1a** with 1-bromonaphtalene afforded (*S*)-(1-naphthyl)-(2-methylphenyl)-phenylphosphine oxide [(*S*)-**3d**] with a somewhat lower optical purity (ee: 88%), which could be attributed to the partial racemization of (*S*)-**1a** at the elevated reaction temperature.

## Experimental Section

### General Information

The commercially available reagents were purchased from commercial sources, and they were used without further purification unless otherwise stated. The TADDOL derivatives [(*R*,*R*)-**2**, (*R*,*R*)-**SI-1**, (*R*,*R*)-**SI-3**, and (*R*,*R*,*R*,*R*)-**SI-4**],^35,36^ benzyl-phenylphosphine oxide (**1j**), methyl-phenylphosphine oxide (**1k**), butyl-phenylphosphine oxide (**1l**), *tert*-butyl-phenylphosphine oxide (**1m**), and cyclohexyl-phenylphosphine oxide (**1n**) were synthesized as described in the literature, and their analytical data were identical to the ones reported in the literature.^12b,24^ The solvents were purchased from Merck Chemical Ltd., and they were used without further purification. Solvents used in moisture sensitive reactions were purified and dried according to the standard procedures.^37^ Dry solvents were stored over molecular sieves of 3 or 4 Å. The ^31^P, ^19^F, ^13^C, and ^1^H NMR spectra were taken on a Bruker AV-300 or DRX-500 spectrometer operating at 121.5, 282.4, 75.5, and 300 MHz or 202.5, 470.6, 125.8, and 500 MHz, respectively. Chemical shifts (δ) are given in parts per million (ppm). Chemical shifts (δ) were for ^1^H and ^13^C in CDCl_3_ and referenced to 7.26 and 77.16 ppm, respectively. An 85% solution of H_3_PO_4_ was the external reference for ^31^P NMR chemical shifts. Coupling constants are expressed in Hertz (Hz). The following abbreviations are used: s = singlet, d = doublet, t = triplet, q = quadruplet, m = multiplet, and dd = doublet of doublets. The exact mass measurements were performed using an Agilent 6230C TOF LCMS System with Agilent Jet Stream source in positive ESI mode (buffer: ammonium-formate in water/acetonitrile; drying gas: 325 °C; capillary: 3000 V; Fragmentor 100 V). LCMS measurements were performed using an Agilent 1100 and Agilent 6130 LCMS system in positive and negative electrospray mode. For the single crystal structure determination, the intensity data were collected on a Rigaku RAXIS-RAPID II diffractometer (using a graphite monochromator; Mo Kα radiation, λ = 0.71075 Å). The crystals were measured with fiber. Crystal Clear (developed by Rigaku Company) software was used for data collection and refinement.^38^ Numerical and empirical absorption corrections were applied to the data.^39^ The structures were solved by direct methods. Anisotropic full-matrix least-squares refinements were performed on F2 for all non-hydrogen atoms. Hydrogen atoms bonded to C atoms were placed in calculated positions and refined in a riding-model approximation. The computer programs used for the structure solution, refinement, and analysis of the structures were Shelx,^40^ Sir2014,^41^ Wingx,^42^ Platon,^43^ and Crystal Explorer.^44^ Melting points were obtained on a melting point apparatus and are uncorrected. The preparation of the air and moisture sensitive intermediates (**6** and **7**) or racemic secondary phosphine oxides (**1a**–**n**) was carried out under a nitrogen atmosphere in Schlenk-type reaction vessels using standard Schlenk techniques.^45^ Thin layer chromatography (TLC) was performed on Merck precoated Silica gel 60 F_254_ or neutral Al_2_O_3_ 60 F_254_ aluminum plates with realization by UV irradiation, iodine, and phosphomolybdic acid. Column chromatography was performed on Silica gel 60 or neutral Al_2_O_3_ 90 with a particle size of 0.063–0.200 mm supplied by Merck. Flash column chromatography was performed using a Combi-Flash (Teledyne ISCO) with gradient elution on Silica gel 60 or neutral Al_2_O_3_ 90 columns. The enantiomeric excess (ee) value of **3e** was determined by ^31^P NMR using 5.0 mg (20 μmol) of the analyte, 4.8 μL (30 μmol) of (*S*)-naphthyl-ethyl-amine as CSA, and 750 μL of CDCl_3_ as solvent. Enantiomeric excess (ee) values of secondary and tertiary phosphine oxides **1a**–**n**, **3a**–**d**, and **3f**–**g** were determined by chiral HPLC on a PerkinElmer Series 200 instrument equipped with Phenomenex Lux 5 μm Cellulose-1, Phenomenex Lux 5 μm Cellulose-2, Phenomenex Lux 5 μm Amylose-2 column, or Kromasil 5-Amycoat (250 × 4.6 mm ID, a mixture of hexane/ethanol was used as the eluent with a flow rate of 0.8 mL/min (*T* = 20 °C, UV detector α = 254 nm). Exact chromatographic parameters are detailed in Table S7. Optical rotations were determined on a Perkin–Elmer 341 polarimeter.

### Preparation of Racemic Diaryl Secondary Phosphine Oxides (**1a**–**i**)

#### *N*,*N*-Diethylamino-chloro-phenylphosphine (**6**)

Under nitrogen atmosphere, 21 mL (260 mmol) of anhydrous pyridine was added dropwise over 5 min to a solution of 17 mL (130 mmol) of *P*,*P*-dichlorophenylphosphine (**4**) in 100 mL of anhydrous hexane, and then, the opaque solution was stirred for 10 min at 25 °C. To this reaction mixture, 27 mL (260 mmol) of anhydrous diethyl-amine (**5**) was added dropwise over 20 min. The resulting white suspension was refluxed in an oil bath for 3 h, and it was cooled to 25 °C. The precipitated salt was filtered through a sintered glass funnel under nitrogen atmosphere, and it was washed 2× with 50 mL of anhydrous hexane. The filtrate was concentrated on the rotary evaporator. Distillation under reduced pressure yielded 24 g (84%) of *N*,*N*-diethylamino-chloro-phenylphosphine (**6**) as colorless oil. bp: 95 °C @ 0.5 mbar; (lit. bp: 68–70 °C @ 0.13 mbar).^46^ The distillate was used immediately.

#### (2-Methylphenyl)-phenylphosphine oxide (**1a**) (Representative Procedure I)

Under nitrogen atmosphere, a solution of 4.3 g (20 mmol) *N*,*N*-diethylamino-chloro-phenylphosphine (**6**) in 10 mL of anhydrous THF was added dropwise over 1 h to a solution of 20 mmol of 2-methylphenyllithium at −78 °C [2-methylphenyllithium was prepared by adding 13 mL (20 mmol) of *n*-butyllithium (1.6 M hexane solution) to a solution of 2.4 mL (20 mmol) of 2-bromotoluene and 24 mL of anhydrous THF over 30 min at −78 °C followed by an additional 30 min of stirring at the same temperature]. The reaction mixture was stirred for 2 h at −78 °C. Then, it was allowed to warm to 25 °C, and it was stirred overnight. The solution was concentrated under vacuum, and the residue was redissolved in 20 mL of toluene. Fifteen mL of cc. HCl solution was added over 10 min at 0 °C, and the resulting emulsion was stirred for 15 min. Then, the pH was adjusted to 8 by adding 20% aqueous NaOH solution. The phases were separated, and the aqueous layer was extracted with DCM (3 × 40 mL). The organic layers were combined, dried (Na_2_SO_4_), and evaporated. The crude product was purified by flash column chromatography (Al_2_O_3_, gradient elution, hexane to EtOAc) to give 4.0 g (92%) of (2-methylphenyl)-phenylphosphine oxide (**1a**) as a white solid. mp: 115–117 °C (mp_lit_: 121.5–123 °C);^9b^^31^P{^1^H} NMR (121.5, MHz, CDCl_3_) δ 21.9 (δ_lit_ 21.9);^9b^^1^H NMR (500 MHz, CDCl_3_) δ 8.09 (d, 1H, *J* = 480.1), 7.71–7.60 (m, 3H), 7.53–7.50 (m, 1H), 7.46–7.43 (m, 3H), 7.33–7.30 (m, 1H), 7.23–7.20 (m, 1H), 2.35 (bs, 3H); ^13^C{^1^H} NMR (125.8 MHz, CDCl_3_) δ 141.5 (d, *J* = 9.2), 132.8 (d, *J* = 2.6), 132.3 (d, *J* = 3.0), 132.3 (d, *J* = 13.6), 131.5 (d, *J* = 100.4), 131.4 (d, *J* = 10.4), 130.8 (d, *J* = 11.4), 129.6 (d, *J* = 101.0), 128.9 (d, *J* = 12.7), 126.0 (d, *J* = 13.6), 20.2 (d, *J* = 6.8); HRMS (ESI/TOF) *m*/*z*: [M + H]^+^ Calcd for C_13_H_14_OP 217.0782; Found 217.0786.

#### (3-Methylphenyl)-phenylphosphine oxide (**1b**)

The (3-methylphenyl)-phenylphosphine oxide (**1b**) was prepared according to Representative Procedure I by reacting 4.3 g (20 mmol) of *N*,*N*-diethylamino-chloro-phenylphosphine (**6**) in 10 mL of anhydrous THF with 20 mmol of 3-methylphenyllithium at −78 °C. 3-Methylphenyllithium was prepared from 2.4 mL (20 mmol) of 3-bromotoluene and 13 mL (20 mmol) of *n*-butyllithium (1.6 M hexane solution) in 24 mL of anhydrous THF at −78 °C. The crude product was purified by flash column chromatography (Al_2_O_3_, gradient elution, hexane to EtOAc) to give 3.0 g (70%) of (3-methylphenyl)-phenylphosphine oxide (**1b**) as a clear oil. ^31^P{^1^H} NMR (121.5 MHz, CDCl_3_) δ 21.8; ^1^H NMR (500 MHz, CDCl_3_) δ 8.04 (d, 1H, *J* = 479.6), 7.72–7.67 (m, 2H), 7.58–7.44 (m, 5H), 7.38–7.36 (m, 2H), 2.38 (s, 3H); ^13^C{^1^H} NMR (75.5 MHz, CDCl_3_) δ 139.0 (d, *J* = 12.7), 133.5 (d, *J* = 2.9), 132.6 (d, *J* = 3.0), 131.8 (d, *J* = 101.1), 131.3 (d, *J* = 11.1), 130.8 (d, *J* = 11.4), 130.0 (d, *J* = 100.8), 129.0 (d, *J* = 12.8), 128.9 (d, *J* = 13.7), 127.8 (d, *J* = 11.8), 21.5; HRMS (ESI/TOF) *m*/*z*: [M + H]^+^ Calcd for C_13_H_14_OP 217.0782; Found 217.0786.

#### (4-Methylphenyl)-phenylphosphine oxide (**1c**)

The (4-methylphenyl)-phenylphosphine oxide (**1c**) was prepared according to Representative Procedure I by reacting 4.3 g (20 mmol) of *N*,*N*-diethylamino-chloro-phenylphosphine (**6**) in 10 mL of anhydrous THF with 20 mmol of 4-methylphenyllithium at −78 °C. The 4-methylphenyllithium was prepared from 2.5 mL (20 mmol) of 4-bromotoluene and 13 mL (20 mmol) of *n*-butyllithium (1.6 M hexane solution) in 24 mL of anhydrous THF at −78 °C. The crude product was purified by flash column chromatography (Al_2_O_3_, gradient elution, hexane to EtOAc) to give 2.9 g (68%) of (4-methylphenyl)-phenylphosphine oxide (**1c**) as white solid. mp: 70–72 °C; ^31^P{^1^H} NMR (121.5 MHz, CDCl_3_) δ 21.6 (δ_lit_ 21.9);^47^^1^H NMR (500 MHz, CDCl_3_) δ 8.04 (d, 1H, *J* = 479.5), 7.71–7.66 (m, 2H), 7.61–7.46 (m, 5H), 7.31–7.29 (m, 2H), 2.40 (s, 3H); ^13^C{^1^H} NMR (75.5 MHz, CDCl_3_) δ 143.4 (d, *J* = 2.6), 132.6 (d, *J* = 3.0), 131.9 (d, *J* = 101.7), 130.9 (d, *J* = 11.9), 130.9 (d, *J* = 96.5), 130.8 (d, *J* = 11.4), 129.8 (d, *J* = 13.3), 129.0 (d, *J* = 12.8), 21.8; HRMS (ESI/TOF) *m*/*z*: [M + H]^+^ Calcd for C_13_H_14_OP 217.0782; Found 217.0782.

#### (2-Trifluoromethylphenyl)-phenylphosphine oxide (**1d**)

The (2-trifluoromethylphenyl)-phenylphosphine oxide (**1d**) was prepared according to the Representative Procedure I by reacting 5.2 g (24 mmol) of *N*,*N*-diethylamino-chloro-phenylphosphine (**6**) in 12 mL of anhydrous THF with 24 mmol of 2-trifluoromethylphenyllithium at −78 °C. The 2-trifluoromethylphenyllithium was prepared from 3.3 mL (24 mmol) of 2-bromobenzotrifluoride and 15 mL (24 mmol) of *n*-butyllithium (1.6 M hexane solution) in 28 mL of anhydrous THF at −78 °C. The crude product was purified by flash column chromatography (Al_2_O_3_, gradient elution, hexane to EtOAc) to give 3.8 g (59%) of (2-trifluoromethylphenyl)-phenylphosphine oxide (**1d**) as white solid. mp: 45 °C; ^31^P{^1^H} NMR (121.5 MHz, CDCl_3_) δ 15.4 (q, *J* = 7.5); ^1^H NMR (500 MHz, CDCl_3_) δ 8.33 (dq, 1 H, *J* = 510.4, 3.1), 8.21–8.17 (m, 1H), 7.80–7.78 (m, 1H), 7.76–7.69 (m, 2H), 7.68–7.61 (m, 2H), 7.57–7.53 (m, 1H), 7.49–7.45 (m, 2H); ^13^C{^1^H} NMR (75.5 MHz, CDCl_3_) δ 134.2 (d, *J* = 6.9), 132.8 (d, *J* = 3.0), 132.6 (d, *J* = 2.5), 132.3 (d, *J* = 10.7), 131.5 (d, *J* = 105.0), 131.4 (dd, *J* = 39.1, 6.4), 130.8 (d, *J* = 11.7), 130.6 (d, *J* = 101.4), 129.0 (d, *J* = 13.2), 126.8 (m), 123.8 (dd, *J* = 274.3, 2.6); ^19^F NMR (282.4 MHz, CDCl_3_) δ −56.9 (d, *J* = 7.4); HRMS (ESI/TOF) *m*/*z*: [M + H]^+^ Calcd for C_13_H_11_F_3_OP 271.0500; Found 271.0499.

#### (3-Trifluoromethylphenyl)-phenylphosphine oxide (**1e**)

(3-Trifluoromethylphenyl)-phenylphosphine oxide (**1e**) was prepared according to Representative Procedure I by reacting 5.2 g (24 mmol) of *N*,*N*-diethylamino-chloro-phenylphosphine (**6**) in 12 mL of anhydrous THF with 24 mmol of 3-trifluoromethylphenyllithium at −78 °C. 3-Trifluoromethylphenyllithium was prepared from 3.3 mL (24 mmol) of 3-bromobenzotrifluoride and 15 mL (24 mmol) of *n*-butyllithium (1.6 M hexane solution) in 28 mL of anhydrous THF at −78 °C. The crude product was purified by flash column chromatography (Al_2_O_3_, gradient elution, hexane to EtOAc) to give 3.2 g (50%) of (3-trifluoromethylphenyl)-phenylphosphine oxide (**1e**) as clear oil. ^31^P{^1^H} NMR (121.5 MHz, CDCl_3_) δ 19.8; ^1^H NMR (500 MHz, CDCl_3_) δ 8.12 (d, 1H, *J* = 486.2), 7.99 (d, 1H, *J* = 13.5), 7.89–7.81 (m, 2H), 7.73–7.69 (m, 2H), 7.66–7.59 (m, 2H), 7.55–7.51 (m, 2H); ^13^C{^1^H} NMR (75.5 MHz, CDCl_3_) δ 134.1 (d, *J* = 11.1), 133.2 (d, *J* = 3.0), 133.2 (d, *J* = 99.9), 131.7 (dd, *J* = 32.1, 13.6), 130.8 (d, *J* = 11.7), 130.6 (d, *J* = 102.9), 129.7 (d, *J* = 12.5), 129.4 (d, *J* = 6.2), 129.3 (d, *J* = 13.1), 127.7 (m), 123.6 (dd, *J* = 272.8, 1.7); ^19^F NMR (282.4 MHz, CDCl_3_) δ −62.8; HRMS (ESI/TOF) *m*/*z*: [M + H]^+^ Calcd for C_13_H_11_F_3_OP 271.0500; Found 271.0500.

#### (4-Trifluoromethylphenyl)-phenylphosphine oxide (**1f**)

(4-Trifluoromethylphenyl)-phenylphosphine oxide (**1f**) was prepared according to Representative Procedure I by reacting 5.2 g (24 mmol) of *N*,*N*-diethylamino-chloro-phenylphosphine (**6**) in 12 mL of anhydrous THF with 24 mmol of 4-trifluoromethylphenyllithium at −78 °C. 4-Trifluoromethylphenyllithium was prepared from 3.3 mL (24 mmol) of 4-bromobenzotrifluoride and 15 mL (24 mmol) of *n*-butyllithium (1.6 M hexane solution) in 28 mL of anhydrous THF at −78 °C. The crude product was purified by flash column chromatography (Al_2_O_3_, gradient elution, hexane to EtOAc) to give 3.3 g (51%) of (4-trifluoromethylphenyl)-phenylphosphine oxide (**1f**) as clear oil. ^31^P{^1^H} NMR (121.5 MHz, CDCl_3_) δ 19.8 (δ_lit_ 19.6);^48^^1^H NMR (500 MHz, CDCl_3_) δ 8.11 (d, 1H, *J* = 486.5), 7.87–7.81 (m, 2H), 7.76–7.68 (m, 4H), 7.63–7.58 (m, 1H), 7.55–7.50 (m, 2H); ^13^C{^1^H} NMR (75.5 MHz, CDCl_3_) δ 135.8 (d, *J* = 97.9), 134.5 (dd, *J* = 32.8, 3.1), 133.2 (d, *J* = 2.9), 131.4 (d, *J* = 11.7), 130.8 (d, *J* = 11.7), 129.9 (d, *J* = not visible), 129.3 (d, *J* = 13.1), 125.9 (dq, *J* = 12.8, 3.6), 123.6 (dd, *J* = 272.9, 1.0); ^19^F NMR (282.4 MHz, CDCl_3_) δ −63.2; HRMS (ESI/TOF) *m*/*z*: [M + H]^+^ Calcd for C_13_H_11_F_3_OP 271.0500; Found 271.0502.

#### (2-Methoxyphenyl)-phenylphosphine oxide (**1g**)

(2-Methoxyphenyl)-phenylphosphine oxide (**1g**) was prepared according to Representative Procedure I by reacting 4.3 g (20 mmol) of *N*,*N*-diethylamino-chloro-phenylphosphine (**6**) in 10 mL of anhydrous THF with 20 mmol of 2-methoxyphenyllithium at −78 °C. 2-Methoxyphenyllithium was prepared from 2.5 mL (20 mmol) of 2-bromoanisole and 13 mL (20 mmol) of *n*-butyllithium (1.6 M hexane solution) in 24 mL of anhydrous THF at −78 °C. The crude product was purified by flash column chromatography (Al_2_O_3_, gradient elution, hexane to EtOAc) to give 3.0 g (65%) of (2-methoxyphenyl)-phenylphosphine oxide (**1g**) as pale yellow solid. mp: 94–96 °C (mp_lit_: 101–103 °C);^49^^31^P{^1^H} NMR (121.5 MHz, CDCl_3_) δ 12.5 (δ_lit_ 14.4);^49^^1^H NMR (500 MHz, CDCl_3_) δ 8.16 (d, 1H, *J* = 499.0), 7.81–7.70 (m, 3H), 7.55–7.43 (m, 4H), 7.11–7.08 (m, 1H), 6.92–6.89 (m, 1H), 3.77 (s, 3H); ^13^C{^1^H} NMR (75.5 MHz, CDCl_3_) δ 160.8 (d, *J* = 3.8), 134.5 (d, *J* = 1.9), 133.2 (d, *J* = 7.1), 132.3 (d, *J* = 104.5), 132.1 (d, *J* = 3.0), 130.6 (d, *J* = 11.8), 128.6 (d, *J* = 13.1), 121.2 (d, *J* = 12.1), 119.6 (d, *J* = 101.9), 110.9 (d, *J* = 6.1), 55.6; HRMS (ESI/TOF) *m*/*z*: [M + H]^+^ Calcd for C_13_H_14_O_2_P 233.0731; Found 233.0733.

#### (2-Phenylphenyl)-phenylphosphine oxide (**1h**)

(2-Phenylphenyl)-phenylphosphine oxide (**1h**) was prepared according to Representative Procedure I by reacting 4.3 g (20 mmol) of *N*,*N*-diethylamino-chloro-phenylphosphine (**6**) in 10 mL of anhydrous THF with 20 mmol of 2-phenylphenyllithium at −78 °C. 2-Phenylphenyllithium was prepared from 3.4 mL (20 mmol) of 2-bromobiphenyl and 13 mL (20 mmol) of *n*-butyllithium (1.6 M hexane solution) in 24 mL of anhydrous THF at −78 °C. The crude product was purified by flash column chromatography (Al_2_O_3_, gradient elution, hexane to EtOAc) to give 4.5 g (81%) of (2-phenylphenyl)-phenylphosphine oxide (**1h**) as clear oil. ^31^P{^1^H} NMR (202.5 MHz, CDCl_3_) δ 18.4 (δ_lit_ 18.4);^10b^^1^H NMR (500 MHz, CDCl_3_) δ 7.95 (dd, 1H, *J* = 14.1, 7.6), 7.88 (d, 1H, *J* = 493.3), 7.60 (t, 1H, *J* = 7.5), 7.51 (t, 1H, *J* = 7.6), 7.43–7.21 (m, 11H); ^13^C{^1^H} NMR (125.8 MHz, CDCl_3_) δ 146.1 (d, *J* = 10.1), 139.3 (d, *J* = 5.2), 132.9 (d, *J* = 10.4), 132.4 (d, *J* = 2.7), 132.0 (d, *J* = 2.9), 131.7 (d, *J* = 102.5), 130.8 (d, *J* = 9.3), 130.6 (d, *J* = 11.5), 130.4 (d, *J* = 105.7), 129.5, 128.5 (d, *J* = 13.1), 128.3, 128.1, 127.6 (d, *J* = 12.0); HRMS (ESI/TOF) *m*/*z*: [M + H]^+^ Calcd for C_18_H_16_OP 279.0939; Found 279.0934.

#### (1-Naphthyl)-phenylphosphine oxide (**1i**)

(1-Naphthyl)-phenylphosphine oxide (**1i**) was prepared according to Representative Procedure I by reacting 5.2 g (24 mmol) of *N*,*N*-diethylamino-chloro-phenylphosphine (**6**) in 12 mL of anhydrous THF with 24 mmol of 1-naphthyllithium at −78 °C. 1-Naphthyllithium was prepared from 3.4 mL (24 mmol) of 1-bromonaphthalene and 15 mL (24 mmol) of *n*-butyllithium (1.6 M hexane solution) in 28 mL of anhydrous THF at −78 °C. The crude product was purified by flash column chromatography (Al_2_O_3_, gradient elution, hexane to EtOAc) to give 4.5 g (74%) of (1-naphthyl)-phenylphosphine oxide (**1i**) as clear oil. ^31^P{^1^H} NMR (121.5 MHz, CDCl_3_) δ 23.2 (δ_lit_ 23.2);^9b^^1^H NMR (500 MHz, CDCl_3_) δ 8.43 (d, 1H, *J* = 484.1), 8.28 (d, 1H, *J* = 8.13), 8.08 (d, 1H, *J* = 8.31), 8.00–7.90 (m, 2H), 7.75–7.70 (m, 2H), 7.60–7.43 (m, 6H); ^13^C{^1^H} NMR (75.5 MHz, CDCl_3_) δ 133.9 (d, *J* = 3.0), 133.8 (d, *J* = 9.0), 132.8 (d, *J* = 8.7), 132.5 (d, *J* = 3.0), 132.3 (d, *J* = 13.4), 131.7 (d, *J* = 102.8), 130.9 (d, *J* = 11.4), 129.2 (d, *J* = 1.6), 129.0 (d, *J* = 12.8), 127.9, 127.6 (d, *J* = 100.3), 127.0, 125.5 (d, *J* = 7.5), 124.9 (d, *J* = 15.4); HRMS (ESI/TOF) *m*/*z*: [M + H]^+^ Calcd for C_16_H_14_OP 253.0782; Found 253.0781.

### Preparation of ((2*R*,3*R*)-1,4-Dioxaspiro[4.5]decane-2,3-diyl)bis(bis(4-(*tert*-butyl)phenyl)methanol) [(*R*,*R*)-**SI-2**]

(*R*,*R*)-**SI-2** was synthesized according to a modified procedure of Beck et al.^35^ To a solution of 44 mmol of (4-(*tert*-butyl)phenyl)magnesium bromide in 35 mL of anhydrous THF was added 2.5 g (8.7 mmol) of diethyl (2*R*,3*R*)-1,4-dioxaspiro[4.5]decane-2,3-dicarboxylate in 15 mL of anhydrous THF over 30 min at 0 °C under nitrogen atmosphere. [(4-(*tert*-butyl)phenyl)magnesium bromide was prepared from 7.6 mL (44 mmol) of 1-bromo-4-(*tert*-butyl)benzene and 1.2 g (48 mmol) of Mg in 35 mL of anhydrous THF]. The resulting solution was heated at reflux in an oil bath for 4 h; then, it was allowed to cool to room temperature, and it was stirred overnight. 40 mL of saturated NH_4_Cl and 20 mL of water were added. The phases were separated, and the aqueous layer was extracted with DCM (3 × 40 mL). The organic layers were combined, dried (Na_2_SO_4_), and evaporated. The crude product was purified by flash column chromatography (silica gel, gradient elution, hexane to CHCl_3_) to give 4.3 g (67%) of ((2*R*,3*R*)-1,4-dioxaspiro[4.5]decane-2,3-diyl)bis(bis(4-(*tert*-butyl)phenyl)methanol) [(*R*,*R*)-**SI-2**] as a white solid. mp: 160–163 °C; [α]_D_^25^ = −60.9 (*c* = 1.00, CHCl_3_); ^1^H NMR (500 MHz, CDCl_3_) δ 7.47–7.29 (m, 16H), 4.59 (s, 2H), 3.97 (s, 2H), 1.46–1.03 (m, 46H); ^13^C{^1^H} NMR (125.8 MHz, CDCl_3_) δ 150.0, 149.7, 143.6, 139.9, 128.1, 127.3, 124.9, 124.0, 109.9, 80.9, 77.9, 36.5, 34.4, 31.4, 31.3, 25.2, 24.1; HRMS (ESI/TOF) *m*/*z*: [M – H]^−^ Calcd for C_50_H_65_O_4_P 729.4888; Found 729.4885.

### Representative Resolution Procedures

Over the course of this research project, it was observed that even the residual water content of the solvent may influence the outcome of the resolution in a negative manner. Thus, all the solvents used for resolution were dried according to the standard procedures. Dry solvents were stored over molecular sieves of 3 or 4 Å.

#### Resolution of (2-Methylphenyl)-phenylphosphine oxide (**1a**) with spiro-TADDOL [(*R*,*R*)-**2**] Using the Crystallization Method (Representative Procedure II)

0.10 g (0.46 mmol) of racemic (2-methylphenyl)-phenylphosphine oxide (**1a**) and 0.23 g (0.46 mmol) of spiro-TADDOL [(*R*,*R*)-**2**] were dissolved in 1.4 mL of hot 2-PrOH using a hot plate as the heat source. The colorless crystalline diastereomeric complex of (*S*)-**1a**·(spiro-TADDOL)_2_ appeared after cooling the mixture to 25 °C. After standing at 25 °C for 3 h, the crystals were separated by filtration and washed with 0.46 mL of 2-PrOH to give 0.25 g (89%) of (*S*)-**1a**·(spiro-TADDOL)_2_ with a de of 79%. The diastereomeric complex (*S*)-**1a**·(spiro-TADDOL)_2_ was purified by two recrystallizations from 1.4 mL of 2-PrOH according to the procedure described above to afford 0.18 g (65%) of the (*S*)-**1a**·(spiro-TADDOL)_2_ with a de of 98% (Scheme 2; Table S2, Entry 1). (*S*)-(2-Methylphenyl)-phenylphosphine oxide [(*S*)-**1a**] was recovered from the diastereomer by flash column chromatography (silica gel, gradient elution, DCM to DCM–MeOH 95:5) to give 0.031 g (61%) of (*S*)-(2-methylphenyl)-phenylphosphine oxide [(*S*)-**1a**] with an ee of 98%.

The resolution of secondary phosphine oxides (**1**) with TADDOL derivatives was performed according to Representative Procedure II when *i*-Pr_2_O, acetone, MeOH, EtOH, or 2-PrOH was used as solvent. All conditions and results can be found in Tables S1, S2, and S4. Scheme 2 shows the selected results.

#### Resolution of (2-Methylphenyl)-phenylphosphine oxide (**1a**) with spiro-TADDOL [(*R*,*R*)-**2**] Using the Precipitation Method (Representative Procedure III)

0.10 g (0.46 mmol) of racemic (2-methylphenyl)-phenylphosphine oxide (**1a**) and 0.12 g (0.23 mmol) of spiro-TADDOL [(*R*,*R*)-**2**] were dissolved in 0.58 mL of hot toluene using a hot plate as the heat source, and then, 0.58 mL of hexane was added. The colorless crystalline diastereomeric complex of (*S*)-**1a**·(spiro-TADDOL) appeared immediately. After standing at 25 °C for 3 h, the crystals were separated by filtration and washed with 0.20 mL of hexane to give 0.13 g (75%) of (*S*)-**1a**·(spiro-TADDOL) with a de of 77%. The diastereomeric complex (S)-**1a**·(spiro-TADDOL) was purified by two recrystallizations from a mixture of 0.58 mL of toluene and 0.58 mL of hexane according to the procedure described above to afford 0.093 g (55%) of the (*S*)-**1a**·(spiro-TADDOL) with a de of 95% (Scheme 2; Table S3, Entry 1). (*S*)-(2-Methylphenyl)-phenylphosphine oxide [(*S*)-**1a**] was recovered from the diastereomer by flash column chromatography (silica gel, gradient elution, DCM to DCM–MeOH 95:5) to give 0.025 g (50%) of (*S*)-(2-methylphenyl)-phenylphosphine oxide [(*S*)-**1a**] with an ee of 95%.

The resolution of secondary phosphine oxides (**1**) with TADDOL derivatives was performed according to Representative Procedure III when a toluene/hexane or EtOAc/hexane mixture was used as solvent. All conditions and results can be found in Tables S1, S3, and S4. Scheme 2 shows the selected results.

##### (*S*)-(2-Methylphenyl)-phenylphosphine Oxide [(*S*)-**1a**]

[α]_D_^25^ = −44.2 (*c* = 1.07, CHCl_3_, ee = 98%), Chiral HPLC: Phenomenex Lux 5 μm Amylose-2 column, hexane/ethanol (85:15), *t*_R1_ 20.8 min (*R*), *t*_R2_ 22.5 min (*S*).

##### (−)-(3-Methylphenyl)-phenylphosphine Oxide [(−)-**1b**]

[α]_D_^25^ = −1.3 (*c* = 1.35, CHCl_3_, ee = 62%), Chiral HPLC: Phenomenex Lux 5 μm Cellulose-2 column, hexane/ethanol (50:50), *t*_R1_ 11.1 min (−), *t*_R2_ 12.8 min (+).

##### (−)-(4-Methylphenyl)-phenylphosphine Oxide [(−)-**1c**]

[α]_D_^25^ = −7.2 (*c* = 0.72, CHCl_3_, ee = 99%), Chiral HPLC: Phenomenex Lux 5 μm Amylose-2 column, hexane/ethanol (50:50), *t*_R1_ 10.3 min (−), *t*_R2_ 11.2 min (+).

##### (2-Trifuoromethylphenyl)-phenylphosphine Oxide (**1d**)

[α]_D_^25^ = not measured, ee = 0%, Chiral HPLC: Phenomenex Lux 5 μm Cellulose-2 column, hexane/ethanol (50:50), *t*_R1_ 7.3 min, *t*_R2_ 7.9 min.

##### (+)-(3-Trifuoromethylphenyl)-phenylphosphine Oxide [(+)-**1e**]

[α]_D_^25^ = +10.0 (*c* = 0.78, CHCl_3_, ee = 79%); Chiral HPLC: Phenomenex Lux 5 μm Amylose-2 column, hexane/ethanol (50:50), *t*_R1_ 6.7 min (+), *t*_R2_ 8.7 min (−).

##### (+)-(4-Trifuoromethylphenyl)-phenylphosphine Oxide [(+)-**1f**]

[α]_D_^25^ = +13.0 (*c* = 0.68, CHCl_3_, ee = 99%); Chiral HPLC: Phenomenex Lux 5 μm Amylose-2 column, hexane/ethanol (50:50), *t*_R1_ 6.4 min (+), *t*_R2_ 8.3 min (−).

##### (*R*)-(2-Methoxylphenyl)-phenylphosphine Oxide [(*R*)-**1g**]

[α]_D_^25^ = +40.9 (*c* = 0.91, CHCl_3_, ee = 67%), Chiral HPLC: Phenomenex Lux 5 μm Cellulose-1 column, hexane/ethanol (85:15), *t*_R1_ 10.8 min (*S*), *t*_R2_ 15.4 min (*R*).

##### (*S*)-(2-Phenylphenyl)-phenylphosphine Oxide [(*S*)-**1h**]

[α]_D_^25^ = −30.4 (*c* = 0.58, CHCl_3_, ee = 40%); Chiral HPLC: Phenomenex Lux 5 μm Cellulose-2 column, hexane/ethanol (50:50), *t*_R1_ 10.0 min (*R*), *t*_R2_ 12.0 min (*S*).

##### (*R*)-(1-Naphthyl)-phenylphosphine Oxide [(*R*)-**1i**]

[α]_D_^25^ = −9.7 (*c* = 1.79, CHCl_3_, ee = 83%); Chiral HPLC: Phenomenex Lux 5 μm Amylose-2 column, hexane/ethanol (50:50), *t*_R1_ 8.9 min (*R*), *t*_R2_ 9.8 min (*S*).

##### (*S*)-Benzyl-phenylphosphine Oxide [(*S*)-**1j**]

[α]_D_^25^ = +47.8 (*c* = 0.49, CHCl_3_, ee = 87%); Chiral HPLC: Phenomenex Lux 5 μm Amylose-2 column, hexane/ethanol (50:50), *t*_R1_ 9.7 min (*S*), *t*_R2_ 16.1 min (*R*); ^31^P{^1^H} NMR (202.5 MHz, CDCl_3_) δ 29.5 (δ_lit_ 29.6);^9b^^1^H NMR (500 MHz, CDCl_3_) δ 7.56–7.41 (m, 5H), 7. 47 (dt, *J* = 474.7, 3.6, 1H), 7.27–7.22 (m, 3H), 7.06–7.04 (m, 2H), 3.48 (ddd, *J* = 17.3, 14.5, 3.0, 1H), 3.35 (td, *J* = 14.9, 4.2, 1H); ^13^C{^1^H} NMR (125.8 MHz, CDCl_3_) δ 132.7 (d, *J* = 3.0), 130.6 (d, *J* = 7.4), 130.2 (d, *J* = 10.8), 130.1 (d, *J* = 97.0), 129.9 (d, *J* = 5.7), 129.0 (d, *J* = 3.1), 128.8 (d, *J* = 12.4), 127.4 (d, *J* = 3.6), 38.9 (d, *J* = 62.6); HRMS (ESI/TOF) *m*/*z*: [M + H]^+^ Calcd for C_13_H_14_OP 217.0782; Found 217.0778.

##### (*R*)-Methyl-phenylphosphine Oxide [(*R*)-**1k**]

[α]_D_^25^ = +11.4 (*c* = 0.70, CHCl_3_, ee = 99%); Chiral HPLC: Phenomenex Lux 5 μm Cellulose-2 column, hexane/ethanol (50:50), *t*_R1_ 9.1 min (*S*), *t*_R2_ 10.8 min (*R*); ^31^P{^1^H} NMR (202.5 MHz, CDCl_3_) δ 20.4 (δ_lit_ 20.3);^9b^^1^H NMR (500 MHz, CDCl_3_) δ 7.69–7.64 (m, 2H), 7.59 (dq, *J* = 472.4, 3.8, 1H), 7.54–7.44 (m, 3H), 1.75 (dd, *J* = 14.0, 3.8 Hz, 3H); ^13^C{^1^H} NMR (125.8 MHz, CDCl_3_) δ 132.5 (d, *J* = 2.9), 132.0 (d, *J* = 99.9), 129.6 (d, *J* = 11.3), 129.0 (d, *J* = 12.7), 16.3 (d, *J* = 69.0); HRMS (ESI/TOF) *m*/*z*: [M + H]^+^ Calcd for C_7_H_10_OP 141.0469; Found 141.0464.

##### (*S*)-Butyl-phenylphosphine Oxide [(*S*)-**1l**]

[α]_D_^25^ = −11.2 (*c* = 0.51, CHCl_3_, ee = 45%); Chiral HPLC: Phenomenex Lux 5 μm Amylose-2 column, hexane/ethanol (50:50), *t*_R1_ 7.1 min (*S*), *t*_R2_ 7.8 min (*R*); ^31^P{^1^H} NMR (202.5 MHz, CDCl_3_) δ 28.0 (δ_lit_ 28.0);^9b^^1^H NMR (500 MHz, CDCl_3_) δ 7.71–7.67 (m, 2H), 7.58–7.48 (m, 3H), 7.47 (dtd, *J* = 463.1, 3.4, 1.6, 1H), 2.04–1.95 (m, 2H), 1.64–1.52 (m, 2H), 1.48–1.37 (m, 2H), 0.90 (td, *J* = 7.3, 1.6, 3H); ^13^C{^1^H} NMR (125.8 MHz, CDCl_3_) δ 132.5 (d, *J* = 2.9), 131.3 (d, *J* = 96.4), 130.0 (d, *J* = 10.9), 129.0 (d, *J* = 12.3), 30.2 (d, *J* = 68.1), 23.8 (d, *J* = 14.7), 23.7 (d, *J* = 3.7), 13.7; HRMS (ESI/TOF) *m*/*z*: [M + H]^+^ Calcd for C_10_H_16_OP 183.0939; Found 183.0934.

##### (*R*)-*tert*-Butyl-phenylphosphine Oxide [(*R*)-**1m**]

[α]_D_^25^ = +34.4 (*c* = 1.32, CHCl_3_, ee = 98%); Chiral HPLC: Kromasil 5-Amycoat column, hexane/ethanol (85:15), *t*_R1_ 8.8 min (*S*), *t*_R2_ 11.9 min (*R*); ^31^P{^1^H} NMR (202.5 MHz, CDCl_3_) δ 47.5 (δ_lit_ 47.6);^9b^^1^H NMR (500 MHz, CDCl_3_) δ 7.71–7.67 (m, 2H), 7.60–7.57 (m, 1H), 7.52–7.50 (m, 2H), 7.04 (d, *J* = 452.8, 1H), 1.16 (d, *J* = 16.6, 9H); ^13^C{^1^H} NMR (125.8 MHz, CDCl_3_) δ 132.6 (d, *J* = 2.7), 131.0 (d, *J* = 10.0), 129.1 (d, *J* = 90.1), 128.6 (d, *J* = 11.8), 32.1 (d, *J* = 69.2), 23.6 (d, *J* = 2.1); HRMS (ESI/TOF) *m*/*z*: [M + H]^+^ Calcd for C_10_H_16_OP 183.0939; Found 183.0936.

##### (*S*)-Cyclohexyl-phenylphosphine Oxide [(*S*)-**1n**]

[α]_D_^25^ = −25.3 (*c* = 1.06, CHCl_3_, ee = 92%); Chiral HPLC: Phenomenex Lux 5 μm Cellulose-2 column, hexane/ethanol (50:50), *t*_R1_ 10.0 min (*S*), *t*_R2_ 19.7 min (*R*); ^31^P{^1^H} NMR (202.5 MHz, CDCl_3_) δ 36.6 (δ_lit_ 36.7);^10b^^1^H NMR (500 MHz, CDCl_3_) δ 7.68–7.64 (m, 2H), 7.58–7.47 (m, 3H), 7.18 (d, *J* = 446.6, 1H), 1.93–1.79 (m, 5H), 1.70–1.68 (m, 1H), 1.39–1.17 (m, 5H); ^13^C{^1^H} NMR (125.8 MHz, CDCl_3_) δ 132.5 (d, *J* = 2.8), 130.4 (d, *J* = 10.4), 130.0 (d, *J* = 92.9), 128.9 (d, *J* = 12.0), 38.7 (d, *J* = 69.7), 26.1 (d, *J* = 5.3), 26.0 (d, *J* = 4.9), 25.9 (d, *J* = 1.7), 25.4 (d, *J* = 1.9), 24.7 (d, *J* = 2.6); HRMS (ESI/TOF) *m*/*z*: [M + H]^+^ Calcd for C_12_H_18_OP 209.1095; Found 209.1093.

### Gram-Scale Resolution of (2-Methylphenyl)-phenylphosphine Oxide (**1a**) or *tert*-Butyl-phenylphosphine Oxide (**1m**) with spiro-TADDOL [(*R*,*R*)-**2**]

#### (2-Methylphenyl)-phenylphosphine Oxide (**1a**)

2.0 g (9.3 mmol) of racemic (2-methylphenyl)-phenylphosphine oxide (**1a**) and 4.7 g (9.3 mmol) of spiro-TADDOL [(*R*,*R*)-**2**] were dissolved in 28 mL of hot 2-PrOH using an oil bath as the heat source. The colorless crystalline diastereomeric complex of (*S*)-**1a**·(spiro-TADDOL)_2_ appeared after cooling the mixture to 25 °C. After standing at 25 °C for 3 h, the crystals were separated by filtration and washed 2× with 2.8 mL of 2-PrOH to give 5.3 g (94%) of (*S*)-**1a**·(spiro-TADDOL)_2_ with a de of 98%. (*S*)-(2-Methylphenyl)-phenylphosphine oxide [(*S*)-**1a**] was recovered from the diastereomer by flash column chromatography (silica gel, gradient elution, DCM to DCM–MeOH 95:5) to give 0.92 g (92%) of (*S*)-(2-methylphenyl)-phenylphosphine oxide [(*S*)-**1a**] with an ee of 98% (Scheme 3).

#### *tert*-Butyl-phenylphosphine Oxide (**1m**)

2.2 g (12.1 mmol) of racemic *tert*-butyl-phenylphosphine oxide (**1m**) and 3.1 g (6.1 mmol) of spiro-TADDOL [(*R*,*R*)-**2**] were dissolved in 18 mL of hot 2-PrOH using an oil bath as the heat source. The colorless crystalline diastereomeric complex of (*R*)-**1m**·(spiro-TADDOL) appeared after cooling the mixture to 25 °C. After standing at 25 °C for 3 h, the crystals were separated by filtration and washed 2× with 1.8 mL of 2-PrOH to give 3.6 g (87%) of (*R*)-**1m**·(spiro-TADDOL) with a de of 78%. The diastereomeric complex (*R*)-**1m**·(spiro-TADDOL) was purified by two recrystallizations from 18 mL of 2-PrOH according to the procedure described above to afford 2.6 g (63%) of the (*R*)-**1m**·(spiro-TADDOL) with a de of 98%. (*R*)-*tert*-Butyl-phenylphosphine oxide [(*R*)-**1m**] was recovered from the diastereomer by flash column chromatography (silica gel, gradient elution, DCM to DCM–MeOH 95:5) to give 0.66 g (60%) of (*R*)-*tert*-butyl-phenylphosphine oxide [(*R*)-**1m**] with an ee of 98% (Scheme 3).

### Preparation of (*S*)-Methyl-(2-methylphenyl)-phenylphosphine Oxide [(*S*)-**3a**] via Stereospecific Alkylation (Representative Procedure IV)

Under argon atmosphere, 20 mg of 60% (w/w%) NaH dispersion (12 mg of NaH, 0.48 mmol) was washed with anhydrous THF (3 × 1.0 mL); then, 1.0 mL of anhydrous THF was added. To this suspension, 80 mg (0.37 mmol) of (*S*)-(2-methylphenyl)-phenylphosphine oxide [(*S*)-**1a**, ee = 98%] in 1.0 mL of anhydrous THF was added dropwise over 10 min at 0 °C, and the reaction mixture was stirred for 45 min at this temperature. Then, 28 μL (0.44 mmol) of MeI in 1.0 mL of anhydrous THF was added dropwise over 30 min at 0 °C. The reaction mixture was allowed to warm to 25 °C, and it was stirred overnight. Then, 1 mL of saturated NH_4_Cl solution and 1 mL of water were added. Phases were separated, and the aqueous layer was extracted with EtOAc (3 × 1 mL). The organic layers were combined, dried (Na_2_SO_4_), and evaporated. The crude product was purified by flash column chromatography (silica gel, gradient elution, CHCl_3_ to CHCl_3_–MeOH 97:3) to give 74 mg (87%) of (*S*)-methyl-(2-methylphenyl)-phenylphosphine oxide [(*S*)-**3a**] with an ee of 98% as a white solid. mp: 104 °C (mp_lit_: 114 °C);^50^ [α]_D_^25^ = −33.0 (*c* = 1.00, CHCl_3_, ee = 98%, *S*_P_) ([α]_lit_ = −28.2 (*c* = 1.0, CHCl_3_, ee = 96%, *S*_P_));^50^ Chiral HPLC: Kromasil 5-Amycoat column, hexane/ethanol (85:15), *t*_R1_ 10.9 min (*S*), *t*_R2_ 13.5 min (*R*); ^31^P{^1^H} NMR (121.5 MHz, CDCl_3_) δ 31.8 (δ_lit_ 31.4);^50^^1^H NMR (500 MHz, CDCl_3_) δ 7.68–7.61 (m, 3H), 7.52–7.40 (m, 4H), 7.30–7.21 (m, 2H), 2.36 (s, 3H), 2.03 (d, 3H, *J* = 13.1); ^13^C{^1^H} NMR (75.5 MHz, CDCl_3_) δ 142.2 (d, *J* = 8.3), 134.8 (d, *J* = 100.1), 132.2 (d, *J* = 2.8), 132.0 (d, *J* = 10.3), 131.6, 131.6 (d, *J* = 14.6), 130.9 (*J* = not visible), 130.5 (d, *J* = 10.0), 128.7 (d, *J* = 12.0), 125.6 (d, *J* = 12.3), 21.4 (d, *J* = 4.7), 17.3 (d, *J* = 74.1); HRMS (ESI/TOF) *m*/*z*: [M + H]^+^ Calcd for C_14_H_16_OP 231.0939; Found 231.0937.

#### (*S*)-Ethyl-(2-methylphenyl)-phenylphosphine Oxide [(*S*)-**3b**]

(*S*)-Ethyl-(2-methylphenyl)-phenylphosphine oxide [(*S*)-**3b**] was prepared according to Representative Procedure IV by reacting 12 mg (0.48 mmol) of NaH in 1.0 mL of anhydrous THF with 80 mg (0.37 mmol) of (*S*)-(2-methylphenyl)-phenylphosphine oxide [(*S*)-**1a**, ee = 98%] in 1.0 mL of anhydrous THF and 36 μL (0.44 mmol) of EtI in 1.0 mL of anhydrous THF at 0 °C. The crude product was purified by flash column chromatography (silica gel, gradient elution, CHCl_3_ to CHCl_3_–MeOH 97:3) to give 72 mg (80%) of (*S*)-ethyl-(2-methylphenyl)-phenylphosphine oxide [(*S*)-**3b**] with an ee of 95% as a white solid. mp 90–92 °C (mp_lit_: 94–95 °C);^21b^ [α]_D_^25^ = −31.0 (*c* = 1.00, CHCl_3_, ee = 95%, *S*_P_) ([α]_lit_ = +32.6 (*c* = 1.2, CHCl_3_, ee = 99%, *R*_P_));^21b^ Chiral HPLC: Kromasil 5-Amycoat column, hexane/ethanol (85:15), *t*_R1_ 8.4 min (*S*), *t*_R2_ 12.6 min (*R*). ^31^P{^1^H} NMR (121.5 MHz, CDCl_3_) δ 36.2 (δ_lit_ 35.5);^51^^1^H NMR (500 MHz, CDCl_3_) δ 7.67–7.60 (m, 3H), 7.50–7.39 (m, 4H), 7.30–7.26 (m, 1H), 7.22–7.20 (m, 1H), 2.43–2.19 (m, 5H), 1.19 (dt, 3H *J* = 16.9, 7.6); ^13^C{^1^H} NMR (75.5 MHz, CDCl_3_) δ 142.7 (d, *J* = 7.6), 133.7 (d, *J* = 96.5), 132.1 (d, *J* = 10.2), 132.0 (d, *J* = 2.6), 131.6 (d, *J* = 11.1), 131.5 (d, *J* = 3.1), 130.9 (d, *J* = 9.5), 130.2 (*J* = not visible), 128.6 (d, *J* = 11.6), 125.5 (d, *J* = 11.8), 22.7 (d, *J* = 73.4), 21.5 (d, *J* = 4.3), 5.8 (d, *J* = 4.9); HRMS (ESI/TOF) *m*/*z*: [M + H]^+^ Calcd for C_15_H_18_OP 245.1095; Found 245.1085.

#### (*S*)-Benzyl-(2-methylphenyl)-phenylphosphine Oxide [(*S*)-**3c**]

(*S*)-Benzyl-(2-methylphenyl)phosphine oxide [(*S*)-**3c**] was prepared according to Representative Procedure IV by reacting 12 mg (0.48 mmol) of NaH in 1.0 mL of anhydrous THF with 80 mg (0.37 mmol) of (*S*)-(2-methylphenyl)-phenylphosphine oxide [(*S*)-**1a**, ee = 98%] in 1.0 mL of anhydrous THF and 53 μL (0.44 mmol) of BnBr in 1.0 mL of anhydrous THF at 0 °C. The crude product was purified by flash column chromatography (silica gel, gradient elution, CHCl_3_ to CHCl_3_–MeOH 95:5) to give 86 mg (76%) of (*S*)-benzyl-(2-methylphenyl)-phenylphosphine oxide [(*S*)-**3c**] with an ee of 96% as a white solid. mp: 167–170 °C; [α]_D_^25^ = −84.9 (*c* = 1.01, CHCl_3_, ee = 96%, *S*_P_) ([α]_lit_ = +29.8 (*c* = 1, CHCl_3_, *R*_P_));^52^ Chiral HPLC: Phenomenex Lux 5 μm Amylose-2 column, hexane/ethanol (85:15), *t*_R1_ 17.2 min (*S*), *t*_R2_ 24.3 min (*R*). ^31^P NMR (121.5 MHz, CDCl_3_) δ 31.0; ^1^H NMR (500 MHz, CDCl_3_) δ 7.70–7.66 (m, 1H), 7.51–7.35 (m, 6H), 7.29–7.17 (m, 5H), 7.09–7.07 (m, 2H), 3.80–3.61 (m, 2H), 2.35 (s, 3H); ^13^C{^1^H} NMR (75.5 MHz, CDCl_3_) δ 143.1 (d, *J* = 7.7), 133.2 (d, *J* = 98.6), 132.2 (d, *J* = 8.7), 132.1, 131.7, 131.6 (d, *J* = 7.9), 131.3 (d, *J* = 7.8), 131.1 (d, *J* = 9.5), 130.6 (d, *J* = 97.6), 130.4 (d, *J* = 5.2), 128.5 (d, *J* = 11.6), 128.4, 126.8 (d, *J* = 3.0), 125.5 (d, *J* = 12.0), 37.8 (d, *J* = 66.7), 21.5 (d, *J* = 4.2); HRMS (ESI/TOF) *m*/*z*: [M + H]^+^ Calcd for C_20_H_20_OP 307.1252; Found 307.1252.

### Preparation of (*S*)-(1-Naphthyl)-(2-methylphenyl)-phenylphosphine Oxide [(*S*)-**3d**]

A solution of 43 μL (0.41 mmol) of 1-bromonaphthalene, 0.10 g (0.74 mmol) of anhydrous K_2_CO_3_, and 22 mg (0.019 mmol) of Pd(PPh_3_)_4_ in 1.0 mL of anhydrous toluene was stirred at 25 °C under argon atmosphere for 30 min. Then, 80 mg (0.37 mmol) of (*S*)-(2-methylphenyl)-phenylphosphine oxide [(*S*)-**1a**, ee = 98%] in 1.0 mL of anhydrous toluene was added dropwise. The reaction mixture was stirred at 110 °C for 22 h using an oil bath as the heat source. After cooling to 25 °C, it was filtered through a short plug of silica gel, washed with EtOAc, and evaporated. The crude product was purified by flash column chromatography (silica gel, gradient elution, hexane to hexane–EtOAc 20:80) to give 107 mg (85%) of (*S*)-(1-naphthyl)-(2-methylphenyl)-phenylphosphine oxide [(*S*)-**3d**] with an ee of 88% as a white solid. mp 87–92 °C; [α]_D_^25^ = −7.8 (*c* = 1.00, CHCl_3_, ee = 88%, *S*_P_) ([α]_lit_ = −8 (*c* = 1, CHCl_3_, *S*_P_));^52^ Chiral HPLC: Kromasil 5-Amycoat column, hexane/ethanol (80:20), *t*_R1_ 8.7 min (*R*), *t*_R2_ 11.1 min (*S*); ^31^P{^1^H} NMR (121.5 MHz, CDCl_3_) δ 35.6; ^1^H NMR (500 MHz, CDCl_3_) δ 8.72 (d, 1H, *J* = 8.4), 8.00 (d, 1H, *J* = 8.1), 7.88 (d, 1H, *J* = 7.9), 7.68–7.64 (m, 2H), 7.56–7.35 (m, 7H), 7.32–7.26 (m, 2H), 7.07 (dt, 1H, *J* = 7.7, 3.9), 7.03–6.98 (m, 1H), 2.56 (s, 3H); ^13^C{^1^H} NMR (125.8 MHz, CDCl_3_) δ 143.5 (d, *J* = 7.9), 134.1 (d, *J* = 1.8), 134.0, 133.6 (d, *J* = 97.2), 133.4 (d, *J* = 12.9), 133.2 (d, *J* = 2.9), 133.1 (d, *J* = 11.9), 132.4 (d, *J* = 9.8), 132.1 (d, *J* = 5.5), 132.0 (d, *J* = 7.6), 131.9 (d, *J* = 2.8), 131.0 (d, *J* = 103.2), 129.1 (d, *J* = 101.2), 128.8, 128.6 (d, *J* = 12.1), 127.9 (d, *J* = 5.3), 127.4, 126.6, 125.4 (d, *J* = 12.9), 124.4 (d, *J* = 14.3), 22.0 (d, *J* = 4.4); HRMS (ESI/TOF) *m*/*z*: [M + H]^+^ Calcd for C_23_H_20_OP 343.1252; Found 343.1238.

### Preparation of (*R*)-(2-Methylphenyl)-phenylphosphothioic Acid [(*R*)-**3e**]

To a solution of 58 mg (0.27 mmol) of (*S*)-(2-methylphenyl)-phenylphosphine oxide [(*S*)-**1a**, ee = 98%] in 0.54 mL of anhydrous THF was added 8.6 mg (0.27 mmol) of sulfur. Using an oil bath, the reaction mixture was heated at 67 °C under argon atmosphere for 1.5 h; then, the solvent was evaporated. The residue was crystallized from 1.1 mL of hexane to afford 53 mg (80%) of (*R*)-(2-methylphenyl)-phenylphosphothioic acid [(*R*)-**3e**] with an ee of >98% as a white solid. mp: 103–104 °C; [α]_D_^25^ = +48.3 (*c* = 0.97, CHCl_3_, ee = 99%, *R*_P_); ^31^P{^1^H} NMR (202.5 MHz, CDCl_3_) δ 75.3; ^1^H NMR (500 MHz, CDCl_3_) δ 7.98 (dd, 1H, *J* = 15.2, 7.7), 7.80–7.76 (m, 2H), 7.48 (dt, 1H, *J* = 7.4, 4.5), 7.42–7.38 (m, 3H), 7.29–7.27 (m, 1H), 7.17 (t, 1H, *J* = 6.4), 2.32 (s, 3H); ^13^C{^1^H} NMR (125.8 MHz, CDCl_3_) δ 141.0 (d, *J* = 11.7), 135.4 (d, *J* = 108.3), 132.5 (d, *J* = 11.6), 132.5 (d, *J* = 109.3), 132.3 (d, *J* = 3.0), 132.0 (d, *J* = 5.9), 131.9 (d, *J* = 3.4), 131.0 (d, *J* = 12.2), 128.5 (d, *J* = 13.7), 125.7 (d, *J* = 13.4), 21.7 (d, *J* = 4.9); HRMS (ESI/TOF) *m*/*z*: [M + H]^+^ Calcd for C_13_H_14_OPS 249.0503; Found 249.0497.

### Preparation of (*S*)-Hydroxymethyl-(2-methylphenyl)-phenylphosphine Oxide [(*S*)-**3f**]

To 2.6 mL of NaOH solution (0.05 M) were added 61 mg (0.28 mmol) of (*S*)-(2-methylphenyl)-phenylphosphine oxide [(*S*)-**1a**, ee = 98%] and 25 μL (0.34 mmol) of 37% formaldehyde solution at 25 °C. The reaction mixture was heated in an oil bath at 80 °C for 16 h and then cooled to 25 °C. Two mL of DCM was added; the phases were separated, and the aqueous layer was extracted with DCM (3 × 2 mL). The organic layers were combined, dried (Na_2_SO_4_), and evaporated to give 72 mg (98%) of (*S*)-hydroxymethyl-(2-methylphenyl)-phenylphosphine oxide [(*S*)-**3f**] with an ee of 96% as a white solid. mp: 140–142 °C; [α]_D_^25^ = −42.7 (*c* = 1.00, CHCl_3_, ee = 96%, *S*_P_); Chiral HPLC: Phenomenex Lux 3 μm Cellulose-4 column, hexane/ethanol (50:50), *t*_R1_ 8.6 min (*S*), *t*_R2_ 9.2 min (*R*); ^31^P{^1^H} NMR (202.5 MHz, DMSO-*d*_6_) δ 29.9; ^1^H NMR (500 MHz, DMSO-*d*_6_) δ 7.78–7.74 (m, 1H), 7.64–7.44 (m, 6H), 7.35–7.26 (m, 2H), 5.76 (s, 1H), 4.34–4.22 (m, 2H), 2.30 (s, 3H); ^13^C{^1^H} NMR (125.8 MHz, DMSO-*d*_6_) δ 141.9 (d, *J* = 7.2), 132.7 (d, *J* = 92.9), 131.9, 131.8 (d, *J* = 9.0), 131.5 (d, *J* = 6.0), 131.6, 131.0 (d, *J* = 9.0), 130.3 (d, *J* = 93.1), 128.4 (d, *J* = 11.0), 125.5 (d, *J* = 11.8), 60.2 (d, *J* = 87.3), 20.6 (d, *J* = 4.2); HRMS (ESI/TOF) *m*/*z*: [M + H]^+^ Calcd for C_14_H_16_O_2_P 247.0888; Found 247.0884.

### Preparation of (*S*_P_)-[(*R*_C_)-Hydroxy(phenyl)methyl]-(2-methylphenyl)-phenylphosphine oxide [(*S*_P_,*R*_C_)-**3g**]

To 2.3 mL of NaOH solution (0.05 M) were added 54 mg (25 mmol) of (*S*)-(2-methylphenyl)-phenylphosphine oxide [(*S*)-**1a**, ee = 98%] and 30 μL (0.30 mmol) of benzaldehyde at 25 °C. The reaction mixture was heated in an oil bath at 80 °C for 16 h and then cooled to 25 °C. The resulting white suspension was filtered and washed with 2.3 mL of water and 2.3 mL of hexane to give 64 mg (80%) of (*S*_P_)-[(*R*_C_)-hydroxy(phenyl)methyl]-(2-methylphenyl)-phenylphosphine oxide [(*S*_P_,*R*_C_)-**3g**] with an ee of 99% and de of 95% as a white solid. mp: 178–179 °C; [α]_D_^25^ = −83.3 (*c* = 0.28, MeOH, ee = 99%, *S*_P_,*R*_C_); Chiral HPLC: Phenomenex Lux 5 μm Amylose-2 column, hexane/ethanol (85:15), *t*_R1_ 10.2 min (*R*_P_,*S*_C_), *t*_R2_ 24.5 min (*S*_P_,*R*_C_); ^31^P{^1^H} NMR (121.5 MHz, DMSO-*d*_6_) δ 31.6 (major diastereomer, 97.5%), 31.3 (minor diastereomer, 2.5%); ^1^H NMR (500 MHz, DMSO-*d*_6_) δ 7.96–7.92 (m, 1H), 7.55–7.18 (m, 13H), 6.49 (dd, 1H, *J* = 17.2, 5.9), 5.62–5.60 (m, 1H), 2.18 (s, 3H); ^13^C{^1^H} NMR (75.5 MHz, DMSO-*d*_6_) δ 143.4 (d, *J* = 6.8), 138.5, 133.8 (d, *J* = 90.2), 133.1 (d, *J* = 8.7), 132.1, 132.0 (d, *J* = 7.8), 131.8 (d, *J* = 2.5), 131.6 (d, *J* = 8.8), 129.7 (d, *J* = 91.5), 128.6 (d, *J* = 11.1), 128.4 (d, *J* = 4.5), 127.8 (d, *J* = 1.9), 127.8 (d, *J* = 2.4), 125.5 (d, *J* = 11.7), 72.7 (d, *J* = 85.9), 21.3 (d, *J* = 3.7); HRMS (ESI/TOF) *m*/*z*: [M + H]^+^ Calcd for C_20_H_20_O_2_P 323.1201; Found 323.1197.

### X-ray Crystallography

#### Diastereomeric Complex (*S*)-**1a**·(spiro-TADDOL)

X-ray quality crystals were prepared by slowly diffusing hexane to the toluene solution of the (*S*)-**1a**·(spiro-TADDOL) diastereomeric complex.

Crystal data: C_34_H_34_O_4_, C_13_H_13_OP, Fwt.: 722.81, colorless, needle, size: 0.500 × 0.040 × 0.040 mm, monoclinic, space group *P*21, *a* = 10.553(3) Å, *b* = 9.719(3) Å, *c* = 18.977(6) Å, α = 90°, β = 93.918(7)°, γ = 90°, *V* = 1941.8(11) Å^3^, *T* = 294(2) K, *Z* = 2, *F*(000) = 768, *D*_*x*_ = 1.236 Mg/m^3^, μ 0.118 mm^–1^. A crystal of (*S*)-**1a**·(spiro-TADDOL) was mounted on a fiber. Cell parameters were determined by least-squares using 10 345 (2.99° ≤ θ ≤ 25.275°) reflections. Intensity data were collected on a Rigaku RAXIS-RAPID II diffractometer (monochromator; Mo Kα radiation, λ = 0.71075 Å) at 293(2) K in the range of 2.991° ≤ θ ≤ 21.967°. A total of 20 355 reflections were collected of which 5047 were unique [*R*(int) = 0.2604, *R*(σ) = 0.2178]; intensities of 2392 reflections were greater than 2σ (*I*). Completeness to θ = 0.997. A NUMABS absorption correction was applied to the data (the minimum and maximum transmission factors were 0.965396 and 0.995849). The structure was solved by direct methods (and subsequent difference syntheses). Anisotropic full-matrix least-squares refinement on *F*^2^ for all non-hydrogen atoms yielded *R*_1_ = 0.0889 and *wR*^2^ = 0.1329 for 1332 [*I* > 2σ (*I*)] and *R*_1_ = 0.1869 and *wR*^2^ = 0.1668 for all (5047) intensity data (number of parameters = 481, goodness-of-fit = 0.960, the maximum and mean shifts/esd are 0.001 and 0.000). The absolute structure parameter is 0.0(3) (Friedel coverage: 0.867; Friedel fraction max.: 0.999; Friedel fraction full: 0.999). The maximum and minimum residual electron densities in the final difference map were 0.222 and −0.219 e·Å^–3^. The weighting scheme applied was *w* = 1/[σ^2^(*F*_o_^2^) + (0.00390.0000*P*)^2^ + 0.0000*P*] where *P* = (*F*_o_^2^ + 2*F*_c_^2^)/3.

#### (*S*_P_)-[(*R*_C_)-Hydroxy(phenyl)methyl]-(2-methylphenyl)-phenylphosphine Oxide [(*S*_P_,*R*_C_)-**3g**]

X-ray quality crystals were prepared by the slow evaporation of the solvent from the dichloromethatne solution of (*S*_P_,*R*_C_)-**3g**.

Crystal data: C_20_H_19_O_2_P, Fwt.: 322.32, colorless, platelet, size: 0.300 × 0.100 × 0.100 mm, orthorhombic, space group *P*212121, *a* = 8.1680(7) Å, *b* = 9.9573(9) Å, *c* = 20.1721(17) Å, α = 90°, β = 90°, γ = 90°, *V* = 1640.6(2) Å^3^, *T* = 294(2) K, *Z* = 4, *F*(000) = 680, *D*_*x*_ = 1.305 Mg/m^3^, μ 0.175 mm^–1^. A crystal of (*S*_P_,*R*_C_)-**3g** was mounted on a fiber. Cell parameters were determined by least-squares using 25 498 (3.21° ≤ θ ≤ 27.475°) reflections. Intensity data were collected on a Rigaku RAXIS-RAPID II diffractometer (monochromator; Mo Kα radiation, λ = 0.71075 Å) at 293(2) K in the range of 3.209° ≤ θ ≤ 25.350°. A total of 32 784 reflections were collected of which 2996 were unique [*R*(int) = 0.0807, *R*(σ) = 0.0367]; intensities of 2614 reflections were greater than 2σ (*I*). Completeness to θ = 0.998. A numerical absorption correction was applied to the data (the minimum and maximum transmission factors were 0.993616 and 0.998669). The structure was solved by direct methods (and subsequent difference syntheses). Anisotropic full-matrix least-squares refinement on *F*^2^ for all non-hydrogen atoms yielded *R*_1_ = 0.0500 and *wR*^2^ = 0.0854 for 1332 [*I* > 2σ(*I*)] and *R*_1_ = 0.0620 and *wR*^2^ = 0.0893 for all (2996) intensity data (number of parameters = 210, goodness-of-fit = 1.122, the maximum and mean shifts/esd are 0.000 and 0.000). The absolute structure parameter is 0.05(4) (Friedel coverage: 0.725; Friedel fraction max.: 1.000; Friedel fraction full: 1.000). The maximum and minimum residual electron densities in the final difference map were 0.209 and −0.196 e·Å^–3^. The weighting scheme applied was *w* = 1/[σ^2^(*F*_o_^2^) + (0.03550.2831*P*)^2^ + 0.2831*P*] where *P* = (*F*_o_^2^ + 2*F*_c_^2^)/3. The crystallographic data are in Table S6.

## Supporting Information Available

Conditions and results of the resolution experiments; optical rotation values [α] of secondary phosphine oxides (**1**) and the assignation of their absolute configuration; X-ray measurements; NMR spectra; HPLC traces (PDF)

## Accession Codes

CCDC 2081817 and 2081818 contain the supplementary crystallographic data for this paper. These data can be obtained free of charge via www.ccdc.cam.ac.uk/data_request/cif, or by emailing data_request@ccdc.cam.ac.uk, or by contacting The Cambridge Crystallographic Data Centre, 12 Union Road, Cambridge CB2 1EZ, UK; fax: +44 1223 336033.
