# Supplementary material for: Enantioseparation of P-Stereogenic Secondary Phosphine Oxides and Their Stereospecific Transformation to Various Tertiary Phosphine Oxides and a Thiophosphinate
Source: J Org Chem. 2021 Oct 11;86(21):14493–507. doi: 10.1021/acs.joc.1c01364 (PMC8576816; doi:10.1021/acs.joc.1c01364)
Supplement: Supplementary file 1 — jo1c01364_si_001.pdf [file jo1c01364_si_001.pdf]

Supporting Information

Enantioseparation of P-stereogenic secondary phosphine oxides and their stereospecific transformation to various tertiary phosphine oxides and a thiophosphinate

Bence Varga,[a] Péter Szemesi,[a,b] Petra Nagy,[a] Réka Herbay,[a] Tamás Holczbauer,[c] Elemér Fogassy,[a] György Keglevich,[a] Péter Bagi[a,*]

[a] *Department of Organic Chemistry and Technology, Budapest University of Technology and Economics, Műegyetem rkp. 3., H-1111 Budapest, Hungary*

[b] *Gedeon Richter Plc., Budapest, Hungary*

[c] Center for Structural Science, Chemical Crystallography Research Laboratory and Institute for Organic Chemistry, Research Centre for Natural Sciences, Magyar tudósok körútja 2., H-1519 Budapest, Hungary

* Corresponding author. Tel.: +36 1 4631111/5886; fax: +36 1 4633648. Email address: bagi.peter@vbk.bme.hu

Table of Contents

Conditions and results of resolution experiments S2

Optical rotation values [α] of secondary phosphine oxides (**1**) and the assignation of their absolute configuration S7

Supplementary information for X-Ray measurements S8

^{31}P, ^{19}F, ^{1}H and ^{13}C NMR spectra of the compounds prepared S11

HPLC traces of the optically active secondary and tertiary phosphine oxides (**1** and **3**) S55

References S80

1. Conditions and results of resolution experiments

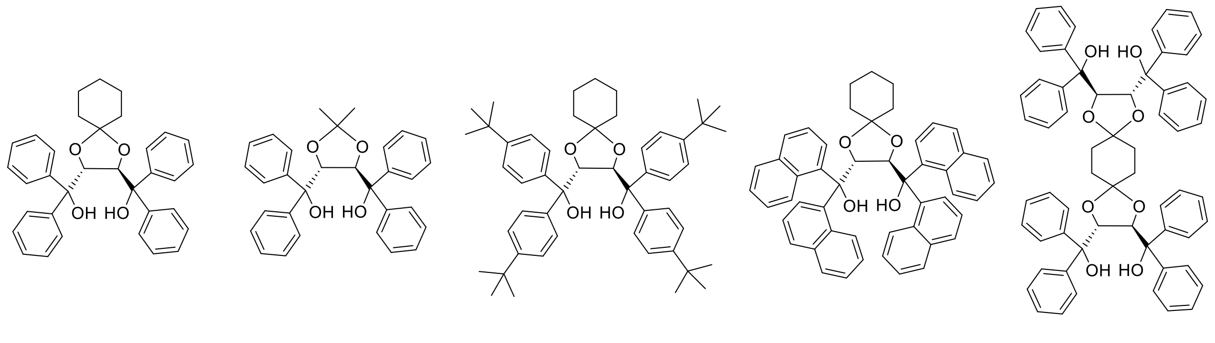

(*R*,*R*)-**2** spiro-TADDOL | (*R*,*R*)-**SI-1** TADDOL | (*R*,*R*)-**SI-2** | (*R*,*R*)-**SI-3** | (*R*,*R*,*R*,*R*)-**SI-4**

Figure S1 TADDOL-derivatives [(*R*,*R*)-**2,** (*R*,*R*)-**SI-1-3** and (*R*,*R*,*R*,*R*)-**SI-4**] used as resolving agents for the enantioseparation of (2-methylphenyl)-phenylphosphine oxide (**1a**)

Table S1 Resolution of (2-methylphenyl)-phenylphosphine oxide (**1a**) with TADDOL-derivatives [(*R*,*R*)-**2,** (*R*,*R*)-**SI-1-3** and (*R*,*R*,*R*,*R*)-**SI-4**]

Entry	Res. agent	Eq.	Solvent[a]	Diastereomeric complex[b]	Y[c,f] (%)	ee[d,f] (%)	S[e,f] (-)	Abs. config.[g]
1	(*R*,*R*)-**SI-1**	0.5	2×EtOAc<br>10×hexane	(**1a**)·(TADDOL)	(65)	(0)	(0.00)	-
2	(*R*,*R*)-**SI-1**	0.5	6×2-PrOH	(**1a**)$_3$·(TADDOL)$_2$	(124)	(1)	(0.01)	-
3	(*R*,*R*)-**SI-1**	0.5	5×toluene<br>5×hexane	(**1a**)·(TADDOL)	(45)	(5)	(0.02)	(*S*)
4	(*R*,*R*)-**2**	0.5	2×EtOAc<br>10×hexane	(**1a**)·(spiro-TADDOL)	(105)	(2)	(0.02)	(*S*)
5	(*R*,*R*)-**2**	0.5	5×toluene<br>5×hexane	(**1a**)·(spiro-TADDOL)	(75)<br>**55**	(77)<br>**95**	(0.58)<br>**0.52**	(*S*)
6	(*R*,*R*)-**2**	1	5×toluene<br>5×hexane	(**1a**)·(spiro-TADDOL)$_2$	(83)<br>**42**	(20)<br>**90**	(0.17)<br>**0.38**	(*S*)
7	(*R*,*R*)-**2**	2	5×toluene<br>5×hexane	(**1a**)·(spiro-TADDOL)$_2$	(119)<br>**52**	(30)<br>**77**	(0.36)<br>**0.40**	(*S*)
8	(*R*,*R*)-**2**	2	6×2-Pr$_2$O	(**1a**)·(spiro-TADDOL)$_2$	(96)<br>**38**	(44)<br>**80**	(0.42)<br>**0.30**	(*S*)
9	(*R*,*R*)-**2**	2	5×acetone	(**1a**)·(spiro-TADDOL)$_4$	(83)	(10)	(0.08)	(*S*)
10	(*R*,*R*)-**2**	0.5	30×H_2O	(**1a**)·(spiro-TADDOL)	(106)	(33)	(0.35)	(*S*)
11	(*R*,*R*)-**2**	1	6×MeOH	(**1a**)·(spiro-TADDOL)$_2$	(71)<br>**30**	(62)<br>**87**	(0.44)<br>**0.26**	(*S*)
12	(*R*,*R*)-**2**	1	6×EtOH	(**1a**)·(spiro-TADDOL)$_2$	(88)<br>**13**	(69)<br>**90**	(0.61)<br>**0.12**	(*S*)
13	(*R*,*R*)-**2**	1	6×2-PrOH	(**1a**)·(spiro-TADDOL)$_2$	(89)<br>**65**	(79)<br>**98**	(0.70)<br>**0.63**	(*S*)
14	(*R*,*R*)-**2**	2	6×2-PrOH	(**1a**)·(spiro-TADDOL)$_2$	(157)<br>**53**	(15)<br>**93**	(0.24)<br>**0.49**	(*S*)
15	(*R*,*R*)-**SI-2**	0.5	5×toluene<br>5×hexane	no complex	-	-	-	-
16	(*R*,*R*)-**SI-2**	0.5	6×2-PrOH	no complex	-	-	-	-
17[h]	(*R*,*R*)-**SI-3**	0.5	5×toluene<br>5×hexane	(**1a**)·(*R*,*R*)-**SI-3**	(56)	(75)	(0.42)	(*S*)
18	(*R*,*R*)-**SI-3**	1	6×2-PrOH	(**1a**)·[(*R*,*R*)-**SI-3**]$_2$	(33)	(0)	(0)	-
19	(*R*,*R*,*R*,*R*)-<br>**SI-4**	0.5	5×toluene<br>5×hexane	no complex	-	-	-	-
20	(*R*,*R*,*R*,*R*)-<br>**SI-4**	0.5	6×2-PrOH	(**1a**)·(*R*,*R*,*R*,*R*)-**SI-4**	(98)<br>**81**	(27)<br>**49**	(0.26)<br>**0.40**	(*S*)

[a]Mixture of solvents for the crystallization and recrystallizations [mL of solvent/g of resolving agent].
[b]The ratio of secondary phosphine oxide (**1**) and the resolving agent was determined by ^{1}H NMR.
[c]The yield of the diastereomer was calculated based on the half of the racemic secondary phosphine oxide (**1**) that is regarded to be 100% for each antipode.

[d]Determined by HPLC using a chiral stationary phase.
[e]Resolving capability, also known as the Fogassy parameter [S (-) = (Yield [%] /100)×(*ee* [%]/100)].[1]
[f]The results obtained after the first crystallization are shown in parentheses, while the results obtained after two recrystallizations are shown in boldface.
[g]The absolute configuration was assigned by either X-ray analysis, specific rotation or chiral chromatography according to literature data (See Table S5).
[h]The diastereomer could not purified by recrystallizations.

Table S2 Resolution of secondary phosphine oxides (**1a-n**) with spiro-TADDOL [(*R*,*R*)-**2**] using 2-PrOH as solvent.

Entry	Y	Solvent[a]	Eq. of (*R*,*R*)-**2**	Diastereomeric complex[b]	Y[c,f] (%)	ee[d,f] (%)	S[e,f] (-)	Abs. config.[g]
1	2-Me-C_6H_4 (**1a**)	6×2-PrOH	1	(**1a**)·(spiro-TADDOL)$_2$	(89) **65**	(79) **98**	(0.70) **0.63**	(*S*)
2	2-Me-C_6H_4 (**1a**)	6×2-PrOH	2	(**1a**)·(spiro-TADDOL)$_2$	(157) **53**	(15) **93**	(0.24) **0.49**	(*S*)
3	3-Me-C_6H_4 (**1b**)	6×2-PrOH	1	(**1b**)·(spiro-TADDOL)$_2$	(78) **38**	(0) **0**	(0) **0.00**	-
4	3-Me-C_6H_4 (**1b**)	6×2-PrOH	2	(**1b**)·(spiro-TADDOL)$_3$	(114) **25**	(0) **0**	(0) **0.00**	-
5	4-Me-C_6H_4 (**1c**)	6×2-PrOH	1	(**1c**)·(spiro-TADDOL)$_2$	(82) **40**	(63) **99**	(0.51) **0.39**	(−)
6	4-Me-C_6H_4 (**1c**)	6×2-PrOH	2	(**1c**)·(spiro-TADDOL)$_2$	(114) **53**	(29) **95**	(0.32) **0.51**	(−)
7	2-CF_3-C_6H_4 (**1d**)	6×2-PrOH	1	spiro-TADDOL	-	-	-	-
8	2-CF_3-C_6H_4 (**1d**)	6×2-PrOH	2	spiro-TADDOL	-	-	-	-
9	3-CF_3-C_6H_4 (**1e**)	6×2-PrOH	1	spiro-TADDOL	-	-	-	-
10	3-CF_3-C_6H_4 (**1e**)	6×2-PrOH	2	spiro-TADDOL	-	-	-	-
11	4-CF_3-C_6H_4 (**1f**)	6×2-PrOH	1	no complex	-	-	-	-
12	4-CF_3-C_6H_4 (**1f**)	6×2-PrOH	2	no complex	-	-	-	-
13	2-MeO-C_6H_4 (**1g**)	6×2-PrOH	0.5	(**1g**)·(spiro-TADDOL)	(70) **18**	(28) **67**	(0.20) **0.12**	(*R*)
14	2-MeO-C_6H_4 (**1g**)	6×2-PrOH	1	(**1g**)·(spiro-TADDOL)$_2$	(152) **66**	(8) **23**	(0.12) **0.15**	(*R*)
15	2-Ph-C_6H_4 (**1h**)	6×2-PrOH	0.5	(**1h**)·(spiro-TADDOL)	(95) **56**	(10) **15**	(0.09) **0.08**	(*S*)
16	2-Ph-C_6H_4 (**1h**)	6×2-PrOH	1	(**1h**)·(spiro-TADDOL)	(82) **33**	(7) **19**	(0.06) **0.06**	(*S*)
17	1-Naph (**1i**)	6×2-PrOH	1	(**1i**)·(spiro-TADDOL)$_2$	(98) **43**	(9) **38**	(0.08) **0.16**	(*R*)
18	1-Naph (**1i**)	6×2-PrOH	2	(**1i**)·(spiro-TADDOL)$_2$	(152) **65**	(11) **35**	(0.17) **0.23**	(*R*)
19	Bn (**1j**)	6×2-PrOH	1	(**1j**)·(spiro-TADDOL)$_2$	(73) **17**	(4) **46**	(0.03) **0.08**	(*S*)
20[h]	Bn (**1j**)	6×2-PrOH	2	(**1j**)·(spiro-TADDOL)$_4$	(72) **35**	(39) **87**	(0.28) **0.31**	(*S*)
21	Me (**1k**)	6×2-PrOH	1	(**1k**)·(spiro-TADDOL)$_2$	(66) **14**	(60) **99**	(0.40) **0.14**	(*R*)
22	Me (**1k**)	6×2-PrOH	2	(**1k**)·(spiro-TADDOL)$_2$	(97) **27**	(61) **99**	(0.59) **0.26**	(*R*)
23[i]	Bu (**1l**)	6×2-PrOH	1	(**1l**)·(spiro-TADDOL)$_2$	(44)	(5)	(0.02)	(*S*)
24	Bu (**1l**)	6×2-PrOH	2	no complex	-	-	-	-
25	^{t}Bu (**1m**)	6×2-PrOH	0.5	(**1m**)·(spiro-TADDOL)	(86) **41**	(67) **98**	(0.57) **0.40**	(*R*)
26	^{t}Bu (**1m**)	6×2-PrOH	1	(**1m**)·(spiro-TADDOL)	(114) **35**	(53) **98**	(0.61) **0.34**	(*R*)
27	^{c}Hex (**1n**)	6×2-PrOH	1	(**1n**)·(spiro-TADDOL)$_2$	(76) **14**	(8) **27**	(0.06) **0.04**	(*S*)
28[h]	^{c}Hex (**1n**)	6×2-PrOH	2	(**1n**)·(spiro-TADDOL)$_2$	(138) **73**	(6) **15**	(0.08) **0.11**	(*S*)

[a-g]See Table S1.
[h]The diastereomer was purified by one crystallization.
[i]The diastereomer was not purified.

Table S3 Resolution of secondary phosphine oxides (**1a-n**) with spiro-TADDOL [(*R*,*R*)-**2**] using toluene and hexane as solvent.

Entry	Y	Solvent[a]	Eq. of (*R*,*R*)-**2**	Diastereomeric complex[b]	Y[c,f] (%)	ee[d,f] (%)	S[e,f] (-)	Abs. config.[g]
1	2-Me-C_6H_4 (**1a**)	5×toluene 5×hexane	0.5	(**1a**)·(spiro-TADDOL)	(75) **55**	(77) **95**	(0.58) **0.52**	(*S*)
2	2-Me-C_6H_4 (**1a**)	5×toluene 5×hexane	1	(**1a**)·(spiro-TADDOL)$_2$	(83) **42**	(20) **90**	(0.17) **0.38**	(*S*)
3	3-Me-C_6H_4 (**1b**)	5×toluene 5×hexane	0.5	(**1b**)·(spiro-TADDOL)	(76) **14**	(16) **62**	(0.12) **0.08**	(−)
4	3-Me-C_6H_4 (**1b**)	5×toluene 5×hexane	1	(**1b**)·(spiro-TADDOL)	(107) **8**	(11) **62**	(0.12) **0.05**	(−)
5[h]	4-Me-C_6H_4 (**1c**)	5×toluene 5×hexane	0.5	(**1c**)·(spiro-TADDOL)	(37) **9**	(4) **6**	(0.01) **0.01**	(−)
6	4-Me-C_6H_4 (**1c**)	5×toluene 5×hexane	1	(**1c**)·(spiro-TADDOL)	(116) **68**	(2) **12**	(0.02) **0.08**	(−)
7	2-CF_3-C_6H_4 (**1d**)	5×toluene 5×hexane	0.5	no complex	-	-	-	-
8	2-CF_3-C_6H_4 (**1d**)	5×toluene 5×hexane	1	no complex	-	-	-	-
9[i]	3-CF_3-C_6H_4 (**1e**)	5×toluene 5×hexane	0.5	(**1e**)·(spiro-TADDOL)	(37)	(49)	(0.18)	(+)
10[h]	3-CF_3-C_6H_4 (**1e**)	5×toluene 5×hexane	1	(**1e**)·(spiro-TADDOL)	(61) **1**	(55) **79**	(0.34) **0.01**	(+)
11[h]	3-CF_3-C_6H_4 (**1e**)	3×toluene 3×hexane	1	(**1e**)·(spiro-TADDOL)	(82) **16**	(32) **79**	(0.26) **0.13**	(+)
12	4-CF_3-C_6H_4 (**1f**)	5×toluene 5×hexane	0.5	(**1f**)·(spiro-TADDOL)	(52) **12**	(53) **99+**	(0.27) **0.12**	(+)
13[h]	4-CF_3-C_6H_4 (**1f**)	5×toluene 5×hexane	1	(**1f**)·(spiro-TADDOL)	(64) **29**	(79) **99**	(0.51) **0.29**	(+)
14	2-MeO-C_6H_4 (**1g**)	5×toluene 5×hexane	0.5	(**1g**)·(spiro-TADDOL)	(148) **51**	(9) **46**	(0.13) **0.23**	(*R*)
15	2-MeO-C_6H_4 (**1g**)	5×toluene 5×hexane	1	(**1g**)·(spiro-TADDOL)	(150) **98**	(8) **28**	(0.13) **0.28**	(*R*)
16	2-Ph-C_6H_4 (**1h**)	5×toluene 5×hexane	0.5	(**1h**)·(spiro-TADDOL)	(78) **24**	(13) **40**	(0.10) **0.10**	(*S*)
17	2-Ph-C_6H_4 (**1h**)	5×toluene 5×hexane	1	(**1h**)·(spiro-TADDOL)	(152) **81**	(7) **21**	(0.11) **0.17**	(*S*)
18	1-Naph (**1i**)	5×toluene 5×hexane	0.5	(**1i**)·(spiro-TADDOL)	(79) **26**	(19) **72**	(0.15) **0.19**	(*R*)
19	1-Naph (**1i**)	7.5×toluene 7.5×hexane	0.5	(**1i**)·(spiro-TADDOL)	(77) **14**	(33) **83**	(0.25) **0.12**	(*R*)
20	1-Naph (**1i**)	5×toluene 5×hexane	1	(**1i**)·(spiro-TADDOL)	(143) **31**	(6) **71**	(0.08) **0.22**	(*R*)
21	Bn (**1j**)	5×toluene 5×hexane	0.5	(**1j**)·(spiro-TADDOL)	(97) **66**	(6) **14**	(0.05) **0.09**	(*S*)
22	Bn (**1j**)	5×toluene 5×hexane	1	(**1j**)·(spiro-TADDOL)$_2$	(90) **57**	(3) **16**	(0.02) **0.09**	(*S*)
23	Me (**1k**)	5×toluene 5×hexane	0.5	(**1k**)·(spiro-TADDOL)	(86) **50**	(30) **70**	(0.26) **0.35**	(*R*)
24	Me (**1k**)	5×toluene 5×hexane	1	(**1k**)·(spiro-TADDOL)	(115) **59**	(16) **59**	(0.18) **0.35**	(*R*)
25[i]	Bu (**1l**)	5×toluene 5×hexane	0.5	(**1l**)·(spiro-TADDOL)	(22)	(45)	(0.10)	(*S*)
26[i]	Bu (**1l**)	5×toluene 5×hexane	1	(**1l**)·(spiro-TADDOL)	(66)	(36)	(0.23)	(*S*)
27	^{t}Bu (**1m**)	5×toluene 5×hexane	0.5	(**1m**)·(spiro-TADDOL)	(89) **64**	(66) **90**	(0.58) **0.58**	(*R*)
28	^{t}Bu (**1m**)	5×toluene 5×hexane	1	(**1m**)·(spiro-TADDOL)	(133) **70**	(33) **90**	(0.44) **0.63**	(*R*)
29[i]	^{c}Hex (**1n**)	5×toluene 5×hexane	0.5	(**1n**)·(spiro-TADDOL)	(23)	(76)	(0.17)	(*S*)
30[h]	^{c}Hex (**1n**)	5×toluene 5×hexane	1	(**1n**)·(spiro-TADDOL)	(68) **12**	(36) **92**	(0.25) **0.11**	(*S*)

[a-i]See Tables S1 and S2.

Table S4 Resolution of selected secondary phosphine oxides (**1g, 1i** and **1m**) with with TADDOL-derivatives [(*R*,*R*)-**SI-1-3** and (*R*,*R*,*R*,*R*)-**SI-4**]

Entry	Y	Solvent[a]	Res. agent	Eq.	Diastereomeric complex[b]	Y[c,f] (%)	ee[d,f] (%)	S[e,f] (-)	Abs. config.[g]
1	2-MeO-C_6H_4 (**1g**)	5×toluene 5×hexane	(*R*,*R*)-**SI-3**	0.5	no complex	-	-	-	-
2	2-MeO-C_6H_4 (**1g**)	6×2-PrOH	(*R*,*R*,*R*,*R*)-**SI-4**	0.5	no complex	-	-	-	-
3	1-Naph (**1i**)	5×toluene 5×hexane	(*R*,*R*)-**SI-3**	0.5	no complex	-	-	-	-
4	1-Naph (**1i**)	12×2-PrOH	(*R*,*R*,*R*,*R*)-**SI-4**	0.5	(**1i**)·(*R*,*R*,*R*,*R*)-**SI-4**	(85) **41**	(51) **70**	(0.43) **0.29**	(*R*)
5	^{t}Bu (**1m**)	5×toluene 5×hexane	(*R*,*R*)-**SI-3**	0.5	no complex	-	-	-	
6	^{t}Bu (**1m**)	6×2-PrOH	(*R*,*R*,*R*,*R*)-**SI-4**	0.5	(**1m**)·(*R*,*R*,*R*,*R*)-**SI-4**	(86)	(0)	(0)	-

[a-g]See Table S1.

2. Optical rotation values [α] of secondary phosphine oxides (1) and the assignation of their absolute configuration

Table S5 The optical rotation values [α] of secondary phosphine oxides (**1**) and the assignation of their absolute configuration.

Compound	[α]lit. (*R*)	[α]lit. (*S*)	[α]measured	Abs. configuration
**1a**	$[\alpha]_D^{25}$ = +22.4 (c = 1.17, $CHCl_3$, *ee* = 74%)[2] Source of chirality: Stereospecific reaction	-	$[\alpha]_D^{25}$ = −44.2 (c = 1.07, $CHCl_3$, *ee* = 98%)	(*S*)[a]
**1b**	-	-	$[\alpha]_D^{25}$ = −1.3 (c = 1.35, $CHCl_3$, *ee* = 62%)	
**1c**	-	-	$[\alpha]_D^{25}$ = −7.2 (c = 0.72, $CHCl_3$, *ee* = 99%)	
**1d**	-	-	not measured, *ee* = 0%	
**1e**	-	-	$[\alpha]_D^{25}$ = +10.0 (c = 0,78, $CHCl_3$, *ee* = 79%)	
**1f**	-	-	$[\alpha]_D^{25}$ = +13.0 (c = 0.68, $CHCl_3$, *ee* = 99%)	
**1g**	known compound, but [α] was not measured[2] Source of chirality: Stereospecific reaction	-	$[\alpha]_D^{25}$ = +40.9 (c = 0.91, $CHCl_3$, *ee* = 67%)	(*R*)[b]
**1h**	known compound, but [α] was not measured[3] Source of chirality: Stereospecific reaction	-	$[\alpha]_D^{25}$ = −30.4 (c = 0.58, $CHCl_3$, *ee* = 40%)	(*S*)[b]
**1i**	$[\alpha]_D^{25}$ = −6.7 (c = 0.30, $CHCl_3$, *ee* = 99%)[2] Source of chirality: Stereospecific reaction	-	$[\alpha]_D^{25}$ = −9.7 (c = 1.79, $CHCl_3$, *ee* = 83%)	(*R*)
**1j**	$[\alpha]_D^{24}$ = −58.4 (c = 0.47, $CHCl_3$, *ee* = 97%)[2] Source of chirality: Stereospecific reaction	-	$[\alpha]_D^{25}$ = +47.8 (c = 0.49, $CHCl_3$, *ee* = 87%)	(*S*)
**1k**	-	$[\alpha]_D^{31}$ = −21.6 (c = 0.23, $CHCl_3$, *ee* = 95%)[2] Source of chirality: Stereospecific reaction	$[\alpha]_D^{25}$ = +11.4 (c = 0.70, $CHCl_3$, *ee* = 99%)	(*R*)
**1l**	$[\alpha]_D^{25}$ = +20.5 (c = 0.58, $CHCl_3$, *ee* = 95%)[2] Source of chirality: Stereospecific reaction	$[\alpha]_D^{31}$ = −22.0 (c = 0.60, $CHCl_3$, *ee* = 99%)[2] Source of chirality: Stereospecific reaction	$[\alpha]_D^{25}$ = −11.2 (c = 0.51, $CHCl_3$, *ee* = 45%)	(*S*)
**1m**	$[\alpha]_D^{25}$ = +33.2 (c = 1.29, $CHCl_3$, *ee* = 99%)[4] Source of chirality: Optical resolution and stereospecific reaction	$[\alpha]_D^{31}$ = −29.2 (c = 0.38, $CHCl_3$, *ee* = 97%)[2] Source of chirality: Stereospecific reaction	$[\alpha]_D^{25}$ = +34.4 (c = 1.32, $CHCl_3$, *ee* = 98%)	(*R*)
**1n**	-	known compound, but [α] was not measured[3] Source of chirality: Stereospecific reaction	$[\alpha]_D^{25}$ = −25.3 (c = 1.06, $CHCl_3$, *ee* = 92%)	(*S*)[b]

[a]The absolute configuration of (*S*)-**1a** was also confirmed by single crystal XRD.

[b]Absolute configuration was assigned using chiral HPLC under the same conditions reported in the literature (see below).

3. Supplementary information for X-Ray measurements

Table S6 Summary of crystallographic data, data collections, structure determination and refinement for (*S*)-**1a**·(spiro-TADDOL) and (S_P,R_C)-**3g**.

	(S_P,R_C)-**3g**	(*S*)-**1a**·(spiro-TADDOL)
CCDC number	2081817	2081818
Empirical formula	$C_{20}H_{19}O_2P$	$C_{34}H_{34}O_4$, $C_{13}H_{13}OP$
Formula weight	322.32	722.81
Temperature	294(2)	294(2)
Radiation and wavelength	Mo-Kα, λ =0.71075Å	Mo-Kα, λ =0.71075Å
Crystal system	orthorhombic	monoclinic
Space group	$P\ 2_1\ 2_1\ 2_1$	$P\ 2_1$
Unit cell dimensions	*a* =8.1680(7)Å<br>*b* =9.9573(9)Å<br>*c* =20.1721(17)Å<br>α =90°<br>β =90°<br>γ =90°	*a* =10.553(3)Å<br>*b* =9.719(3)Å<br>*c* =18.977(6)Å<br>α =90°<br>β =93.918(7)°<br>γ =90°
Volume	1641(1)Å^3	1942(1)Å^3
Z,Z'	4, 1	2, 1
Density (calculated)	1.305 Mg/m^3	1.236 Mg/m^3
Absorption coefficient, μ	0.175 mm^{-1}	0.118 mm^{-1}
F(000)	680	768
Crystal colour	colourless	colourless
Crystal description	platelet	needle
Crystal size	0.30 x 0.10 x 0.10 mm	0.50 x 0.04 x 0.04 mm
Absorption correction	numerical	numerical
Max. and min. transmission	0.993616 and 0.998669	0.965396 and 0.995849
θ–range for data collection	3.209 ≤ θ ≤ 25.350°	2.991 ≤ θ ≤ 22.463°
Index ranges	-9 ≤ *h* ≤9;<br>-11≤ *k* ≤11;<br>-24 ≤ *l* ≤24	-11 ≤ *h* ≤11;<br>-10≤ *k* ≤10;<br>-20 ≤ *l* ≤20
Reflections collected	32784	20355
Completeness to 2θ	0.998	0.997
Absolute structure parameter	0.05(4)	0.0(3)
Friedel coverage	0.725	0.867
Friedel fraction max.	1.000	0.999
Friedel fraction full	1.000	0.999
Independent reflections	2996 [*R*(int) =0.0807]	5047 [*R*(int) =0.2604]
Reflections *I*>2σ(*I*)	2614	2392
Data / restraints / parameters	2996 /0 /210	5047 /1 /481
Goodness-of-fit on *F*2	1.122	0.960
Final *R* indices [*I*>2σ(*I*)]	R_1 =0.0500,<br>wR^2 =0.0854	R_1 =0.0889,<br>wR^2 =0.1329
R indices (all data)	R_1 =0.0620,<br>wR^2 =0.0893	R_1 =0.1869,<br>wR^2 =0.1668
Max. and mean shift/esd	0.000;0.000	0.001;0.000
Largest diff. peak and hole	0.21;-0.20 e.Å^{-3}	0.22;-0.20 e.Å^{-3}

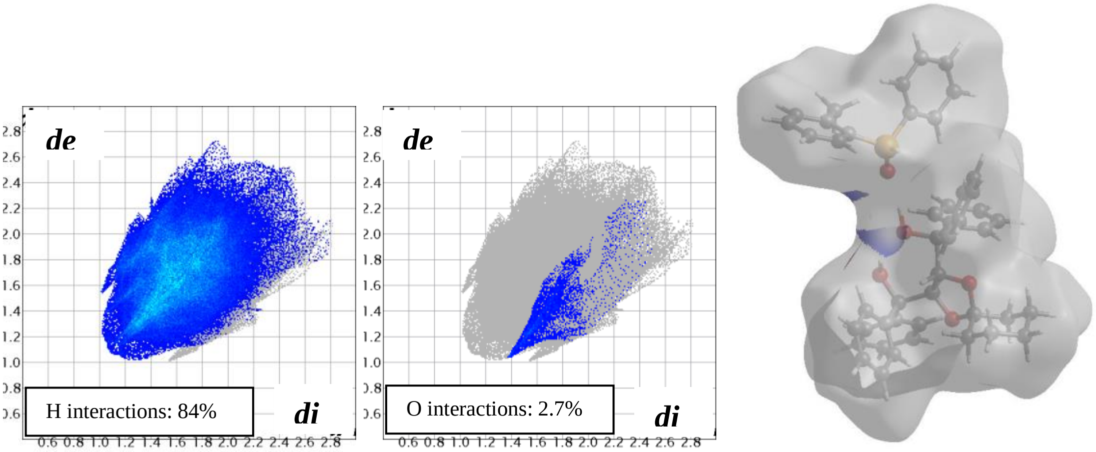

a. **b.** **c.**

Figure S2 a.) H atoms are responsible for the 84% of the intermolecular interactions in the deastereomer (by Hirshfeld surface analysis), b.) O atoms are responsible for the 2.7%. of the intermolecular interactions in the diastereomer (by Hirshfeld surface analysis) c.) The connection surface for O atoms presented by blue colours. (*di* is the distance of H atoms to the Hirshfeld surface, while *de* is the distance from Hirshfeld surface to the neighbouring atom).

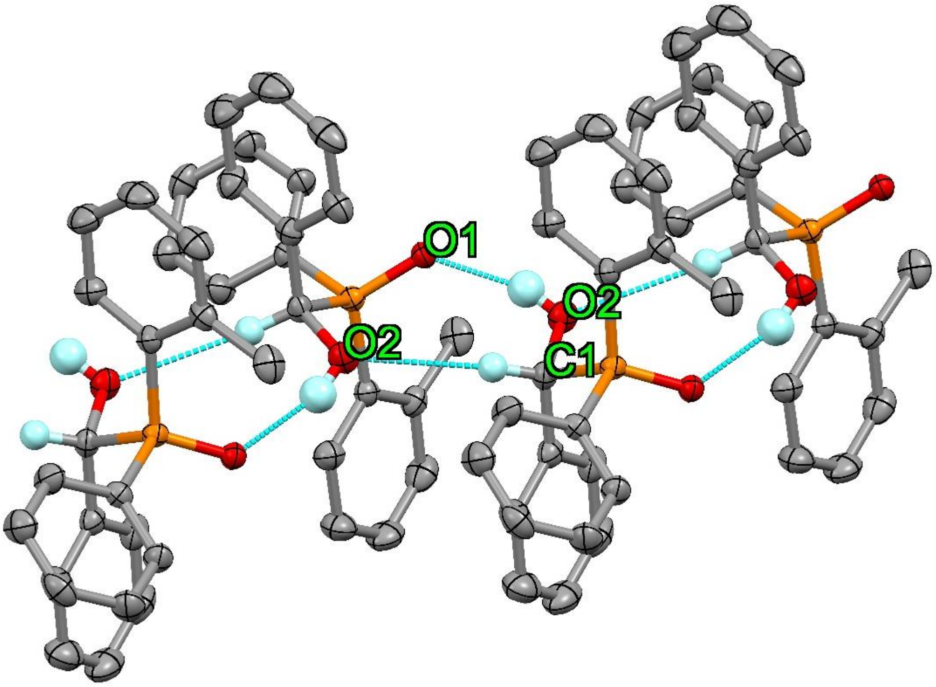

Figure S3 The hydrogen bridge motifs in the crystal structure of (S_P,R_C)-**3g**. Hydrogen atoms are omitted except the hydrogens involved in the hydrogen bridge. The atomic ellipsoids are drawn on 30% probability level.

**Direction**	***a* axis**	***b* axis**	***c* axis**
(*S*$_P$,*R*$_C$)-**3g**	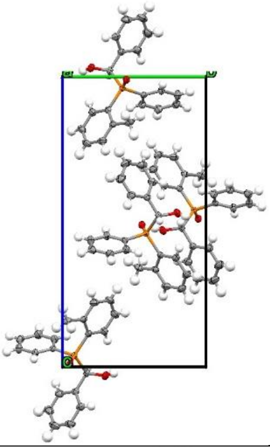	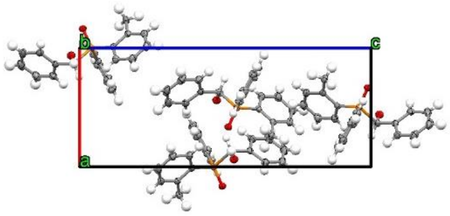	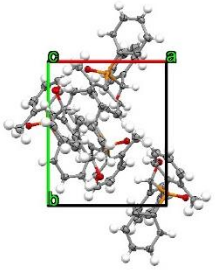
(*S*)-**1a**·(spiro-TADDOL)	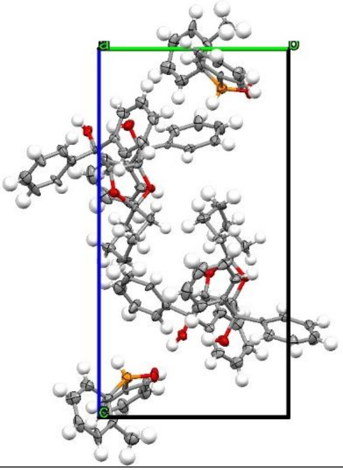	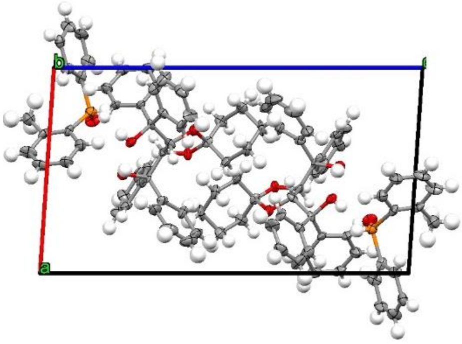	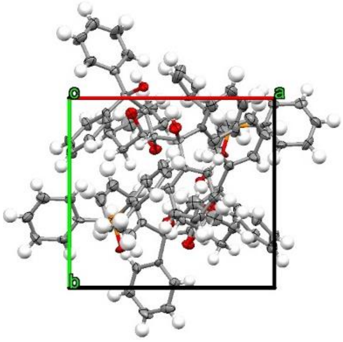

Figure S4 Comparison of the packing motifs in the different crystals of (*S*$_P$,*R*$_C$)-**3g** and (*S*)-**1a**·(spiro-TADDOL).

^{31}P, ^{19}F, ^{1}H and ^{13}C NMR spectra of the compounds prepared

(2-methylphenyl)-phenylphosphine oxide (**1a**)

$^{31}P\{^1H\}$ NMR (121.5, MHz, $CDCl_3$)

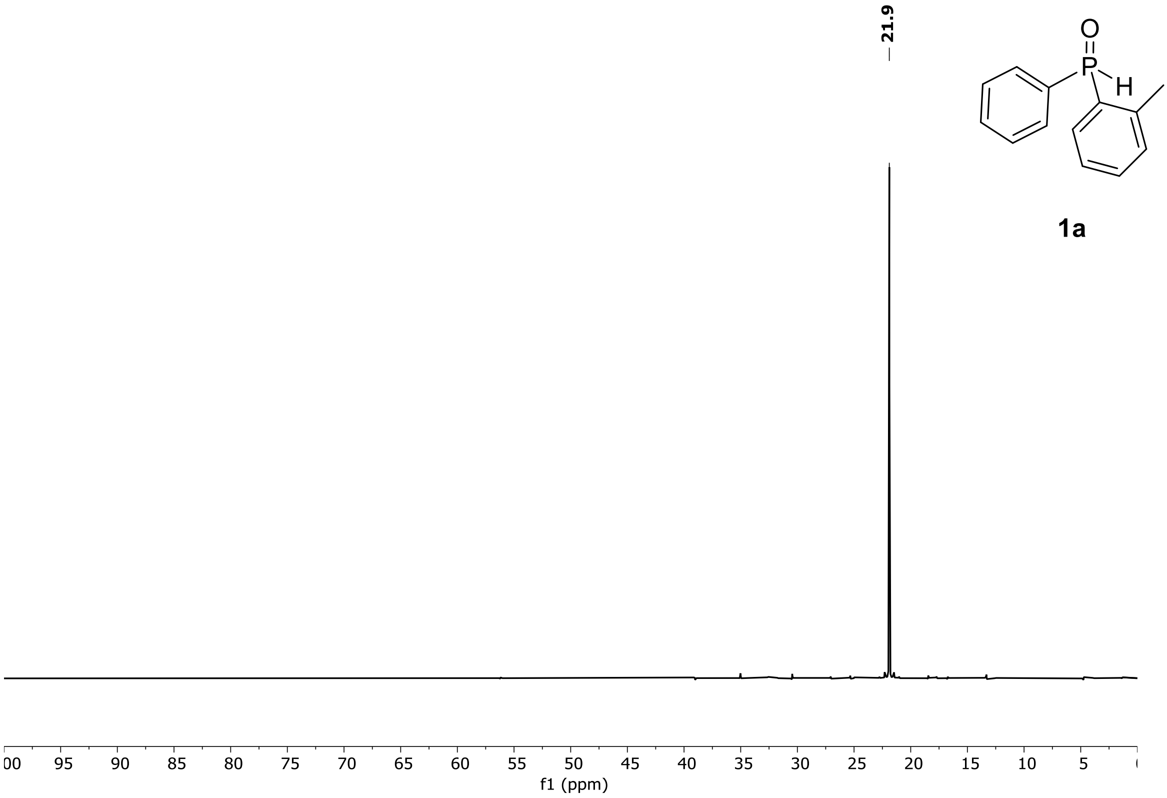

^1H NMR (500 MHz, $CDCl_3$)

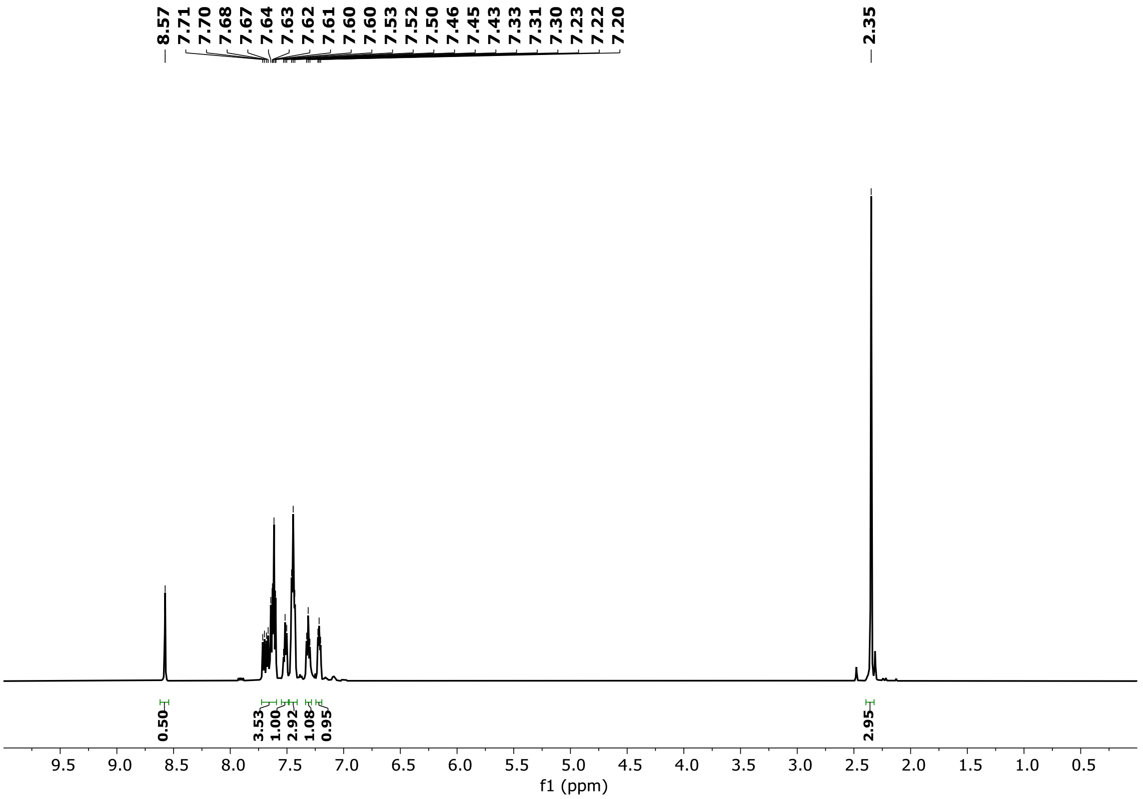

$^{13}C\{^{1}H\}$ NMR (125.8 MHz, $CDCl_3$)

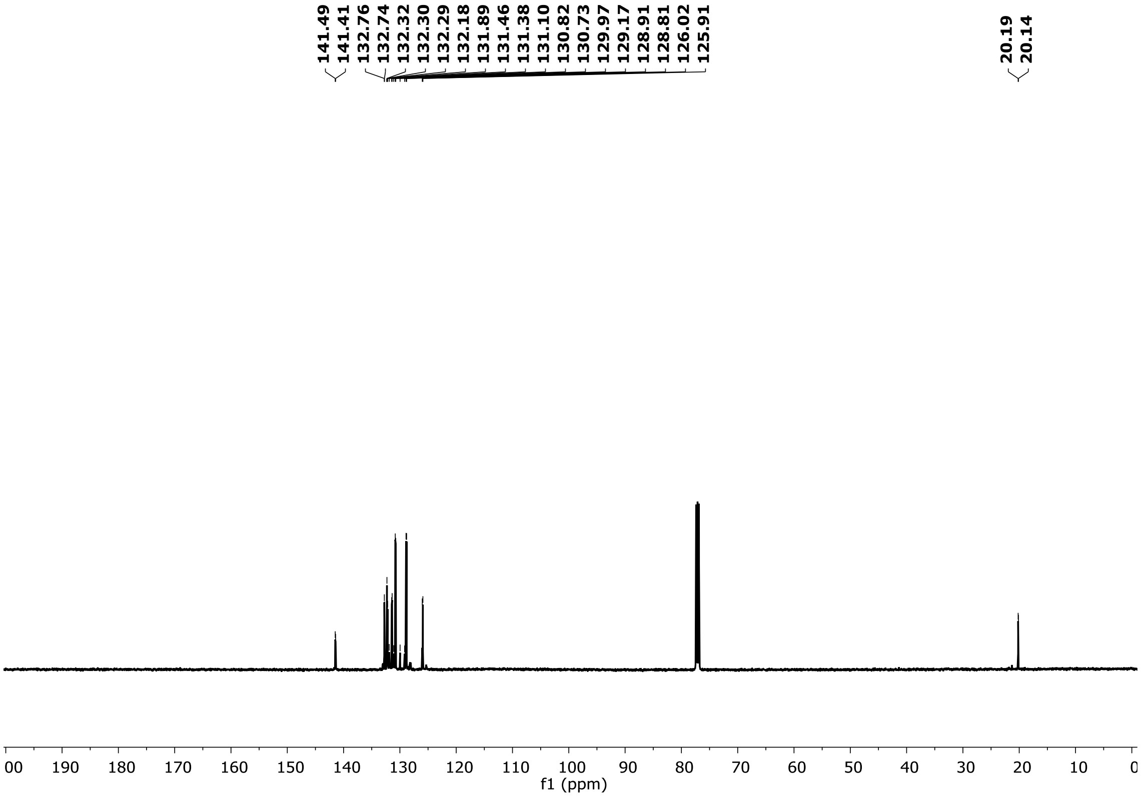

(3-methylphenyl)-phenylphosphine oxide (**1b**)

$^{31}P\{^{1}H\}$ NMR (121.5, MHz, $CDCl_3$)

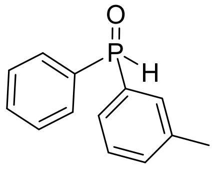

1b

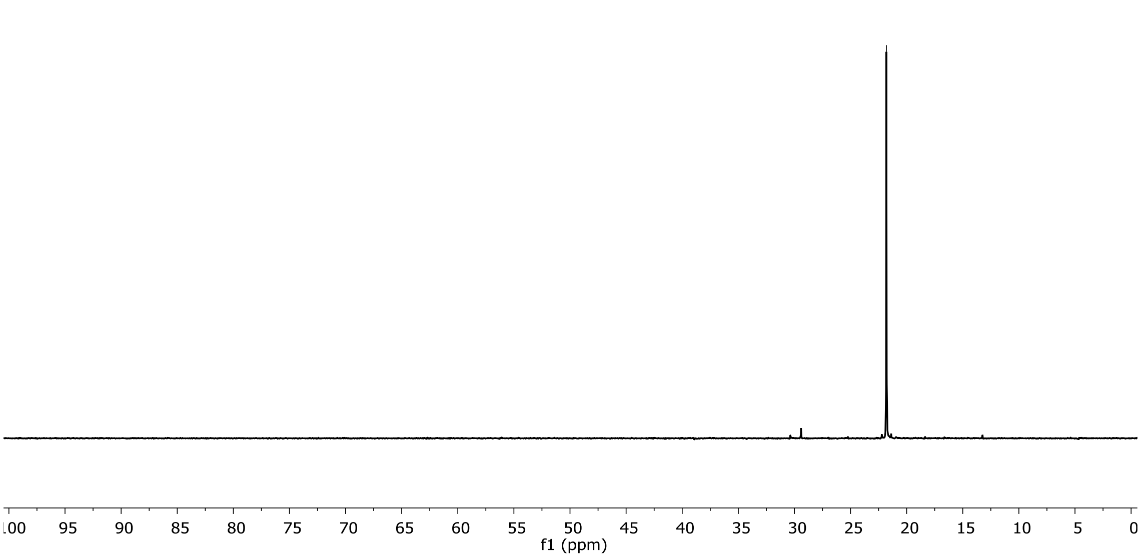

^{1}H NMR (500 MHz, $CDCl_3$)

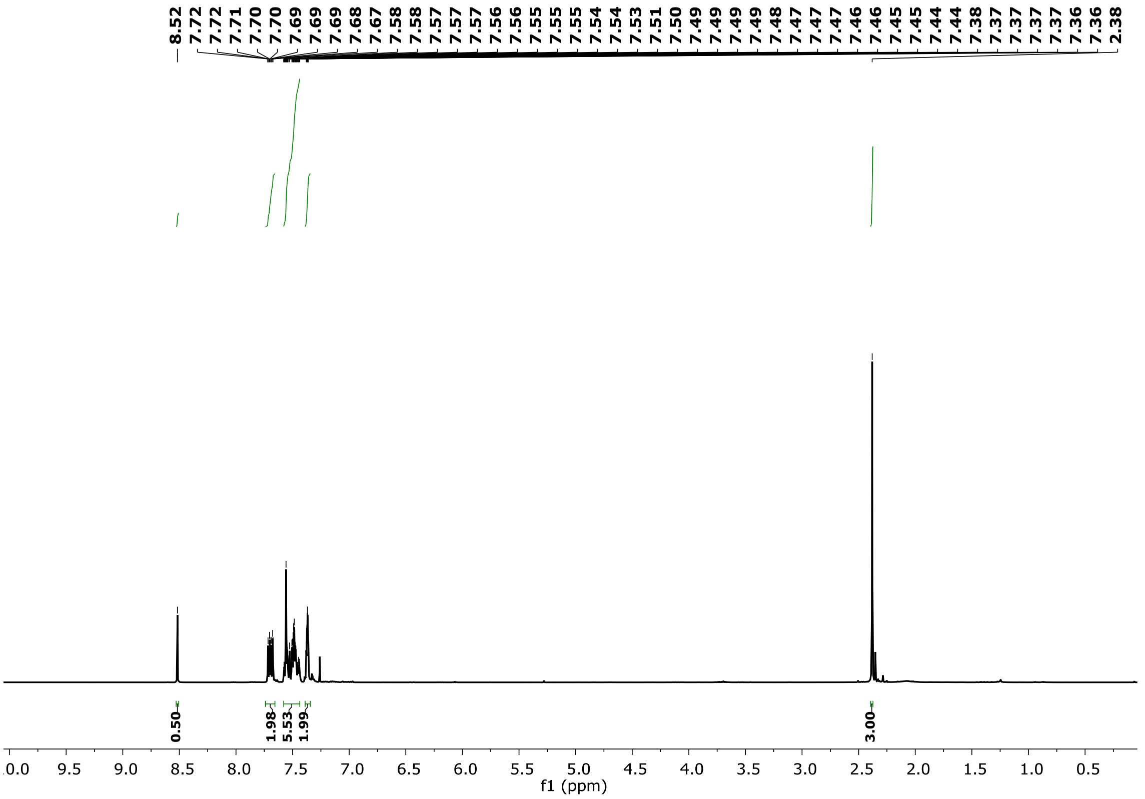

$^{13}C\{^{1}H\}$ NMR (75.5 MHz, $CDCl_3$)

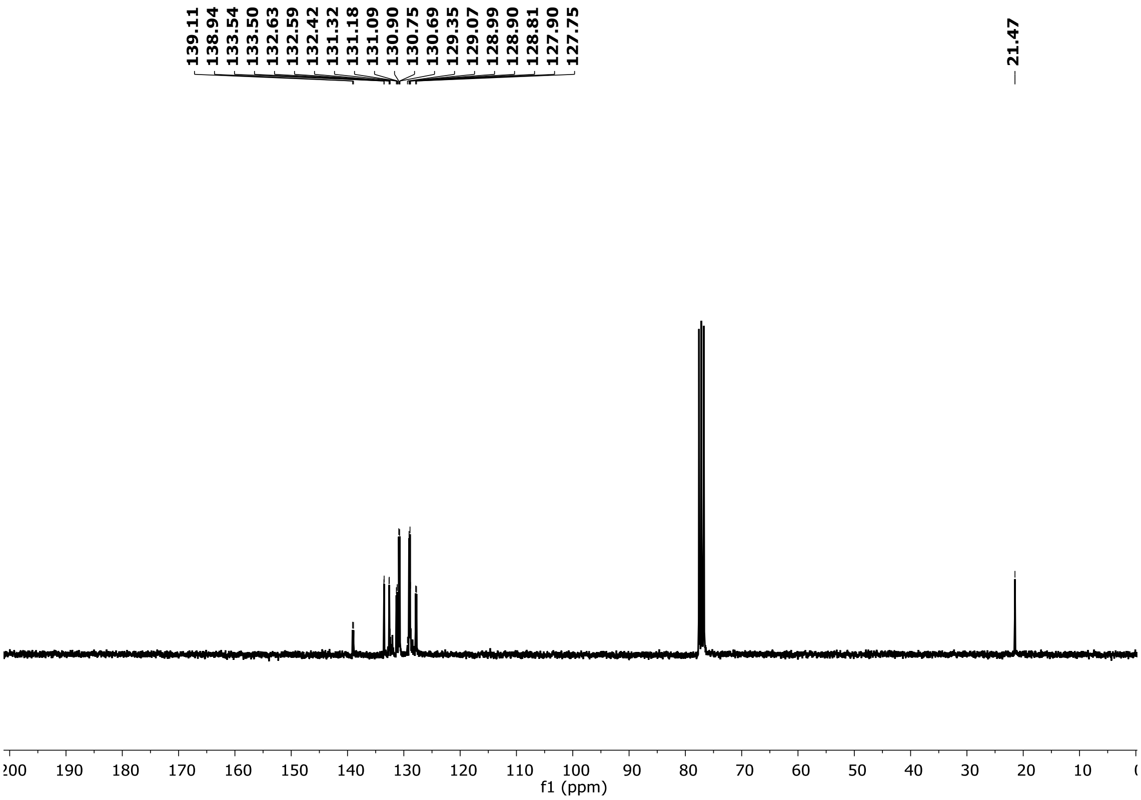

(4-methylphenyl)-phenylphosphine oxide (**1c**)

$^{31}P\{^{1}H\}$ NMR (121.5, MHz, $CDCl_3$)

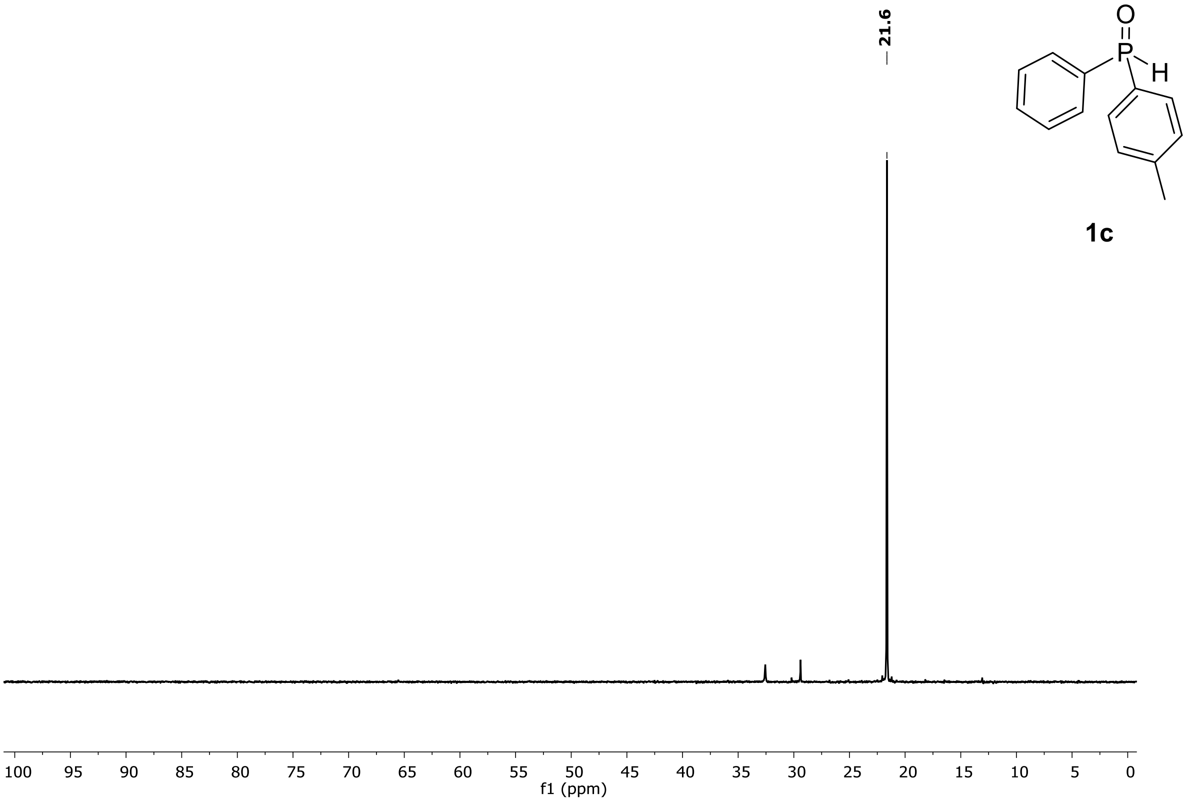

^{1}H NMR (500 MHz, $CDCl_3$)

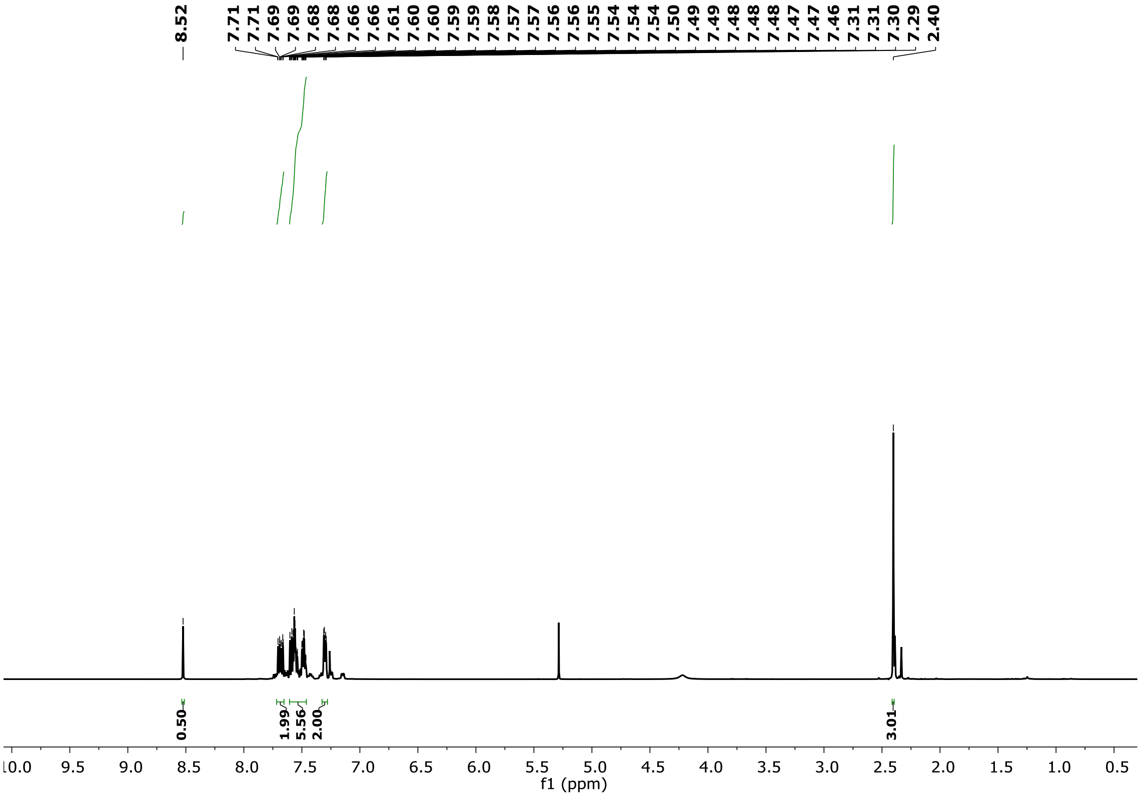

$^{13}C\{^{1}H\}$ NMR (75.5 MHz, CDCl$_3$)

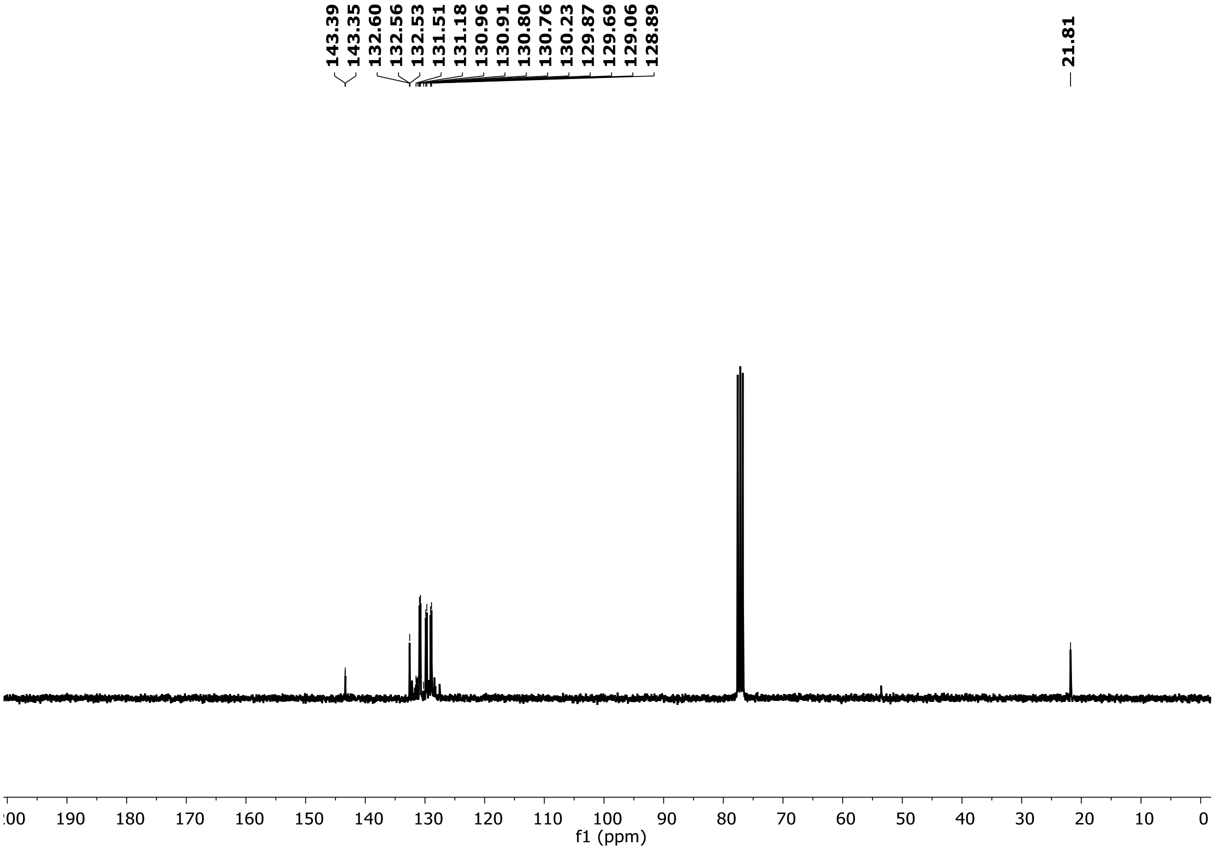

(2-trifluoromethylphenyl)-phenylphosphine oxide (**1d**)

$^{31}P\{^{1}H\}$ NMR (121.5, MHz, $CDCl_3$)

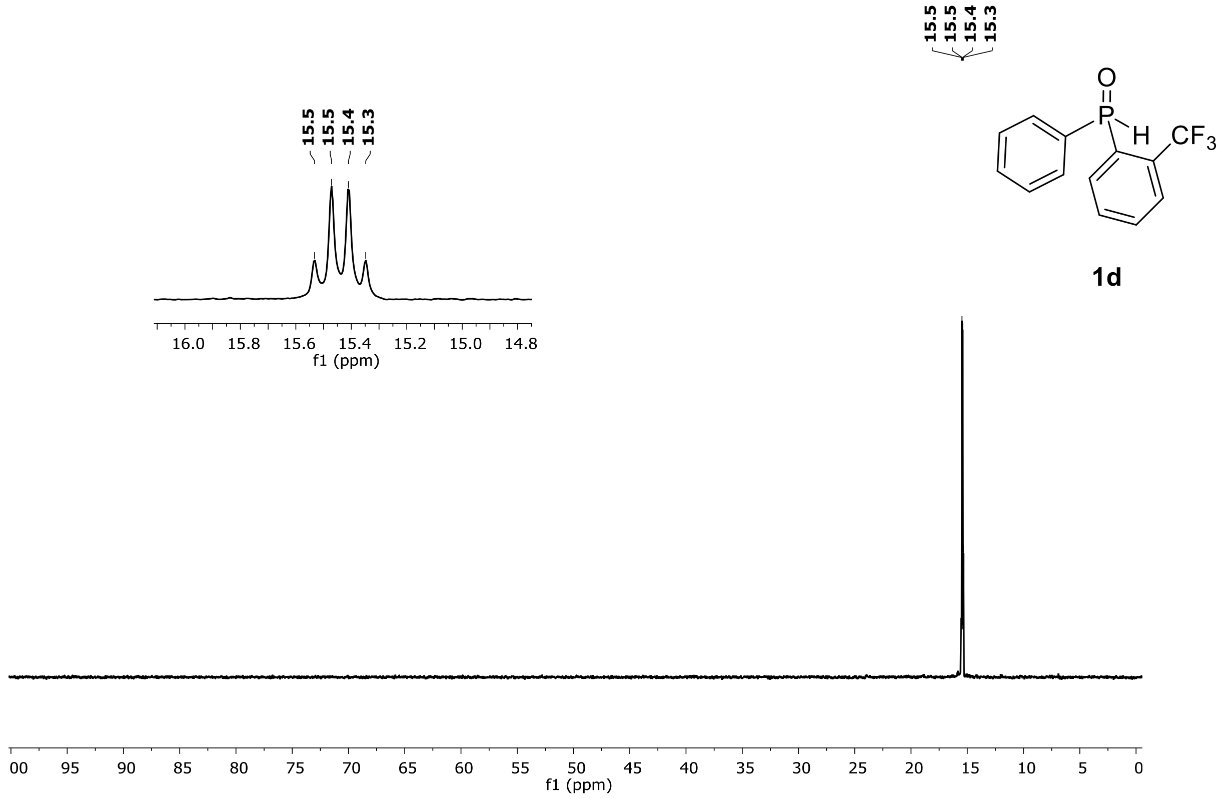

^{1}H NMR (500 MHz, $CDCl_3$)

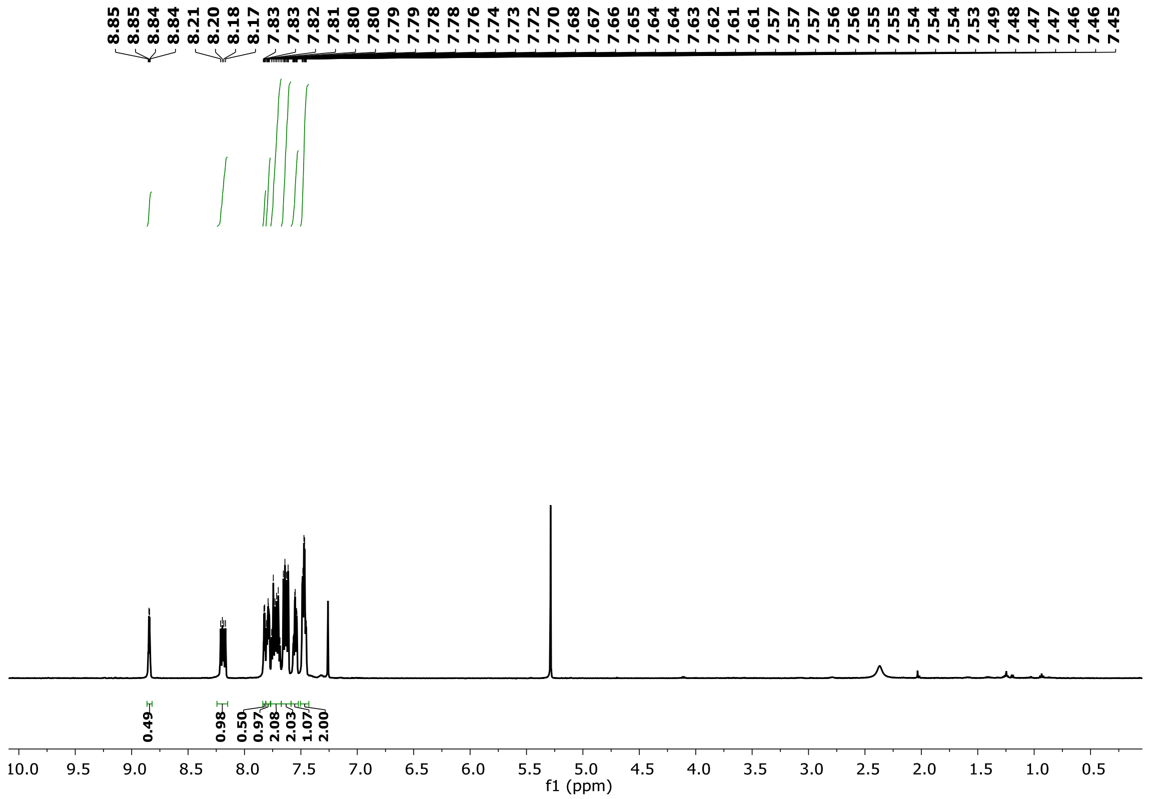

$^{13}C\{^{1}H\}$ NMR (75.5 MHz, $CDCl_3$)

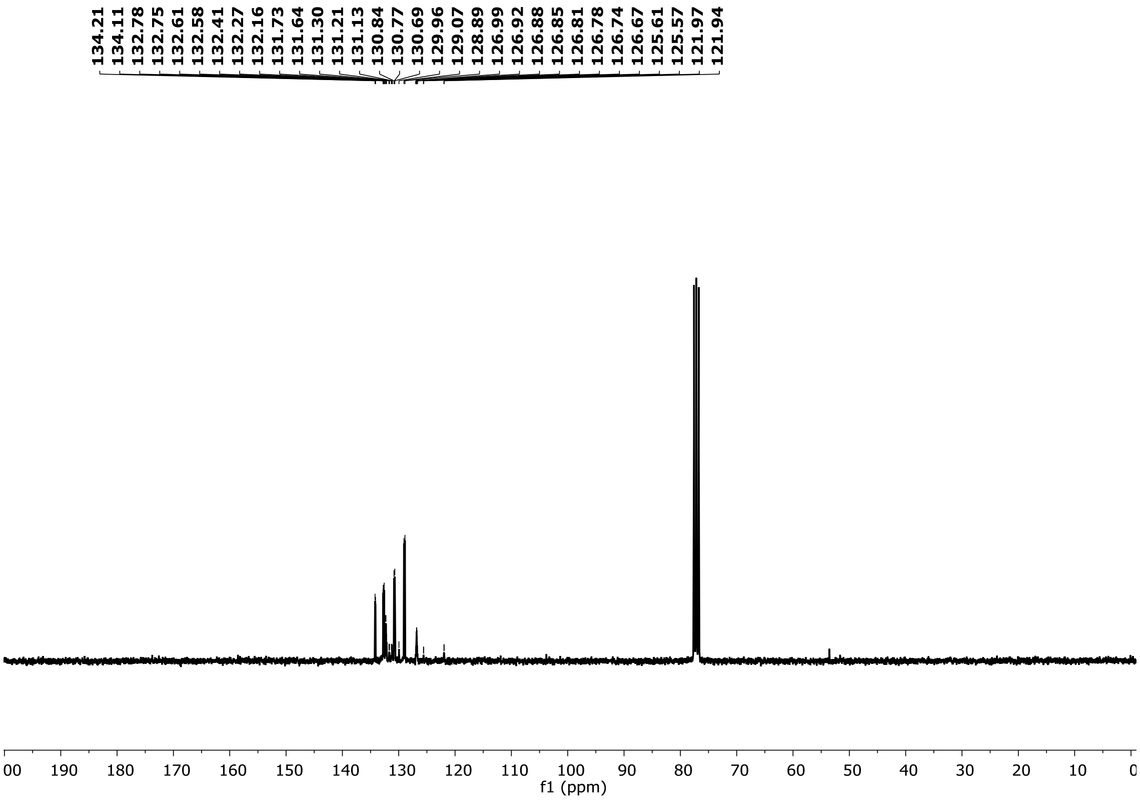

^{19}F NMR (282.4 MHz, $CDCl_3$)

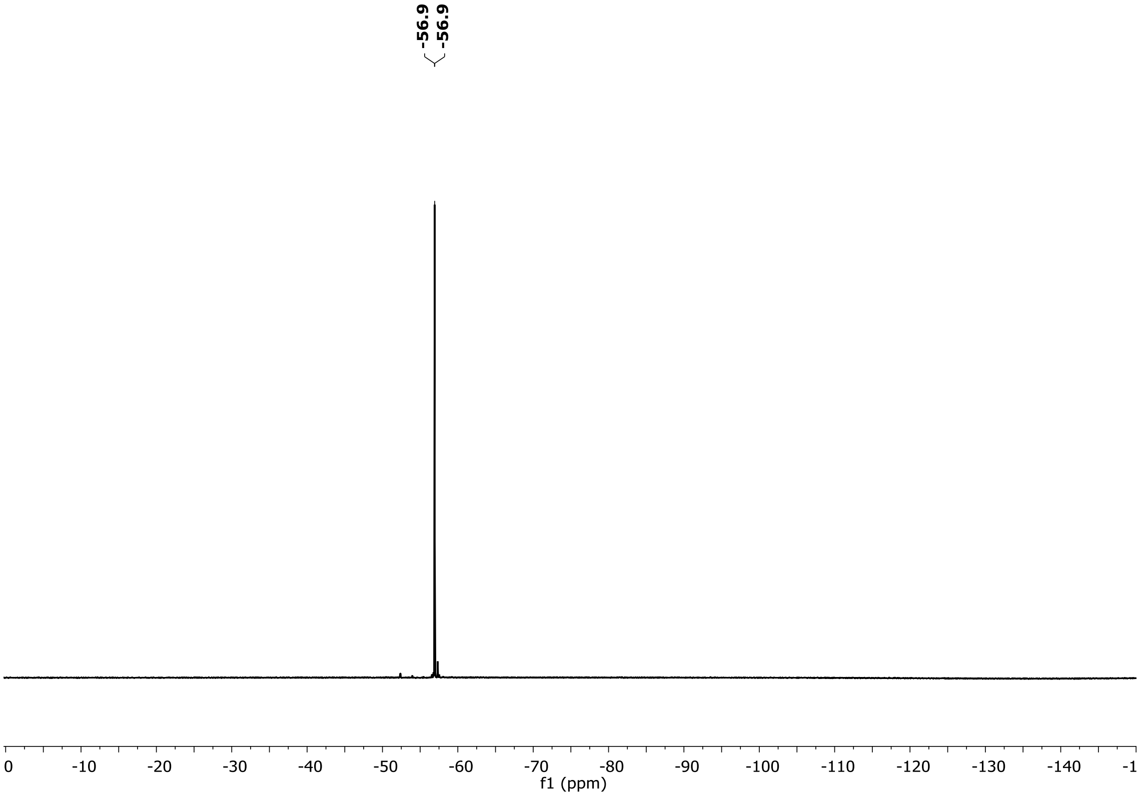

(3-trifluoromethylphenyl)-phenylphosphine oxide (**1e**)

$^{31}P\{^{1}H\}$ NMR (121.5, MHz, $CDCl_3$)

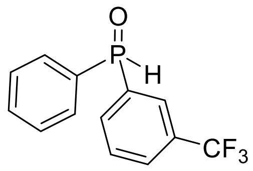

1e

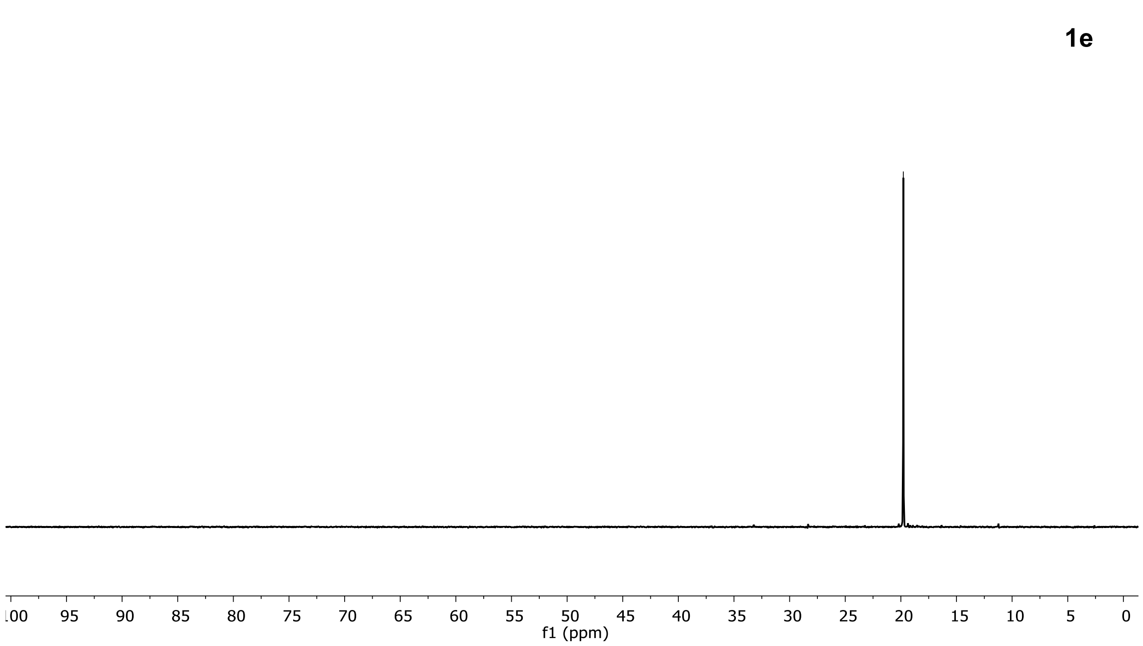

^{1}H NMR (500 MHz, $CDCl_3$)

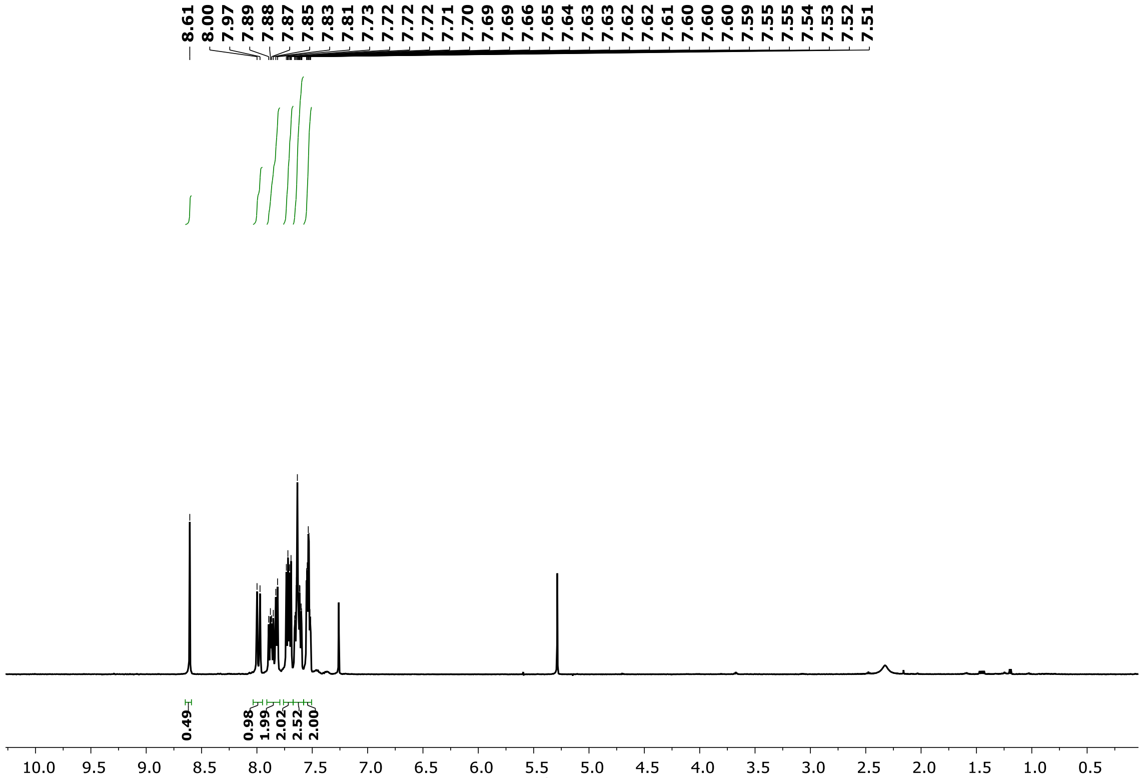

$^{13}C\{^{1}H\}$ NMR (75.5 MHz, $CDCl_3$)

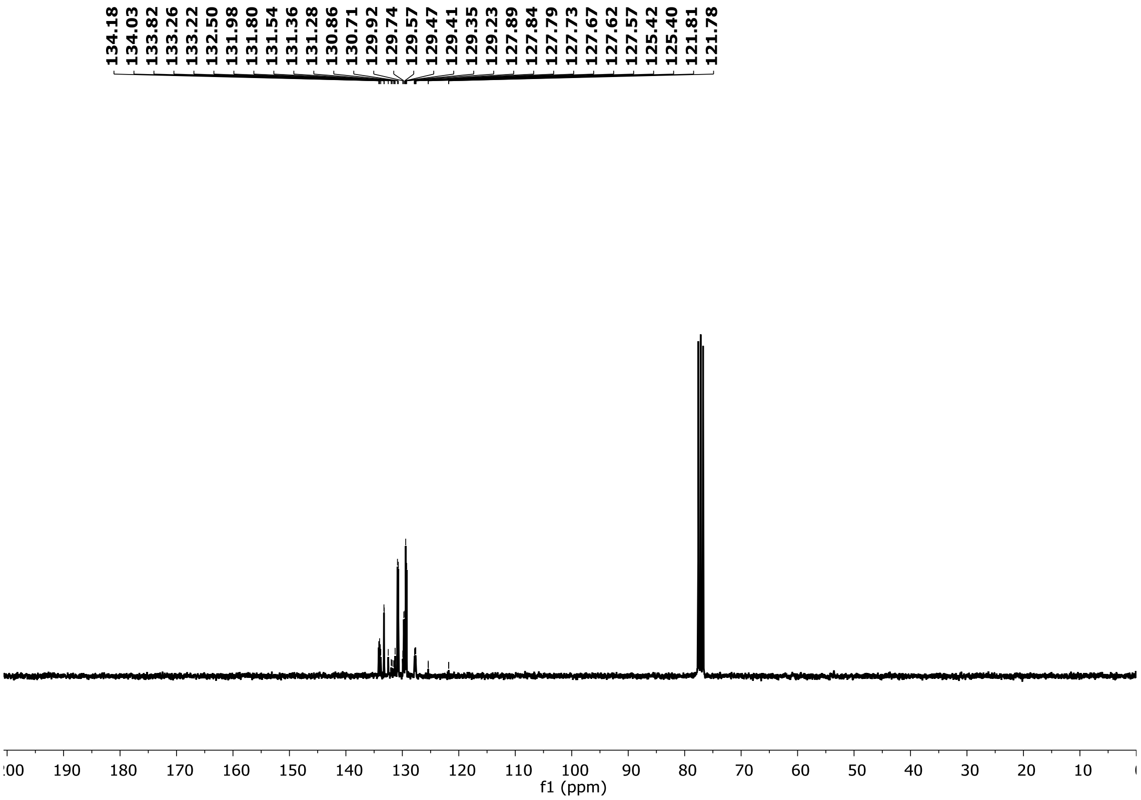

^{19}F NMR (282.4 MHz, $CDCl_3$)

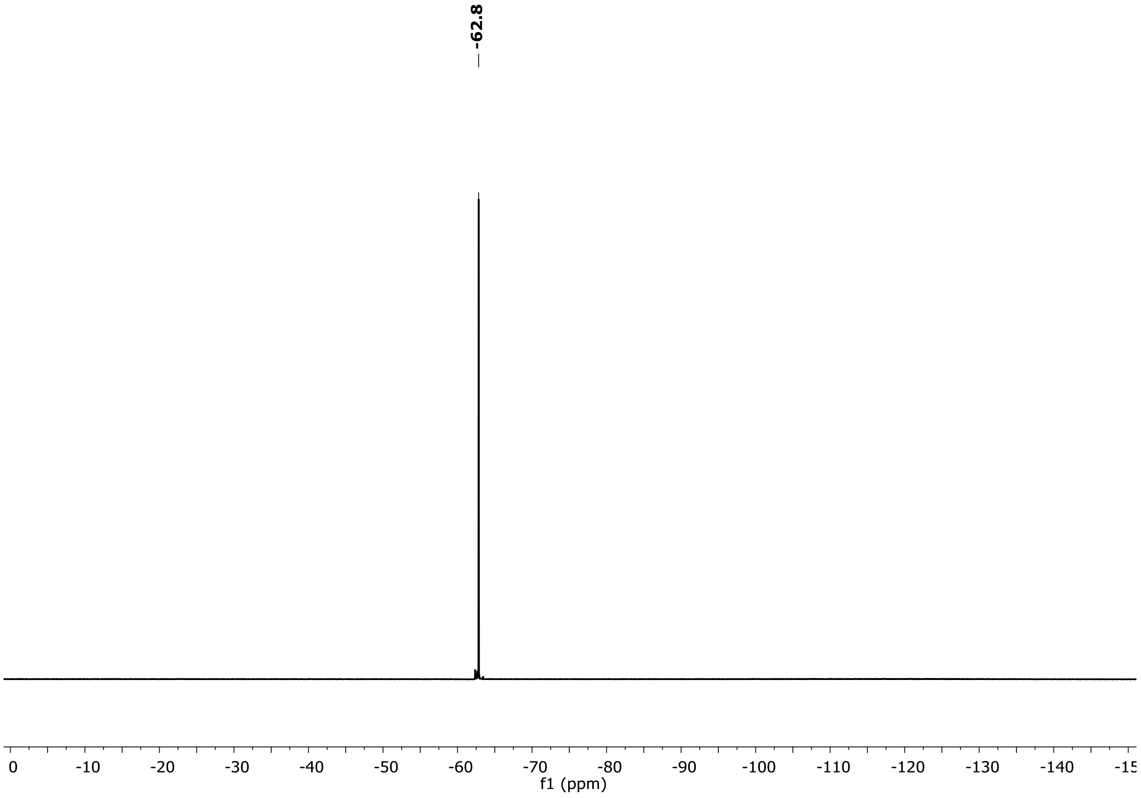

(4-trifluoromethylphenyl)-phenylphosphine oxide (**1f**)

$^{31}P\{^{1}H\}$ NMR (121.5, MHz, $CDCl_3$)

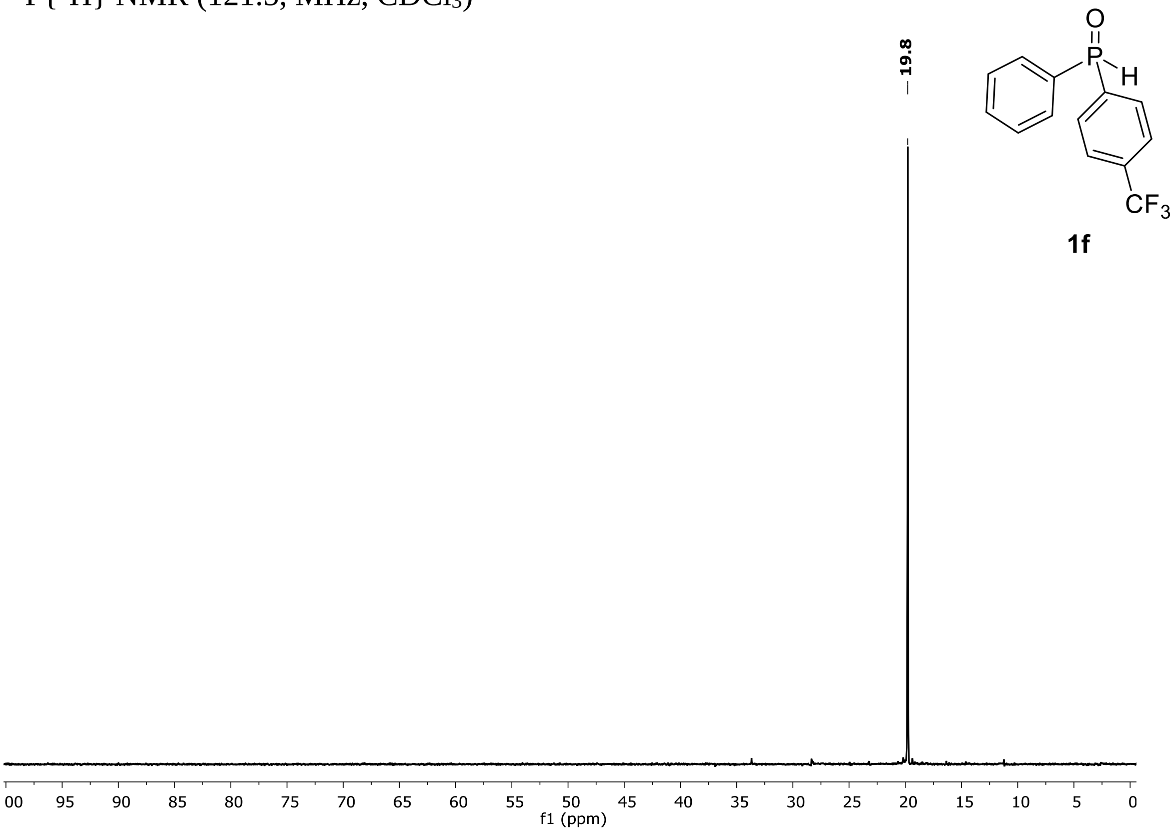

^{1}H NMR (500 MHz, $CDCl_3$)

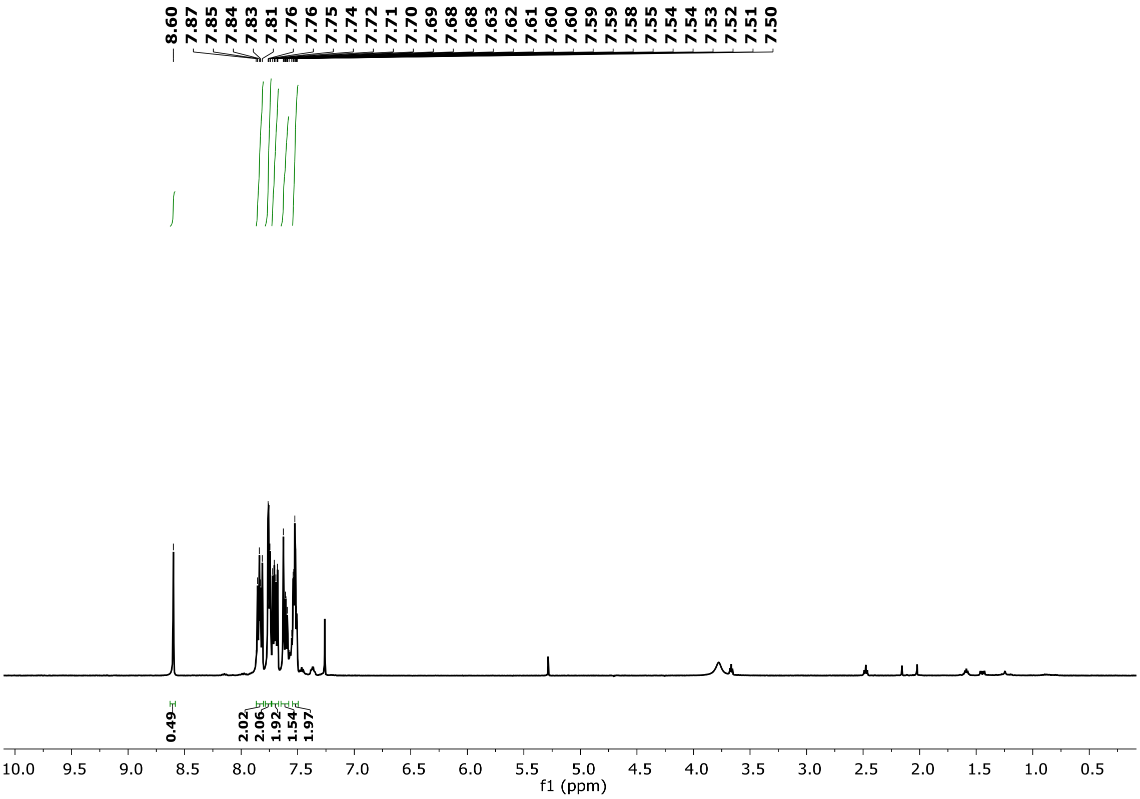

$^{13}C\{^{1}H\}$ NMR (75.5 MHz, $CDCl_3$)

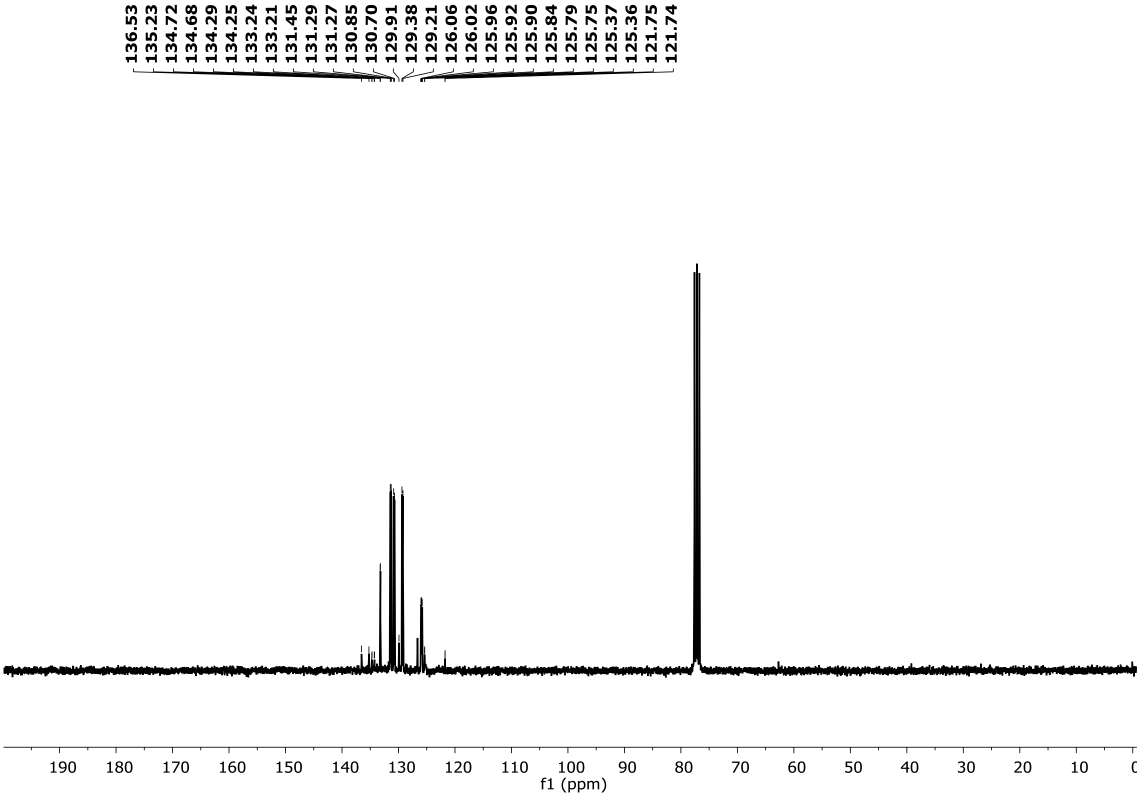

^{19}F NMR (282.4 MHz, $CDCl_3$)

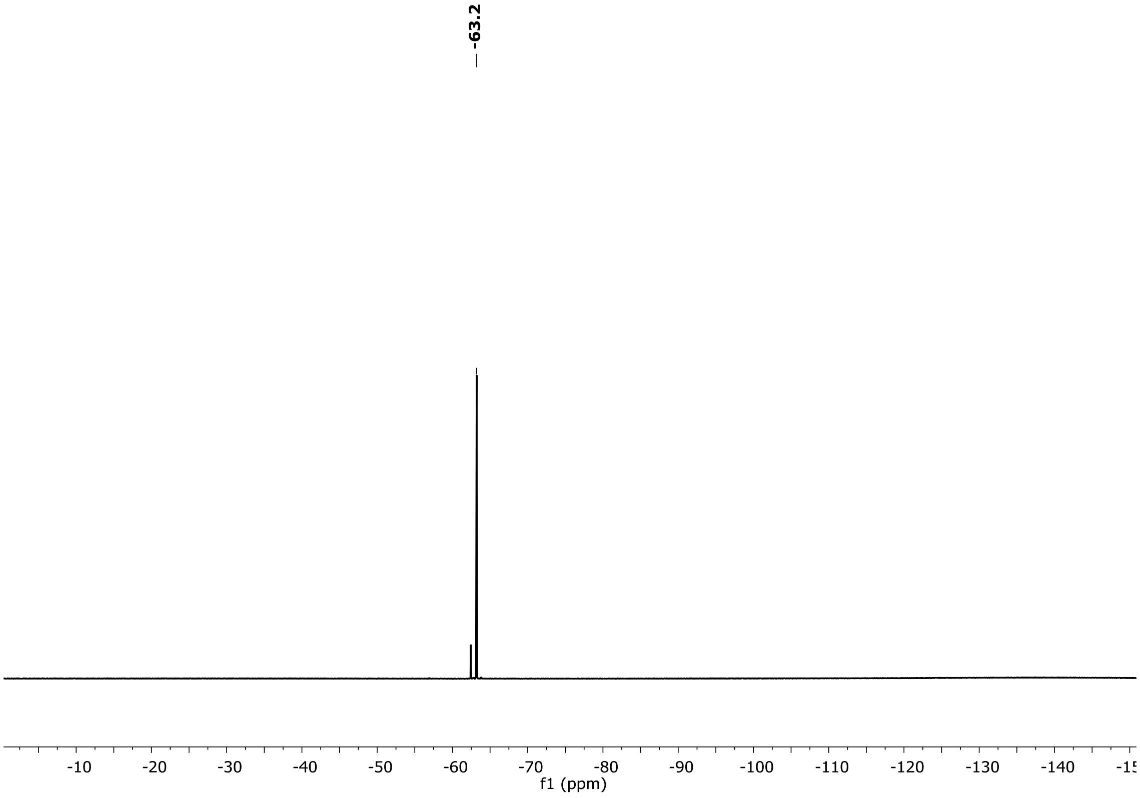

(2-methoxyphenyl)-phenylphosphine oxide (**1g**)

$^{31}P\{^{1}H\}$ NMR (121.5, MHz, $CDCl_3$)

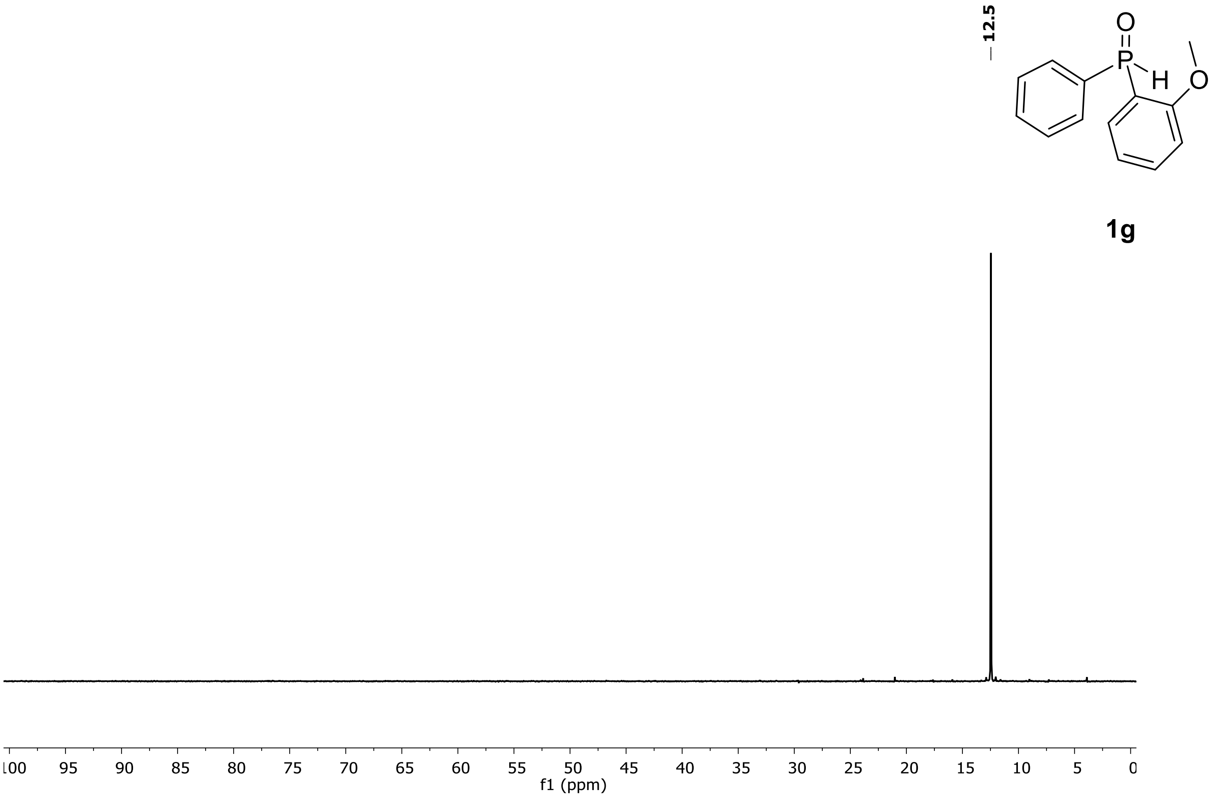

^{1}H NMR (500 MHz, $CDCl_3$)

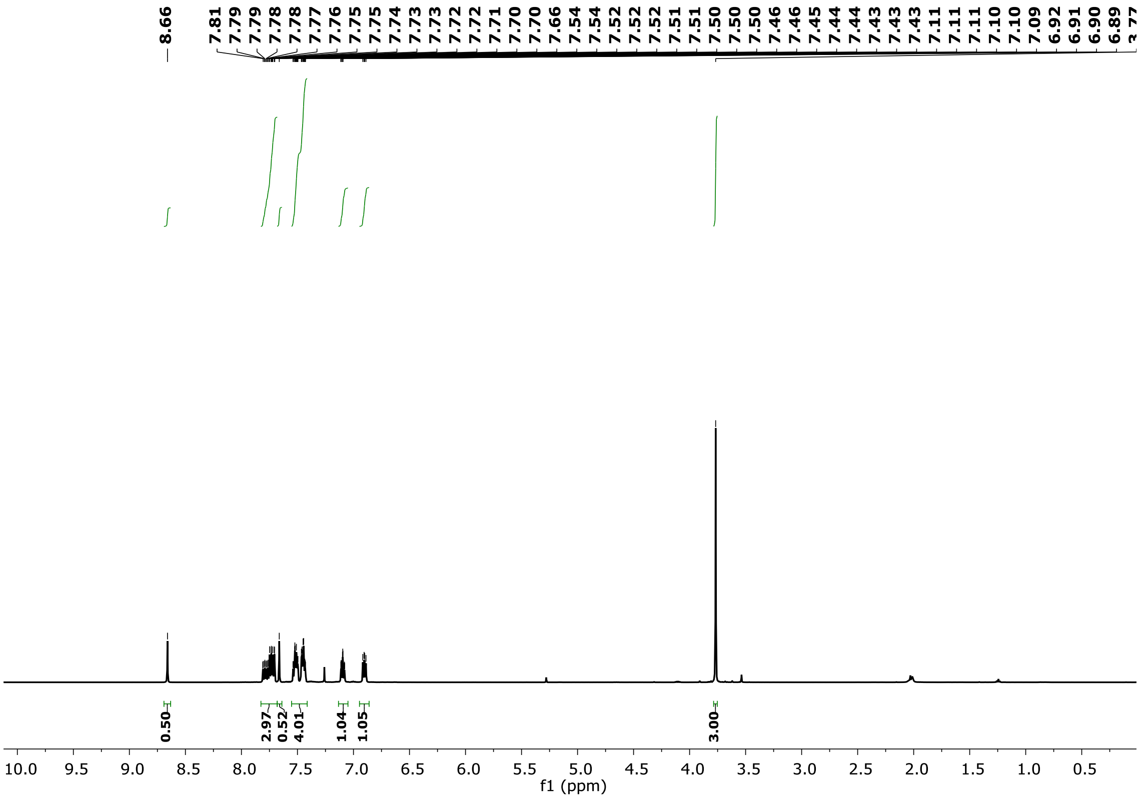

$^{13}C\{^{1}H\}$ NMR (75.5 MHz, $CDCl_3$)

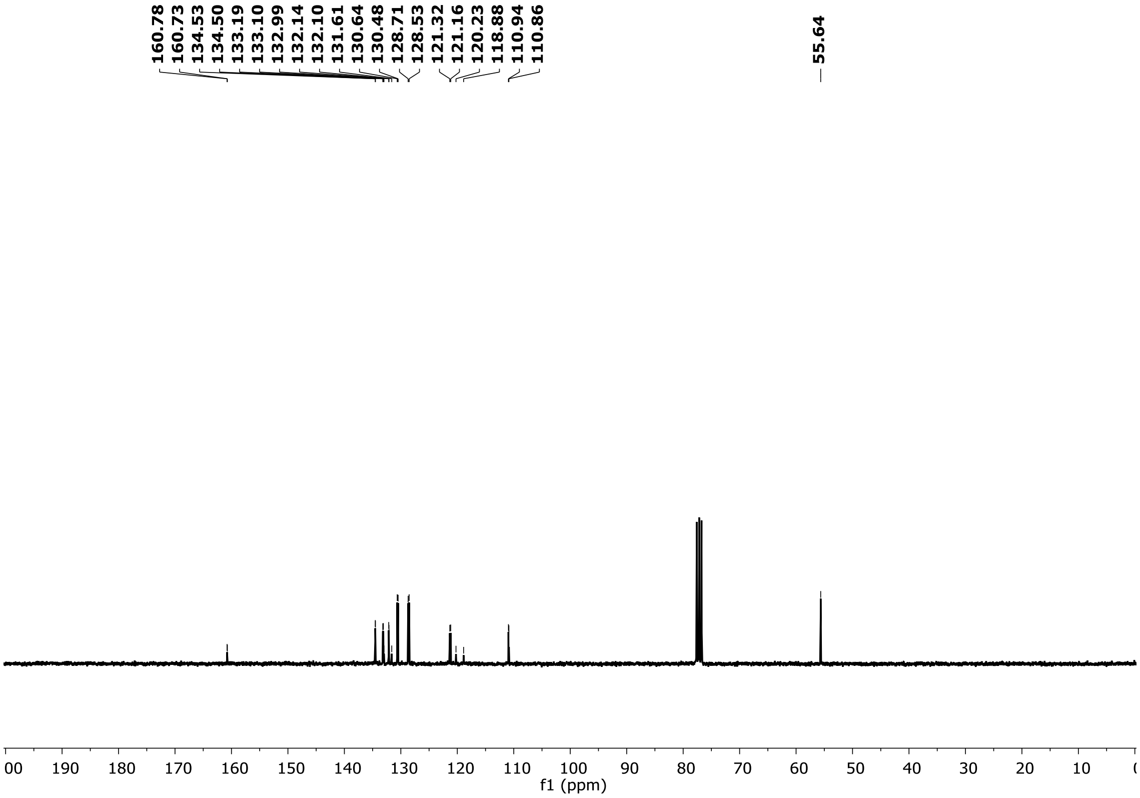

(2-phenylphenyl)-phenylphosphine oxide (**1h**)

$^{31}P\{^{1}H\}$ NMR (121.5, MHz, $CDCl_3$)

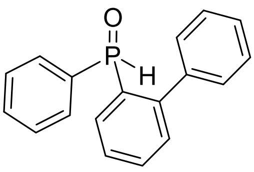

1h

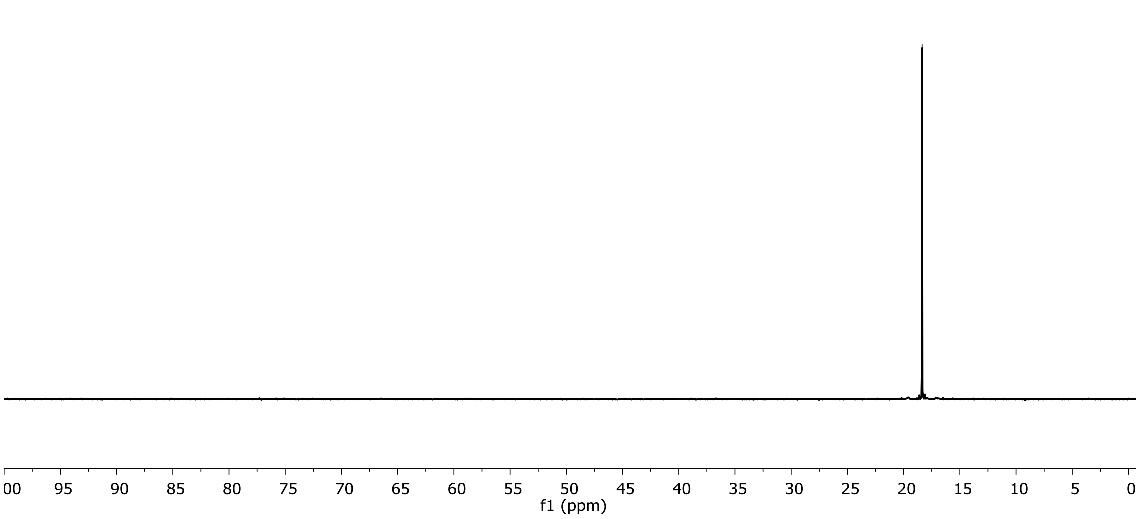

^{1}H NMR (500 MHz, $CDCl_3$)

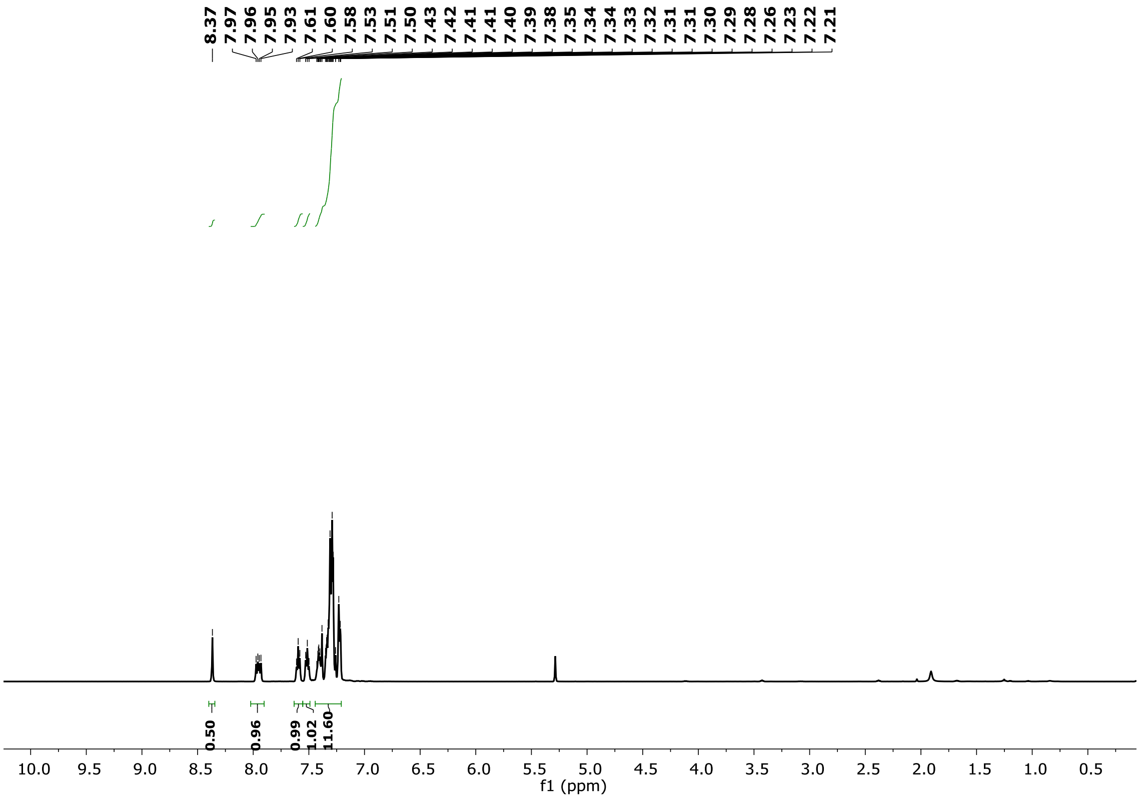

$^{13}C\{^{1}H\}$ NMR (125.8 MHz, $CDCl_3$)

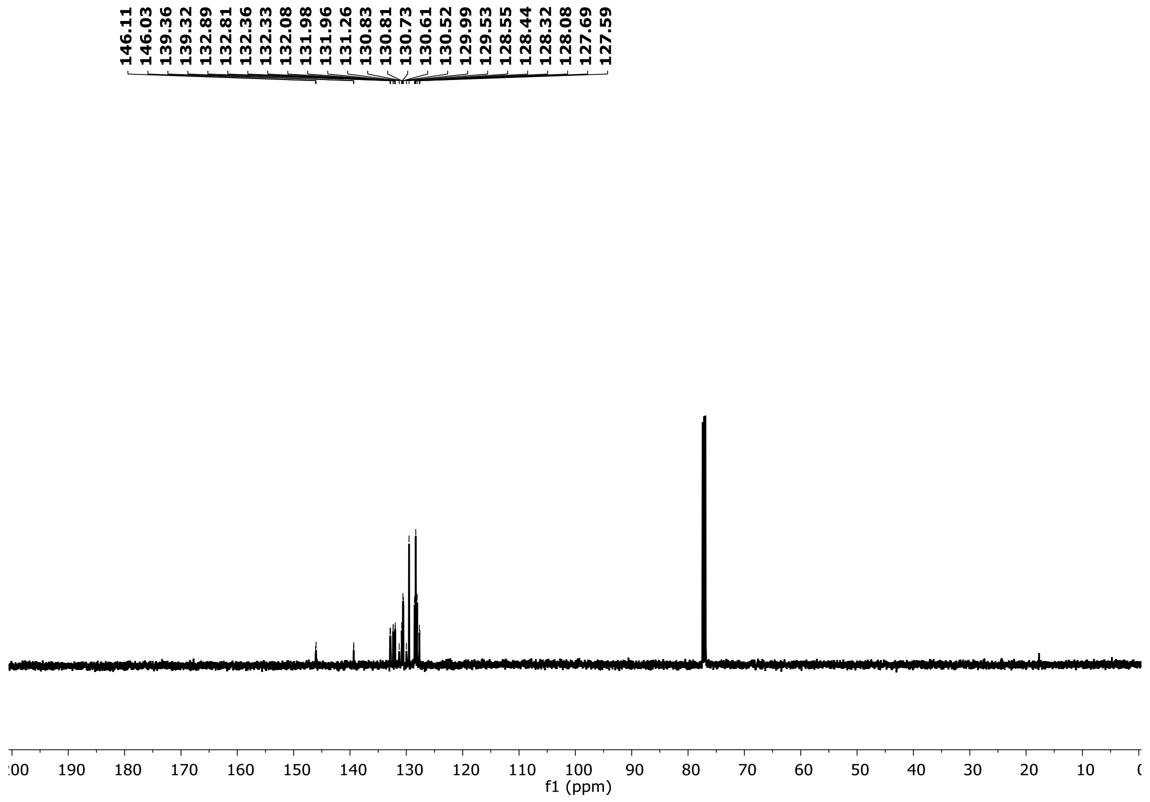

(1-napthyl)-phenylphosphine oxide (**1i**)

$^{31}P\{^{1}H\}$ NMR (121.5, MHz, $CDCl_3$)

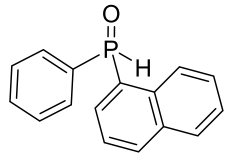

1i

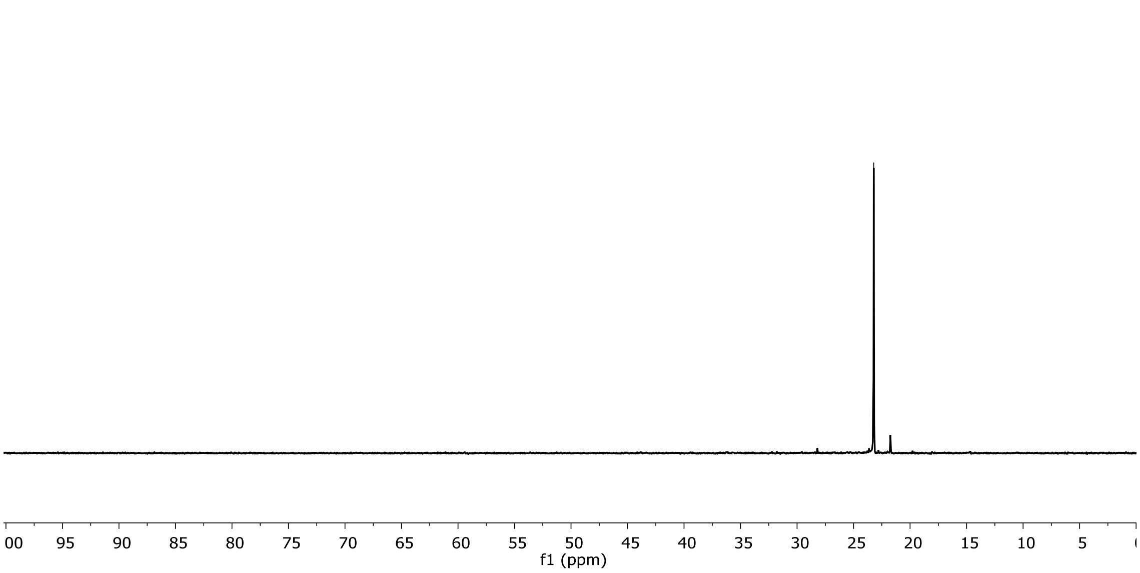

^{1}H NMR (500 MHz, $CDCl_3$)

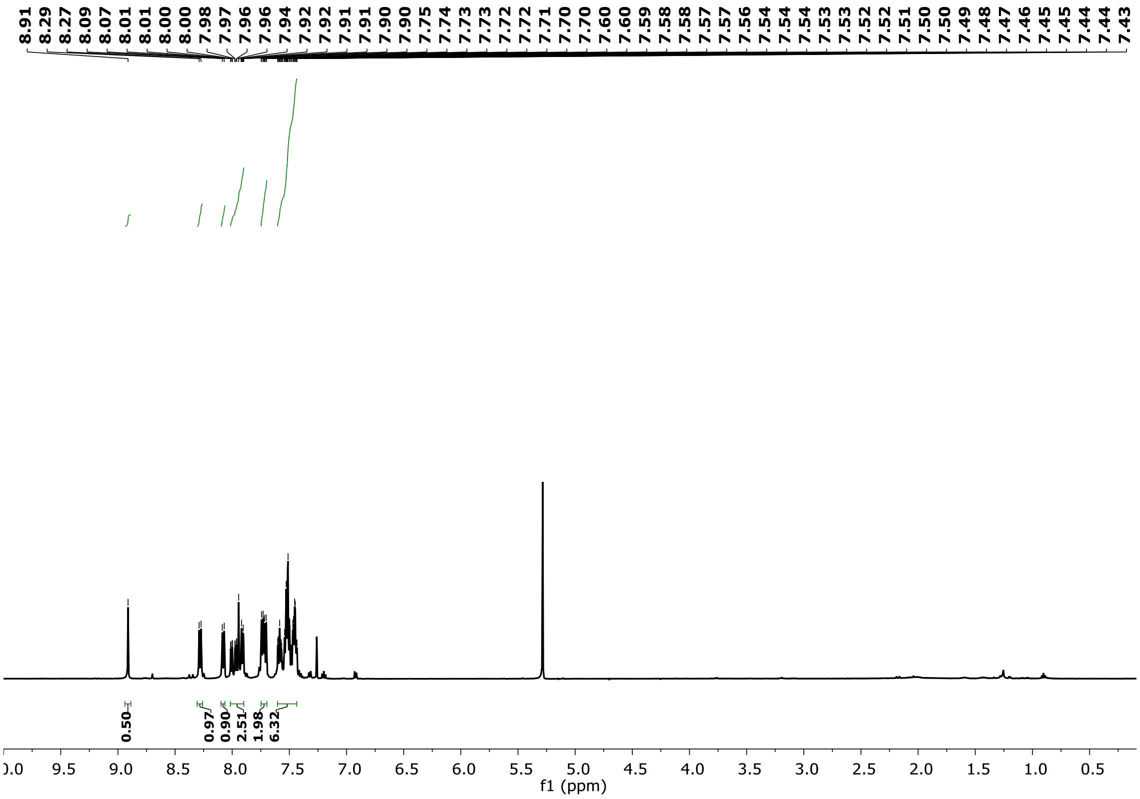

$^{13}C\{^{1}H\}$ NMR (75.5 MHz, $CDCl_3$)

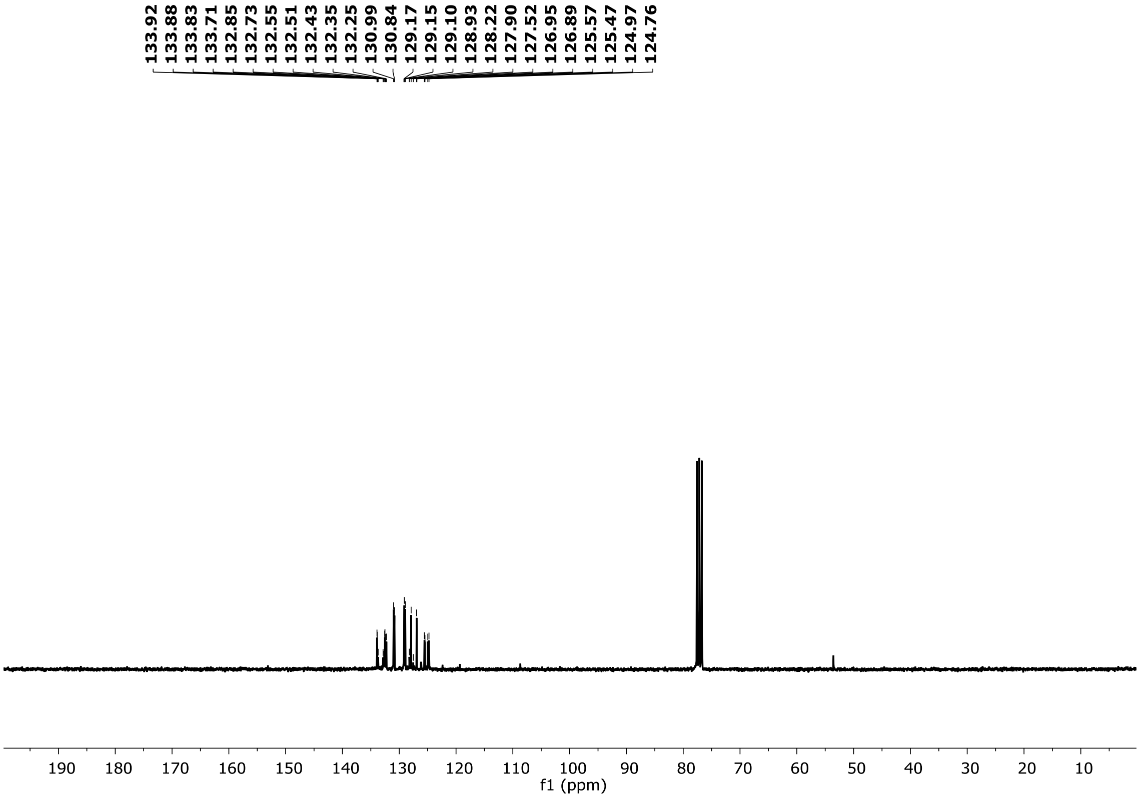

(*S*)-Benzyl-phenylphosphine oxide [(*S*)-**1j**]

$^{31}P\{^{1}H\}$ NMR (202.5 MHz, $CDCl_3$)

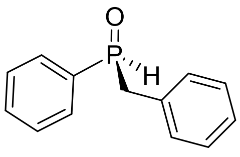

(*S*)-**1j**

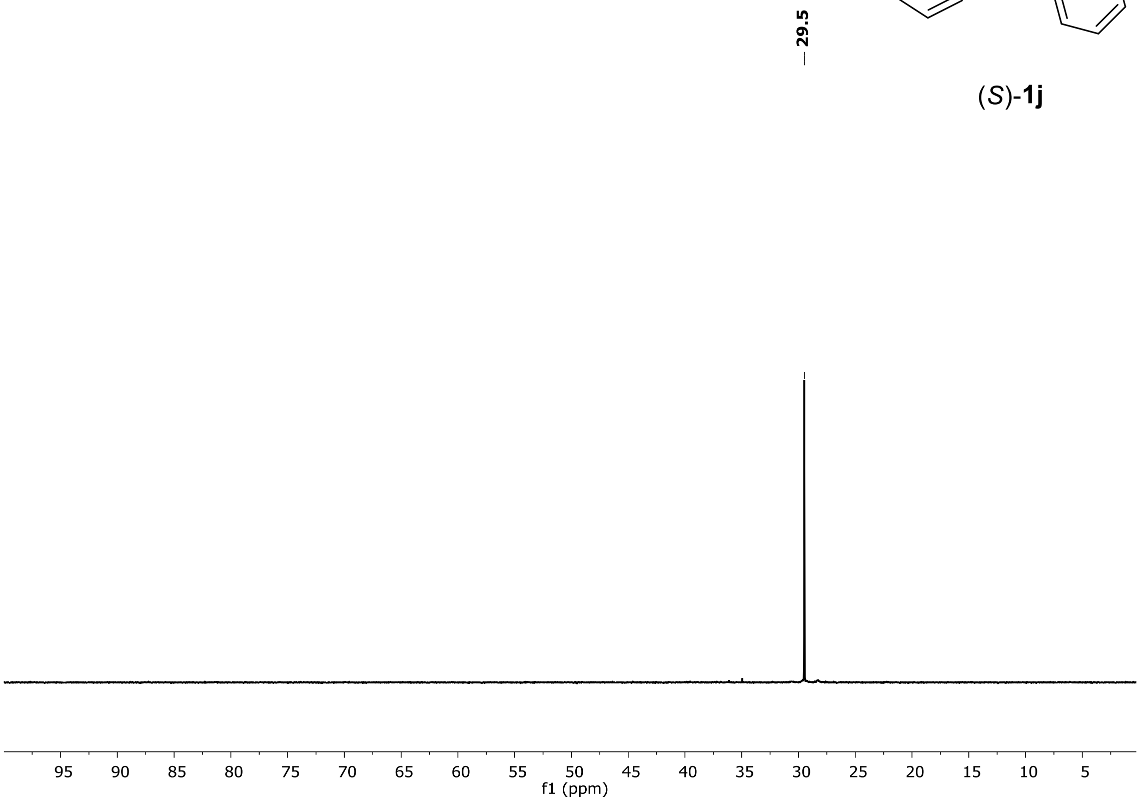

^{1}H NMR (500 MHz, $CDCl_3$)

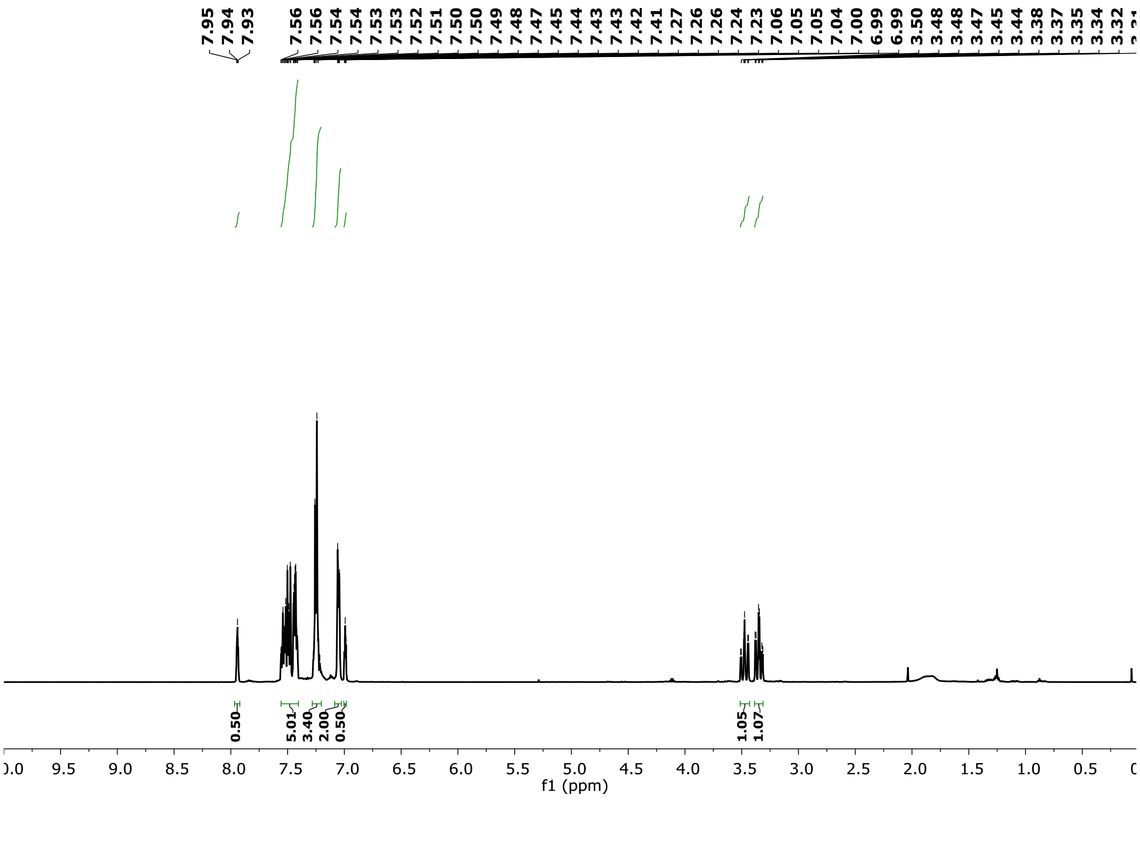

$^{13}C\{^1H\}$ NMR (125.8 MHz, $CDCl_3$)

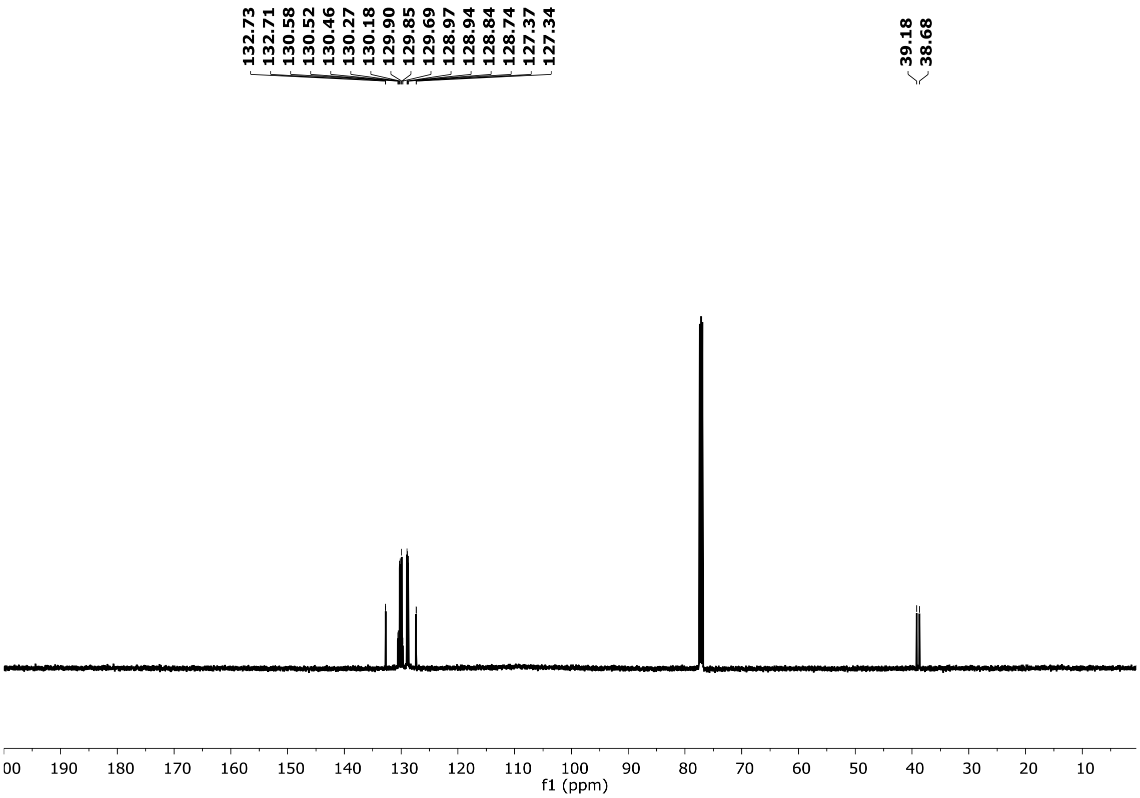

(*R*)-Methyl-phenylphosphine oxide [(*R*)-**1k**]

$^{31}P\{^{1}H\}$ NMR (202.5 MHz, $CDCl_3$)

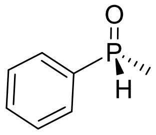

(*R*)-**1k**

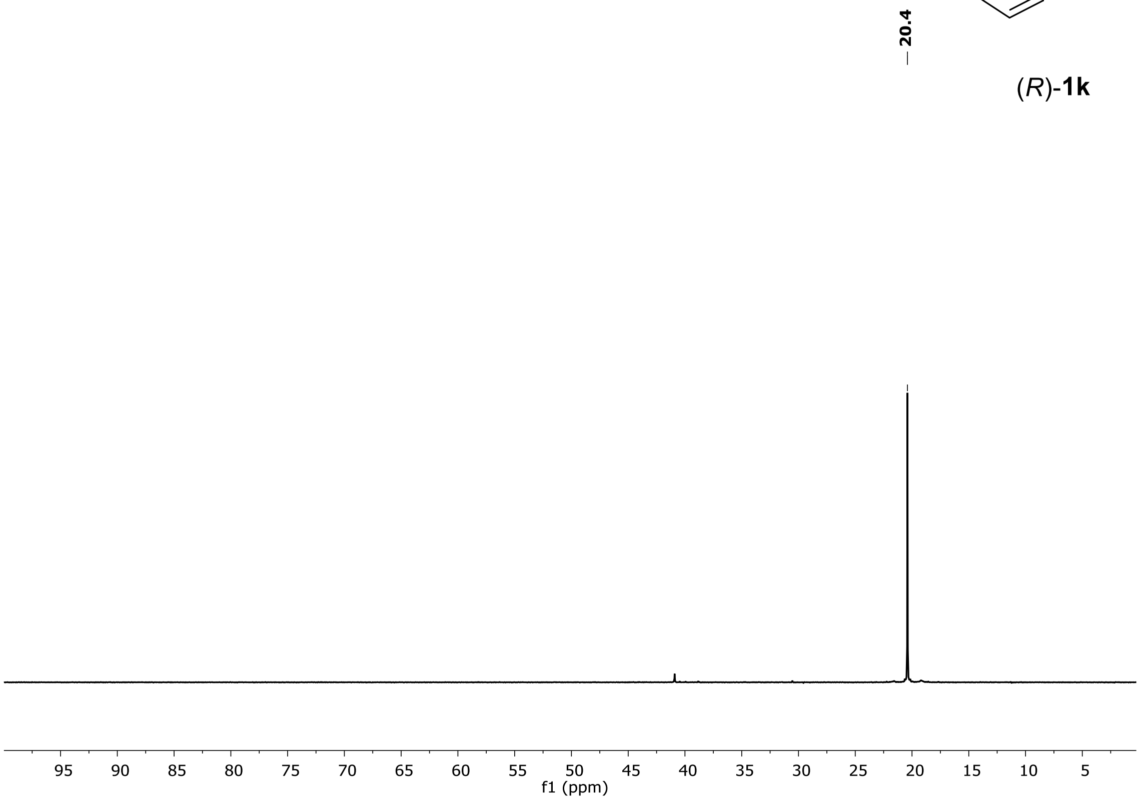

^{1}H NMR (500 MHz, $CDCl_3$)

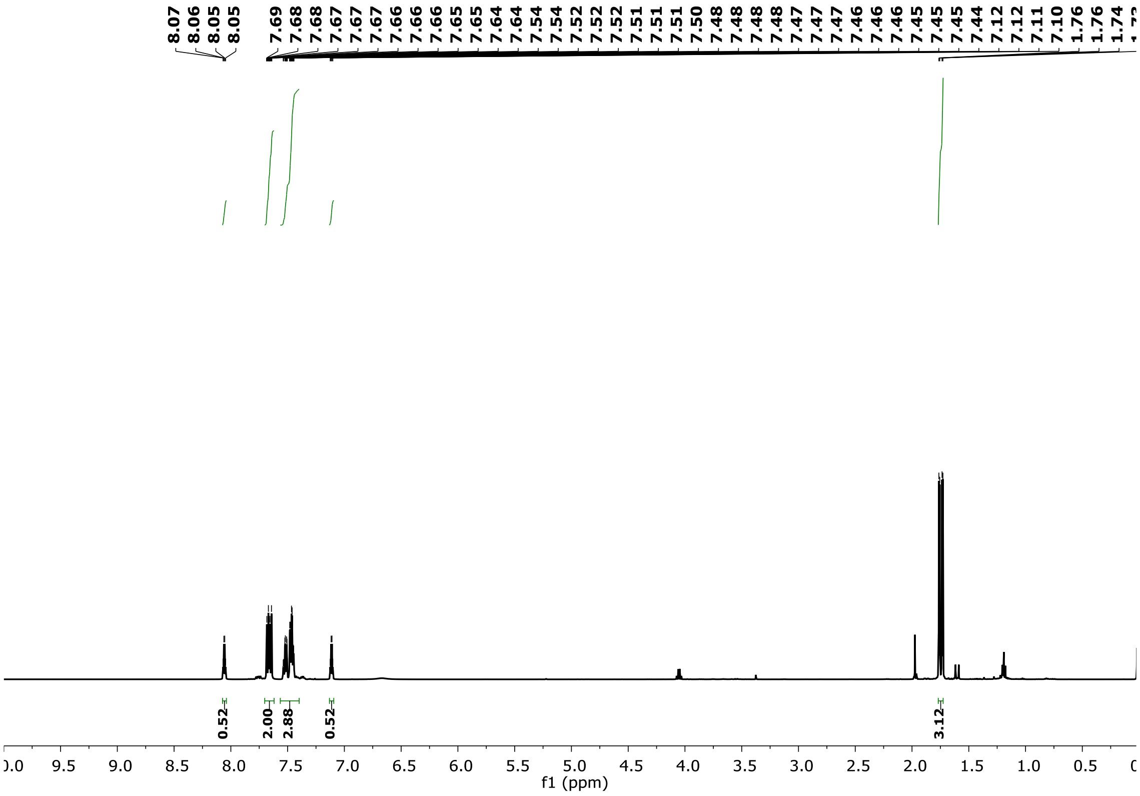

$^{13}C\{^1H\}$ NMR (125.8 MHz, $CDCl_3$)

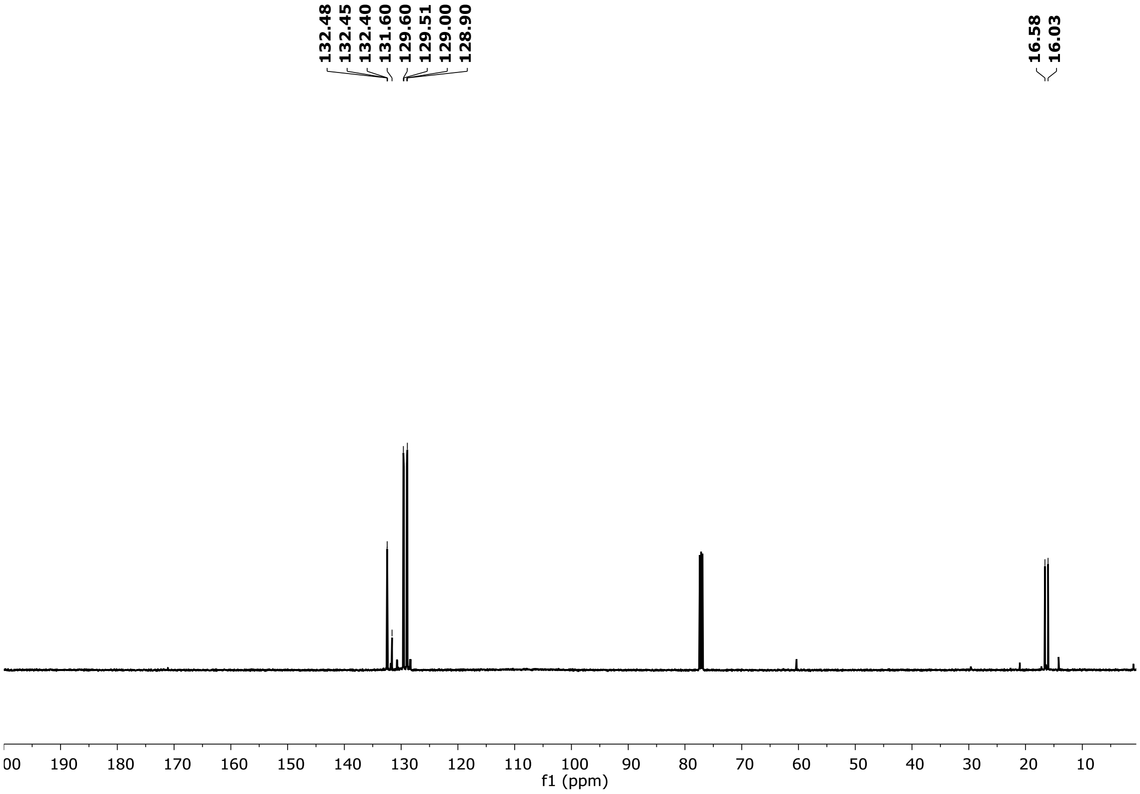

(*S*)-Butyl-phenylphosphine oxide [(*S*)-**1l**]

$^{31}P\{^{1}H\}$ NMR (202.5 MHz, $CDCl_3$)

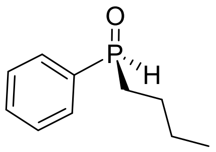

(*S*)-**1l**

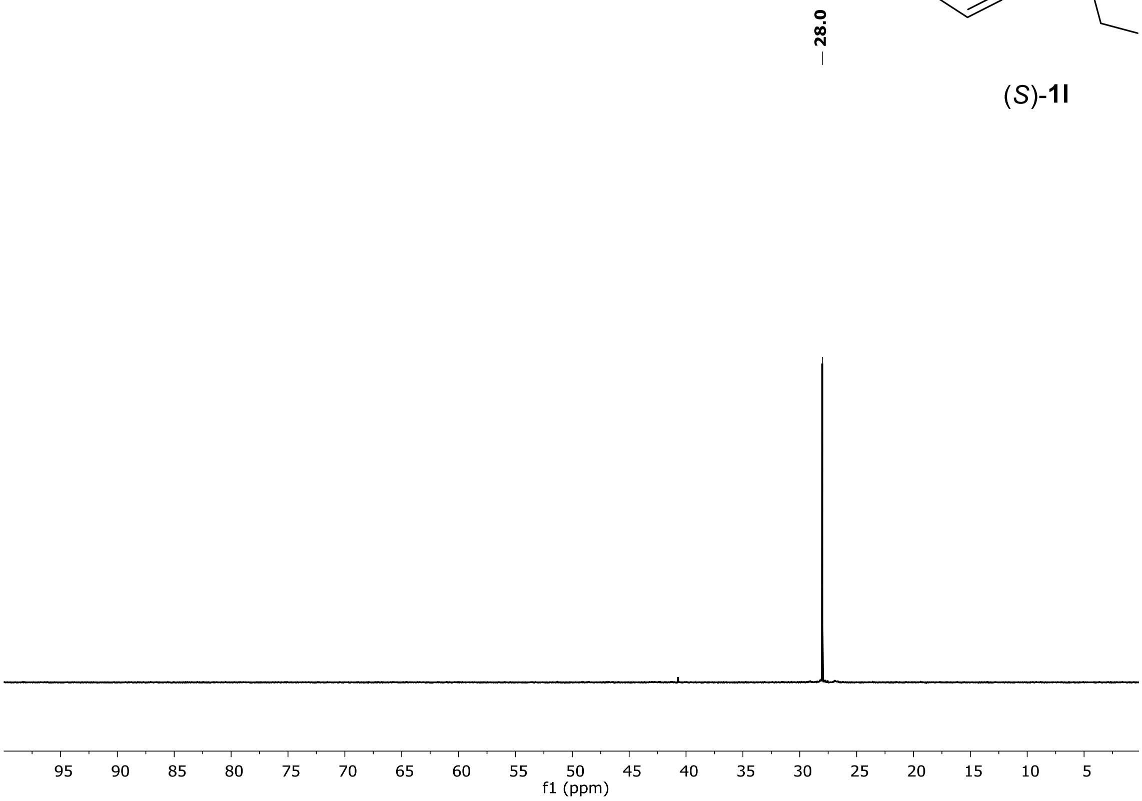

^{1}H NMR (500 MHz, $CDCl_3$)

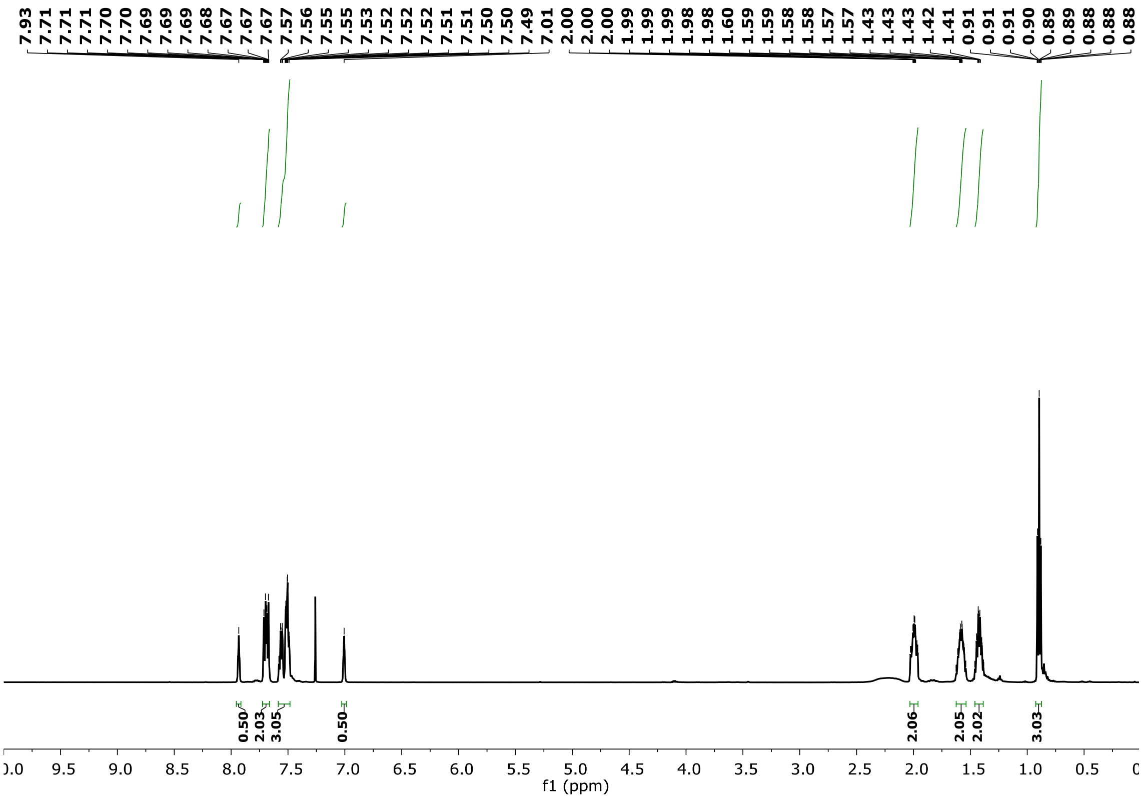

$^{13}C\{^{1}H\}$ NMR (125.8 MHz, $CDCl_3$)

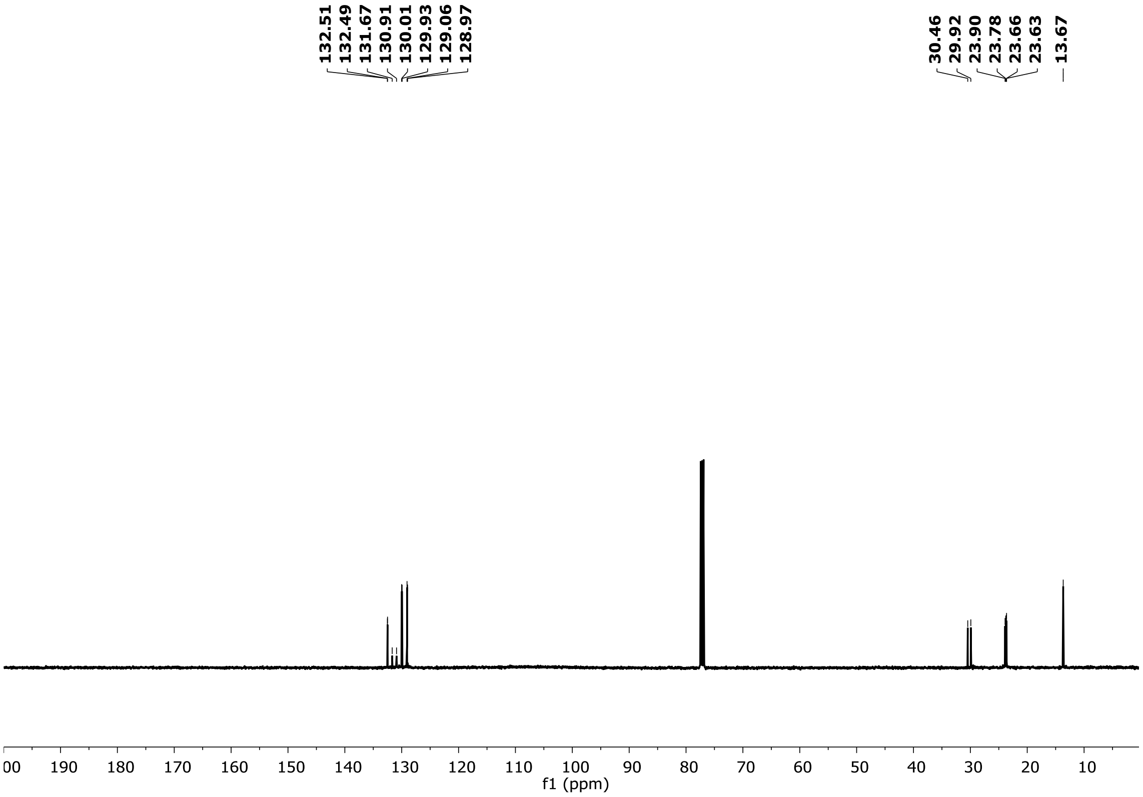

(*R*)-*tert*-Butyl-phenylphosphine oxide [(*R*)-**1m**]

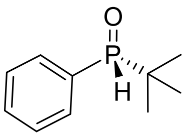

(*R*)-**1m**

$^{31}P\{^{1}H\}$ NMR (202.5 MHz, $CDCl_3$)

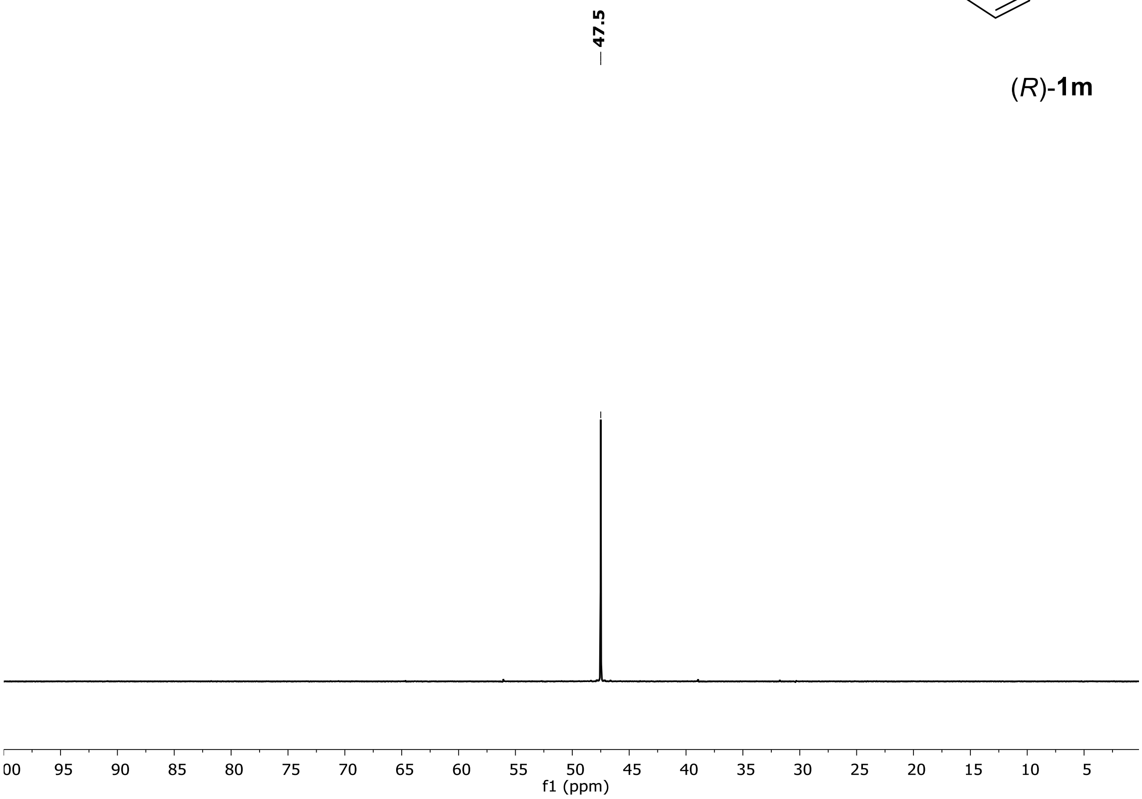

^{1}H NMR (500 MHz, $CDCl_3$)

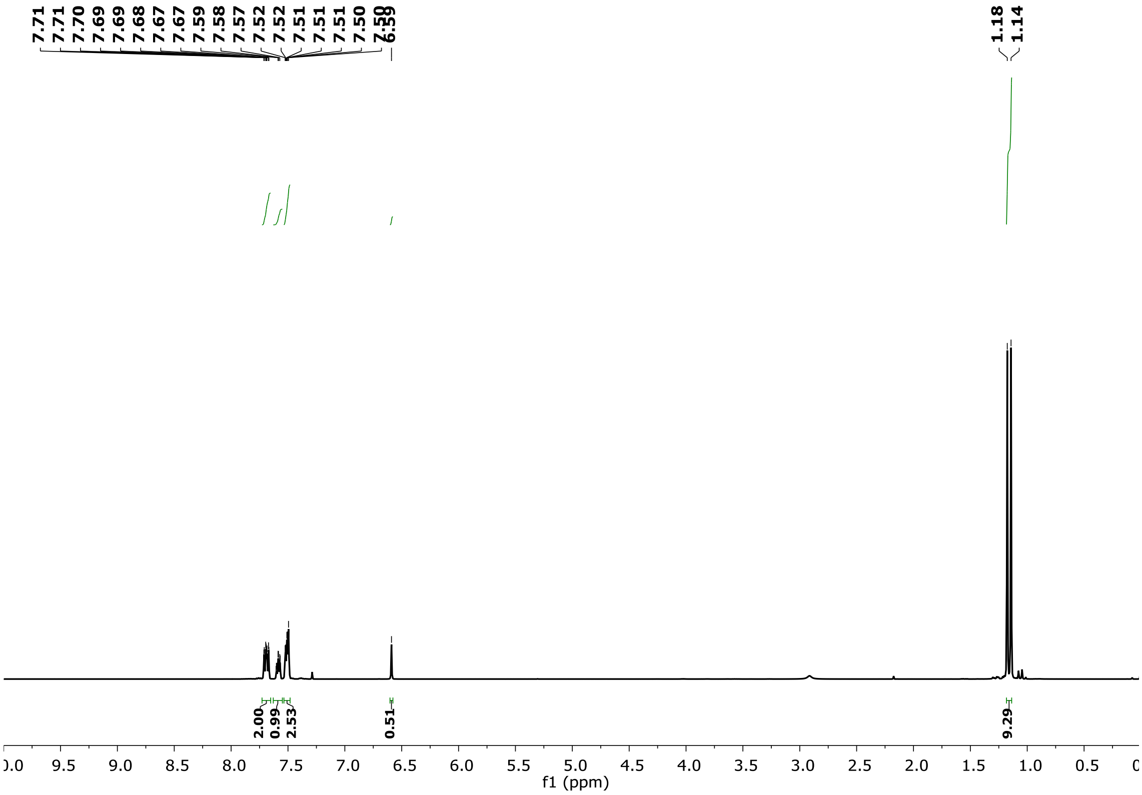

$^{13}C\{^{1}H\}$ NMR (125.8 MHz, $CDCl_3$)

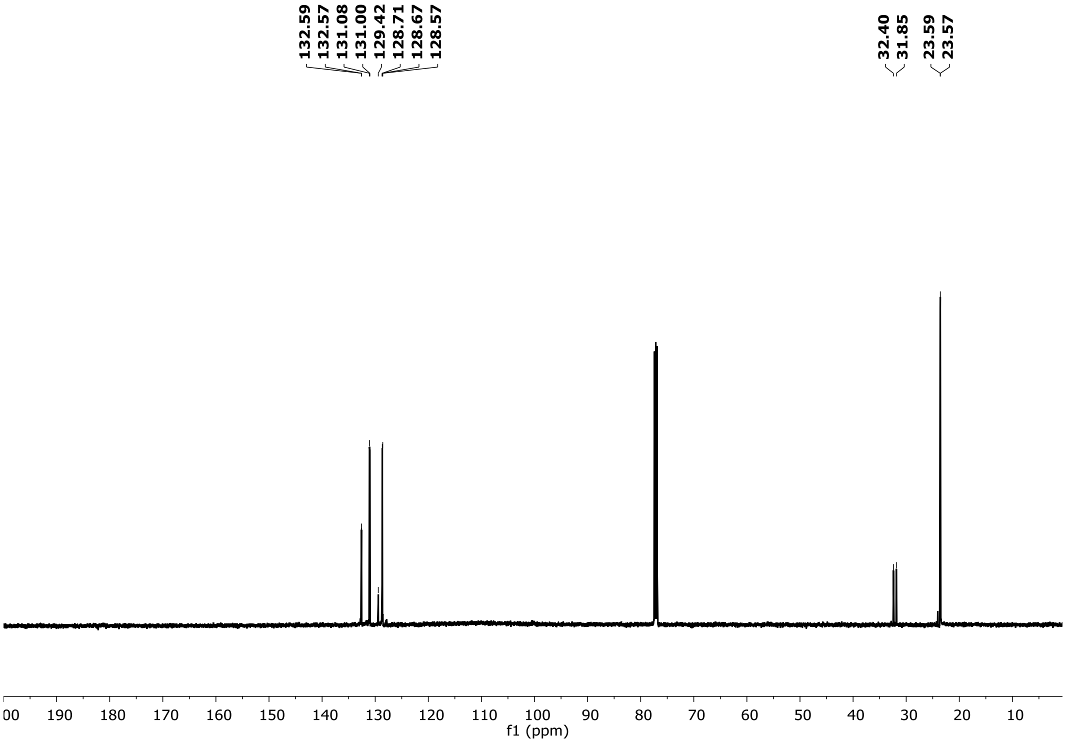

(*S*)-Cyclohexyl-phenylphosphine oxide [(*S*)-**1n**]

$^{31}P\{^{1}H\}$ NMR (202.5 MHz, $CDCl_3$)

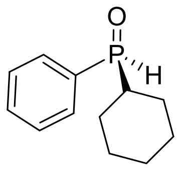

(*S*)-**1n**

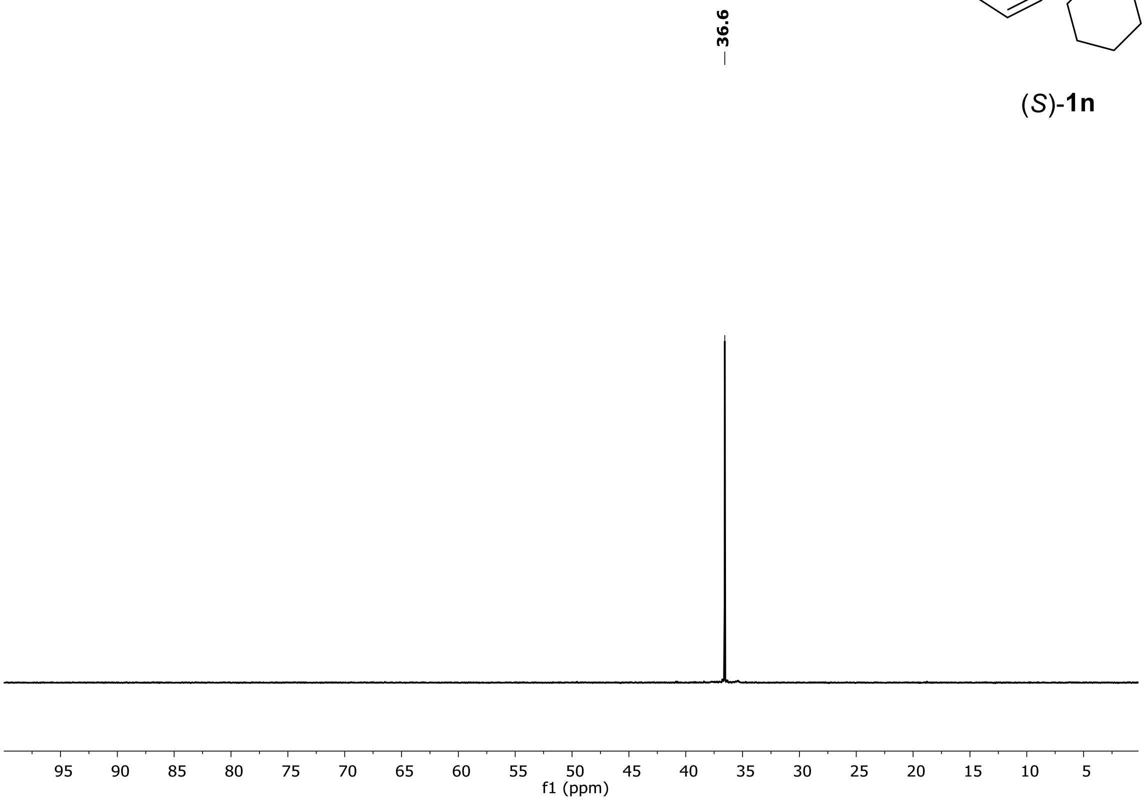

^{1}H NMR (500 MHz, $CDCl_3$)

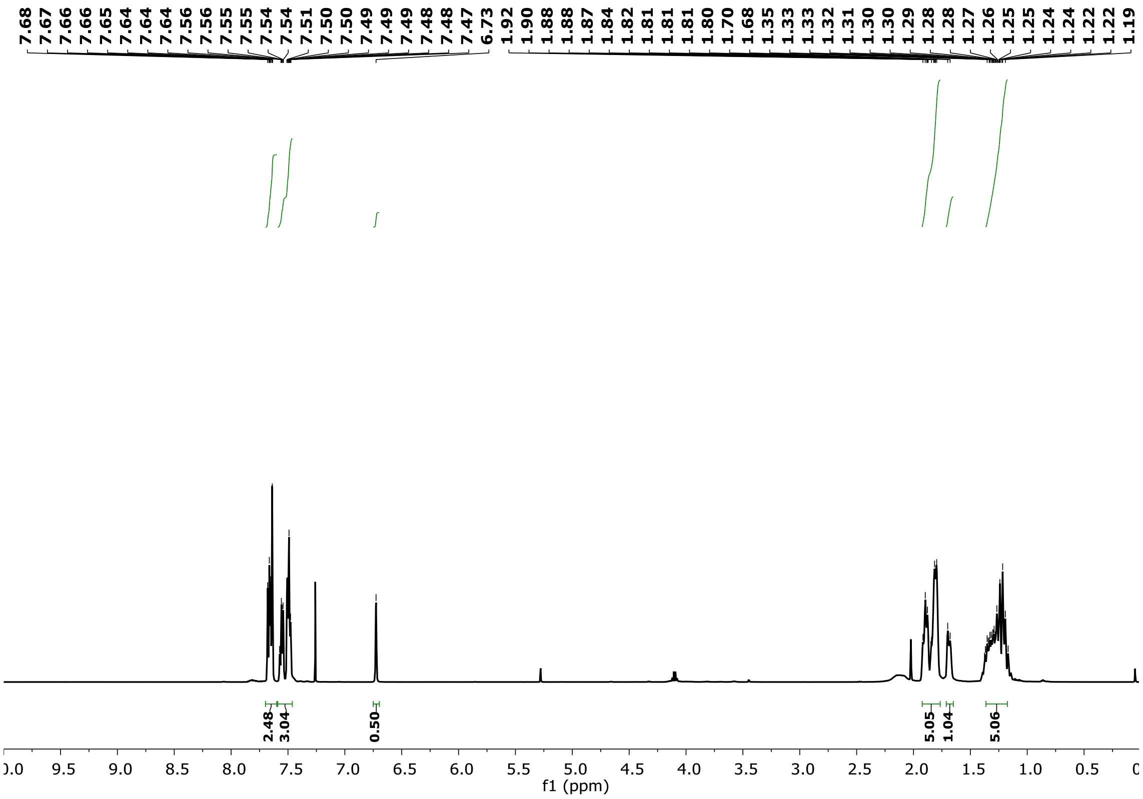

$^{13}C\{^{1}H\}$ NMR (125.8 MHz, $CDCl_3$)

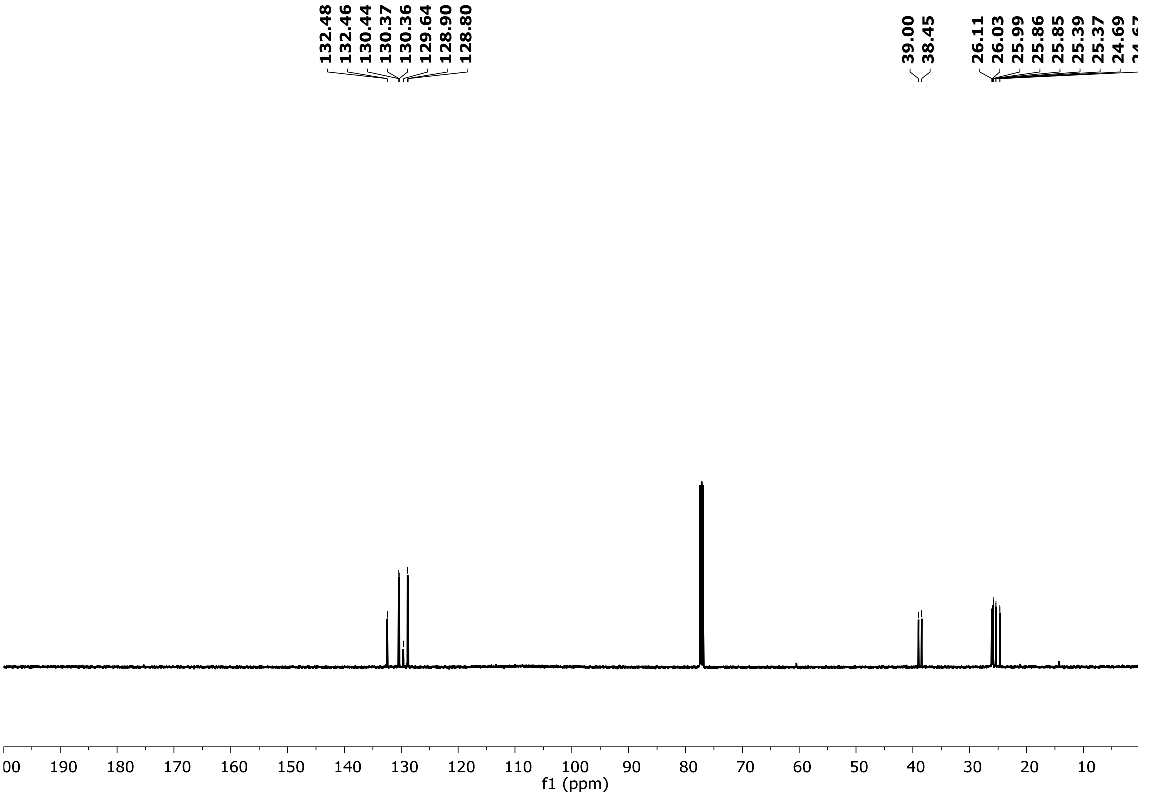

(*S*)-methyl-(2-methylphenyl)-phenylphosphine oxide [(*S*)-**3a**]

$^{31}P\{^{1}H\}$ NMR (121.5, MHz, $CDCl_3$)

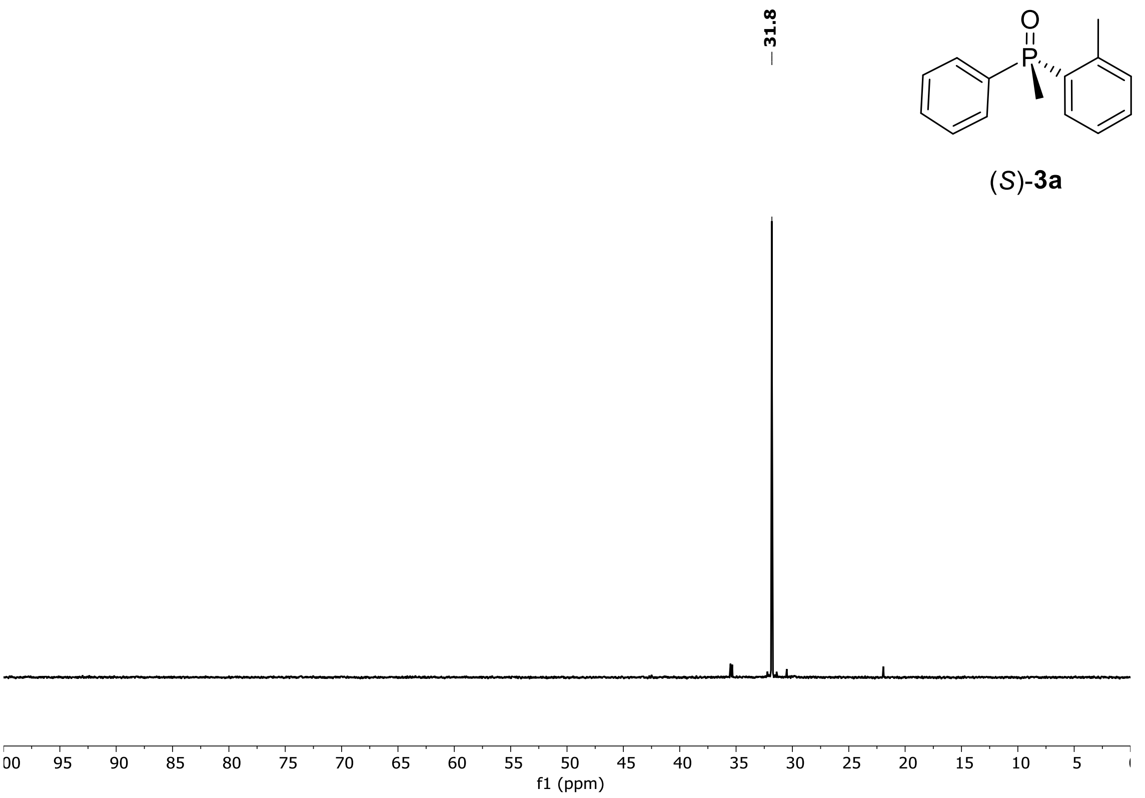

^{1}H NMR (500 MHz, $CDCl_3$)

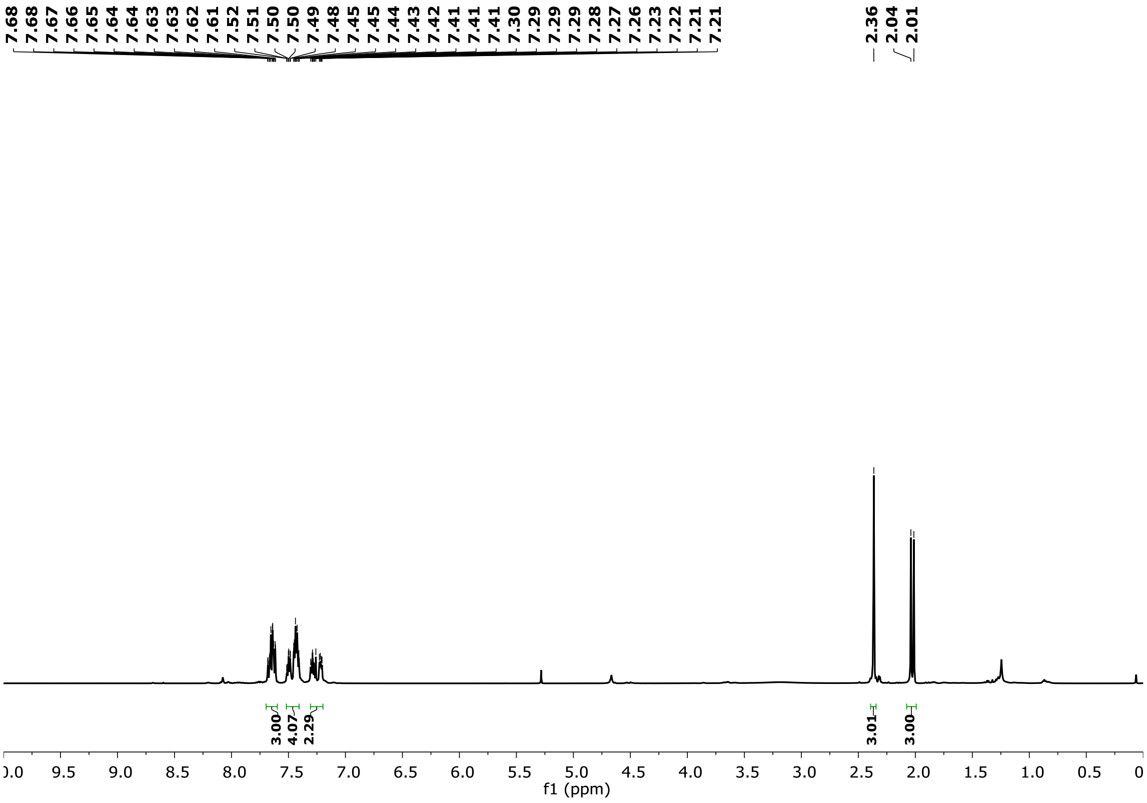

$^{13}C\{^{1}H\}$ NMR (75.5 MHz, $CDCl_3$)

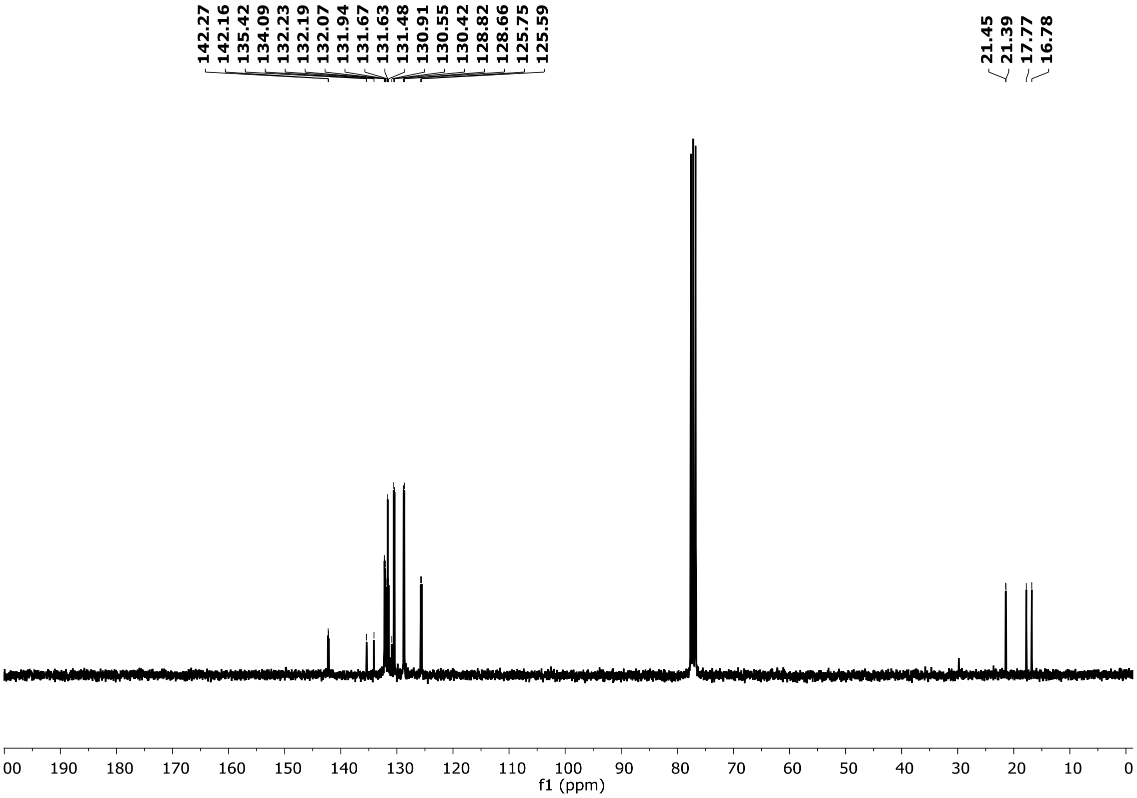

(*S*)-ethyl-(2-methylphenyl)-phenylphosphine oxide [(*S*)-**3b**]

$^{31}P\{^{1}H\}$ NMR (121.5, MHz, $CDCl_3$)

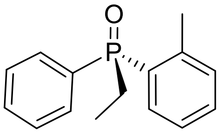

(*S*)-**3b**

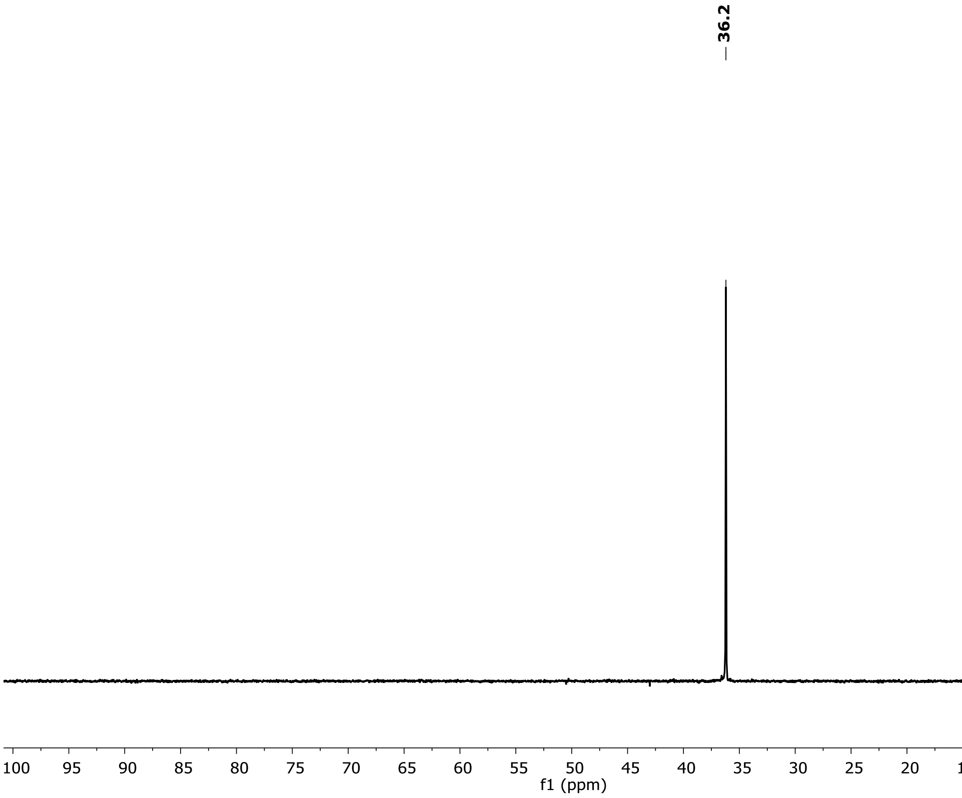

^{1}H NMR (500 MHz, $CDCl_3$)

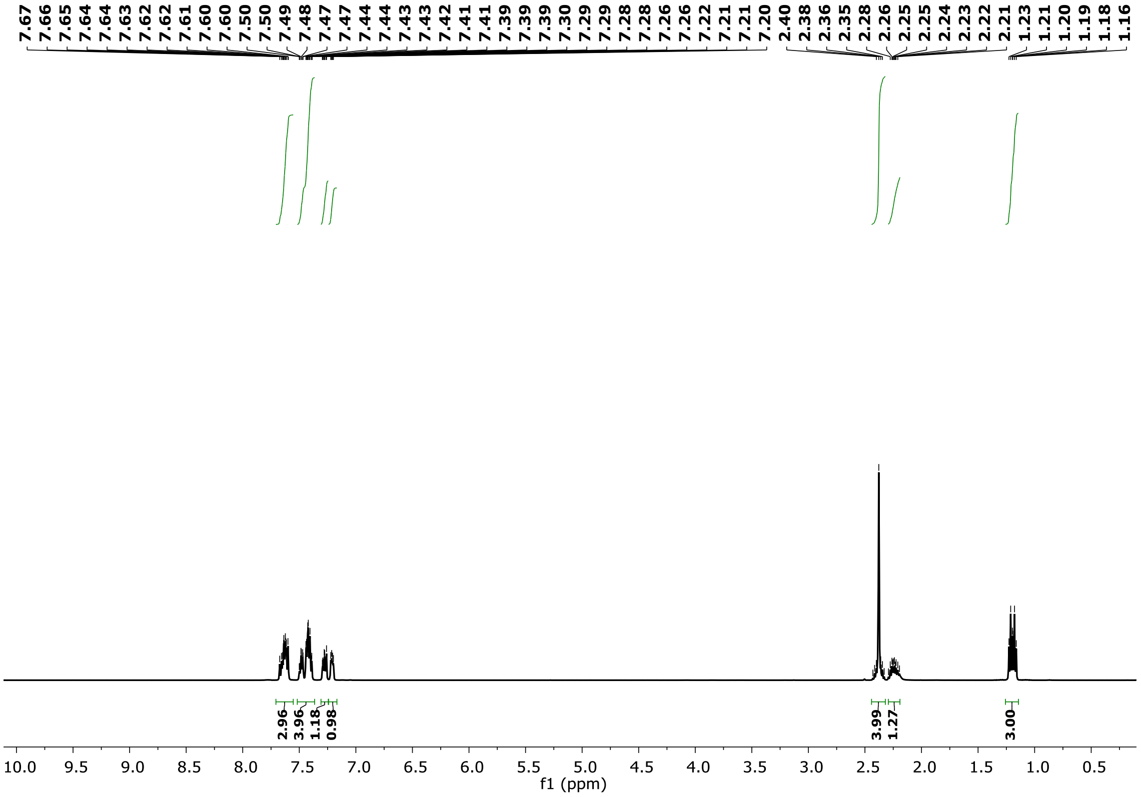

$^{13}C\{^{1}H\}$ NMR (75.5 MHz, $CDCl_3$)

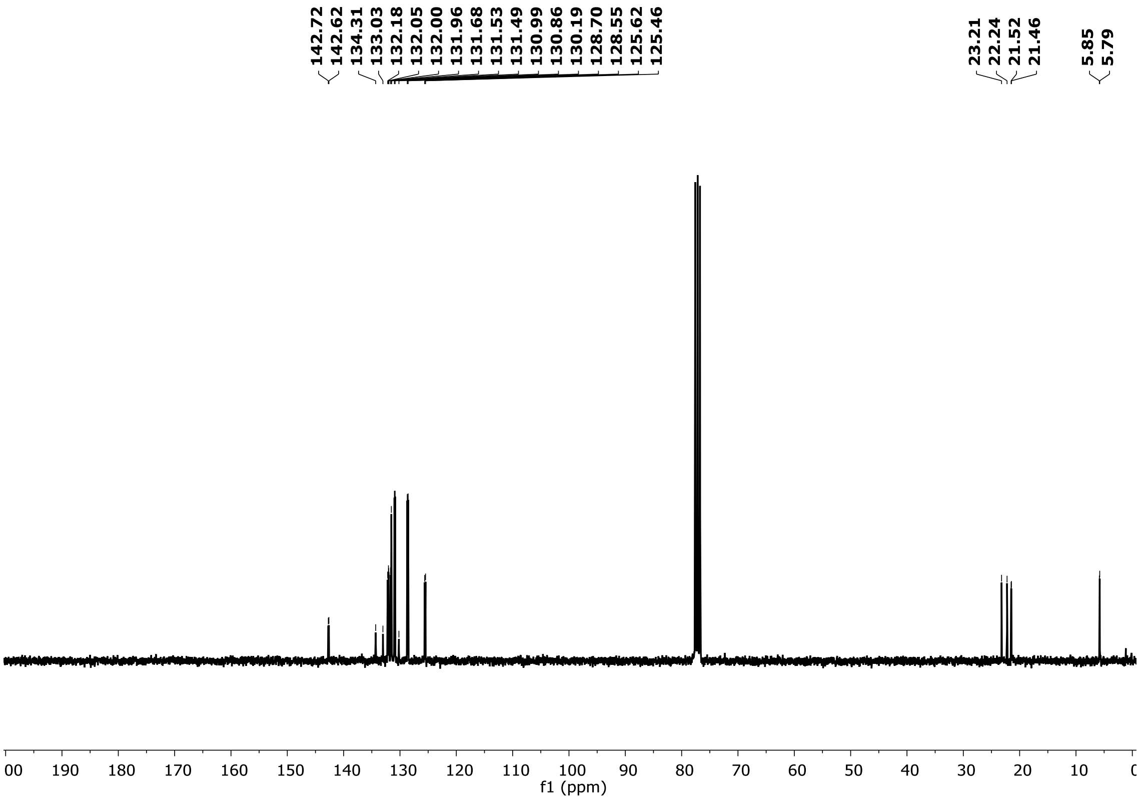

(*S*)-benzyl-(2-methylphenyl)-phenylphosphine oxide [(*S*)-**3c**]

$^{31}P\{^{1}H\}$ NMR (121.5, MHz, $CDCl_3$)

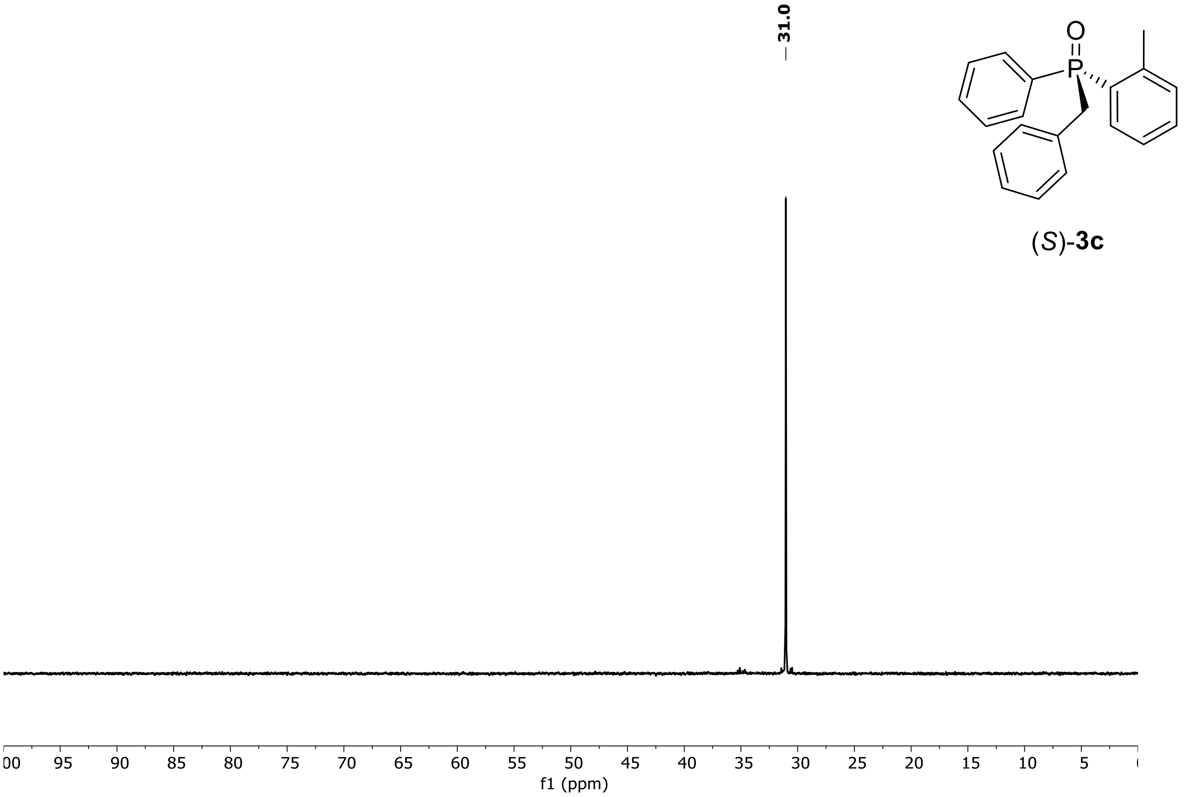

^{1}H NMR (500 MHz, $CDCl_3$)

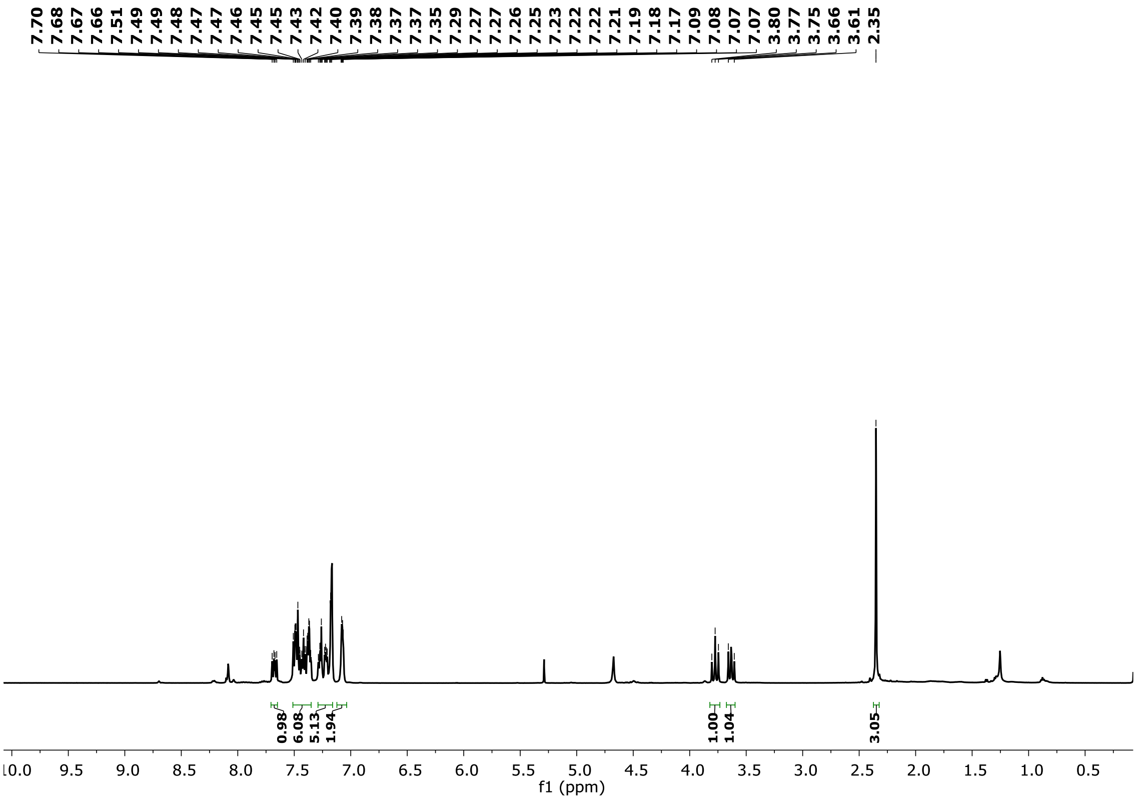

$^{13}C\{^{1}H\}$ NMR (75.5 MHz, $CDCl_3$)

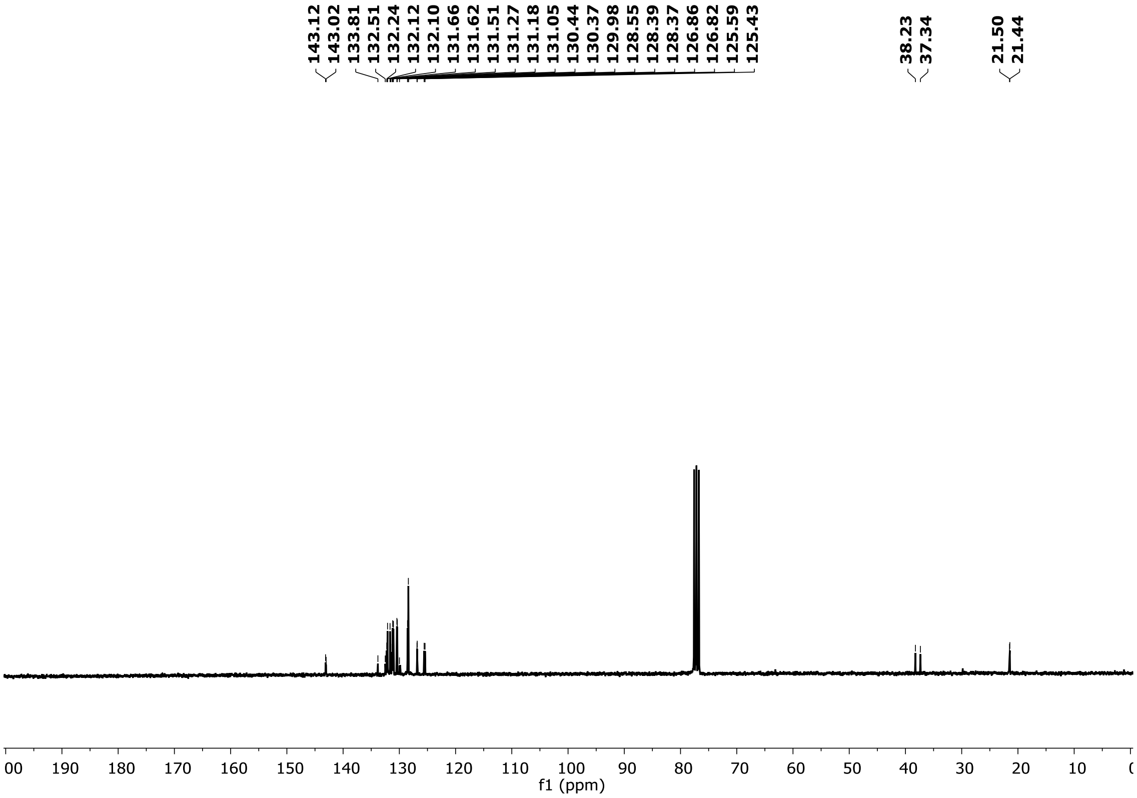

(*S*)-(1-napthyl)-(2-methylphenyl)-phenylphosphine oxide [(*S*)-**3d**]

$^{31}P\{^{1}H\}$ NMR (121.5, MHz, $CDCl_3$)

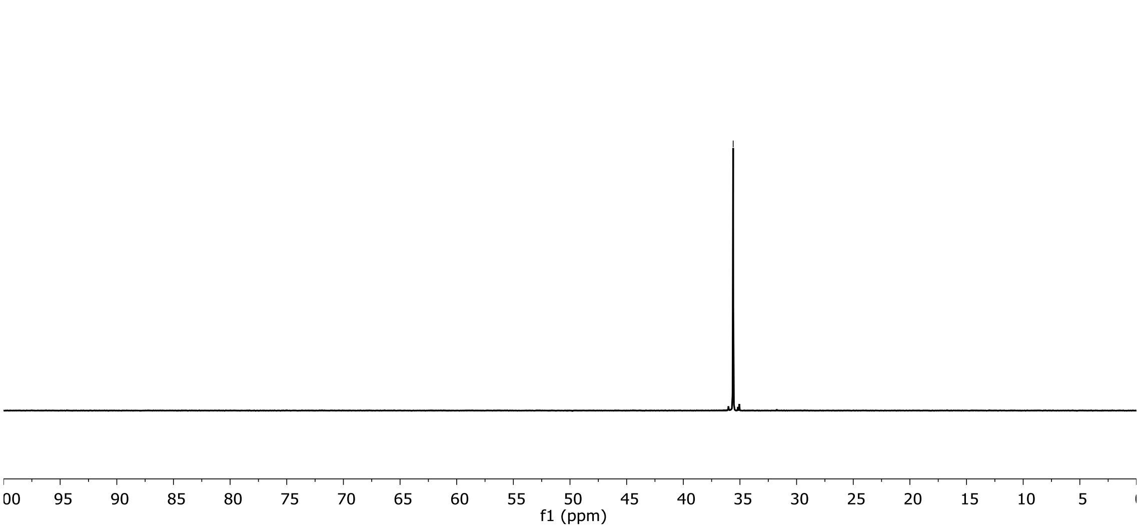

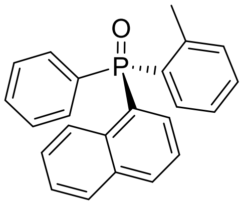

(*S*)-**3d**

^{1}H NMR (500 MHz, $CDCl_3$)

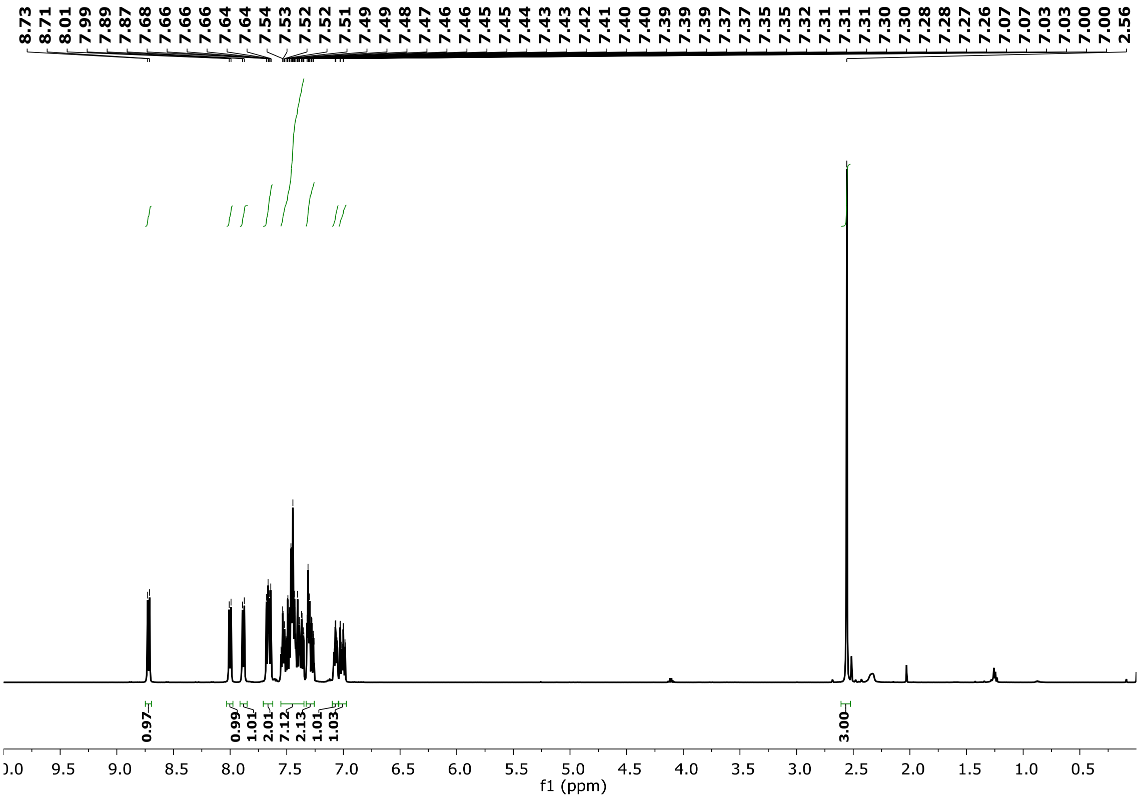

$^{13}C\{^{1}H\}$ NMR (125.8 MHz, $CDCl_3$)

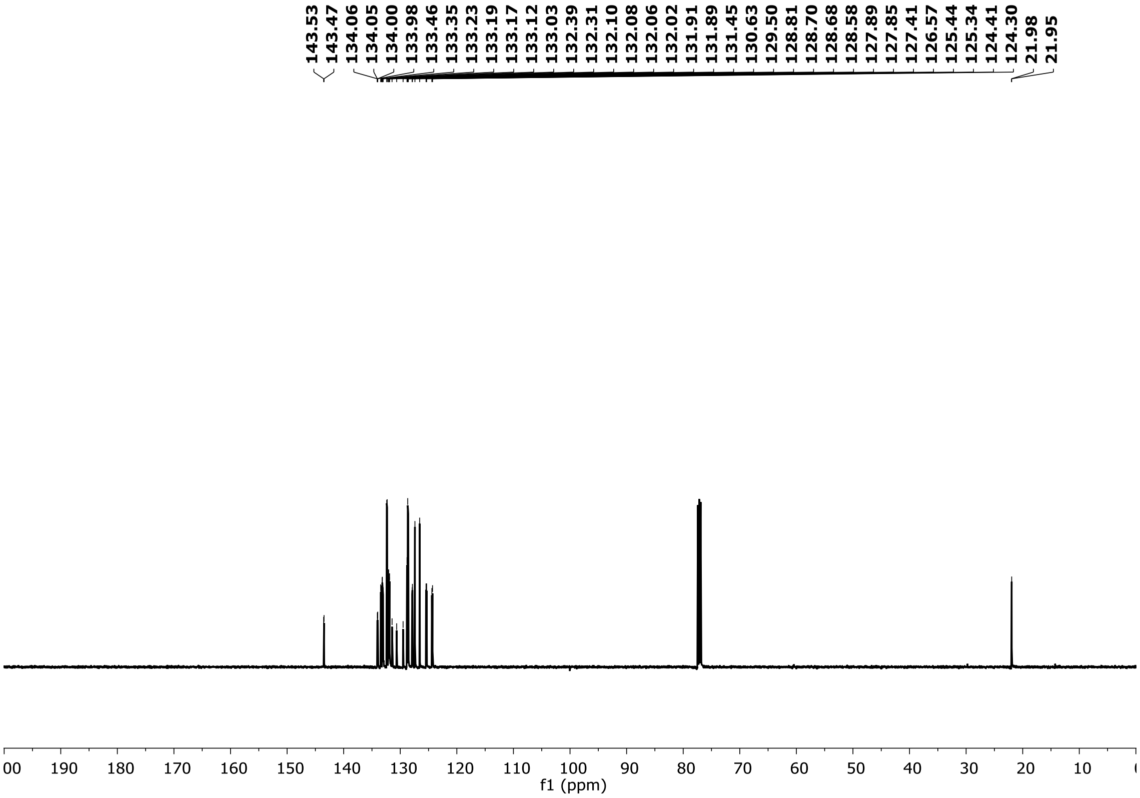

(*R*)-(2-methylphenyl)-phenylphosphothioic acid [(*R*)-**3e**]

$^{31}P\{^{1}H\}$ NMR (202.5, MHz, $CDCl_3$)

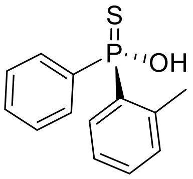

(*R*)-**3e**

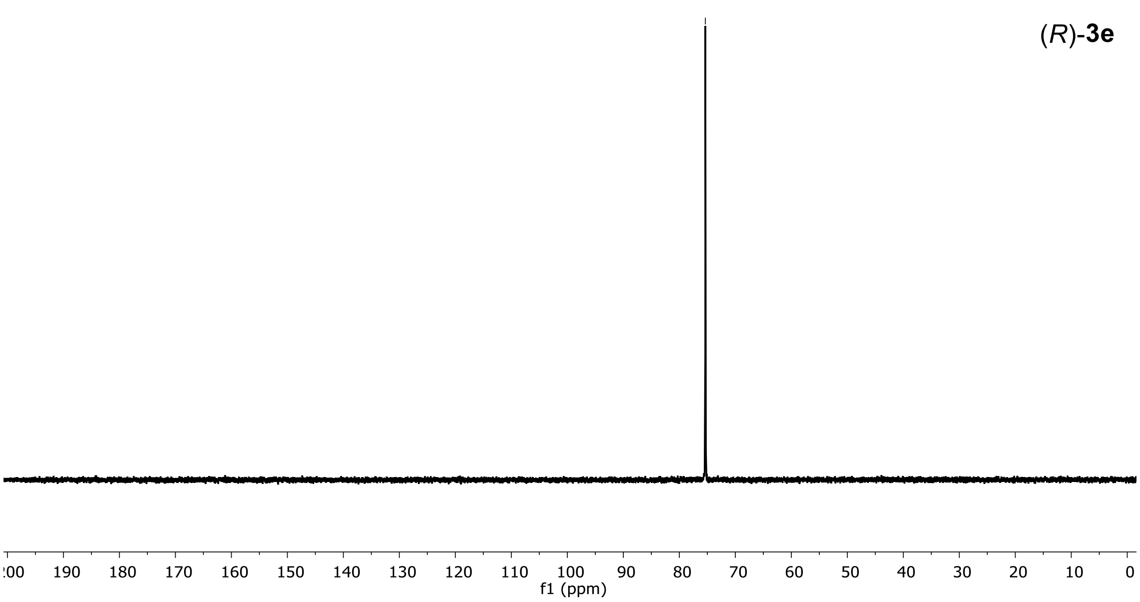

^{1}H NMR (500 MHz, $CDCl_3$)

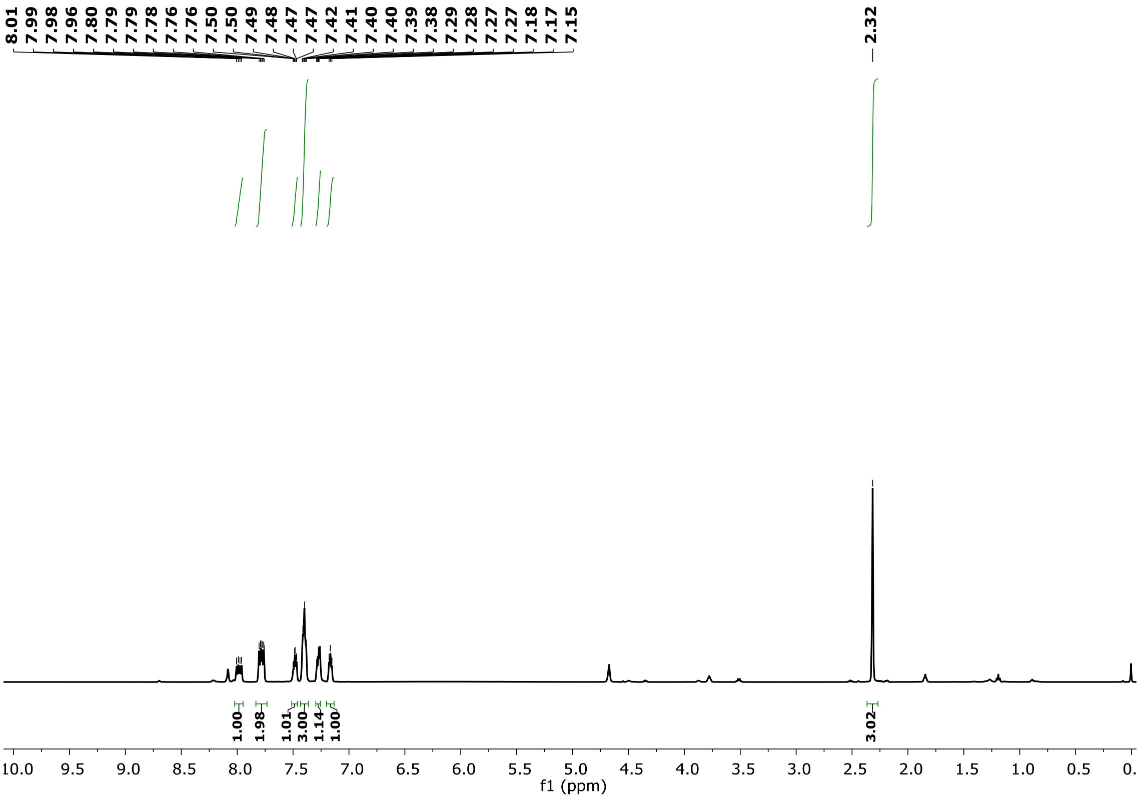

$^{13}C\{^{1}H\}$ NMR (125.8 MHz, $CDCl_3$)

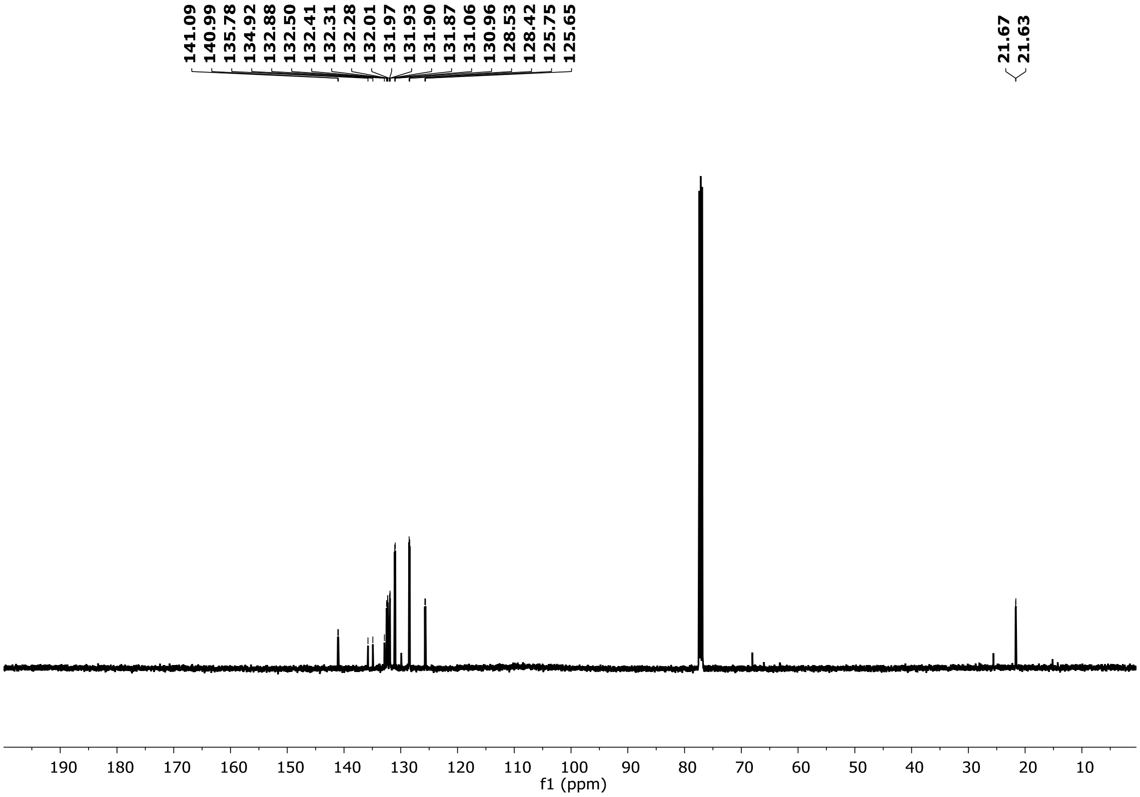

(*S*)-hydroxymethyl-(2-methylphenyl)-phenylphosphine oxide [(*S*)-**3f**]

$^{31}P\{^{1}H\}$ NMR (202.5, MHz, DMSO-*d*6)

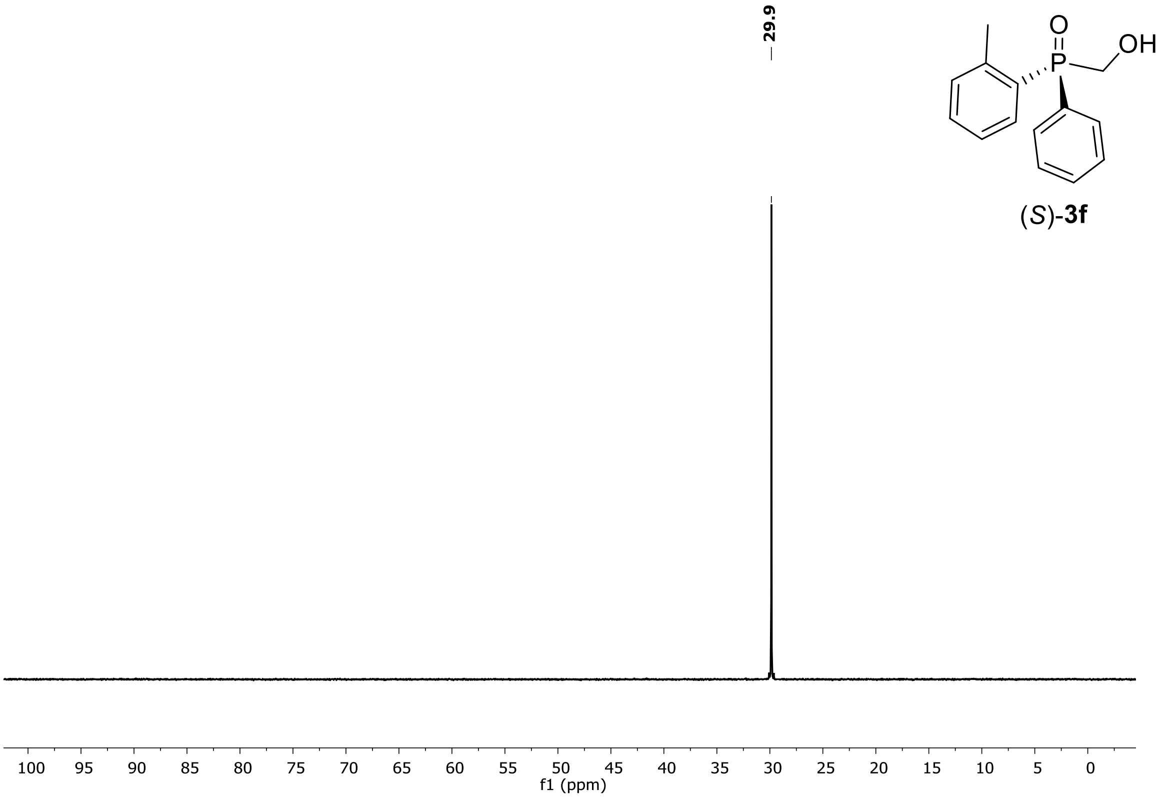

^{1}H NMR (500 MHz, DMSO-*d*6)

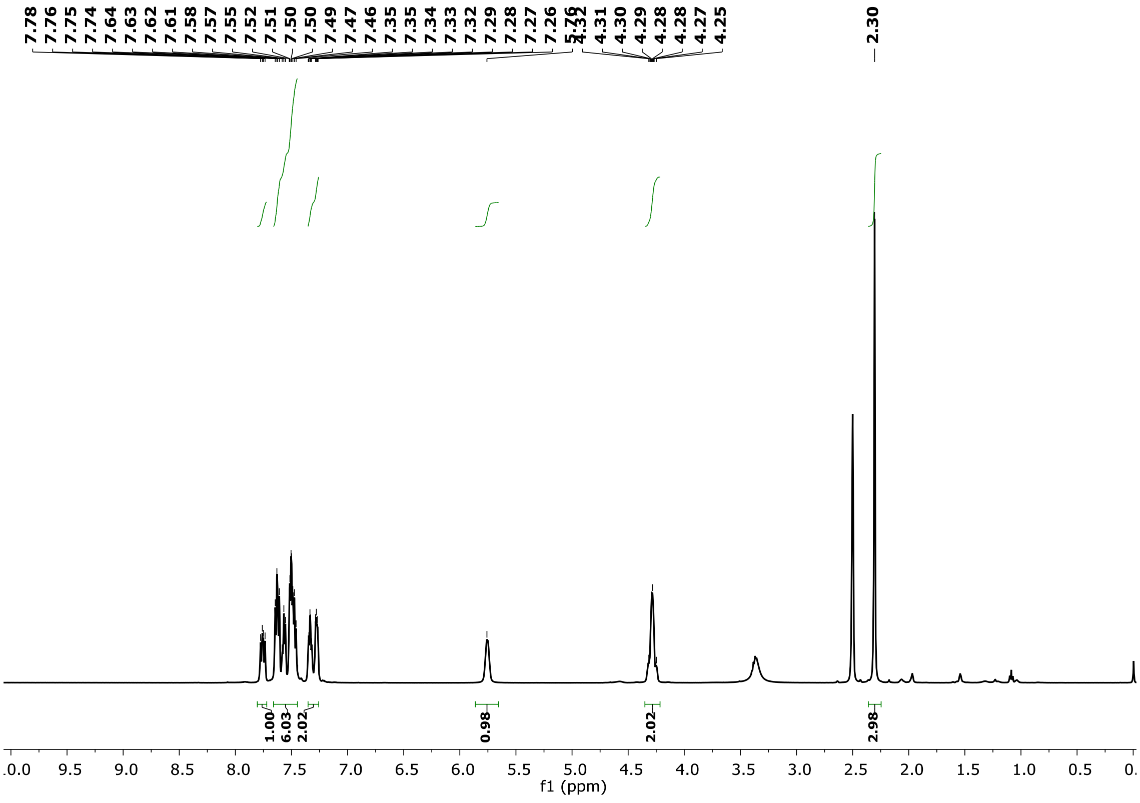

$^{13}C\{^1H\}$ NMR (125.8 MHz, DMSO-*d*6)

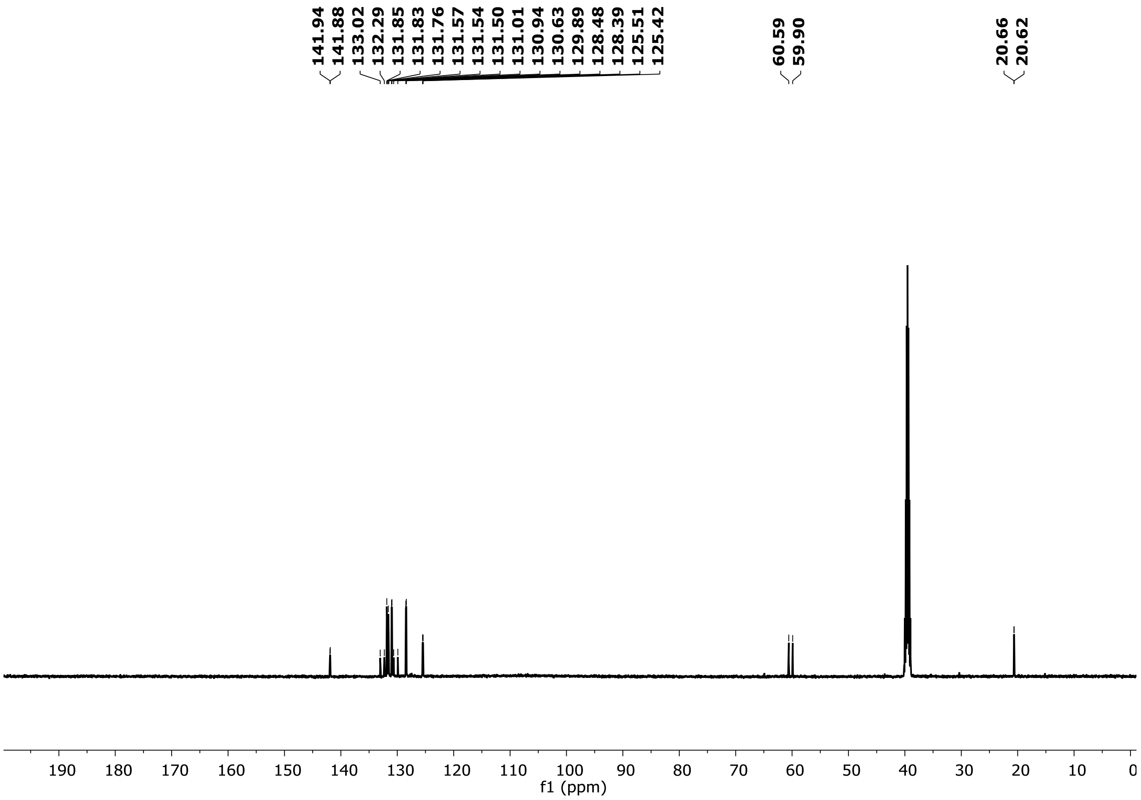

(S_P)-[(R_C)-hydroxy(phenyl)methyl]-(2-methylphenyl)-phenylphosphine oxide [(S_P,R_C)-**3g**]

$^{31}P\{^1H\}$ NMR (121.5, MHz, DMSO-*d*6)

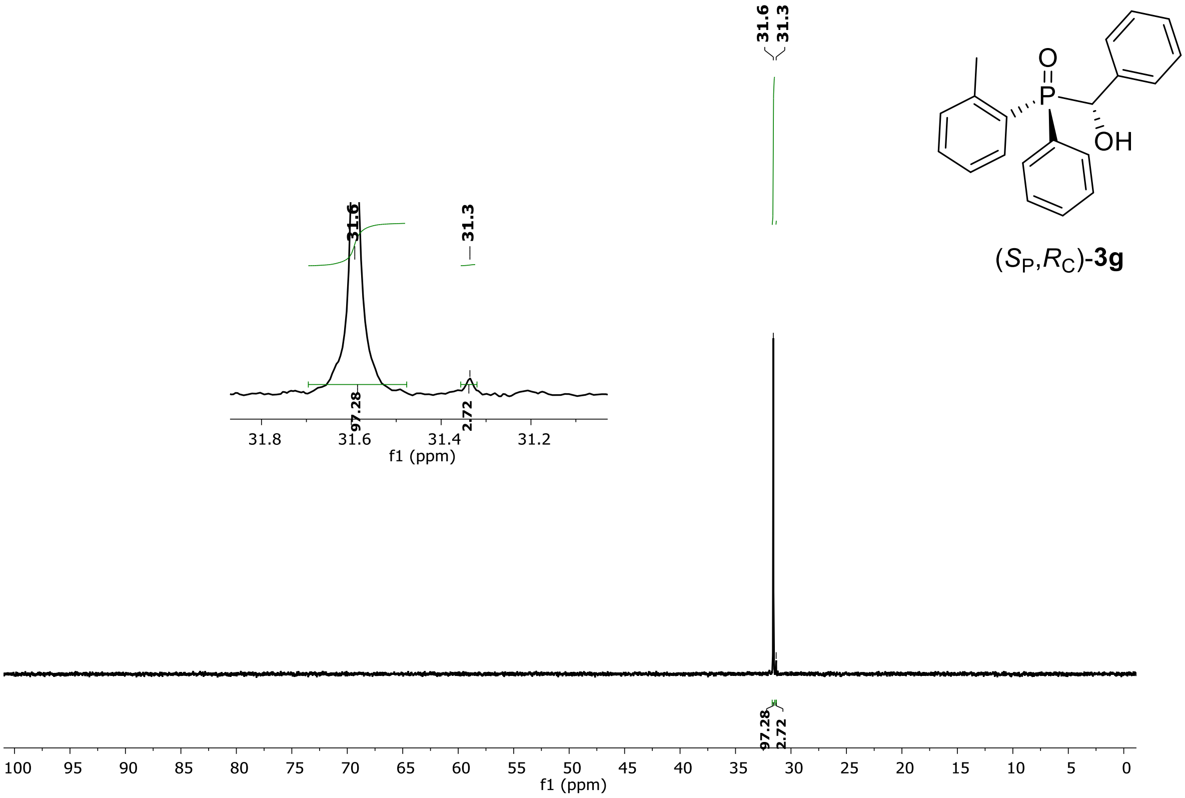

(S_P,R_C)-**3g**

^1H NMR (500 MHz, DMSO-*d*6)

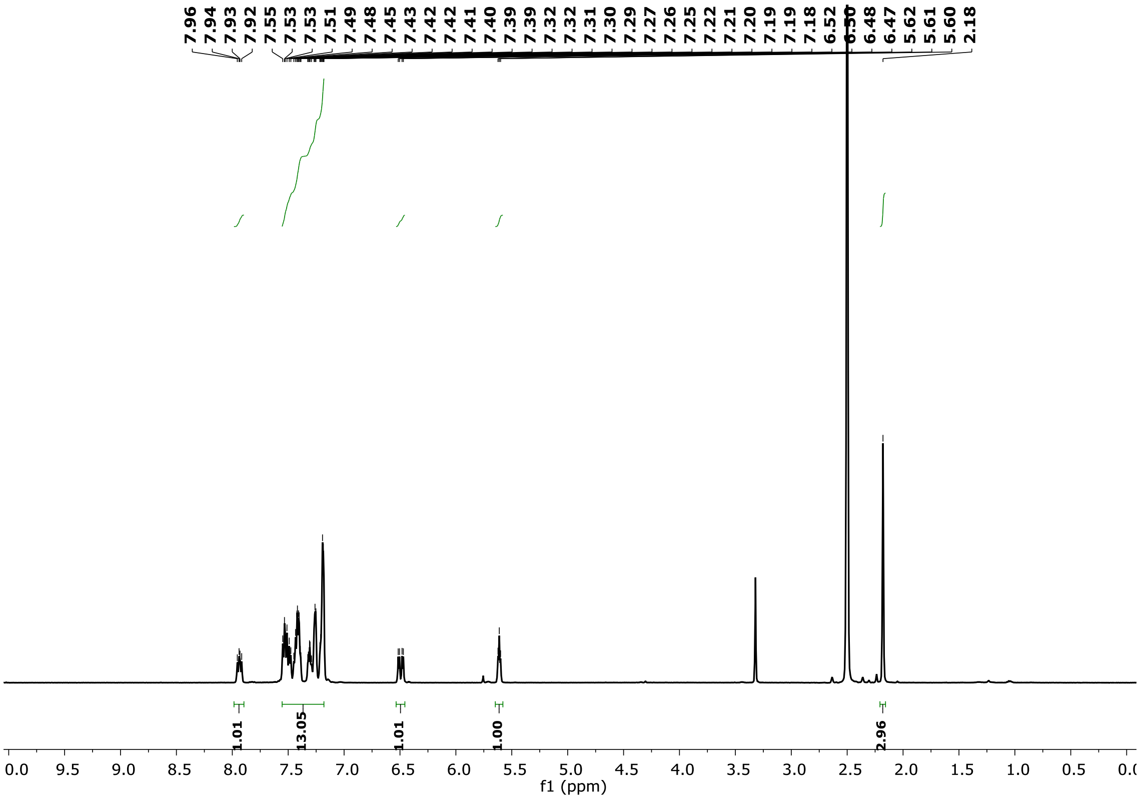

$^{13}C\{^1H\}$ NMR (75.5 MHz, DMSO-*d*6)

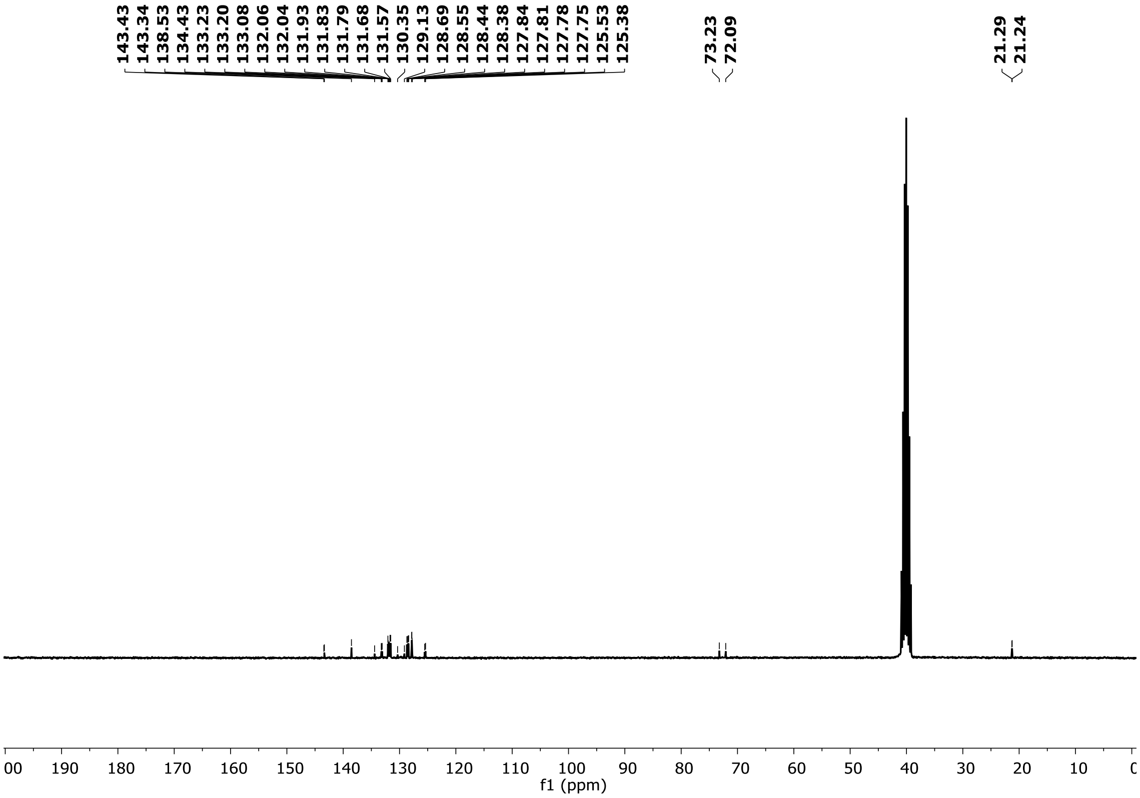

((2*R*,3*R*)-1,4-dioxaspiro[4.5]decane-2,3-diyl)bis(bis(4-(*tert*-butyl)phenyl)methanol) **[(*R*,*R*)-SI-2]**

^{1}H NMR (500 MHz, $CDCl_3$)

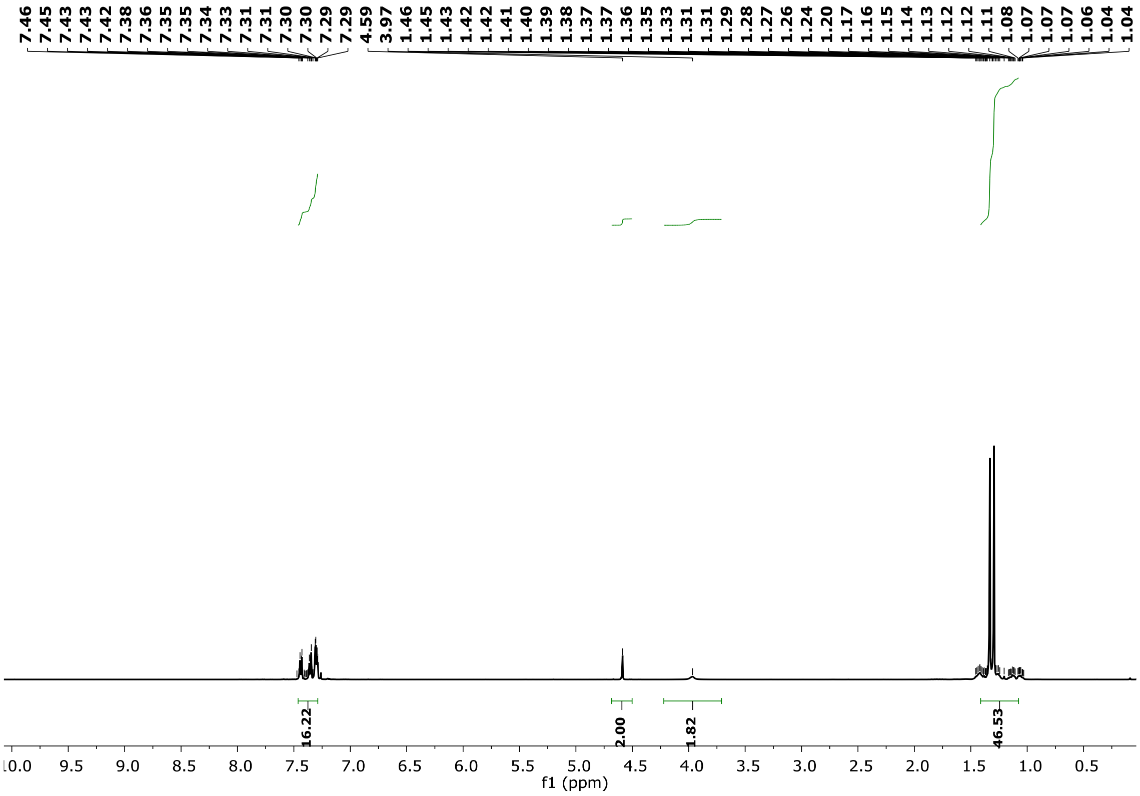

^{13}C{^{1}H} NMR (125.8 MHz, $CDCl_3$)

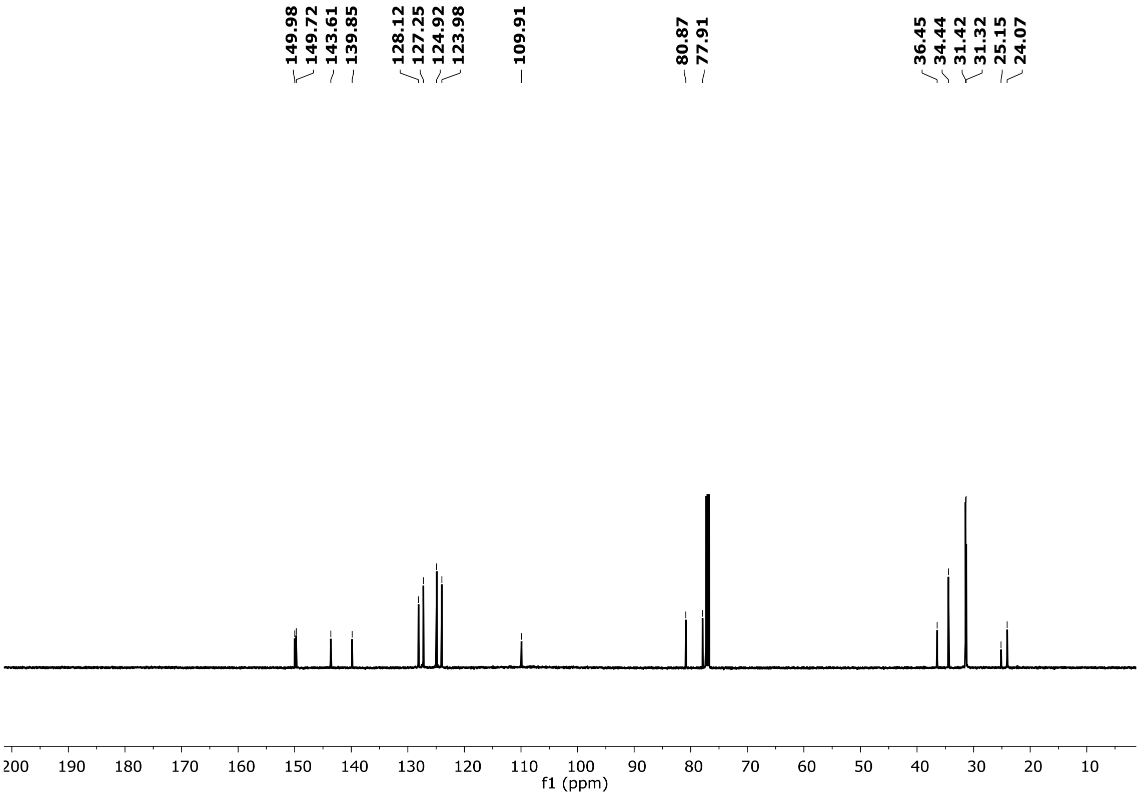

HPLC traces of the optically active secondary and tertiary phosphine oxides (1 and 3)

Table S7 HPLC parameters for the *ee* determination of phosphine oxides (**1** and **3**)

Y^1, Y^2	Column	Hexane : Ethanol ratio	Retention time 1 (min)	Enantiomer 1	Retention time 2 (min)	Enantiomer 2
2-Me-C_6H_4, H (**1a**)	Amylose-2[a]	85:15	20.8	(*R*)	22.5	(*S*)
3-Me-C_6H_4, H (**1b**)	Cellulose-2[b]	50:50	11.1	(−)	12.8	(+)
4-Me-C_6H_4, H (**1c**)	Amylose-2[a]	50:50	10.3	(−)	11.2	(+)
2-CF_3-C_6H_4, H (**1d**)	Cellulose-2[b]	50:50	7.3	-	7.9	-
3-CF_3-C_6H_4, H (**1e**)	Amylose-2[a]	50:50	6.7	(+)	8.7	(−)
4-CF_3-C_6H_4, H (**1f**)	Amylose-2[a]	50:50	6.4	(+)	8.3	(−)
2-OMe-C_6H_4, H (**1g**)	Cellulose-1[c]	85:15	10.8	(*S*)	15.4	(*R*)
2-Ph-C_6H_4, H (**1h**)	Cellulose-2[b]	50:50	10.0	(*R*)	12.0	(*S*)
1-Naph, H (**1i**)	Amylose-2[a]	50:50	8.9	(*R*)	9.8	(*S*)
Bn, H (**1j**)	Amylose-2[a]	50:50	9.7	(*S*)	16.1	(*R*)
Me, H (**1k**)	Cellulose-2[b]	50:50	9.1	(*S*)	10.8	(*R*)
Bu, H (**1l**)	Amylose-2[a]	50:50	7.1	(*S*)	7.8	(*R*)
^{t}Bu, H (**1m**)	Amycoat[d]	85:15	8.8	(*S*)	11.9	(*R*)
^{c}Hex, H (**1n**)	Cellulose-2[b]	50:50	10.0	(*S*)	19.7	(*R*)
2-Me-C_6H_4, Me (**3a**)	Amycoat[d]	85:15	10.9	(*S*)	13.5	(*R*)
2-Me-C_6H_4, Et (**3b**)	Amycoat[d]	85:15	8.4	(*S*)	12.6	(*R*)
2-Me-C_6H_4, Bn (**3c**)	Amylose-2[a]	85:15	17.2	(*S*)	24.3	(*R*)
2-Me-C_6H_4, 1-Naph (**3d**)	Amycoat[d]	80:20	8.7	(*R*)	11.1	(*S*)
2-Me-C_6H_4, CH_2OH (**3f**)	Cellulose-4[e]	50:50	8.6	(*S*)	9.2	(*R*)
2-Me-C_6H_4, CH(OH)Ph (**3g**)	Amylose-2[a]	85:15	10.2	(R_P,R_C)	24.5	(S_P,R_C)

[a] Phenomenex Lux ® 5µm Amylose-2 column
[b] Phenomenex Lux ® 5µm Cellulose-2 column
[c] Phenomenex Lux ® 5µm Cellulose-1 column
[d] Kromasil ® 5-Amycoat column
[e] Phenomenex Lux ® 3µm Cellulose-4 column

(2-methylphenyl)-phenylphosphine oxide (**1a**)

Racemic

Peak #	Time [min]	Area [μV·s]	Height [μV]	Area [%]	Norm. Area [%]	BL	Area/Height [s]
1	20.757	9136905.13	286647.07	50.00	50.00	*BB	31.8751
2	22.476	9138218.12	278419.28	50.00	50.00	*BB	32.8218
		18275123.26	565066.35	100.00	100.00		

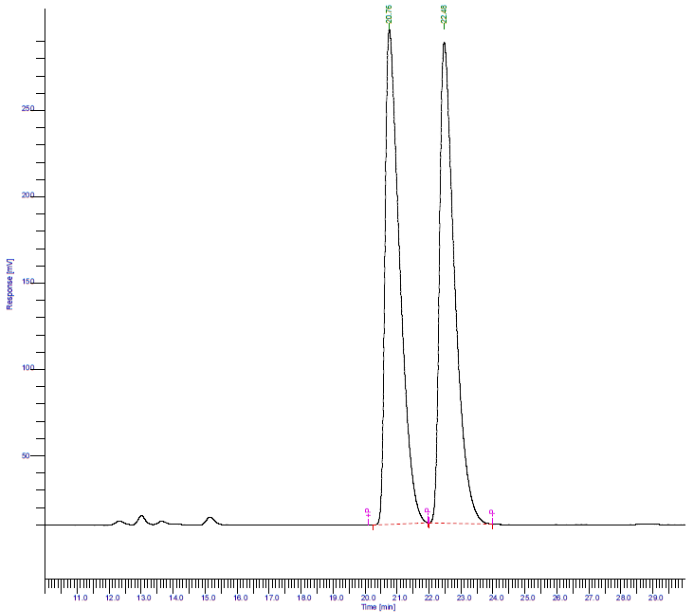

(*S*)-**1a** (Scheme 2; Table S1, Entry 1)

Peak #	Time [min]	Area [μV·s]	Height [μV]	Area [%]	Norm. Area [%]	BL	Area/Height [s]
1	21.286	141223.73	5477.40	0.82	0.82	*BB	25.7830
2	22.588	17045233.23	503359.05	99.18	99.18	*BB	33.8630
		17186456.96	508836.46	100.00	100.00		

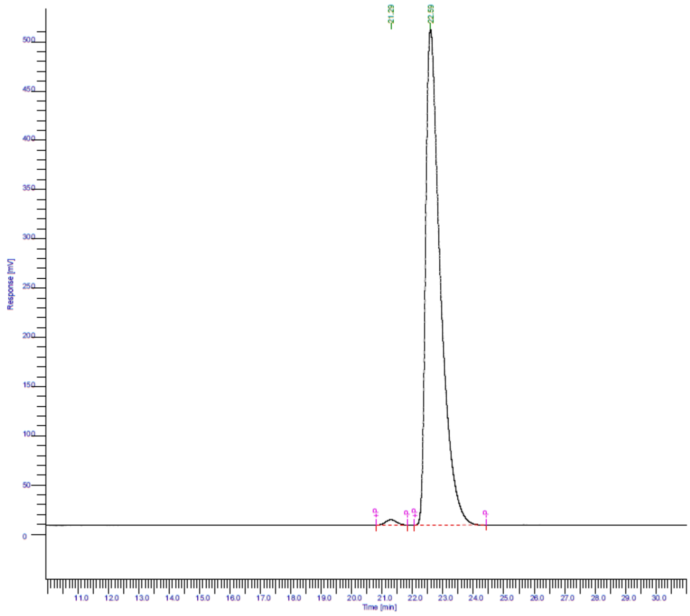

(3-methylphenyl)-phenylphosphine oxide (**1b**)

Racemic

Peak #	Time [min]	Area [µV·s]	Height [µV]	Area [%]	Norm. Area [%]	BL	Area/Height [s]
1	11.148	9159475.82	508003.74	50.05	50.05	*BB	18.0303
2	12.830	9142536.54	413145.24	49.95	49.95	*BB	22.1291
		18302012.36	921148.98	100.00	100.00		

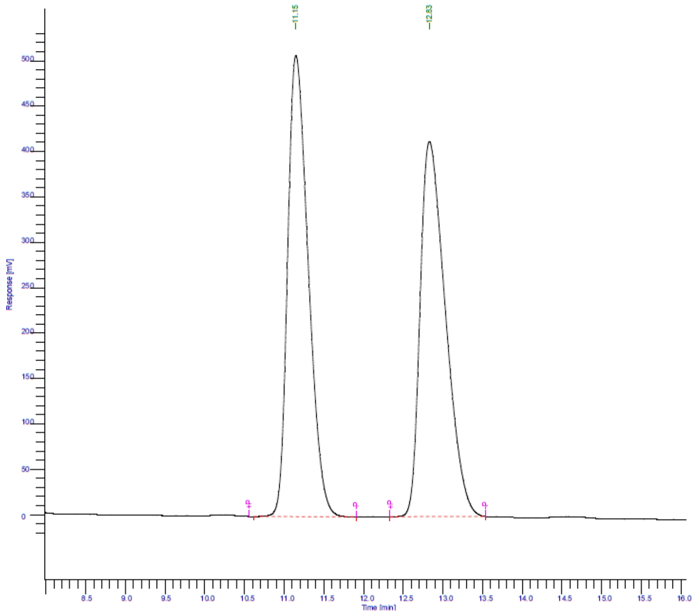

(−)-**1b** (Scheme 2; Table S3, Entry 3)

Peak #	Time [min]	Area [µV·s]	Height [µV]	Area [%]	Norm. Area [%]	BL	Area/Height [s]
1	11.476	3448528.83	197887.56	80.73	80.73	*BB	17.4267
2	13.284	823111.66	40427.16	19.27	19.27	*BB	20.3604
		4271640.49	238314.71	100.00	100.00		

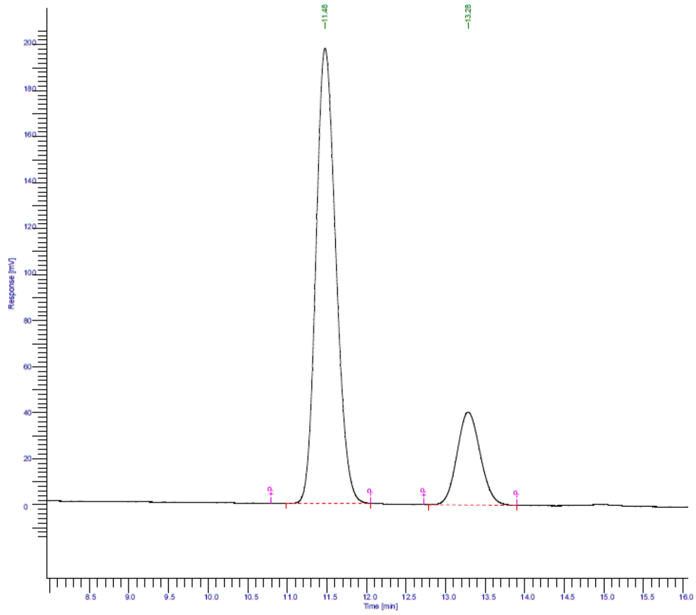

(4-methylphenyl)-phenylphosphine oxide (**1c**)

Racemic

Peak #	Time [min]	Area [µV·s]	Height [µV]	Area [%]	Norm. Area [%]	BL	Area/Height [s]
1	10.295	9748530.30	593761.87	49.99	49.99	*BB	16.4182
2	11.177	9753192.93	534519.03	50.01	50.01	*BB	18.2467
		19501723.24	1.13e+06	100.00	100.00		

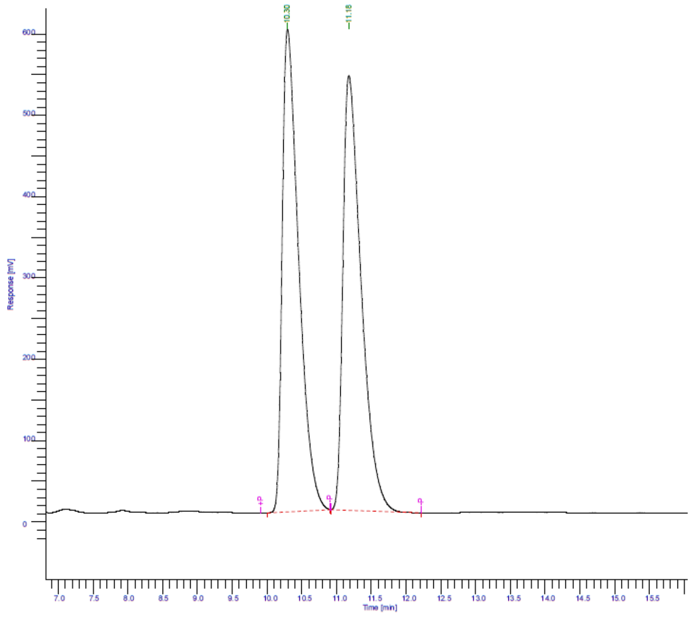

(−)-**1c** (Scheme 2; Table S2, Entry 5)

Peak #	Time [min]	Area [µV·s]	Height [µV]	Area [%]	Norm. Area [%]	BL	Area/Height [s]
1	10.382	4446333.14	286807.90	99.33	99.33	*BB	15.5028
2	11.343	30108.23	2034.44	0.67	0.67	*BB	14.7993
		4476441.38	288842.34	100.00	100.00		

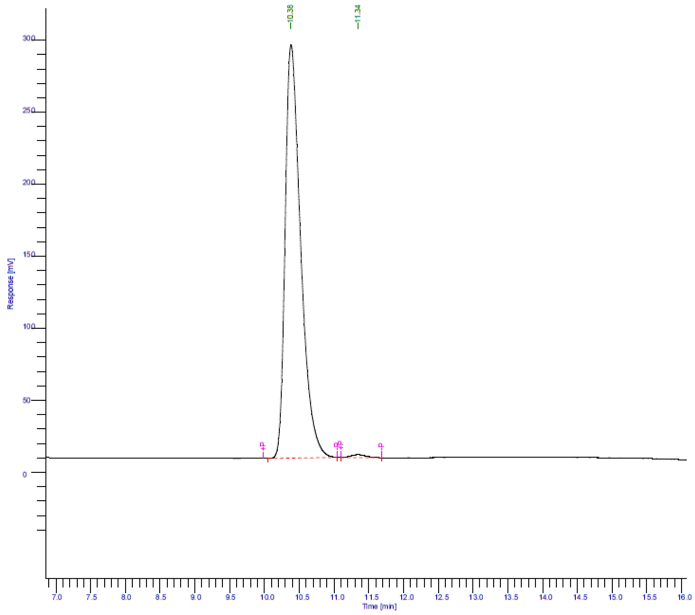

(3-trifuoromethylphenyl)-phenylphosphine oxide (**1e**)

Racemic

Peak #	Time [min]	Area [μV·s]	Height [μV]	Area [%]	Norm. Area [%]	BL	Area/Height [s]
1	6.738	2636599.53	262514.34	49.49	49.49	*BB	10.0436
2	8.699	2691269.24	192497.13	50.51	50.51	*BB	13.9808
		5327868.77	455011.46	100.00	100.00		

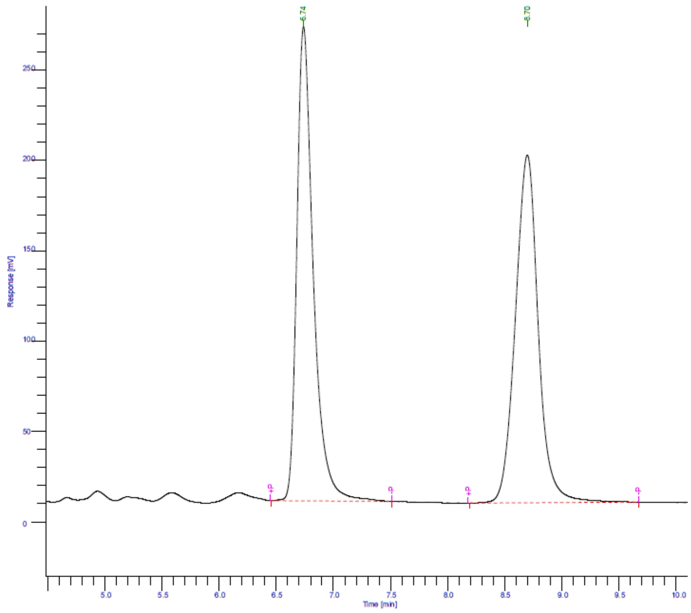

(+)-**1e** (Scheme 2; Table S3, Entry 11)

Peak #	Time [min]	Area [μV·s]	Height [μV]	Area [%]	Norm. Area [%]	BL	Area/Height [s]
1	6.730	4349035.65	424839.94	89.72	89.72	*BB	10.2369
2	8.687	498120.91	37404.44	10.28	10.28	*BB	13.3172
		4847156.55	462244.37	100.00	100.00		

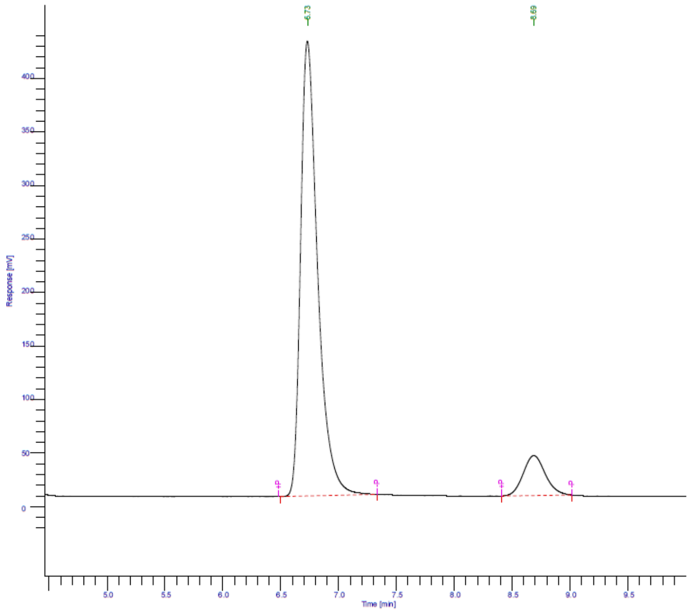

(4-trifuoromethylphenyl)-phenylphosphine oxide (**1f**)

Racemic

Peak #	Time [min]	Area [µV·s]	Height [µV]	Area [%]	Norm. Area [%]	BL	Area/Height [s]
1	6.531	1884579.88	201219.13	49.86	49.86	*BB	9.3658
2	8.339	1895084.49	144618.98	50.14	50.14	*BB	13.1040
		3779664.37	345838.11	100.00	100.00		

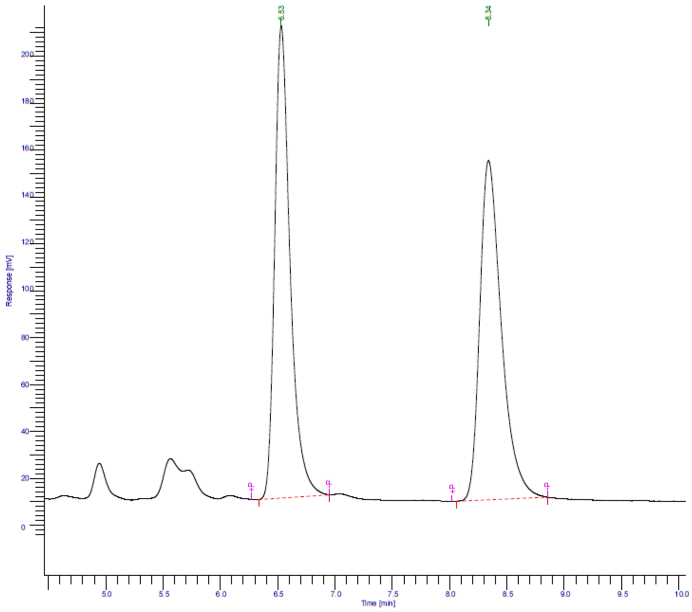

(+)-**1f** (Scheme 2; Table S3, Entry 12)

Peak #	Time [min]	Area [µV·s]	Height [µV]	Area [%]	Norm. Area [%]	BL	Area/Height [s]
1	6.442	6768900.37	685187.15	99.74	99.74	*BB	9.8789
2	8.240	17939.98	1455.61	0.26	0.26	*BB	12.3247
		6786840.34	686642.76	100.00	100.00		

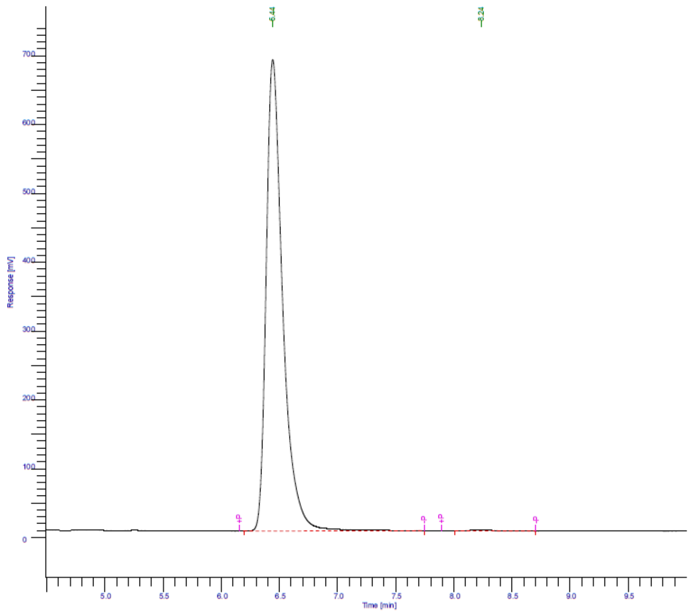

(2-methoxylphenyl)-phenylphosphine oxide (**1g**)

Racemic

Peak #	Time [min]	Area [μV·s]	Height [μV]	Area [%]	Norm. Area [%]	BL	Area/Height [s]
1	10.810	8782304.23	486920.14	49.82	49.82	*BB	18.0364
2	15.395	8846850.67	358610.06	50.18	50.18	*BB	24.6698
		17629154.89	845530.20	100.00	100.00		

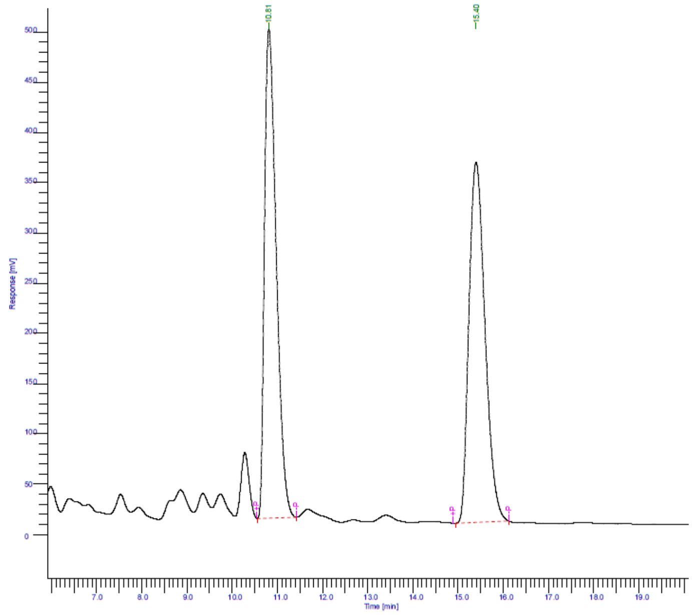

(*R*)-**1g** (Scheme 2; Table S2, Entry 13)

Peak #	Time [min]	Area [μV·s]	Height [μV]	Area [%]	Norm. Area [%]	BL	Area/Height [s]
1	11.009	750437.63	46157.07	16.46	16.46	*BB	16.2583
2	15.443	3808714.59	162049.48	83.54	83.54	*BB	23.5034
		4559152.22	208206.54	100.00	100.00		

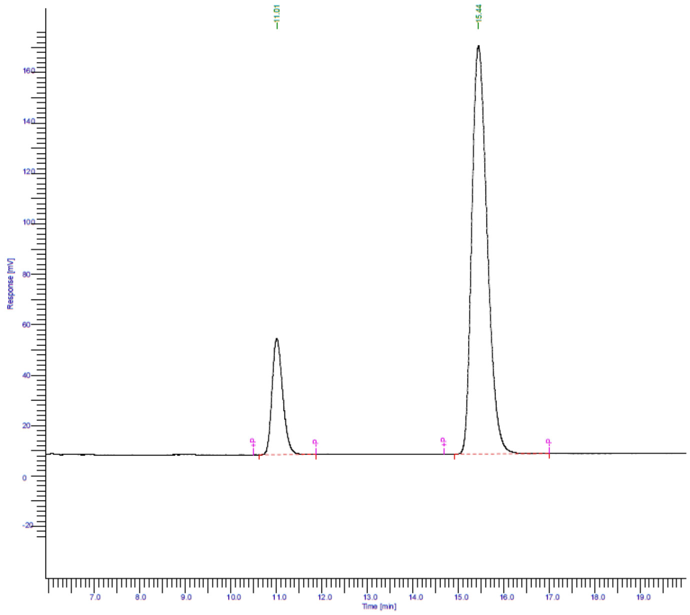

Absolute configuration of (2-methoxylphenyl)-phenylphosphine oxide (**1g**) was assigned using the same chiral stationary phase as reported in the literature [(S_P) enantiomer was reported to elute first):[2] Daicel Chiralpak AS-H column; hexane/2-PrOH (30:70) eluent; 0.3 mL/min flow rate; 20°C column temperature.

Racemic

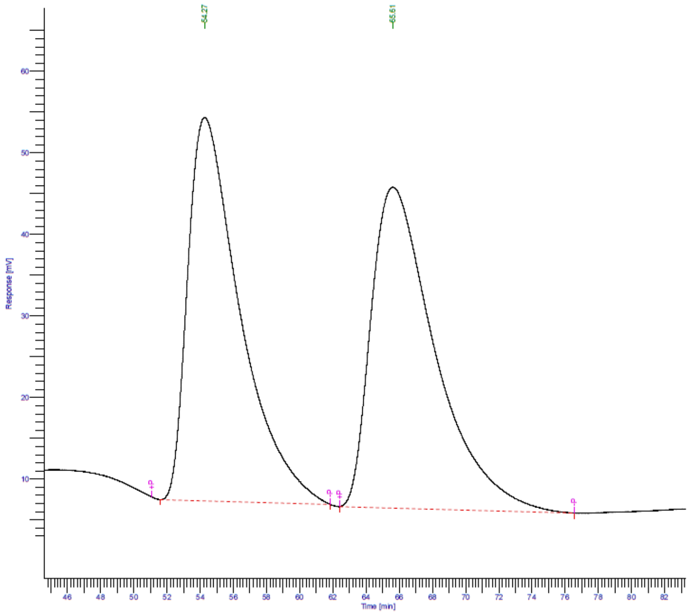

(*R*)-**1g**

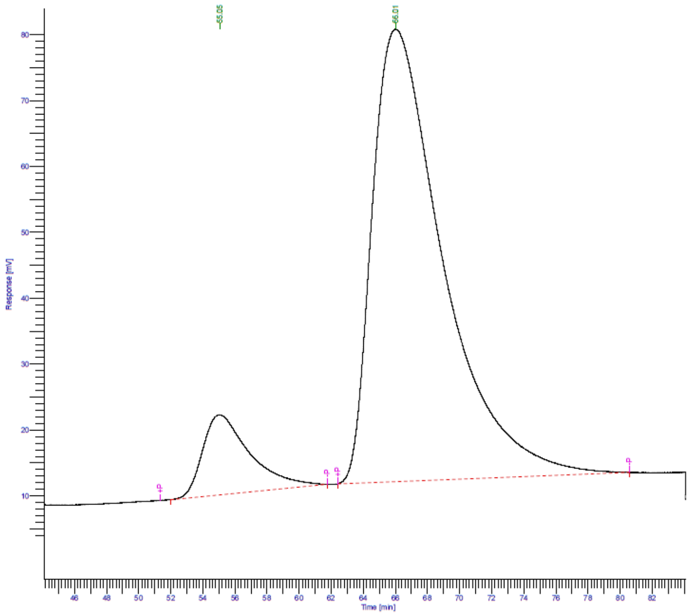

(2-phenylphenyl)-phenylphosphine oxide (**1h**)

Racemic

Peak #	Time [min]	Area [µV·s]	Height [µV]	Area [%]	Norm. Area [%]	BL	Area/Height [s]
1	9.956	6491411.31	432188.74	49.72	49.72	*BB	15.0199
2	11.948	6563251.67	348016.49	50.28	50.28	*BB	18.8590
		13054662.99	780205.23	100.00	100.00		

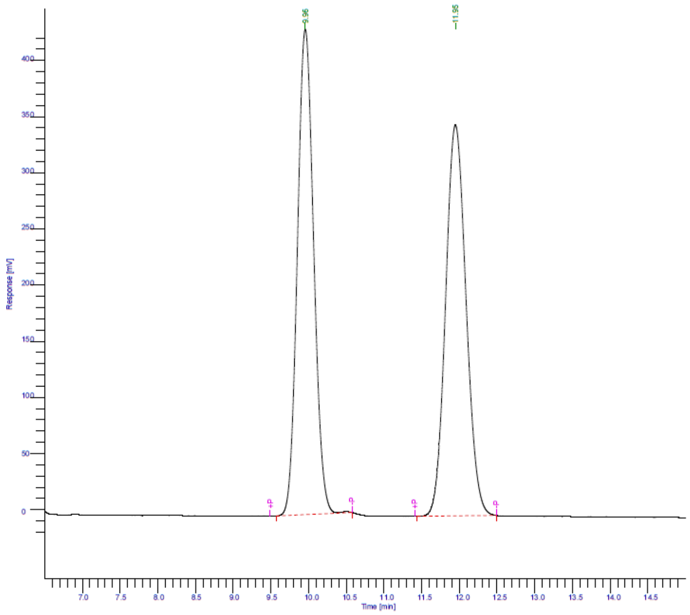

(*S*)-**1h** (Scheme 2; Table S3, Entry 16)

Peak #	Time [min]	Area [µV·s]	Height [µV]	Area [%]	Norm. Area [%]	BL	Area/Height [s]
1	9.777	6768812.90	439585.19	30.23	30.23	*BB	15.3982
2	11.763	15622212.27	792013.32	69.77	69.77	*BB	19.7247
		22391025.18	1.23e+06	100.00	100.00		

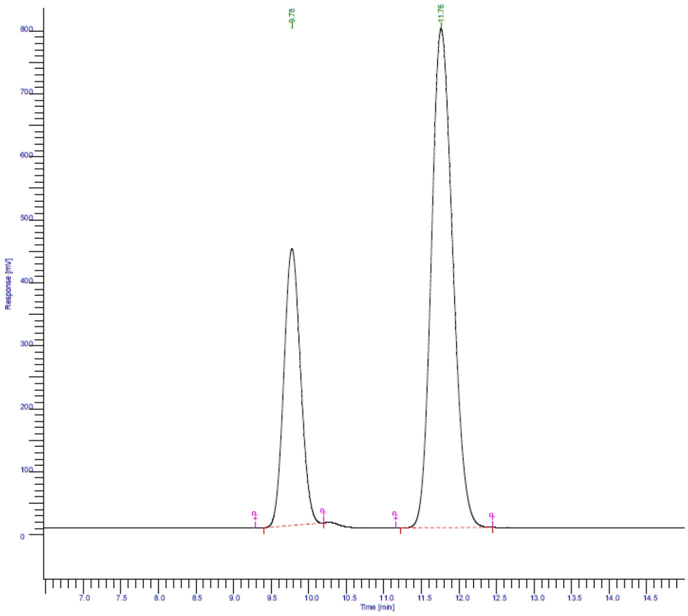

Absolute configuration of (2-phenylphenyl)-phenylphosphine oxide (**1h**) was assigned using a column containing the same chiral selector as the one reported in the literature [(S_P) enantiomer was reported to elute first]:[3] Kromasil® 5-Amycoat column; hexane/2-PrOH (90:10) eluent; 0.8 mL/min flow rate; 20°C column temperature.

Racemic

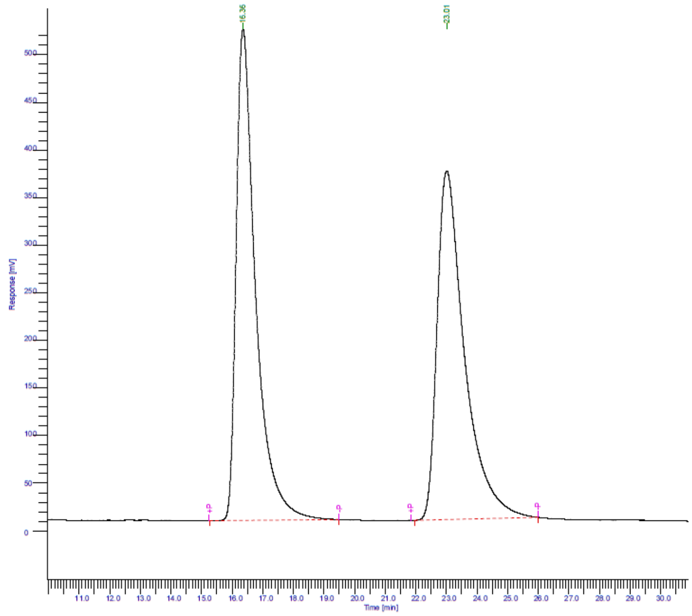

(*S*)-**1h**

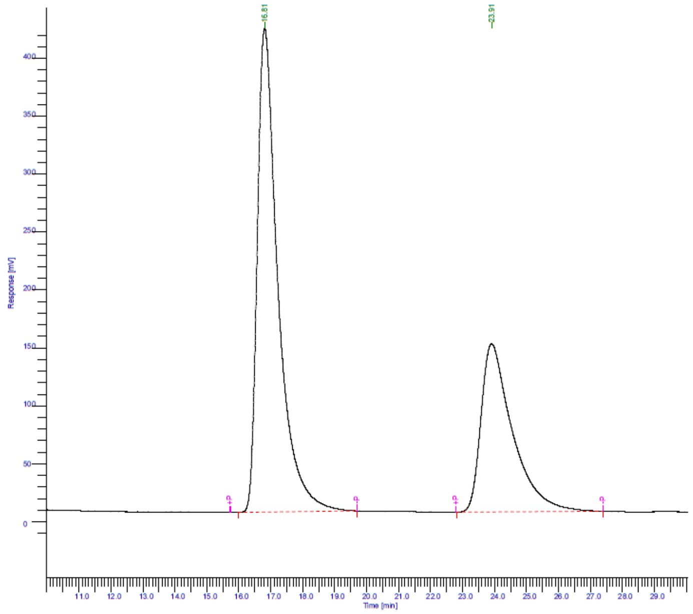

(1-napthyl)-phenylphosphine oxide (**1i**)

Racemic

Peak #	Time [min]	Area [µV·s]	Height [µV]	Area [%]	Norm. Area [%]	BL	Area/Height [s]
1	8.878	21106446.41	1.48e+06	50.04	50.04	BB	14.3074
2	9.753	21075999.74	1.32e+06	49.96	49.96	*BB	16.0187
		42182446.14	2.79e+06	100.00	100.00		

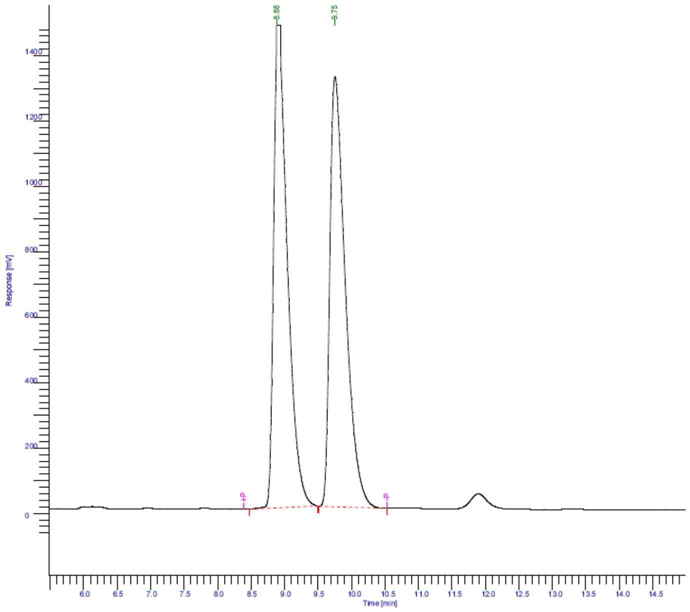

(*R*)-**1i** (Scheme 2; Table S3, Entry 19)

Peak #	Time [min]	Area [µV·s]	Height [µV]	Area [%]	Norm. Area [%]	BL	Area/Height [s]
1	9.012	6447467.63	478926.25	91.58	91.58	*BB	13.4623
2	9.933	592522.47	42278.49	8.42	8.42	*BB	14.0148
		7039990.09	521204.74	100.00	100.00		

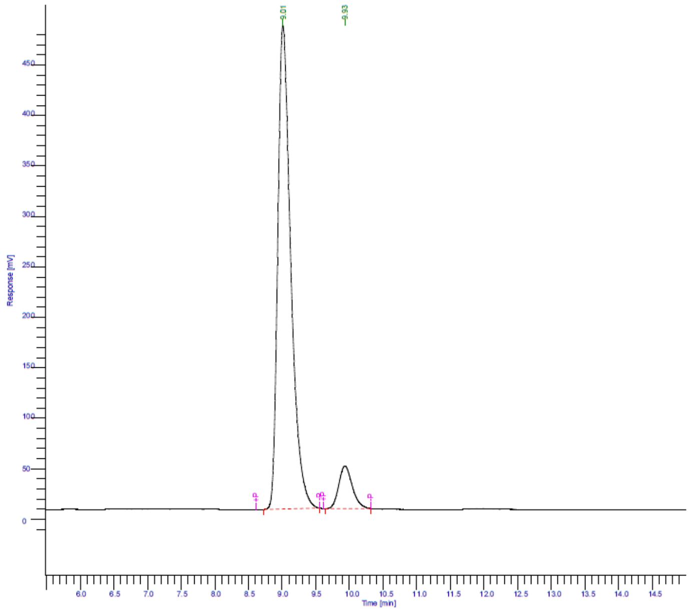

benzyl-phenylphosphine oxide (**1j**)

Racemic

Peak #	Time [min]	Area [µV·s]	Height [µV]	Area [%]	Norm. Area [%]	BL	Area/Height [s]
1	9.724	4010569.24	265936.78	50.15	50.15	*BB	15.0809
2	16.117	3986806.14	154671.40	49.85	49.85	*BB	25.7760
		7997375.38	420608.18	100.00	100.00		

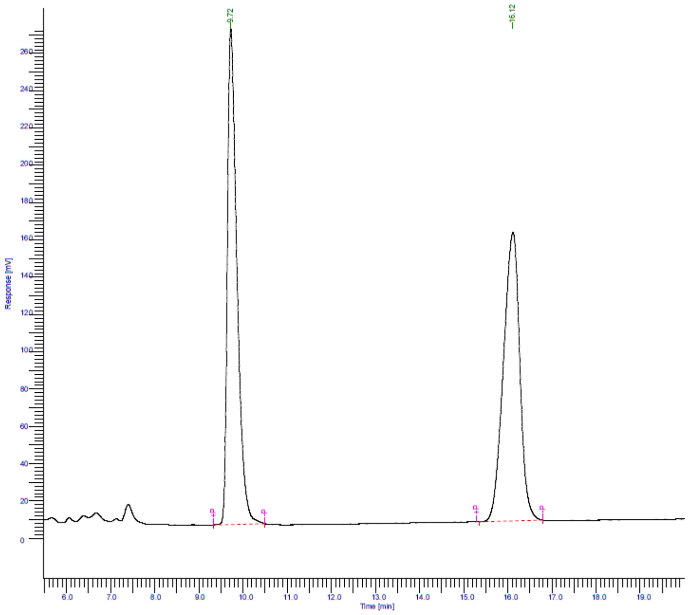

(*S*)-**1j** (Scheme 2; Table S2, Entry 20)

Peak #	Time [min]	Area [µV·s]	Height [µV]	Area [%]	Norm. Area [%]	BL	Area/Height [s]
1	9.702	1972768.26	137980.91	93.68	93.68	*BB	14.2974
2	15.894	133048.58	6032.47	6.32	6.32	*BB	22.0554
		2105816.83	144013.38	100.00	100.00		

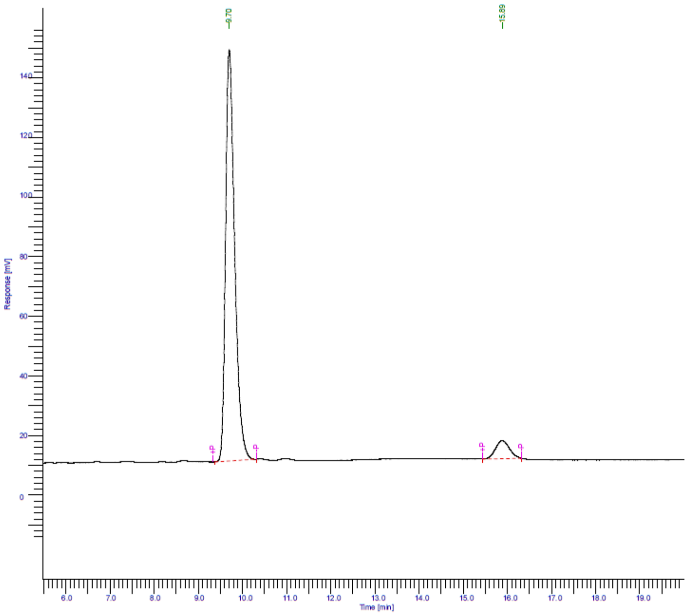

methyl-phenylphosphine oxide (**1k**)

Racemic

Peak #	Time [min]	Area [µV·s]	Height [µV]	Area [%]	Norm. Area [%]	BL	Area/Height [s]
1	9.075	3422416.89	270595.07	49.95	49.95	*BB	12.6477
2	10.814	3429276.23	217175.12	50.05	50.05	*BB	15.7904
		6851693.12	487770.20	100.00	100.00		

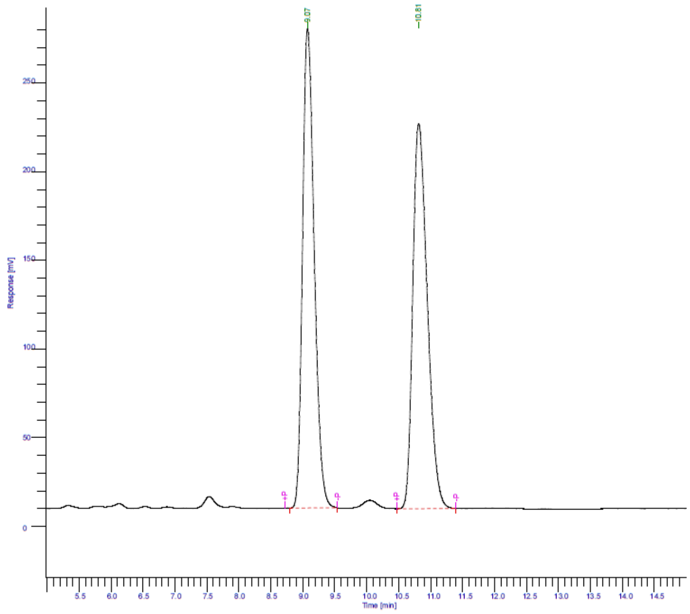

(*R*)-**1k** (Scheme 2; Table S2, Entry 22)

Peak #	Time [min]	Area [µV·s]	Height [µV]	Area [%]	Norm. Area [%]	BL	Area/Height [s]
1	9.100	10942.90	907.03	0.62	0.62	*BB	12.0645
2	10.831	1744784.75	114282.92	99.38	99.38	*BB	15.2672
		1755727.65	115189.95	100.00	100.00		

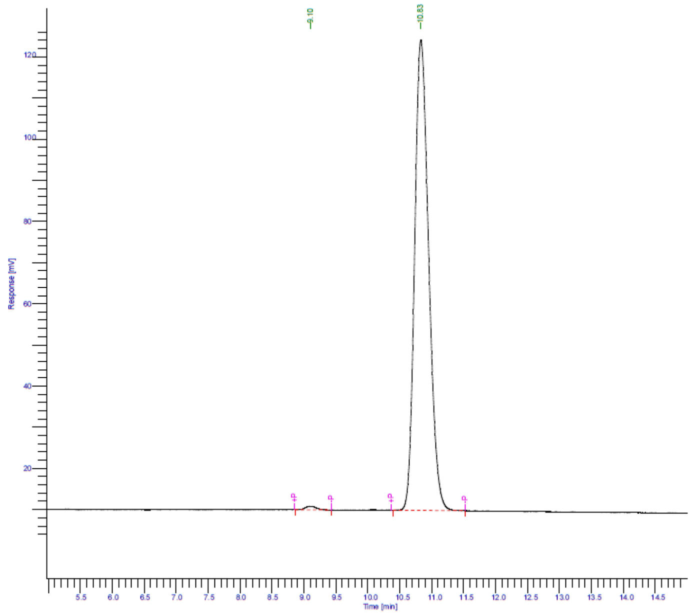

butyl-phenylphosphine oxide (**1l**)

Racemic

Peak #	Time [min]	Area [µV·s]	Height [µV]	Area [%]	Norm. Area [%]	BL	Area/Height [s]
1	7.118	4513961.28	415914.08	50.00	50.00	*BB	10.8531
2	7.777	4513764.43	389821.96	50.00	50.00	*BB	11.5790
		9027725.71	805736.04	100.00	100.00		

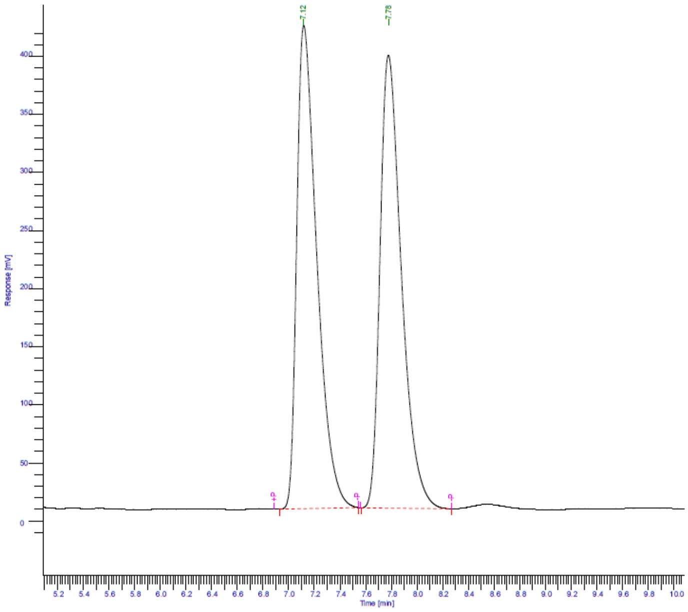

(*S*)-**1l** (Scheme 2; Table S3, Entry 25)

Peak #	Time [min]	Area [µV·s]	Height [µV]	Area [%]	Norm. Area [%]	BL	Area/Height [s]
1	7.103	2828381.50	267894.34	72.34	72.34	*BB	10.5578
2	7.772	1081469.30	97804.95	27.66	27.66	*BB	11.0574
		3909850.80	365699.29	100.00	100.00		

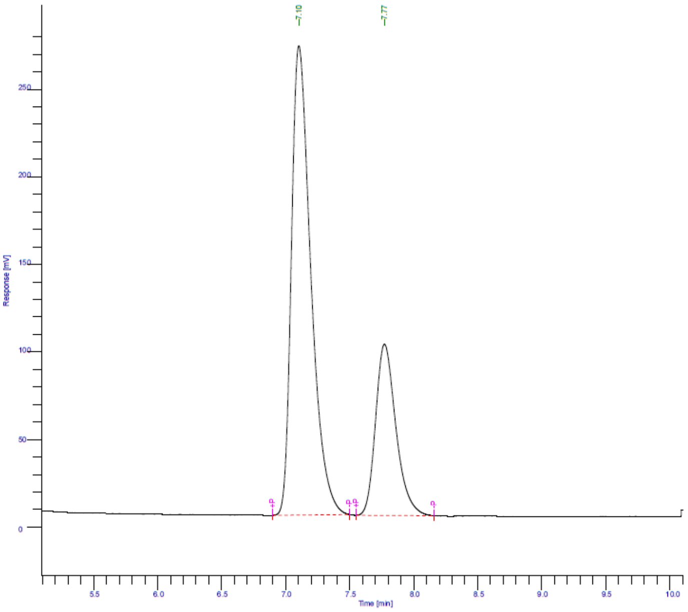

tert-butyl-phenylphosphine oxide (**1m**)

Racemic

Peak #	Time [min]	Area [µV·s]	Height [µV]	Area [%]	Norm. Area [%]	BL	Area/Height [s]
1	8.795	3225669.70	144652.28	49.85	49.85	*BB	22.2995
2	11.851	3245676.45	100289.21	50.15	50.15	*BB	32.3632
		6471346.15	244941.49	100.00	100.00		

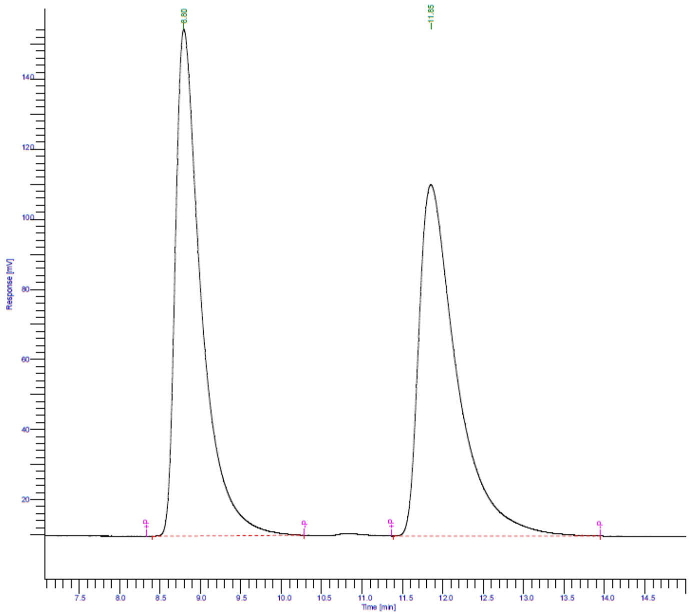

(*R*)-**1m** (Scheme 2; Table S2, Entry 25)

Peak #	Time [min]	Area [µV·s]	Height [µV]	Area [%]	Norm. Area [%]	BL	Area/Height [s]
1	8.878	29649.01	1708.47	0.96	0.96	*BB	17.3541
2	11.835	3043695.39	97592.35	99.04	99.04	*BB	31.1878
		3073344.40	99300.82	100.00	100.00		

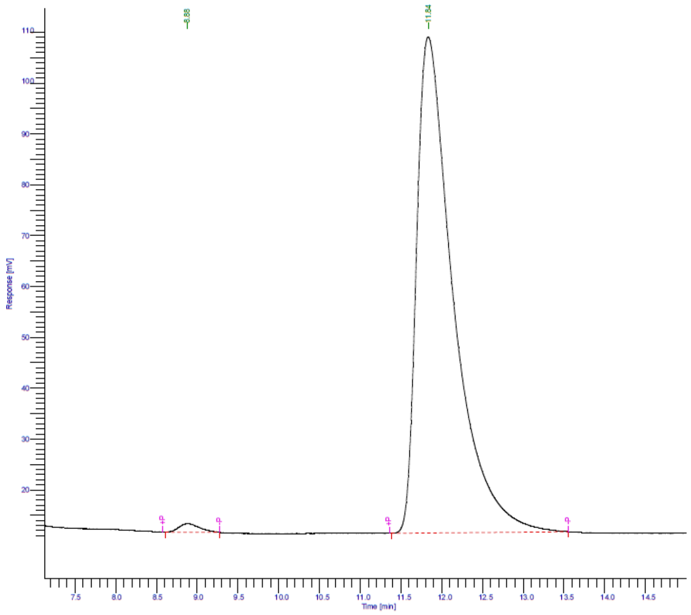

cyclohexyl-phenylphosphine oxide (**1n**)

Racemic

Peak #	Time [min]	Area [µV·s]	Height [µV]	Area [%]	Norm. Area [%]	BL	Area/Height [s]
1	10.007	3828821.57	239506.74	49.89	49.89	*BB	15.9863
2	19.716	3845502.66	91286.37	50.11	50.11	*BB	42.1257
		7674324.22	330793.12	100.00	100.00		

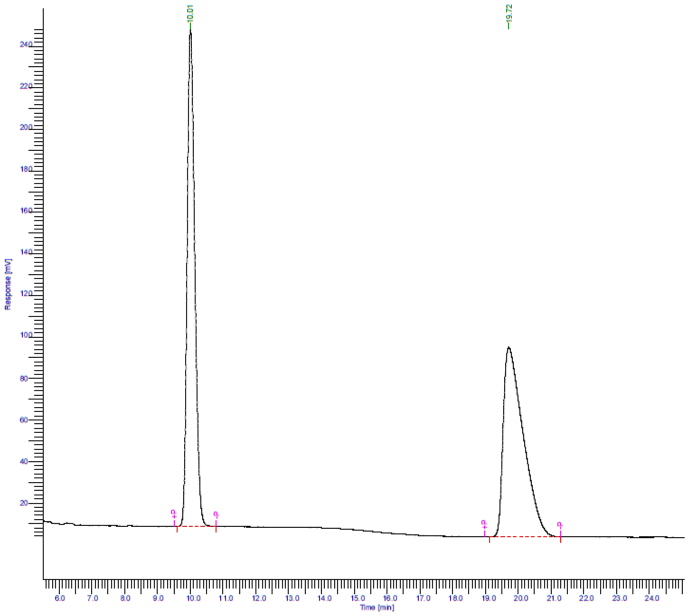

(*S*)-**1n** (Scheme 2; Table S3, Entry 30)

Peak #	Time [min]	Area [µV·s]	Height [µV]	Area [%]	Norm. Area [%]	BL	Area/Height [s]
1	10.042	3380227.14	212308.16	96.06	96.06	*BB	15.9213
2	20.414	138482.11	4130.25	3.94	3.94	*BB	33.5287
		3518709.25	216438.41	100.00	100.00		

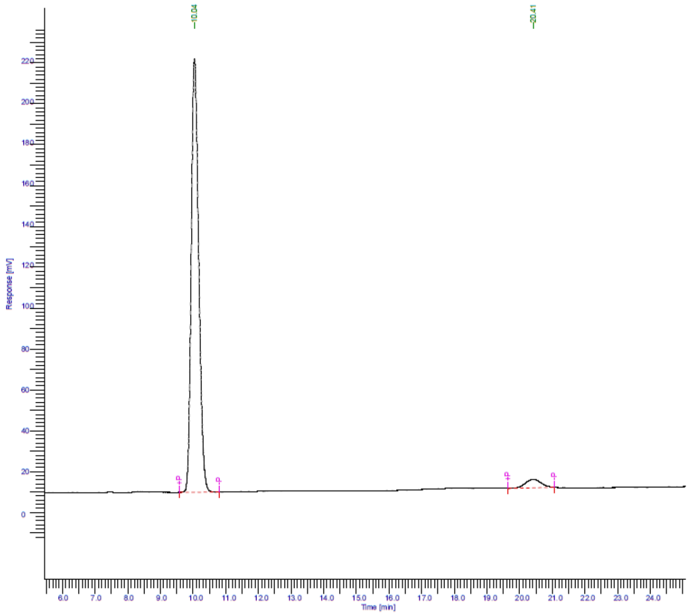

Absolute configuration of cyclohexyl-phenylphosphine oxide (**1n**) was assigned using a column containing the same chiral selector as the one reported in the literature [(S_P) enantiomer was reported to elute first]:[3] Kromasil® 5-Amycoat column; hexane/2-PrOH (90:10) eluent; 0.8 mL/min flow rate; 20°C column temperature.

Racemic

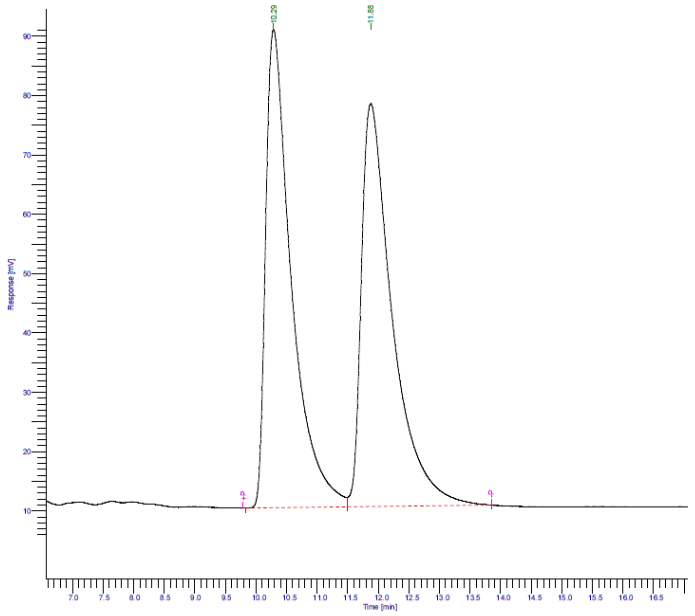

(*S*)-**1n**

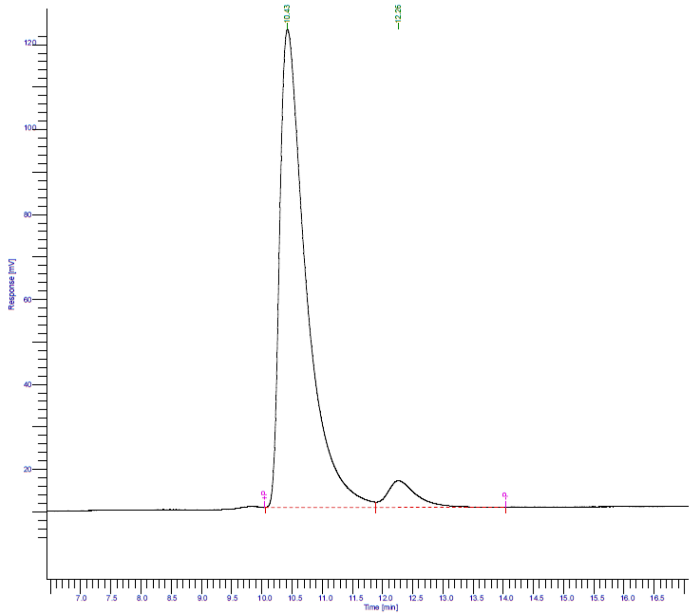

methyl-(2-methylphenyl)-phenylphosphine oxide (**3a**)

Racemic

Peak #	Time [min]	Area [µV·s]	Height [µV]	Area [%]	Norm. Area [%]	BL	Area/Height [s]
1	10.887	1594660.38	59538.03	50.38	50.38	*BB	26.7839
2	13.474	1570319.45	48819.61	49.62	49.62	*BB	32.1658
		3164979.83	108357.64	100.00	100.00		

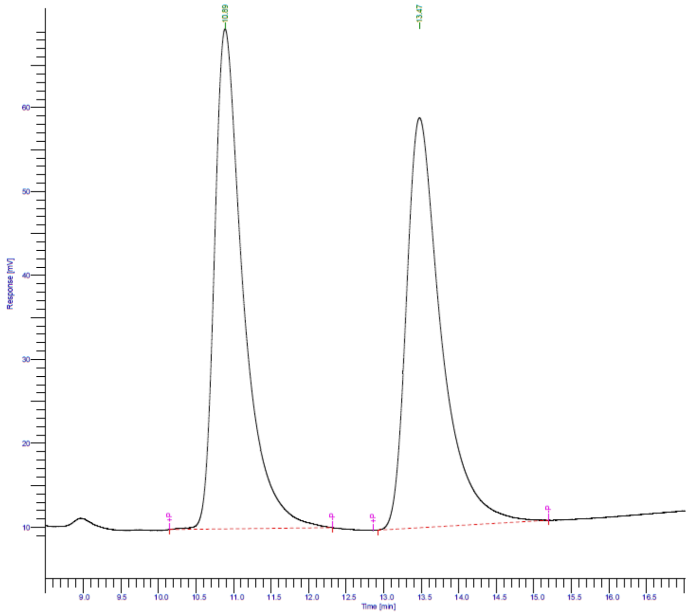

(*S*)-**3a** (Scheme 4)

Peak #	Time [min]	Area [µV·s]	Height [µV]	Area [%]	Norm. Area [%]	BL	Area/Height [s]
1	10.948	6559969.38	235443.10	98.96	98.96	*BB	27.8622
2	13.610	69237.40	3027.61	1.04	1.04	*BB	22.8686
		6629206.78	238470.71	100.00	100.00		

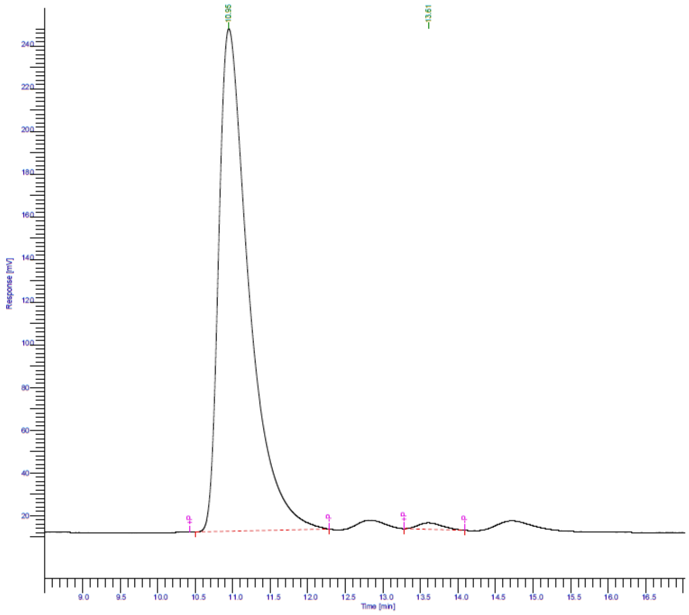

ethyl-(2-methylphenyl)-phenylphosphine oxide (**3b**)

Racemic

Peak #	Time [min]	Area [µV·s]	Height [µV]	Area [%]	Norm. Area [%]	BL	Area/Height [s]
1	8.385	3929133.34	179328.92	48.73	48.73	*BB	21.9102
2	12.586	4134360.88	120634.41	51.27	51.27	*BB	34.2718
		8063494.22	299963.33	100.00	100.00		

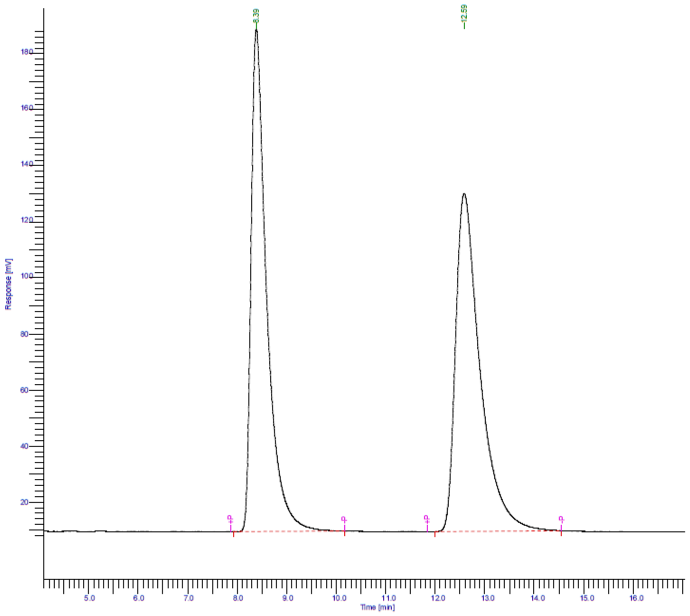

(*S*)-**3b** (Scheme 4)

Peak #	Time [min]	Area [µV·s]	Height [µV]	Area [%]	Norm. Area [%]	BL	Area/Height [s]
1	8.531	8391087.51	360874.94	97.68	97.68	*BB	23.2521
2	12.967	199589.04	6545.56	2.32	2.32	*BB	30.4923
		8590676.56	367420.49	100.00	100.00		

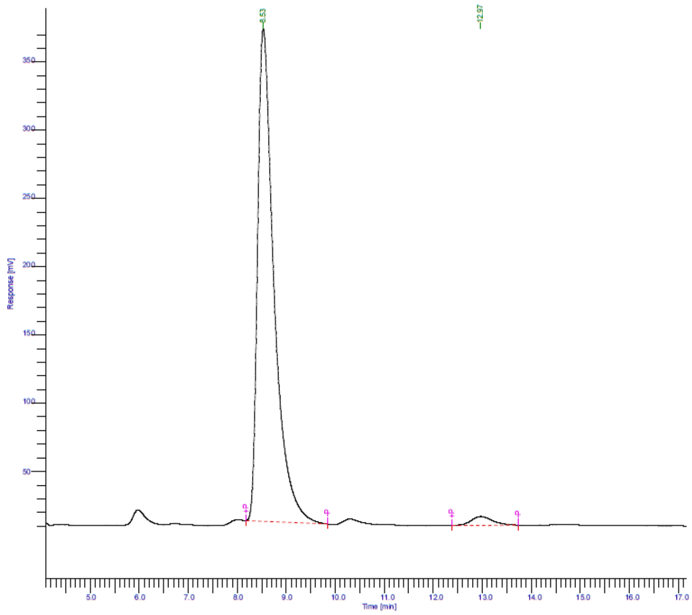

benzyl-(2-methylphenyl)-phenylphosphine oxide (**3c**)

Racemic

Peak #	Time [min]	Area [µV·s]	Height [µV]	Area [%]	Norm. Area [%]	BL	Area/Height [s]
1	17.191	2439306.72	86468.41	49.97	49.97	*BB	28.2104
2	24.325	2442487.21	59778.49	50.03	50.03	*BB	40.8590
		4881793.93	146246.90	100.00	100.00		

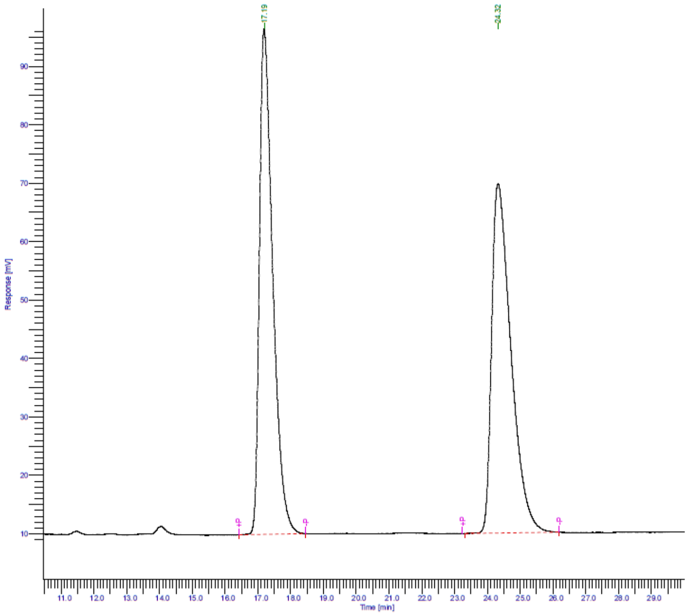

(*S*)-**3c** (Scheme 4)

Peak #	Time [min]	Area [µV·s]	Height [µV]	Area [%]	Norm. Area [%]	BL	Area/Height [s]
1	16.989	7863071.14	239549.57	97.98	97.98	*BB	32.8244
2	24.773	162421.59	4770.64	2.02	2.02	*BB	34.0460
		8025492.73	244320.21	100.00	100.00		

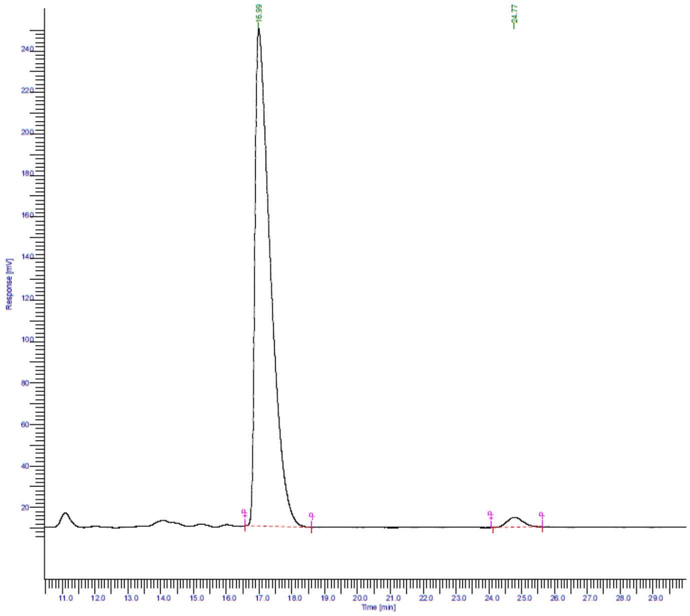

(1-napthyl)-(2-methylphenyl)-phenylphosphine oxide (**3d**)

Racemic

Peak #	Time [min]	Area [µV·s]	Height [µV]	Area [%]	Norm. Area [%]	BL	Area/Height [s]
1	8.713	3506444.64	156084.31	50.44	50.44	*BB	22.4651
2	11.097	3445294.04	121125.63	49.56	49.56	BB	28.4440
		6951738.68	277209.94	100.00	100.00		

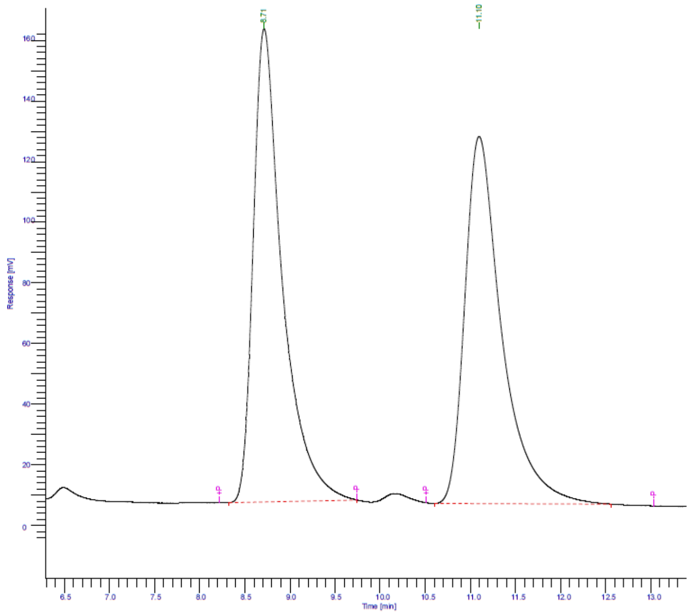

(*S*)-**3d** (Scheme 4)

Peak #	Time [min]	Area [µV·s]	Height [µV]	Area [%]	Norm. Area [%]	BL	Area/Height [s]
1	8.365	716357.26	26714.10	6.24	6.24	*BB	26.8157
2	11.033	10757835.03	306081.37	93.76	93.76	*BB	35.1470
		11474192.29	332795.47	100.00	100.00		

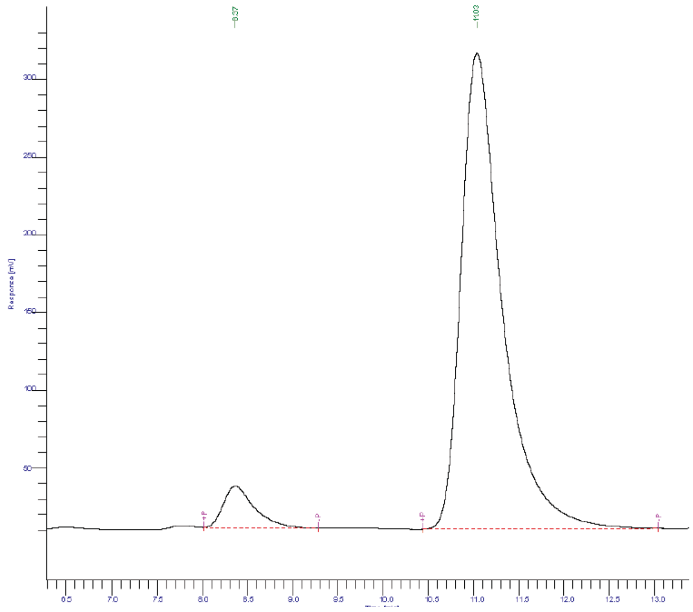

Hydroxymethyl-(2-methylphenyl)-phenylphosphine oxide (**3f**)

Racemic

Peak #	Time [min]	Area [µV·s]	Height [µV]	Area [%]	Norm. Area [%]	BL	Area/Height [s]
1	8.618	9216317.68	699955.21	49.83	49.83	BV	13.1670
2	9.218	9278943.03	611548.45	50.17	50.17	*VB	15.1729
		18495260.72	1.31e+06	100.00	100.00		

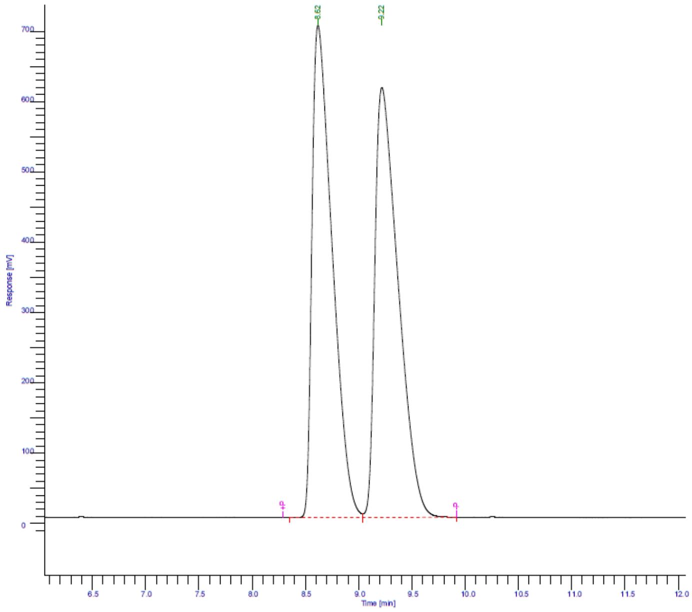

(*S*)-**3f** (Scheme 4)

Peak #	Time [min]	Area [µV·s]	Height [µV]	Area [%]	Norm. Area [%]	BL	Area/Height [s]
1	8.670	7959861.25	600184.27	97.98	97.98	BV	13.2624
2	9.409	164265.09	12807.13	2.02	2.02	*VB	12.8261
		8124126.34	612991.40	100.00	100.00		

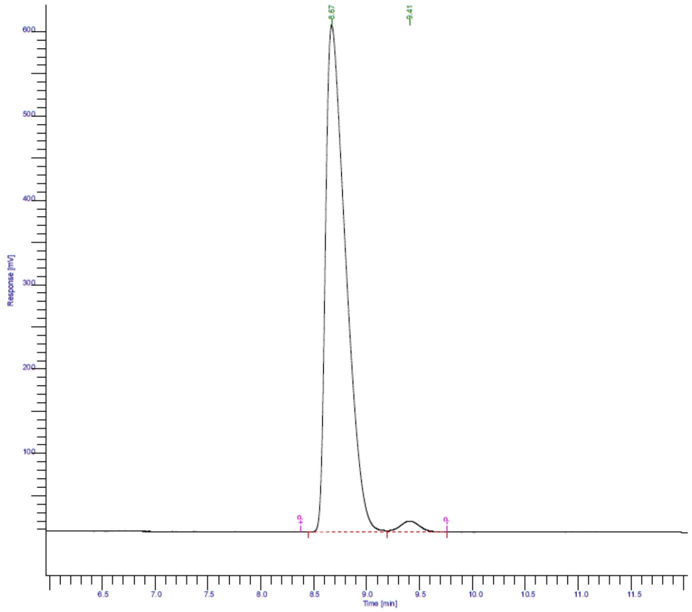

[(R_C)-hydroxy(phenyl)methyl]-(2-methylphenyl)-phenylphosphine oxide (**3g**)

P-racemic

Peak #	Time [min]	Area [µV·s]	Height [µV]	Area [%]	Norm. Area [%]	BL	Area/Height [s]
1	10.154	5915390.40	309971.33	50.22	50.22	*BB	19.0837
2	24.514	5864511.45	103621.09	49.78	49.78	*BB	56.5957
		11779901.85	413592.42	100.00	100.00		

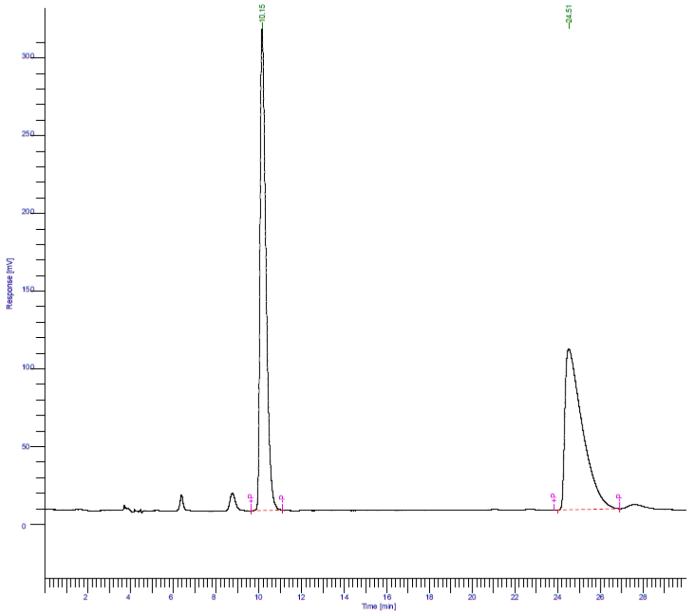

(S_P,R_C)-**3g** (Scheme 4)

Peak #	Time [min]	Area [µV·s]	Height [µV]	Area [%]	Norm. Area [%]	BL	Area/Height [s]
1	10.337	566.05	55.43	0.01	0.01	*BB	10.2116
2	24.286	10921110.66	168743.64	99.99	99.99	*BB	64.7201
		10921676.71	168799.07	100.00	100.00		

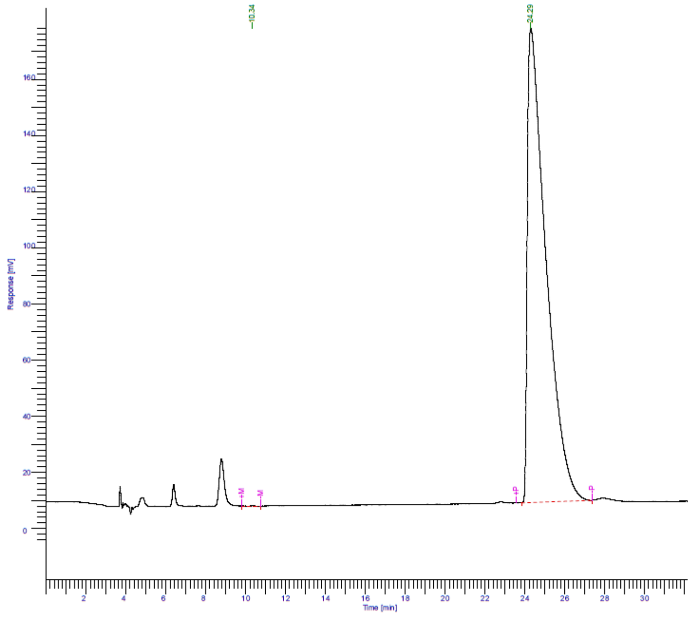

(*R*)-(2-methylphenyl)-phenylphosphothioic acid (**3e**)

The enantiomeric excess (*ee*) values of **3e** was determined by ^{31}P NMR using 5.0 mg (20 μmol) of the analyte, 4.8μL (30 μmol) (*S*)-napthylethylamine as CSA and 750 μL $CDCl_3$ as solvent.

^{31}P{^{1}H} NMR (202.5 MHz, $CDCl_3$): Racemic **3e** + (*S*)-napthylethylamine

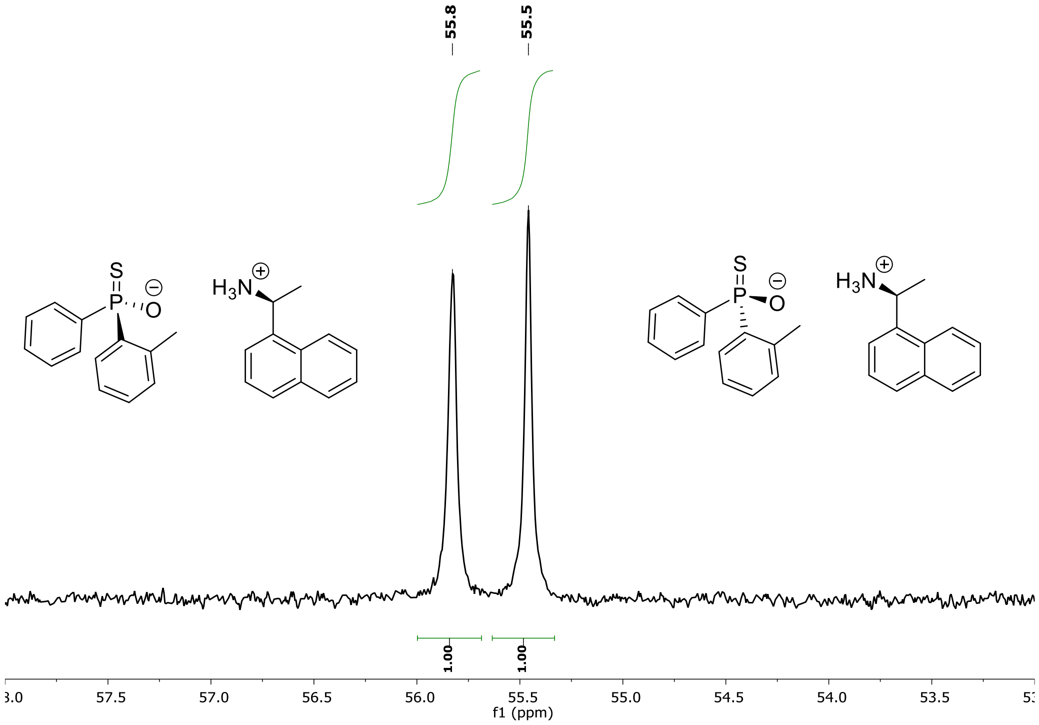

^{31}P{^{1}H} NMR (202.5 MHz, $CDCl_3$): (*R*)-**3e** + (*S*)-napthylethylamine

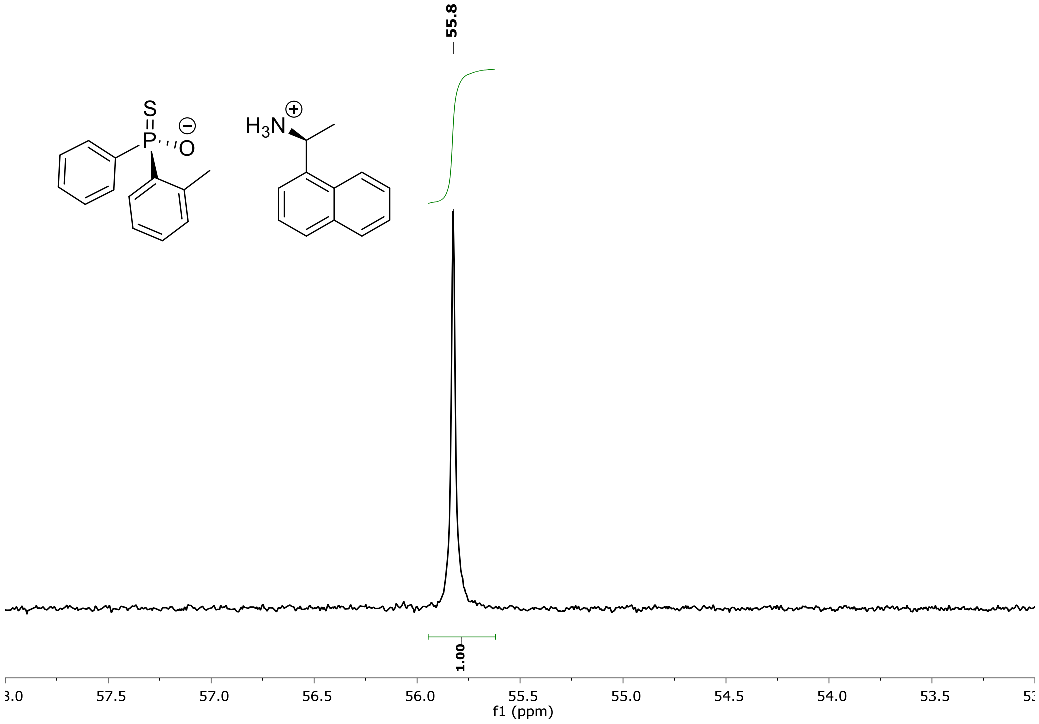

References

1 R. A. Sheldon, *Chirotechnology: Industrial Synthesis of Optically Active Compounds*, Marcel Dekker, New York, 1993.

2 Q. Xu, C.-Q. Zhao and L.-B. Han, *J. Am. Chem. Soc.*, **2008**, 130, 12648–12655.

3 L. Copey, L. Jean-Gérard, B. Andrioletti and E. Framery, *Tetrahedron Lett.*, **2016**, 57, 543–545.

4 R. K. Haynes, T.-L. L. Au-Yeung, W.-K. K. Chan, W.-L. L. Lam, Z.-Y. Y. Li, L.-L. L. Yeung, A. S. C. C. Chan, P. Li, M. Koen, C. R. Mitchell and S. C. Vonwiller, *Eur. J. Org. Chem.*, **2000**, 3205–3216.
